# Synthesis of Structural ADP-Ribose Analogues as Inhibitors for SARS-CoV-2 Macrodomain 1

**DOI:** 10.1021/acs.orglett.4c01792

**Published:** 2024-06-27

**Authors:** Koen J. Rijpkema, Marion Schuller, Miriam S. van der Veer, Sjoerd Rieken, Diego L. R. Chang, Pascal Balić, Alex Todorov, Hugo Minnee, Sven Wijngaarden, Isaac A. Matos, Nicolas C. Hoch, Jeroen D. C. Codée, Ivan Ahel, Dmitri V. Filippov

**Affiliations:** † Leiden Institute of Chemistry, Leiden University, Einsteinweg 55, 2333 CC Leiden, The Netherlands; ∥ Sir William Dunn School of Pathology, University of Oxford, South Parks Road, Oxford OX1 3RE, United Kingdom; § Departamento de Bioquímica, Instituto de Química, Universidade de Sao Paulo, Av. Prof. Lineu Prestes, 748, Cidade Universitária, Sao Paulo 055800-000, Brasil

## Abstract

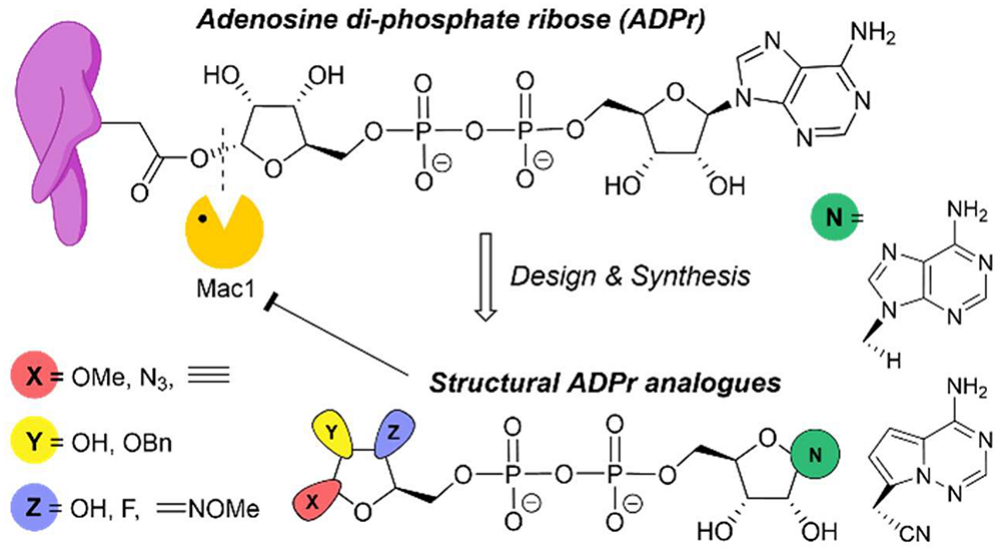

Protein adenosine diphosphate (ADP)-ribosylation is crucial for a proper immune response. Accordingly, viruses have evolved ADP-ribosyl hydrolases to remove these modifications, a prominent example being the SARS-CoV-2 NSP3 macrodomain, “Mac1”. Consequently, inhibitors are developed by testing large libraries of small molecule candidates, with considerable success. However, a relatively underexplored angle in design pertains to the synthesis of structural substrate mimics. Here, we present the synthesis and biophysical activity of novel adenosine diphosphate ribose (ADPr) analogues as SARS-CoV-2 NSP3 Mac1 inhibitors.

The COVID-19 pandemic caused by SARS-CoV-2 has prompted extensive research on different treatment possibilities of this disease.^1^ Various studies led to the discovery of promising protein targets for drug- or antibody-based therapies for SARS-CoV-2 infection like the coronavirus spike-protein,^2^ human receptor ACE2,^2^ RNA polymerase RdRp,^3^ and the macrodomain Mac1.^4^ Mac1 is a domain of a larger viral protein known as nonstructural protein (NSP3) and belongs to the family of macrodomains, which are the proteins that specifically bind the post-translational modification (PTM) adenosine diphosphate ribose (ADPr, Figure 1A).^5−8^ Mac1 not only binds ADPr but also hydrolyses the glycosidic bond between the distal ribose of ADPr and the side chain of the amino acid thus removing the PTM.^7,9^ The capability of Mac1 to cleave ADPr from the protein has evolved by the virus to counteract the host immune responses. Specifically, mammalian antiviral ADPribosyl-transferases such as PARP14 modify protein targets by attaching a single ADPr residue to an amino acid side chain in the protein. Such mono-ADP-ribosylation (MARylation) results in the induction of interferon and initiation of antiviral immune responses.^10,11^ Recent studies suggest that Mac1 reverses the PARP14-catalyzed MARylation by removing ADPr from the signaling proteins and thus abolishes the antiviral response of the host.^7^ Therefore, Mac1 inhibition could lead to reinstating the protective PARP-mediated immunologic function after infection.^12^ To probe this, various screening assays have been established for Mac1 inhibitor discovery and an array of different Mac1 inhibitors have been proposed over the past few years.^13−17^ A number of small molecule inhibitors of micromolar potency^13^ demonstrated encouraging Mac1 inhibitory properties. Notably, mimicking the natural substrate with ADPr analogues is a relatively underexplored approach for the development of Mac1 inhibitors but has recently led to highly potent Mac1 binders.^18^ Here, we report on such an approach to Mac1 inhibition by synthesizing advanced ADPr derivatives and evaluating their potential as inhibitors for Mac1. The key synthetic step is the construction of the pyrophosphate bridge using P(III)–P(V) phosphoramidite-based chemistry,^19−21^ which we adapted for ADP-ribose and extensively used through the years for the preparation of a variety of ADP-ribosylated biomolecules.^22−26^ In this work, we first coupled different 5′-*O*-phosphoryl ribosides as the P(V) components to a 5′-phosphoramidite of a protected adenosine as the P(III) component, generating a focused library of ADPr-mimics (Figure 1). After evaluating the binding potency to Mac1 of these first-generation mimics, we substituted the adenosine nucleoside for remdesivir using the P(III)–P(V) chemistry (Scheme 1) in two of the best binders from the ADPr library and obtained NDPr derivatives that demonstrate binding potency in the low nanomolar range.

**Figure 1:**
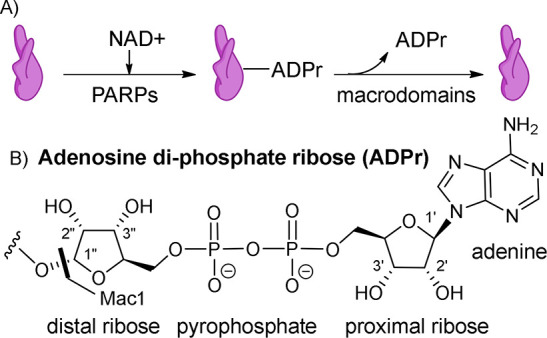
(A) Protein ADP-ribosylation. (B) ADP-ribose numbering.

**Scheme 1:**
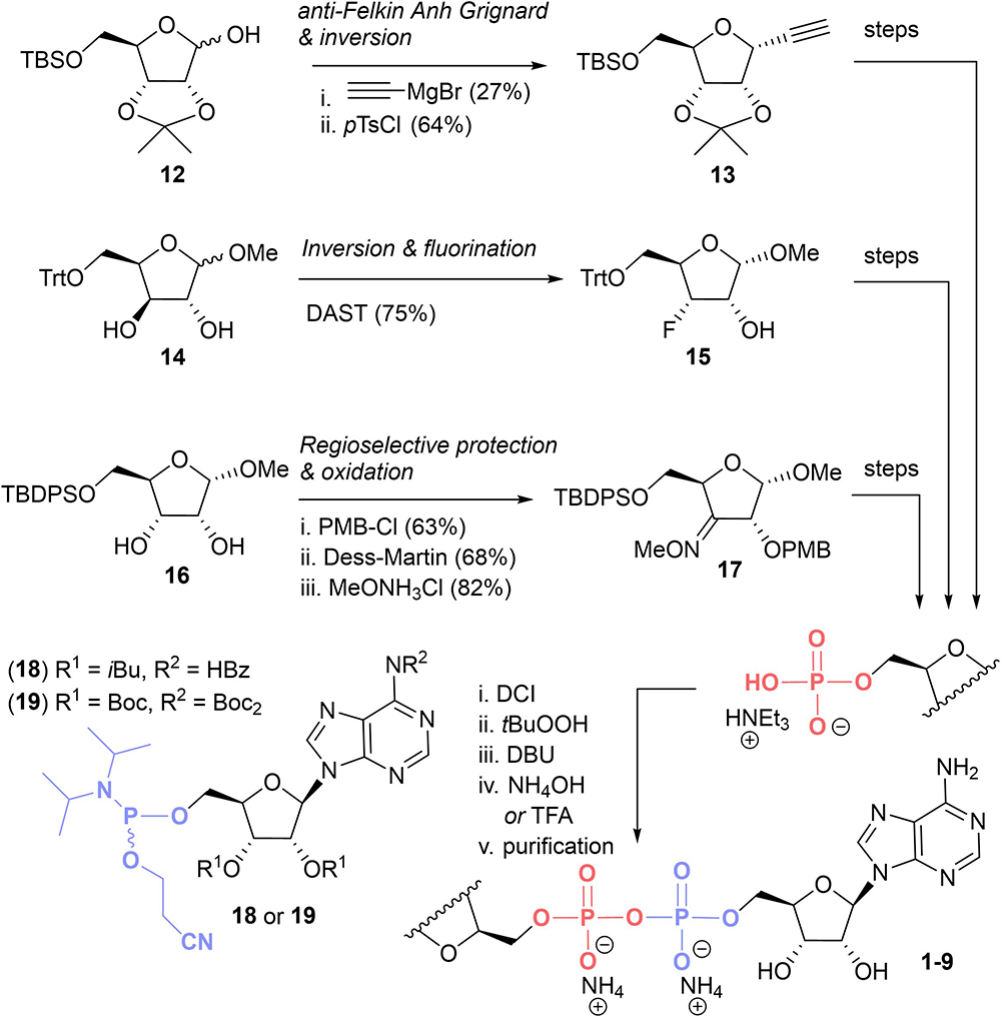
Key Steps Towards ADPr Analogues **5**, **8**, and **9** and General Synthetic Strategy

We set out to synthesize the ADPr derivatives with the distal ribose modified at three sites, the 1″ (anomeric), 2″, and 3″ positions, while keeping the ADP-part unchanged (Figure 1B, Table 1). The general synthetic strategy and the key steps toward the ADPr-mimics are depicted in Scheme 1, while the synthesis is detailed in Schemes S1–S6. We started with the synthesis and evaluation of α-*O*-methyl-ADPr **1** (Table 1, Scheme S1) that we compared with previously described^23^ β-*O*-methyl-ADPr derivative **2** (Table 1) since they are known to inhibit other macrodomains. As expected, the β-oriented ADPr **2**, in which the *O*-methyl would sterically interfere with the glycine-rich loop region, is a notably worse inhibitor for Mac1. Interestingly, installation of an α-azide at the anomeric center of the distal ribose^25^ increased the Mac1 inhibitory activity to the high nanomolar range (**3**, IC_50_ = 0.49 μM), a higher potency than native ADPr, which we used as reference (IC_50_ = 1.2 μM). Notably, the binding of compound **3** to Mac1 with submicromolar potency has been reported by Lin and co-workers and is in line with our findings.^18^ It is likely that the polarity of the azide group may allow additional interactions with the glycine-rich loop region (G46-G48) of Mac1 surrounding the 1″ position of the distal ribose, consequently increasing its affinity for Mac1 binding. The previously reported^25^ β-azide-ADPr **4** is a notably worse inhibitor (IC_50_ = 127 μM).

**Table 1:**
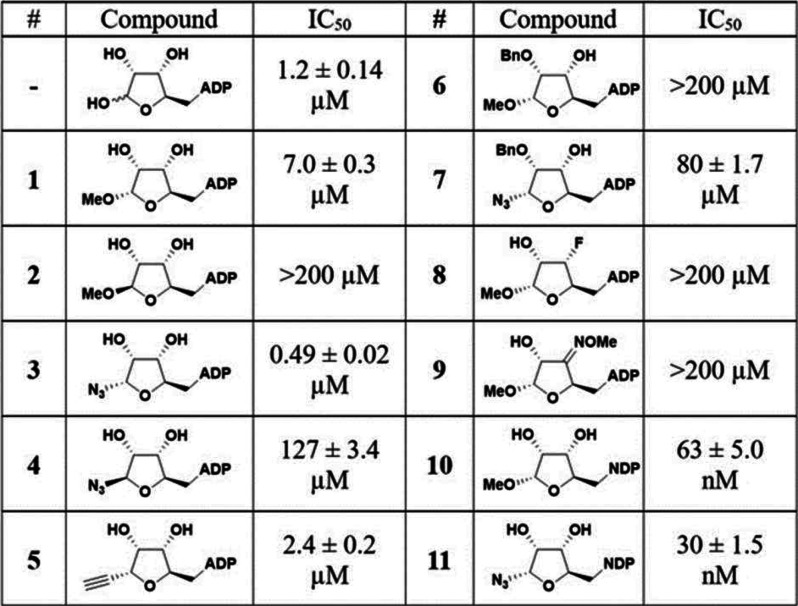
IC_50_ Values for ADPr Analogues

Because of the possible hydrolytic sensitivity of α-ribosylazide, a stable isostere bearing a linear substituent that mimics the anomeric azide was considered. To this end, we prepared ADPr-mimic **5** (Schemes 1 and S2). A key step in the synthesis of **5** was the establishment of the *C*-glycosidic bond in **13** that was installed through a stereoselective Grignard reaction on the C-1 aldehyde in the open-chain form of d-ribose derivative **12** (d.r. = 95:5). Interestingly, the protecting group choice is known to be a decisive factor in shaping the configuration of *C*-glycoside **13**. It has been reported that Felkin-Anh control gives the β-alkyne which occurs in the case of 4-methoxybenzyl (PMB) protection groups, while *anti*-Felkin-Anh control gives the corresponding α-alkyne in the case of *iso*-propylidene protection.^27^ Alkyne **13** was subsequently converted into the required 5′-phosphate, and then, the corresponding ADPr analogue **5** was produced via the P(III)–(PV) pyrophosphate coupling method applying 5′-phosphoramidite of adenosine as a P(III) component.^28^ Notably, Mac1 affinity for the *C*-glycoside **5** did not significantly diminish compared to the *N*- or *O*-linked glycosides **1** or **3** (2.4 μM), indicating that the glycosidic linkage is dispensable for binding.

With lead compounds **1** and **3** in hand, the 2″ and 3″ positions were modified next. These positions have been reported to participate in hydrogen bonding within the enzymatic pocket.^4^ However, a phenylalanine residue of Mac1 (Phe132) near the distal ribose could potentially interact with apolar functionalities like benzyl groups. Such beneficial hydrophobic interactions could possibly offset the loss of hydrogen bonding.^29^ To this end, monobenzylated mimics **6** and **7** were prepared (Schemes S3 and S4). However, both compounds showed poor affinity for Mac1 with IC_50_ values far above of those of the parent α-*O*-methyl-ADPr **1** and α-azide-ADPr **3**, indicating that loss of hydrogen bonding, steric bulk, or a combination of the two counteracted any favorable hydrophobic interactions. Additionally, analogue **8** with a fluorine at the 3″ position was prepared by using diethylaminosulfur trifluoride (DAST) to displace a secondary alcohol from d-lyxose derivative **14** to afford **15** (75%, for the α-anomer), which was then further transformed into its corresponding ADPr analogue **8** (Schemes 1 and S5). Interestingly, the introduction of the fluorine also drastically diminished binding affinity, indicating that not necessarily group size alone but also lack of hydrogen bonding was one of the issues at hand. Finally, to probe the binding pocket flexibility toward changes in geometry at the 3″ position, an *O*-methyl oxime was proposed. To this end, the 2″ position of d-ribose analogue **16** was regioselectivity protected with a PMB group (63%), whereafter oxidation of the remaining alcohol using Dess-Martin Periodinane (DMP) to its corresponding ketone (68%) and subsequent transformation into the *O*-methyl oxime gave intermediate **17** with 82% yield (Schemes 1 and S6). This intermediate was then further developed into its corresponding ADPr analogue **9**. However, biophysical activity testing showed that the oxime was detrimental to binding affinity to Mac1, thus resulting in an IC_50_ of >200 μM.

Since modifications on the 2″ and 3″ positions did not yield any desirable results in terms of potency, we did not pursue the modification of these positions any further. Instead, we thought to capitalize on the enhanced potency of the adenosine mimic known as the remdesivir metabolite GS-441524 for binding Mac1.^30^ To this end, compounds **10** and **11** were synthesized, possessing either the α-*O*-methyl riboside or α-azide riboside moiety, respectively (Scheme 2, Table 1). Conveniently the corresponding ribosyl-5-*O*-phosphate (**20** and **21**) building blocks were readily available from the precursors used in the synthesis of ADPr-mimics **1** and **3**.

**Scheme 2:**
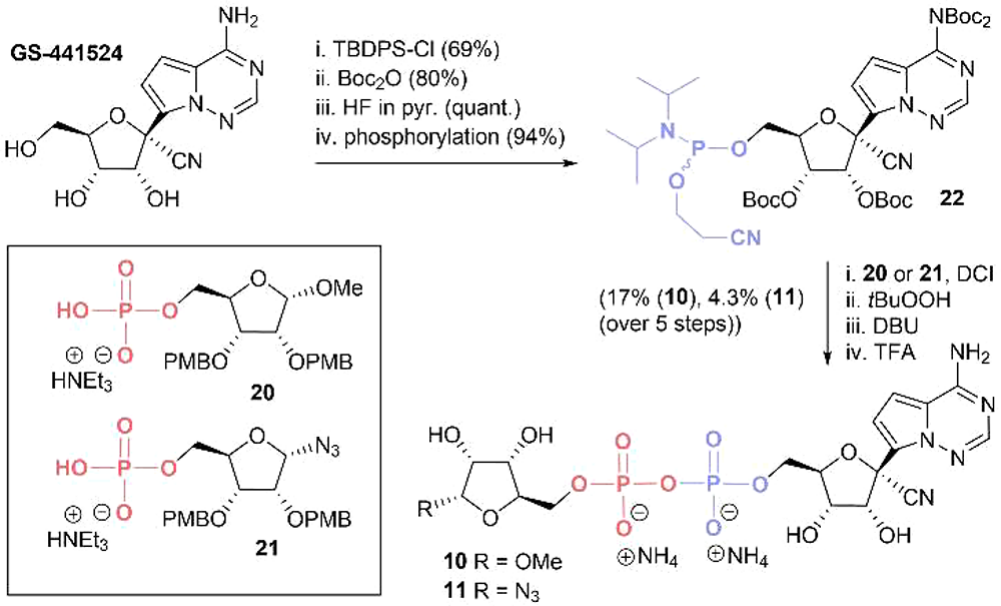
Synthesis of ADPr Analogues **10** and **11** with GS-441524 as an Adenosine Mimic

We therefore focused on preparation of phosphoramidite **22** (Scheme 2), starting from GS-441524, the synthesis of which has been reported as part of efforts in the development of remdesivir.^31^ To this end, we selectively protected the 5′-position with a *tert*-butyldiphenylsilyl group, and the remaining positions were protected with Boc-groups using an excess of di-*tert*-butyl dicarbonate and 4-dimethylaminopyridine (DMAP). This afforded the fully protected intermediate in 80% yield, with the exocyclic amine being double protected. Subsequent removal of the silyl protecting group using HF in pyridine (quantitative) and installing the phosphoramidite gave the required P(III)-building block **22** in 94% yield. Coupling of this phosphoramidite with phosphates **20** and **21** and subsequent acidic global deprotection and HPLC purification delivered the desired pyrophosphates **10** and **11** in 17% and 4.3% yield over five steps.

Interestingly, both analogues demonstrated excellent IC_50_ values for Mac1 inhibition upon biophysical activity testing: 63 nM and 30 nM for α-*O*-methyl riboside **10** and α-ribosylazide **11**, respectively, which is in line with the work of Lin and co-workers,^32^ who also described the development of GS-441524-based ADPr analogues, which was reported as this manuscript was in preparation. The slightly higher inhibitory activity of compound **11** compared to compound **10** mirrors the higher binding affinity of the azide ADPr analogue **3** in comparison to *O*-methyl-ADPr **1** (Figure S2).

To shed light on the binding interactions, we docked inhibitors **10** and **11** into the binding pocket of Mac1 (PDB 7KQP) (Figure 2). Both analogues demonstrated an additional hydrogen bond with Asp157 through the cyano group, which is not possible for ADPr **1** and **3**. The docking study also revealed a new hydrogen bond for the anomeric azide in **11** to Gly47, which is not observed for **10** (Figure S1). The binding energies derived from the molecular docking study (Δ*G* = −11.55, −12.04, and −10.08 kcal/mol for **10**, **11**, and ADPr, respectively) are in good agreement with the inhibitory potency of the two binders with respect to ADPr. Further analysis by determining hydrogen bond occupancy values from the docked structures (Figure S1) indicated that the most potent binder interacts the strongest with Val49, Ile131, and Phe132. Finally, molecular dynamics computations delivered binding enthalpies that were in good agreement with the IC_50_ values and docking energies obtained for ADPr and the two analogues.

**Figure 2:**
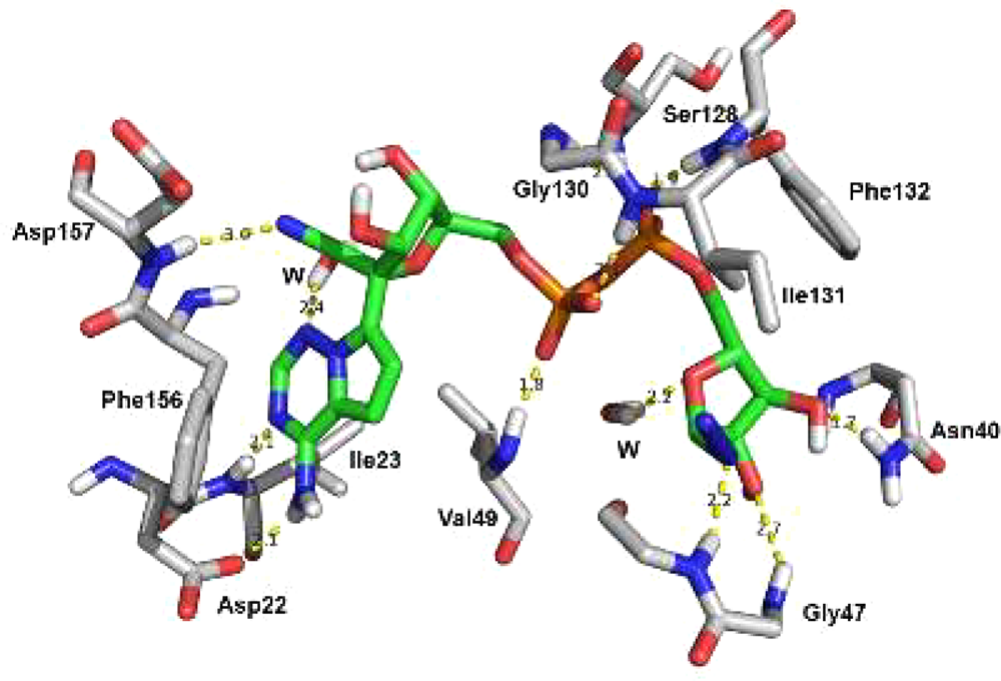
Docking of compound **11** in the Mac1 active site.

In conclusion, we report the synthesis and biophysical evaluation of a focused array of ADPr-mimics to reveal that modification of the 2″ and 3″ alcohols on the distal ribose (as in **6**–**9**) is detrimental for inhibitor potency but that installing an α-azido group on the ribose significantly enhances binding. The modular approach, in which the phosphate of the modified ribose was coupled to a phosphoramidite of GS-441524, resulted in the development of a nanomolar Mac1 inhibitor. Our molecular docking studies identified several new hydrogen bonding interactions established by both the cyano group of the GS-441524 moiety as well as the anomeric azide on the distal ribose, explaining/rationalizing the high binding affinity of the inhibitor to Mac1. Furthermore, the pyrophosphate **11** is a promising lead compound for the development of more potent and more stable analogues as future therapeutic antivirals against SARS-CoV-2 NSP3Mac1 and other macrodomain-containing viruses.

## Data Availability

The data underlying this study are available in the published article and its Supporting Information.

## Supporting Information Available

Experimental details, NMR and LCMS spectra, computational simulations and HTRF assay, molecular binding study, and biological evaluation of the NDPr-style inhibitors (PDF)
