# Supplementary material for: Synthesis of Structural ADP-Ribose Analogues as Inhibitors for SARS-CoV-2 Macrodomain 1
Source: Org Lett. 2024 Jun 27;26(27):5700–4. doi: 10.1021/acs.orglett.4c01792 (PMC11249776; doi:10.1021/acs.orglett.4c01792)

Supporting information
of
Synthesis of Structural ADP-Ribose Analogues as Inhibitors for SARS-CoV-2 Macrodomain 1

Koen J. Rijpkema[1], Marion Schuller[2], Miriam S. van der Veer[1], Sjoerd Rieken[1], Diego L. R. Chang[1], Pascal Balić[1], Alex Todorov[1], Hugo Minnee[1], Sven Wijngaarden[1], Isaac A. Matos[2,3], Nicolas C. Hoch[3], Jeroen D. C. Codée[1], Ivan Ahel[2*], Dmitri V. Filippov[1*]

[1]Leiden Institute of Chemistry, Leiden University, Einsteinweg 55, 2333 CC Leiden, The Netherlands

[2]Sir William Dunn School of Pathology, University of Oxford, South Parks Road, Oxford, OX1 3RE, United Kingdom

[3]Departamento de Bioquímica, Instituto de Química, Universidade de Sao Paulo, Av. Prof. Lineu Prestes, 748 Cidade Universitária, Sao Paulo, 055800-000 Brasil

E-mail: ivan.ahel@path.ox.ac.uk; filippov@chem.leidenuniv.nl

Table of contents

General experimental procedures –-- S3

Scheme S1: Synthesis of 1”-α-*O*-methyl ADPr--- S4

Scheme S2: Synthesis of 1”-α-*C*-ethynyl ADPr --- S6

Scheme S3: Synthesis of 1”-α-*O*-methyl-2”-*O*-benzyl-ADPr---S9

Scheme S4: Synthesis of 1”-α-azido-2”-*O*-benzyl-ADPr --- S13

Scheme S5: Synthesis of Fluorinated ADPr -- S17

Scheme S6: Synthesis of 1”-α-*O*-methyl-D-erythro-pentofuran-3”-uloside *O*-methyl oxime ADPr--------------- S20

Scheme S7: Synthesis of acid-labile P(V) donors--- S24

Scheme S8: Synthesis of GS-441524 P(III) donors and NDPr molecules -- S26

References --- S30

NMR spectra ---S31

Computational simulations & HTRF assay ---S83

Figure S1--- S84

Figure S2 -- S85

LCMS spectra--- S86

General experimental procedures

All chemicals were used as received unless stated otherwise. HF in pyridine and *t*BuOOH (5.5 M in nonane or decanes) were purchased at Sigma Aldrich. Molecular sieves were flamedried (3x) in vacuo before use. Solvents were dried over activated 4Å molsieves for 24 h except for MeCN and MeOH which were dried over 3Å molsieves Reactions were performed under N_2 or argon atmosphere unless stated otherwise. A Julabo FT902 cryostat was used for low temperature glycosylation reactions. Reaction mixtures were concentrated under reduced pressure using rotary evaporators at 40-45 ˚C unless state otherwise. Reactions were monitored by thin layer chromatography (TLC) analysis using silica gel 60 F254 coated aluminium sheets from Merck. TLC plates were visualized with ultraviolet light (254 nm) or sprayed with H_2SO_4 (20% v/v in MeOH), potassium permanganate (1 gram $KMnO_4$, 5 grams K_2CO_3, in 200 ml H_2O) or ceric ammonium molybdate (1 gram $Ce(NH_4)_4(SO_4)_4$•$2H_2O$, 2.5 grams $(NH_4)_6Mo_7O_{24}$•$4H_2O$, 10 mL H_2SO_4 in 90 mL H_2O). Infrared (IR) values are reported in cm^{-1}. Analytical LC-MS was performed on a LCQ Adventage Max (Thermo Finnigan) ion-trap spectrometer (ESI+) coupled to a surveyor HPLC system (Thermo Finnigan) equipped with a C18 column (Gemini, 4.6 mm x 50 mm, 5 µm particle size, phenomex) in combination buffers A: H_2O, B:acetonitrile and C: 1% aq. TFA. Alternatively, LC-MS analysis was performed on a JASCO HPLC system (detection simultaneously at 214 and 254 nm) coupled to a PE/SCIEX API 165 single quadrupole mass spectrometer (Perkin-Elmer) equipped with a C18 column (Gemini, 4.6 x 50 mm, 3 µm particle size, Phenomenex) in combination with buffers A: H_2O, B:acetonotrile and C: 0.1 M aq. NH4OAc. High resolution mass spectra were recorded by direct injection on a mass spectrometer (Thermo Finnigan LTQ Orbitrap) equipped with an electrospray ion source in positive mode (source voltage 3.5 kV, sheath gas flow 10, capillary temperature 250°C) with resolution R = 60000 at m/z 400 (mass range m/z 150 – 4000) and dioctylpthalate (m/z = 391.28428) as a 'lock mass". For compounds **10** and **11** HRMS data were recorded on Sciex X500B QTOF mass spectrometer calibrated as recommended by the manufacturer. ^{1}H NMR, ^{13}C NMR, ^{19}F NMR and ^{31}P NMR spectra were recorded on Bruker AV-300 (300 MHz), AV-400 (400 MHz) or AV-500 (500 MHz) spectrometer. ^{13}C NMR spectra are acquired via the attached proton test (APT) experiment and are presented with even signals S-3 (Cq and CH_2) pointing upwards and odd signals (CH and CH_3) pointing downwards. The chemical shifts are noted as δ-values in parts per million (ppm) relative to the tetramethylsilane signal (δ = 0 ppm) or solvent signal of D_2O (δ = 4.79 ppm) for ^{1}H NMR and relative to the solvent signal of $CDCl_3$ (δ = 77.16 ppm) for ^{13}C NMR. Phosphorylation reactions were monitored with ^{31}P NMR using an acetone-d6 insert for a locking signal and the resulting spectra were indirectly calibrated with H_3PO_4. HRMS samples were prepared in either MeOH, acetonitrile or MilliQ grade water with an approximate concentration of 1 mM and measured on a Thermo Scientific LTQ Orbitrap XL. Size exclusion chromatography (SEC), here sometimes referred to as "gel filtration", was performed by constant elution (1 ml/min) with an aqueous NH_4OAc (0.15 M) + 10% acetonitrile buffer system over an HW-40-S resin (16x 600 mm) from TOYOPEARL. Purification by preparative high pressure liquid chromatography (HPLC) if carried out is performed on a Gilson-preparative-system equipped with a Phenomenex-Gemini-NX C18 column (5µm, 10x250 mm) using Buffer A (25 mM NH_4OAc in water) and Buffer B (MeCN) (0 → 20% A/B). Yields for the ADPr analogues after size exclusion chromatography were calculated assuming its obtained as NH_4 salt.

Scheme S1: Synthesis of 1”-α-*O*-methyl ADPr

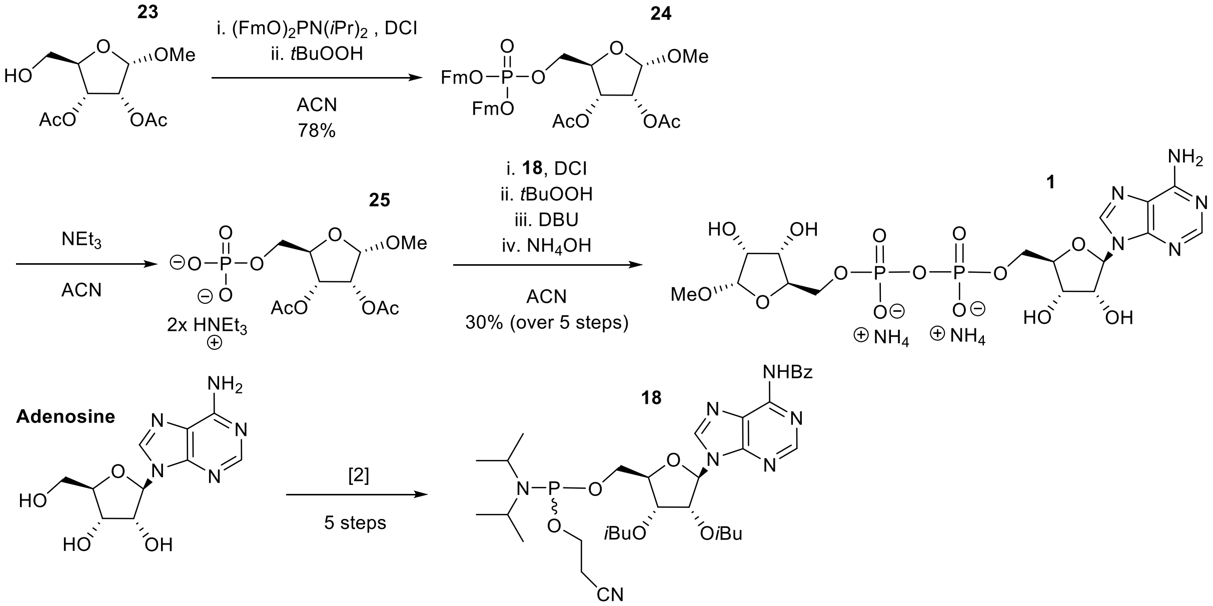

1-α-*O*-methyl-2,3-di-*O*-acetyl-D-ribofuranoside (23) Compound **23** was prepared following a procedure previously described.[1] All spectra are in full accordance with literary precedence. ^{1}H NMR (500 MHz, $CDCl_3$) δ 5.21 (dd, *J* = 7.5, 3.6 Hz, 1H), 5.15 (d, *J* = 4.4 Hz, 1H), 4.97 (dd, *J* = 7.5, 4.5 Hz, 1H), 4.15 (q, *J* = 3.5 Hz, 1H), 3.90 – 3.77 (m, 2H), 3.45 (s, 3H), 2.21 (dd, *J* = 7.8, 4.8 Hz, 1H), 2.14 (s, 3H), 2.14 (s, 3H). ^{13}C NMR (126 MHz, $CDCl_3$) δ 170.9, 170.1, 101.7, 82.5, 71.3, 70.1, 62.4, 55.6, 21.0, 20.7. HRMS (ESI) m/z: $[M+Na]^+$ Calcd for $C_{10}H_{16}O_7Na$ 271.0788; Found 271.0790.

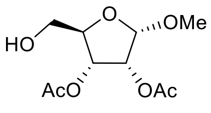

1-α-*O*-methyl-2,3-di-*O*-acetyl-5-*O*-(di(9*H*-fluoren-9-yl))-phosphoryl-D-ribofuranoside (24) To compound **23** (124 mg, 0.500 mmol) was added 4,5-di-cyanoimidazole (DCI) (177 mg, 1.50 mmol, 3.00 eq.) and the mixture was co-evaporated thrice with dried and distilled toluene. The resulting mixture was then dissolved in dry acetonitrile (3.3 mL, 0.15 M) and brought under a nitrogen atmosphere. While stirring, to the now clear solution was added $(FmO)_2PN(iPr)_2$ as a solution (0.32 M in dry acetonitrile) (2.3 mL, 0.75 mmol, 1.5 eq.) and the resulting solution was allowed to stir at room temperature for 80 minutes. Next, a solution of *tert*-butyl hydroperoxide (*t*BuOOH) (5.5 M in decanes) (0.45 mL, 2.5 mmol, 5.0 eq.) was added and the reaction mixture was stirred until thin layer chromatography (TLC) analysis indicated full conversion of the phosphite intermediate to the protected phosphate. Upon completion, the reaction was quenched by the addition of water and the product was extracted using dichloromethane. The aqueous phases were back-extracted multiple times using dichloromethane. The combined organic phases were dried over $MgSO_4$, filtered off and concentrated *in vacuo*. Purification by silica column chromatography (ethyl acetate/pentane, 2/3 → 3/1, v/v) gave compound **24** (268 mg, 0.393 mmol, 78%). TLC: R_f 0.5 (ethyl acetate/pentane, 4/1, v/v). ^{1}H NMR (400 MHz, $CDCl_3$) δ 7.77 – 7.67 (m, 4H), 7.60 – 7.47 (m, 4H), 7.43 – 7.31 (m, 4H), 7.31 – 7.24 (m, 4H), 5.12 (dd, *J* = 7.4, 3.5 Hz, 1H), 4.97 (d, *J* = 4.5 Hz, 1H), 4.83 (dd, *J* = 7.4, 4.5 Hz, 1H), 4.36 – 4.23 (m, 4H), 4.19 – 4.07 (m, 5H), 3.36 (s, 3H), 2.13 (s, 3H), 2.10 (s, 3H). ^{13}C NMR (101 MHz, $CDCl_3$) δ 170.6, 169.9, 143.2, 143.1, 143.0, 141.5, 141.5, 128.1, 128.0, 127.3, 125.27, 125.25, 125.23, 120.16, 120.1, 101.7, 80.4 (d, *J* = 7.4 Hz), 70.9, 69.6 (d, *J* = 5.2 Hz), 69.5, 66.9 (d, *J* = 5.5 Hz), 55.7, 48.0 (d, *J* = 2.7 Hz), 47.9 (d, *J* = 2.6 Hz), 20.9, 20.6. ^{31}P NMR (122 MHz, aceton-capil) δ -1.2. HRMS (ESI) m/z: $[M+Na]^+$ Calcd for $C_{38}H_{37}O_{10}PNa$ 707.2017; Found 707.2024.

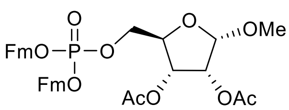

1-α-*O*-methyl-2,3-di-*O*-acetyl-5-*O*-phosphate-D-ribofuranoside (25) Compound **24** (132 mg, 0.193 mmol) was dissolved in dry acetonitrile (4.5 mL, 0.044 M) and was brought under an atmosphere of nitrogen whereafter upon stirring was added triethylamine (0.45 mL, 3.3 mmol, 17 eq.). The resulting solution was allowed to stir for 40 hours at room temperature at which point full conversion of the starting material and its mono-protected intermediate into the corresponding phosphate was observed. The reaction mixture was directly concentrated *in vacuo* after which it was used crude in the next reaction without further purification.

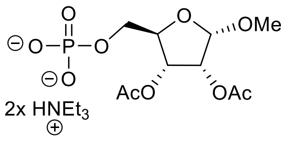

2-cyanoethoxy-N,N'-diisopropylamino-(6-N-benzoyl-2',3'-di-O-isobutyryl-β-D-adenyl-5'-yl)phosphine (18)

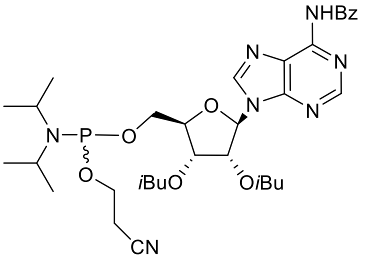

Compound **18** was prepared following a procedure previously described.[2] All spectra are in full accordance with literary precedence. ^{1}H NMR (400 MHz, $CDCl_3$) δ 9.42 (bs, 1H), 8.78 (d, *J* = 4.8 Hz, 1H), 8.54 (d, *J* = 9.5 Hz, 1H), 8.06 – 7.99 (m, 2H), 7.63 – 7.54 (m, 1H), 7.54 – 7.45 (m, 2H), 6.44 (dd, *J* = 18.7, 6.9 Hz, 1H), 5.83 (ddd, *J* = 13.6, 6.9, 5.4 Hz, 1H), 5.64 (ddd, *J* = 10.6, 5.4, 2.3 Hz, 1H), 4.42 (p, *J* = 2.5 Hz, 1H), 4.14 – 3.79 (m, 4H), 3.73 – 3.56 (m, 1H), 2.80 – 2.59 (m, 3H), 2.59 – 2.44 (m, 1H), 1.31 – 1.14 (m, 21H), 1.14 – 1.04 (m, 7H). ^{13}C NMR (101 MHz, $CDCl_3$) δ 175.84, 175.79, 175.3, 175.2, 164.8, 152.8, 152.7, 152.1, 151.9, 149.6, 133.64, 133.63, 132.7, 128.9, 128.76, 128.74, 128.1, 127.8, 123.2, 123.1, 117.7, 117.6, 85.4, 84.9, 83.8, 83.7, 83.5, 83.4, 77.4, 73.9, 73.9, 71.9, 71.6, 63.2, 63.0, 62.9, 62.8, 58.8, 58.6, 58.6, 58.4, 43.1, 43.0, 33.8, 33.8, 33.6, 33.5, 24.70, 24.67, 24.62, 24.60, 20.4, 20.34, 20.28, 20.25, 18.90, 18.88, 18.8, 18.7, 18.6. ^{31}P NMR (162 MHz, $CDCl_3$) δ 149.5, 149.0.

1”-*α*-*O*-methyl-ADPr (1) Crude compound **25** (~0.19 mmol) was co-evaporated thrice with a mixture of

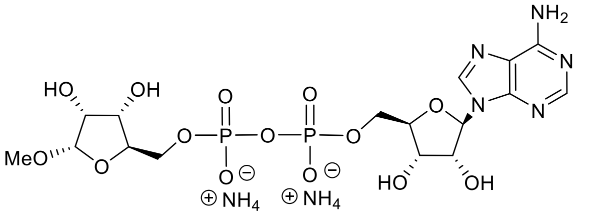

pyridine/acetonitrile (1/1, v/v) after which it was co-evaporated one more time with dry acetonitrile. Then, to the mixture was added DCI (59 mg, 0.50 mmol, 2.5 eq.) after which the resulting mix was co-evaporated three more times with dry acetonitrile. The mixture was then suspended in dry acetonitrile (3.6 mL, 0.055 M) and brought under a nitrogen atmosphere. While stirring, a solution of phosphoramidite **18** (0.92 mL, 0.20 mmol, 1.0 eq.) in dry acetonitrile (0.91 mL, 0.22 M) was added dropwise to the suspension, after which the resulting off-white suspension was allowed to stir at room temperature for 100 minutes. Then, *t*BuOOH (5.5 M in decanes) (75 µl, 0.41 mmol, 2.1 eq.) was added and the same amount of *t*BuOOH was added again after one hour of stirring, after which ^{31}P-NMR indicated full conversion of the P(III) into its P(V) counterpart. Next, 1,8-diazabicyclo(5.4.0)undec-7-ene (DBU) (0.15 mL, 1.0 mmol, 5.0 eq.) was added to the reaction mixture and the resulting solution was stirred at room temperature for 100 minutes after which aqueous ammonium hydroxide solution (28% w/w, 3.6 mL) was added and the reaction vessel was sealed with a glass stopper. The resulting clear yellow solution was stirred at room temperature for 23 hours when LCMS analysis indicated full deprotection of the construct. The reaction mixture was diluted with water and diethyl ether after which the aqueous phase was washed multiple times with diethyl ether. The aqueous phase was then concentrated *in vacuo* and the crude product was further purified using gel filtration (HW-40, NH_4OAc buffer) after which the collected fractions were lyophilized to afford compound **1** (34.8 mg, 57.4 µmol, 30% (over 5 steps)) as the ammonium salt as a white foam. ^{1}H NMR (400 MHz, D_2O) δ 8.40 (s, 1H), 8.09 (s, 1H), 6.03 (d, *J* = 5.8 Hz, 1H), 4.83 – 4.77 (m, 1H), 4.69 (t, *J* = 5.5 Hz, 1H), 4.46 (dd, *J* = 5.2, 3.7 Hz, 1H), 4.33 (dq, *J* = 5.2, 2.9 Hz, 1H), 4.19 – 4.13 (m, 2H), 4.13 – 4.07 (m, 1H), 4.06 – 4.02 (m, 2H), 3.93 (dd, *J* = 5.3, 3.9 Hz, 2H), 3.30 (s, 3H). ^{13}C NMR (101 MHz, D_2O) δ 155.3, 152.6, 148.9, 139.7, 118.4, 103.2, 86.9, 83.8 (d, *J* = 8.3 Hz), 83.1 (d, *J* = 8.1 Hz), 74.2, 70.8, 70.3, 69.6, 65.6 (d, *J* = 5.4 Hz), 65.2 (d, *J* = 4.5 Hz), 55.4. ^{31}P NMR (162 MHz, D_2O) δ -10.9 – -11.6 (m). HRMS (ESI) m/z: $[M+H]^+$ Calcd for $C_{16}H_{26}N_5O_{14}P_2$ 574.0946; Found 574.0947.

Scheme S2: Synthesis of 1”-α-*C*-ethynyl ADPr

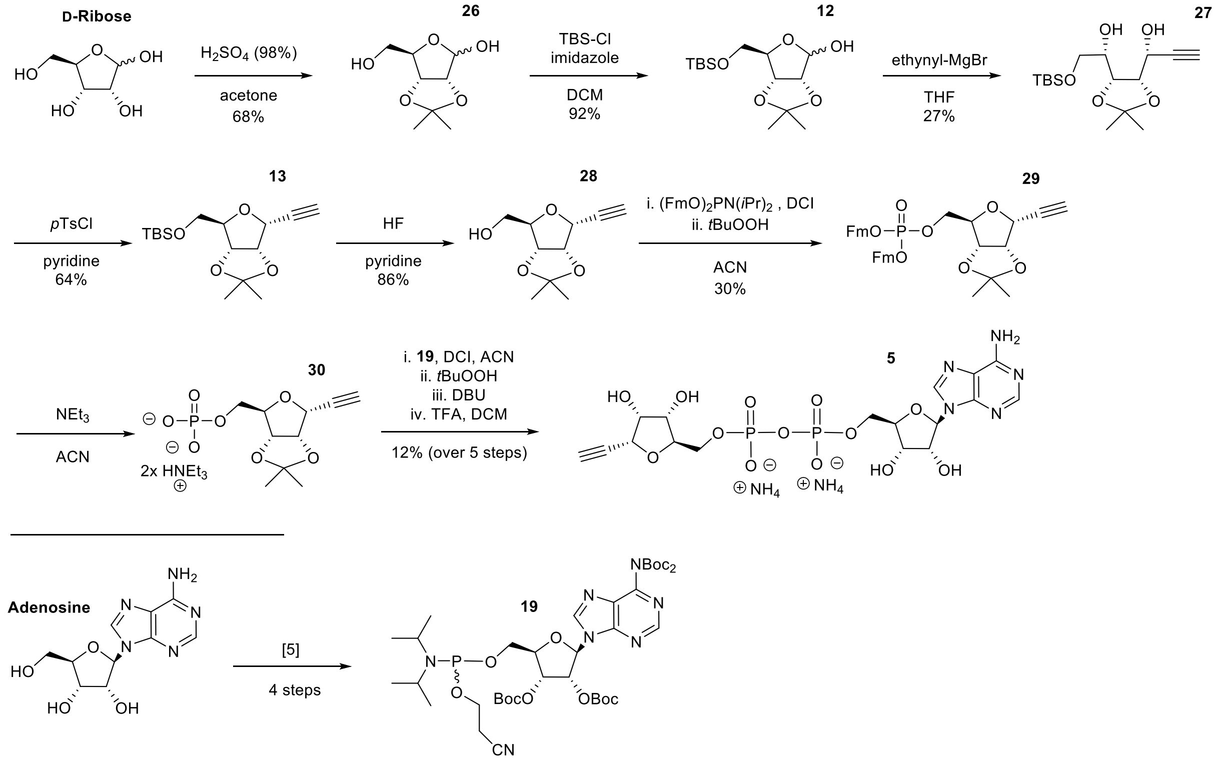

2,3-*O*-*iso*-propylidene-D-ribofuranoside (26) D-Ribose (5.0 grams, 33 mmol) was dissolved in anhydrous

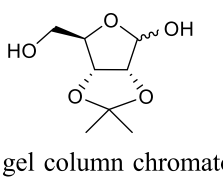

acetone (50 mL, 0.66 M) at 0 °C and stirred. A catalytic amount of sulfuric acid (98%, w/w) was added and the reaction mixture turned clear after 25 minutes. After an additional hour, TLC analysis indicated full consumption of the starting material and triethylamine was added until the solution reached a pH value of approximately 8. The reaction mixture was filtered over a celite pad and concentrated *in vacuo* to yield a straw coloured oil. Purification by silica gel column chromatography (dichloromethane/methanol, 49/1 → 9/1, v/v) afforded title compound **26** (4.30 g, 22.6 mmol, 68%) as both anomers as a clear oil. Only major is reported. Rf 0.4 (dichloromethane/methanol, 23/2, v/v). ^{1}H NMR (400 MHz, $CDCl_3$) δ 5.43 (q, *J* = 5.8, 5.4, 4.2 Hz, 1H), 4.85 (dt, *J* = 5.8, 1.3 Hz, 1H), 4.60 (d, *J* = 5.9 Hz, 1H), 4.54 (bs, 1H), 4.42 (t, *J* = 2.8 Hz, 1H), 3.82 – 3.67 (m, 2H), 3.43 (s, 1H), 1.50 (s, 3H), 1.33 (s, 3H). ^{13}C NMR (101 MHz, $CDCl_3$) δ 103.2, 87.9, 87.0, 81.8, 63.9, 26.5, 24.8. HRMS (ESI) m/z: $[M+Na]^+$ Calcd for $C_8H_{14}O_5Na$ 213.0739; Found 213.0722. Note: compound would not readily ionize. However, NMR is in accordance with literature.[3]

2,3-*O*-*iso*-propylidene-5-O-(*tert*-butyl-dimethylsilyl)-D-ribofuranoside (12) Compound **26** (4.30 grams, 22.6

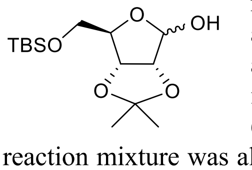

mmol) was co-evaporated thrice with anhydrous toluene before it was dissolved in anhydrous dichloromethane (75 mL, 0.30 M). The solution was brought under a nitrogen atmosphere, cooled to 0 °C using an icebath and stirred. To the resulting solution was added imidazole (1.85 grams, 27.2 mmol, 1.20 eq.) and *tert*-butyldimethylsilyl chloride (TBS-Cl) (4.09 grams, 27.2 mmol, 1.20 eq.) after which the icebath was removed and the resulting reaction mixture was allowed to stir for 17 hours at room temperature. At this point TLC analysis indicated full consumption of the starting material and the reaction was quenched by addition of a 1/3 mixture of water/brine. The resulting mixture was diluted with dichloromethane after which the phases were separated and the aqueous phase was washed once more with dichloromethane. The combined organic phases were then dried over $MgSO_4$, filtered off and concentrated *in vacuo* to afford a yellow oil. Purification by silica gel column chromatography (pentane/ethyl acetate, 19/1 → 9/1 → 0/1, v/v) afforded title compound **12** (6.35 grams, 20.9 mmol, 92 %, anomeric mixture, only major reported) as a clear oil, which solidified into a white plaque upon standing. R_f 0.6 (pentane/ethyl acetate, 9/1, v/v). ^{1}H NMR (400 MHz, $CDCl_3$) δ 5.33 (s, 1H), 4.83 (dt, *J* = 5.9, 0.6 Hz, 1H), 4.48 (d, *J* = 5.9 Hz, 1H), 4.34 (t, *J* = 2.7 Hz, 1H), 3.73 – 3.61 (m, 2H), 3.62 – 3.51 (m, 1H), 3.44 (dd, *J* = 10.8, 2.3 Hz, 1H), 1.43 (s, 3H), 1.26 (s, 3H), 0.85 (s, 9H), 0.12 (s, 3H), 0.11 (s, 3H). ^{13}C NMR (101 MHz, $CDCl_3$) δ 112.1, 103.8, 88.9, 88.2, 81.9, 64.0, 26.5, 25.7, 24.8, 18.0, -4.5, -5.2. HRMS (ESI) m/z: $[M+Na]^+$ Calcd for $C_{14}H_{28}O_5SiNa$ 327.1598; Found 327.1598.

(2R,3R,4S,5S)-1-*O*-(*tert*-butyl-dimethylsilyl)-hept-6-yne-2,5-diol-3,4-*O*-*iso*-propylidene (27) Compound **12** (3.00 grams, 9.85 mmol) was co-evaporated thrice with anhydrous toluene and dissolved in anhydrous tetrahydrofuran (33 mL, 0.30 M). The solution was cooled to 0 °C, purged with argon gas and vigorously stirred. Then, to the stirring solution was added ethylene magnesium bromide (0.5 M in anhydrous tetrahydrofuran) (49 mL, 25 mmol, 2.5 eq.) dropwise, resulting in a brown solution which was allowed to stir for 48 hours at room temperature. At this point reaction progress had halted and more of the Grignard reagent was added (20 mL, 10 mmol, 1.0 eq.) was added. The resulting mixture was then allowed to stir for an additional two hours before the reaction was quenched by addition of sat. aq, ammonium chloride solution. The organic phase was then washed thrice with sat. aq. ammonium chloride solution, once with water and once with brine. The organic phase was then dried over Na_2SO_4, filtered off and concentrated *in vacuo* which afforded a yellow oil. Subsequent purification by silica gel column chromatography (pentane/ethyl acetate, 97.5/2.5 → 87.5/12.5 → 4/1, v/v) afforded title compound **27** (0.89 grams, 2.7 mmol, 27 %, d.r = 95/5) as a clear oil. R_f 0.5 (pentane/ethyl acetate, 24/1, v/v). ^{1}H NMR (400 MHz, $CDCl_3$) δ 4.66 (ddd, *J* = 8.3, 4.6, 2.1 Hz, 1H), 4.30 (dd, *J* = 8.4, 5.5 Hz, 1H), 4.21 (d, *J* = 4.6 Hz, 1H), 4.13 – 4.07 (m, 1H), 3.89 – 3.81 (m, 2H), 3.69 – 3.62 (m, 1H), 3.25 (d, *J* = 4.0 Hz, 1H), 2.52 (d, *J* = 2.2 Hz, 1H), 1.43 (s, 3H), 1.36 (s, 3H), 0.91 (s, 9H), 0.09 (s, 6H). ^{13}C NMR (101 MHz, $CDCl_3$) δ 109.5, 82.6, 80.1, 77.36, 76.6, 73.7, 69.3, 64.2, 61.4, 27.9, 26.0, 25.5, 18.5, -5.2, -5.3. HRMS (ESI) m/z: $[M+Na]^+$ Calcd for $C_{16}H_{30}O_5SiNa$ 353.1755; Found 353.1753.

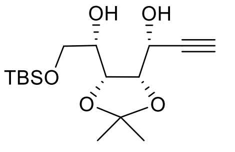

1-α-*C*-ethynyl-2,3-*O*-*iso*-propylidene-5-*O*-(*tert*-butyl-dimethylsilyl)-D-ribofuranoside (13) Compound **27** (0.89 grams, 2.7 mmol) was co-evaporated thrice with anhydrous toluene before it was dissolved in anhydrous pyridine (56 mL, 0.048 M), brought under a nitrogen atmosphere and stirred vigorously. Then, *para*-toluenesulfonyl chloride (*p*TsCl) (2.12 grams, 11.1 mmol, 4.11 eq.) was added and the resulting reaction mixture was allowed to stir for 48 hours at which point TLC analysis indicated complete consumption of the starting material. The reaction mixture was quenched by careful addition of water and concentrated *in vacuo*. Purification by silica gel column chromatography (pentane/ethyl acetate 24/1 → 22/3, v/v) yielded the title compound **13** (543 mg, 1.74 mmol, 64%) as a straw coloured oil. R_f 0.4 (pentane/ethyl acetate 1/4, v/v). ^{1}H NMR (400 MHz, $CDCl_3$) δ 4.89 (ddd, *J* = 4.5, 2.2, 0.6 Hz, 1H), 4.83 (dd, *J* = 6.1, 1.0 Hz, 1H), 4.75 (dd, *J* = 6.0, 4.5 Hz, 1H), 4.16 (td, *J* = 2.8, 0.8 Hz, 1H), 3.79 (dd, *J* = 11.0, 2.8 Hz, 1H), 3.69 (dd, *J* = 11.0, 2.6 Hz, 1H), 2.61 (d, *J* = 2.3 Hz, 1H), 1.57 (s, 3H), 1.38 (s, 3H), 0.87 (s, 9H), 0.05 (s, 6H). ^{13}C NMR (101 MHz, $CDCl_3$) δ 113.2, 84.6, 83.2, 82.5, 78.6, 76.0, 73.9, 65.6, 26.6, 25.9, 25.4, 18.2, -5.4, -5.6. HSQC-HECADE NMR (400 MHz, $CDCl_3$): $^2J_{1''H\text{-}2''C}$ = 0.2 Hz (α), $^2J_{2''H\text{-}1''C}$ = 6.7 Hz (α) HRMS (ESI) m/z: $[M+Na]^+$ Calcd for $C_{16}H_{28}O_4SiNa$ 335.1649; Found 335.1651.

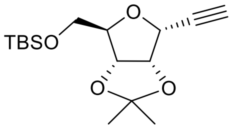

1-α-*C*-ethynyl-2,3-*O*-*iso*-propylidene-D-ribofuranoside (28) Compound **13** (31 mg, 0.10 mmol) was dissolved in pyridine (1.0 mL, 0.1 M) and transferred to a falcon tube wherein it was brought under a nitrogen atmosphere, stirred and cooled to 0 °C. To the stirring solution was added HF-pyridine (70% w/w) (0.1 mL) after which the reaction mixture was stirred for three and a half hours while being allowed to warm to room temperature. When TLC analysis indicated complete conversion of the starting material the reaction was quenched by the careful addition of aq. sat. sodium bicarbonate. The resulting mixture was subsequently diluted with dichloromethane and the organic phase was dried over Na_2SO_4, filtered off and concentrated *in vacuo*. Purification by silica gel column chromatography (pentane/ethyl acetate, 24/1 → 22/3, v/v) yielded the title compound **28** (18 mg, 0.091 mmol, 86%) as a yellow oil. R_f 0.5 (dichloromethane/methanol, 19/1, v/v). ^{1}H NMR (400 MHz, $CDCl_3$) δ 4.85 – 4.78 (m, 2H), 4.75 (dd, *J* = 5.9, 1.8 Hz, 1H), 4.23 (ddd, *J* = 5.5, 3.6, 1.8 Hz, 1H), 3.80 – 3.63 (m, 2H), 2.66 (d, *J* = 2.1 Hz, 1H), 2.04 (bs, 1H), 1.60 (s, 3H), 1.39 (s, 3H). ^{13}C NMR (101 MHz, $CDCl_3$) δ 114.1, 84.3, 82.3, 81.9, 78.1, 76.7, 72.5, 62.8, 26.4, 25.4. HRMS (ESI) m/z: $[M+Na]^+$ Calcd for $C_{10}H_{14}O_4Na$ 221.0784; Found 221.0779. Note: compound would not readily ionize. NMR is in accordance with literature.[4]

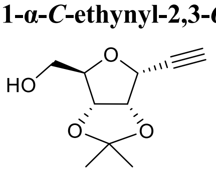

1-α-*C*-ethynyl-2,3-*O*-*iso*-propylidene-5-*O*-(di(9*H*-fluoren-9-yl))-phosphoryl-D-ribofuranoside (29) A mixture of compound **28** (15 mg, 74 µmol) and DCI (26.8 mg, 0.23 mmol, 3.0 eq.) was co-evaporated thrice with anhydrous toluene, dissolved in anhydrous acetonitrile and purged with argon. In another flask (argon purged and flame dried), $(FmO)_2PN(i\text{-}Pr)_2$ was dissolved in anhydrous acetonitrile (0.4 M). Some of the $(FmO)_2PN(i\text{-}Pr)_2$ stock solution (0.35 mL, 0.14 mmol, 1.5 eq.) was added slowly to the reaction mixture containing compound **28** and DCI. The resulting solution was allowed to stir for two hours at room temperature after which a second batch of the $(FmO)_2PN(i\text{-}Pr)_2$ stock solution (0.35 mL, 1.5 eq) was added and the reaction mixture was allowed to stir for an additional hour. At this point TLC analysis indicated complete consumption of the starting material and *t*BuOOH (5.5 M in decanes) (67 µL, 0.37 mmol, 5.0 eq.) was added. The resulting mixture was allowed to stir for 30 minutes when ^{31}P-NMR indicated full conversion of all phosphite

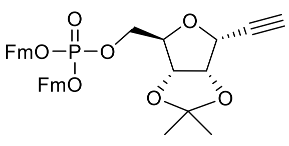

species into their corresponding phosphates. The reaction mixture was quenched with water and diluted with dichloromethane after which the phases were separated and the aqueous phase was washed thrice more with dichloromethane. The combined organic phases were then dried over Na_2SO_4, filtered off and concentrated *in vacuo*. Purification by silica gel column chromatography (pentane/ethyl acetate, 19/1 → 9/1, v/v) afforded title compound **29** (16 mg, 25 µmol, 30 %) as a yellow oil. R_f 0.5 (pentane/ethyl acetate, 1/1, v/v). TLC-MS: [M+Na] = 657. ^{1}H NMR (400 MHz, $CDCl_3$) δ 7.77 – 7.66 (m, 5H), 7.60 – 7.27 (m, 13H), 4.88 – 4.69 (m, 1H), 4.57 – 4.44 (m, 2H), 4.38 – 4.20 (m, 4H), 4.12 (dd, *J* = 6.5, 2.3 Hz, 3H), 3.82 – 3.60 (m, 3H), 2.62 (d, *J* = 1.9 Hz, 1H), 1.54 (s, 3H), 1.32 (s, 3H). ^{13}C NMR (101 MHz, $CDCl_3$) δ 142.8, 141.5, 139.7, 128.18, 128.16, 128.1, 127.34, 127.31, 125.0, 120.3, 120.2, 114.1, (82.3, 82.2, 82.1, 81.7) (C-P coupling not resolved), 77.4, 76.8, 72.8, 69.5 (d, *J* = 6.1 Hz), 67.4 (appears as t, *J* = 6.0 Hz), 62.8, 47.9 (d, *J* = 2.6 Hz), 47.8 (d, *J* = 2.2 Hz), 26.4, 25.4. ^{31}P NMR (162 MHz, $CDCl_3$) δ -2.7. HRMS (ESI) m/z: $[M+Na]^+$ Calcd for $C_{38}H_{35}O_7PNa$ 657.2013; Found 657.2018.

2-cyanoethoxy-*N'*,*N'*-di-*iso*-propylamino-(6-*N*,*N*-di-(*tert*-butyloxycarbonyl)-2',3'-*O*-di-(*tert*-butyloxycarbonyl-β-D-adenyl-5'-yl)phosphine (19) Compound **19** was prepared following a procedure previously described.[5] All spectra are in full accordance with literary precedence. ^{1}H NMR (400 MHz, $CDCl_3$) δ 8.85 (dd, *J* = 4.6, 1.4 Hz, 1H), 8.57 (d, *J* = 10.8 Hz, 1H), 6.43 (dd, *J* = 18.7, 6.6 Hz, 1H), 5.83 – 5.73 (m, 1H), 5.60 – 5.35 (m, 1H), 4.54 – 4.46 (m, 2H), 4.07 (ddd, *J* = 10.9, 6.1, 3.9 Hz, 1H), 4.05 – 3.75 (m, 5H), 3.72 – 3.56 (m, 2H), 2.72 (dt, *J* = 20.5, 6.5 Hz, 2H), 1.53 (d, *J* = 4.5 Hz, 10H), 1.48 – 1.42 (m, 24H), 1.40 (s, 9H), 1.24 – 1.14 (m, 14H). ^{13}C NMR (101 MHz, $CDCl_3$) δ 153.4, 153.2, 152.4, 152.3, 152.25, 152.24, 151.8, 151.7, 150.4, 150.35, 150.32, 143.1, 129.1, 129.0, 117.55, 117.53, 85.4, 84.8, 83.8, 83.5, 83.38, 83.36, 82.7, 82.6, 82.3, 82.2, 76.0, 75.9, 74.1, 73.7, 63.0, 62.9, 62.83, 62.76, 58.9, 58.7, 58.7, 58.5, 43.3, 43.3, 43.2, 43.1, 27.78, 27.78, 27.69, 27.67, 27.5, 24.8, 24.73, 24.69, 24.65, 24.6, 20.30, 20.29, 20.22, 20.21. ^{31}P NMR (162 MHz, $CDCl_3$) δ 149.7, 149.4.

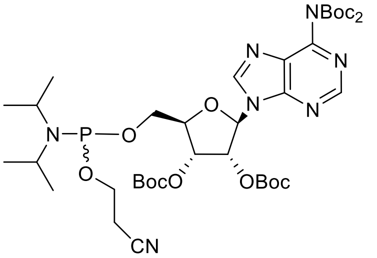

1”-α-*C*-ethynyl ADPr (5) Compound **29** (16 mg, 25 µmol) was co-evaporated thrice with anhydrous toluene before it was dissolved in anhydrous acetonitrile (1.0 mL, 0.025 M). To the solution was then added triethylamine (0.17 mL, 1.2 mmol, 50 eq.) and the reaction mixture was allowed to stir at room temperature for four days under an argon atmosphere. At this point TLC analysis indicated complete consumption of the starting material as well as its mono-protected intermediate. The reaction mixture was directly concentrated *in vacuo* and DCI was added (12 mg, 0.10 mmol, 4.0 eq.). The resulting mixture was subsequently co-evaporated thrice with anhydrous acetonitrile after which the materials were suspended in dry acetonitrile (0.42 mL, 0.060 M) and brought under an argon atmosphere. In another flask, compound **19** (33 mg, 40 µmol, 1.6 eq.) was co-evaporated thrice with anhydrous toluene, dissolved in anhydrous acetonitrile (0.2 mL, 0.2 M) after which the resulting solution was slowly added to the preceding suspension containing the phosphate **30**. The solution was then allowed to stir for six hours at room temperature before *t*BuOOH (5.5 M in decanes) (45 µL, 10 eq.) was added and the resulting solution was allowed to stir for 18 hours at 4 °C after which ^{31}P-NMR indicated full consumption of all phosphite species into their corresponding phosphates. The reaction mixture was then allowed to warm to room temperature and to it was added DBU (32 µL, 0.23 mmol, 9.2 eq.) after which the mixture was allowed to stir for 40 minutes. The solution was then concentrated *in vacuo* after which the concentrate was dissolved in a solvent system consisting of water and trifluoroacetic acid (TFA) (1/1, v/v) (0.42 mL, 0.060 M). The resulting reaction mixture was allowed to stir for 48 hours at which point LCMS analysis indicated full deprotection of the construct and the reaction mixture was subsequently directly concentrated *in vacuo*. The resulting concentrate was dissolved in water (6.0 mL), washed thrice with ethyl acetate and the aqueous phase was concentrated *in vacuo*. Purification by size exclusion chromatography (HW-40, NH_4OAc buffer) and preparatory HPLC afforded compound **5** (1.7 mg, 3.0 µmol, 12% (over 5 steps)) as the ammonium salt as a white solid. ^{1}H NMR (850 MHz, D_2O) δ 8.45 (s, 1H), 8.19 (s, 1H), 6.07 (d, *J* = 5.7 Hz, 1H), 4.69 – 4.68 (m, 1H), 4.59 (dd, *J* = 3.9, 2.2 Hz, 1H), 4.46 (dd, *J* = 5.1, 3.7 Hz, 1H), 4.32 (d, *J* = 3.6 Hz, 1H), 4.20 (dd, *J* = 7.4, 4.7 Hz, 1H), 4.15 (s, 1H), 4.09 (t, *J* = 4.3 Hz, 1H), 4.03 (q, *J* = 7.1 Hz, 2H), 3.97 (dd, *J* = 7.7, 3.8 Hz, 1H), 3.95 – 3.89 (m, 1H), 2.84 (d, *J* = 2.1 Hz, 1H). ^{13}C NMR (214 MHz, D_2O) δ 155.7, 152.9, 129.4, 104.1, 86.9, 83.8 (d, *J* = 7.5 Hz), 80.5 (d, *J* = 5.5 Hz), 78.1, 77.7, 74.3, 72.2, 71.4, 71.1, 70.3, 68.5, 65.3 (d, *J* = 4.1 Hz), 65.2 (d, *J* = 3.9 Hz). ^{31}P NMR (1H-decoupled, 162 MHz, D_2O) δ -11.4. HRMS (ESI) m/z: $[M+H]^+$ Calcd for $C_{17}H_{23}N_5O_{13}P_2$ 568.0840; Found 568.0834.

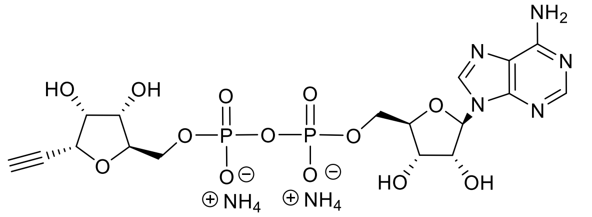

Scheme S3: Synthesis of 1"-α-*O*-methyl-2"-*O*-benzyl-ADPr

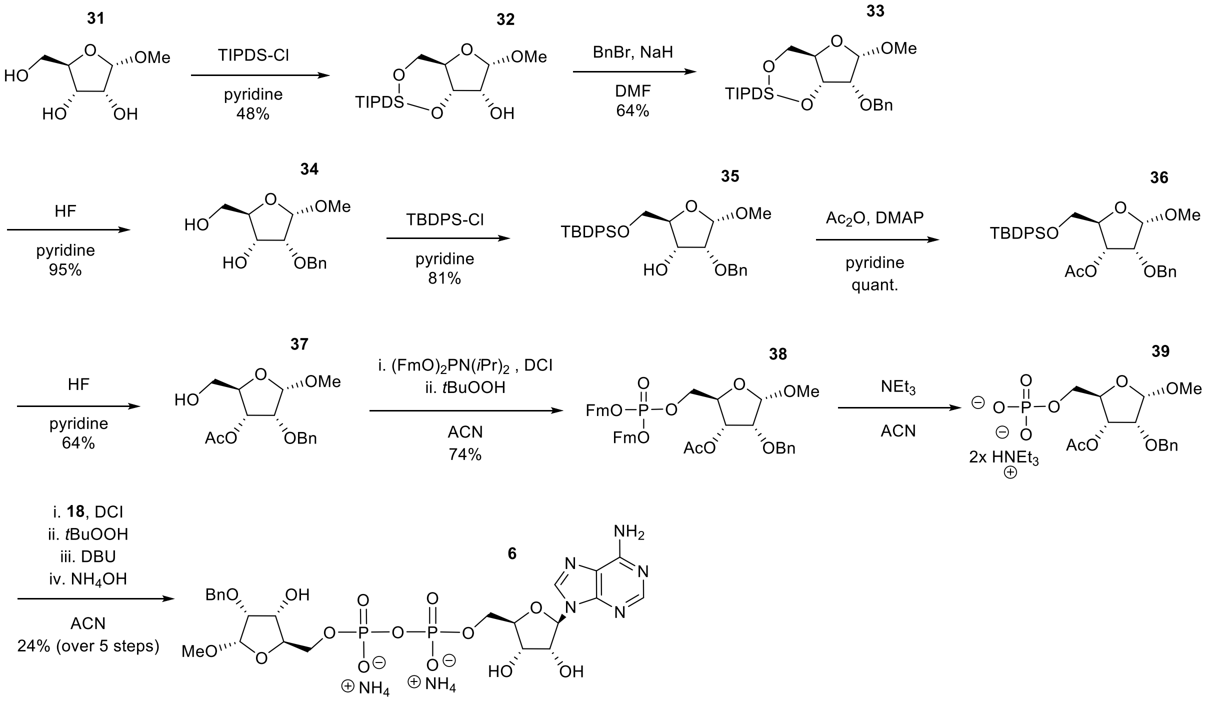

1-α-*O*-methyl-D-ribofuranoside (31) Commercially available 1-α-*O*-methyl-2,3,5-tri-*O*-acetyl-D-ribofuranoside

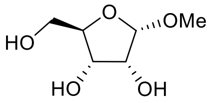

(8.6 grams, 29 mmol) was dissolved in methanol (190 mL), cooled to 0 °C and was brought under a nitrogen atmosphere. Additionally, sodium methoxide (NaOMe) (0.41 grams, 7.6 mmol, 0.40 eq) was slowly added and the resulting solution was allowed to stir at 0 °C for 3.5 hours while regularly checking reaction progress via TLC analysis. Upon completion, the mixture was quenched by the addition of solid carbon dioxide until pH 7 was reached. After addition of celite, the mixture was concentrated *in vacuo*. Purification by column chromatography (methanol/dichloromethane 0/1 → 1/4, v/v) gave compound **31** (3.89 grams, 23.7 mmol, 82%) as a light-yellow oil. TLC: R_f 0.5 (methanol/dichloromethane, 1/4, v/v). ^{1}H NMR (400 MHz, MeOD) δ 4.85 (d, *J* = 4.3 Hz, 1H), 4.03 – 3.96 (m, 2H), 3.94 (dd, *J* = 6.5, 3.1 Hz, 1H), 3.70 – 3.57 (m, 2H), 3.43 (s, 3H), 3.35 (s, 2H). ^{13}C NMR (101 MHz, MeOD) δ 104.6, 86.7, 73.1, 71.3, 63.3, 55.5. HRMS (ESI) m/z: $[M+Na]^+$ Calcd for $C_6H_{12}O_5Na$ 187.0577; Found 187.0577.

1-α-*O*-methyl-3,5-*O*-(1,1,3,3-tetraisopropyldisiloxane-1,3-diyl)-D-ribofuranoside (32) Compound **31** (1.30

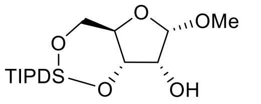

grams, 7.92 mmol) was co-evaporated thrice using dry pyridine whereafter it was dissolved in dry pyridine (12 mL, 0.65 M) and stirred under a nitrogen atmosphere at -20 °C. To the stirring and cooled solution is added 1,3-dichloro-1,1,3,3-tetraisopropyldisiloxane (TIPDS-Cl) (2.8 mL, 8.7 mmol, 1.1 eq.) dropwise over a period of 1 hour after which the solution was allowed to stir at -20 °C for another 2.5 hours. At this point TLC analysis indicated no further reaction progress and the reaction was quenched by the addition of water. The quenched mixture was then diluted with diethyl ether and the organic phased was washed with water multiple times to remove the pyridine. The organic phase dried over Na_2SO_4, filtered off and concentrated *in vacuo*. Purification by silica column chromatography (ethyl acetate/pentane, 0/1 → 1/9, v/v) gave compound **32** (1.53 grams, 3.77 mmol, 48%) as a colourless oil. R_f 0.5 (pentane/ethyl acetate, 19/1, v/v). ^{1}H NMR (400 MHz, $CDCl_3$) δ 4.87 (d, *J* = 4.2 Hz, 1H), 4.24 – 4.17 (m, 1H), 4.11 – 3.98 (m, 3H), 3.79 – 3.68 (m, 1H), 3.44 (s, 3H), 3.03 (d, *J* = 8.7 Hz, 1H), 1.12 – 1.02 (m, 28H). ^{13}C NMR (101 MHz, $CDCl_3$) δ 102.7, 83.2, 71.4, 71.1, 64.1, 55.2, 17.7, 17.6, 17.52, 17.49, 17.2, 17.1, 17.0, 16.9, 13.7, 13.5, 13.2, 12.6. HRMS (ESI) m/z: $[M+Na]^+$ Calcd for $C_{18}H_{38}O_6Si_2Na$ 429.2099; Found 429.2099.

1-α-*O*-methyl-2-*O*-benzyl-3,5-*O*-(1,1,3,3-tetraisopropyldisiloxane-1,3-diyl)-D-ribofuranoside (33)

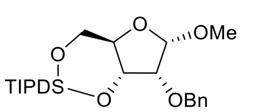

Compound **32** (2.71 grams, 6.66 mmol) was co-evaporated thrice using dry and distilled toluene, whereafter it was dissolved in dry dimethylformamide (DMF) (40 mL, 0.17 M). The resulting solution was flushed with nitrogen gas and left under nitrogen gas while stirring, after which it was cooled to 0 °C. Once cooled, benzyl bromide (2.4 mL, 20 mmol, 3.0 eq.) was added whereafter sodium hydride (60 w/w % dispersion in mineral oil) (0.32 grams, 8.0 mmol,

1.2 eq.) was added. The reaction mixture was allowed to stir at 0 °C for 40 minutes, whereafter TLC-analysis indicated full consumption of the starting material. The reaction was quenched by the addition of ice-water and subsequently extracted using diethyl ether. The organic phase was washed with brine and dried over Na_2SO_4, filtered off and concentrated *in vacuo*. Purification by silica column chromatography (diethyl ether/pentane, 1/99 → 1/3, v/v) gave compound **33** (2.11 grams, 4.24 mmol, 64%). R_f 0.4 (pentane/ethyl acetate, 19/1, v/v). ^{1}H NMR (400 MHz, $CDCl_3$) δ 7.44 – 7.38 (m, 2H), 7.37 – 7.23 (m, 3H), 4.94 (d, *J* = 3.9 Hz, 1H), 4.84 (d, *J* = 12.8 Hz, 1H), 4.77 (d, *J* = 12.8 Hz, 1H), 4.25 (dd, *J* = 7.1, 5.6 Hz, 1H), 4.19 – 4.10 (m, 1H), 3.98 (dd, *J* = 12.5, 3.0 Hz, 1H), 3.92 – 3.83 (m, 2H), 3.48 (s, 3H), 1.13 – 0.95 (m, 28H). ^{13}C NMR (101 MHz, $CDCl_3$) δ 138.6, 128.4, 127.8, 127.6, 103.0, 81.8, 77.1, 72.8, 70.8, 61.9, 56.0, 17.6, 17.5, 17.4, 17.3, 17.1, 17.0, 13.7, 13.4, 13.0, 12.7. HRMS (ESI) m/z: $[M+Na]^+$ Calcd for $C_{25}H_{44}O_6Si_2Na$ 519.2569; Found 519.2567.

1-α-*O*-methyl-2-*O*-benzyl-D-ribofuranoside (34) Compound **33** (2.11 grams, 4.24 mmol) was dissolved in dry pyridine (43 mL, 0.10 M) and brought under a nitrogen atmosphere where it was stirred. While stirring, added to the solution was HF in pyridine (70% w/w) (4.3 mL) and the corresponding reaction mixture was allowed to stir at room temperature for 1 hour after which TLC analysis indicated full consumption of the starting material. The reaction was then quenched by the addition of aq. sat. sodium bicarbonate solution, and the product was extracted using dichloromethane. The combined organic phases were dried over $MgSO_4$, filtered off and concentrated *in vacuo*. Purification by silica column chromatography (methanol/dichloromethane, 1/99 → 1/20, v/v) gave compound **34** (1.02 grams, 4.02 mmol, 95%). R_f 0.4 (dichloromethane/methanol, 19/1, v/v). ^{1}H NMR (400 MHz, $CDCl_3$) δ 7.41 – 7.28 (m, 5H), 4.89 (d, *J* = 4.1 Hz, 1H), 4.75 (d, *J* = 11.8 Hz, 1H), 4.61 (d, *J* = 11.8 Hz, 1H), 4.20 – 4.13 (m, 1H), 4.08 (s, 1H), 3.82 (dd, *J* = 6.2, 4.1 Hz, 1H), 3.75 (dd, *J* = 12.0, 3.2 Hz, 1H), 3.65 (dd, *J* = 12.0, 3.8 Hz, 1H), 3.43 (s, 3H), 3.04 (s, 1H), 2.26 – 2.05 (m, 1H). ^{13}C NMR (101 MHz, $CDCl_3$) δ 137.2, 128.6, 128.33, 128.27, 102.7, 86.5, 78.1, 72.5, 69.6, 62.9, 55.2. HRMS (ESI) m/z: $[M+Na]^+$ Calcd for $C_{13}H_{18}O_5Na$ 277.1046; Found 277.1047.

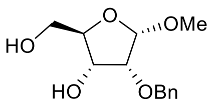

1-α-*O*-methyl-2-*O*-benzyl-5-*O*-(*tert*-butyl-di-phenylsilyl)-D-ribofuranoside (35) Compound **34** (51 mg, 0.20 mmol) was dissolved in dry pyridine (1.0 mL, 0.20 M) and the resulting solution was stirred under a nitrogen atmosphere. While stirring, to the solution was added *tert*-butyldimethylsilyl chloride (TBDPS-Cl) (62 μL, 0.24 mmol, 1.2 eq.) and the resulting mixture was stirred at room temperature for 21 hours at which point TLC analysis indicated full consumption of the starting material. The reaction was quenched by the addition of water, after which it was diluted with ethyl acetate. The organic phase was washed thrice with water and once with brine, after which it was dried over $MgSO_4$, filtered off and concentrated *in vacuo*. Purification by silica column chromatography (ethyl acetate/pentane) gave compound **35** (80 mg, 0.16 mmol, 81%). R_f 0.6 (pentane/ethyl acetate, 7/3, v/v). ^{1}H NMR (400 MHz, $CDCl_3$) δ 7.66 – 7.58 (m, 4H), 7.48 – 7.28 (m, 11H), 4.92 (d, *J* = 4.1 Hz, 1H), 4.77 (d, *J* = 12.1 Hz, 1H), 4.63 (d, *J* = 12.1 Hz, 1H), 4.23 (ddd, *J* = 9.0, 5.8, 1.5 Hz, 1H), 4.18 (td, *J* = 2.9, 1.4 Hz, 1H), 4.01 (dd, *J* = 5.9, 4.1 Hz, 1H), 3.79 – 3.64 (m, 2H), 3.44 (s, 3H), 3.03 (d, *J* = 9.1 Hz, 1H), 0.96 (s, 9H). ^{13}C NMR (101 MHz, $CDCl_3$) δ 137.4, 135.8, 135.7, 133.2, 133.0, 130.0, 129.9, 128.7, 128.3, 127.9, 127.8, 102.7, 87.1, 78.4, 72.5, 70.1, 64.2, 55.3, 26.9, 19.3. HRMS (ESI) m/z: $[M+Na]^+$ Calcd for $C_{29}H_{36}O_5SiNa$ 515.2224; Found 515.2224.

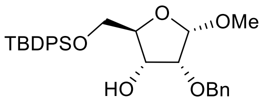

1-α-*O*-methyl-2-*O*-benzyl-3-*O*-acetyl-5-*O*-(*tert*-butyl-di-phenylsilyl)-D-ribofuranoside (36) Compound **35** (72 mg, 0.15 mmol) was co-evaporated thrice with dry and distilled toluene, after which it was dissolved in dry pyridine (1.5 mL, 0.10 M) and stirred under a nitrogen atmosphere at 0 °C. To the stirring mixture was then added acetic anhydride (30 μL, 0.30 mmol, 2.0 eq.) and DMAP (2.0 mg, 15 μmol, 0.10 eq.) after which the resulting mixture was allowed to warm to room temperature and stirred for 20 hours. At this point TLC analysis indicated full consumption of the starting material and the reaction was quenched by the addition of water. The mixture was then diluted with diethyl ether after which the organic phase was washed with water and then brine. The organic phase was dried over $MgSO_4$, filtered off and concentrated *in vacuo*. Purification by silica column chromatography (diethyl ether/pentane, 1/9 → 1/1, v/v) gave compound **36** (78 mg, 0.15 mmol, quant.). R_f 0.4 (pentane/diethyl ether, 3/2, v/v). ^{1}H NMR (400 MHz, $CDCl_3$) δ 7.67 – 7.59 (m, 4H), 7.47 – 7.27 (m, 11H), 5.33 (dd, *J* = 6.8, 2.4 Hz, 1H), 4.87 (d, *J* = 4.5 Hz, 1H), 4.69 (d, *J* = 12.6 Hz, 1H), 4.60 (d, *J* = 12.6 Hz, 1H), 4.12 (q, *J* = 2.8 Hz, 1H), 3.99 (dd, *J* = 6.8, 4.5 Hz, 1H), 3.83 (dd, *J* = 11.1, 2.8 Hz, 1H), 3.73 (dd, *J* = 11.1, 3.0 Hz, 1H), 3.46 (s, 3H), 2.14 (s, 3H), 0.96 (s, 9H). ^{13}C NMR (101 MHz, $CDCl_3$) δ 171.2, 137.6, 135.8, 135.7, 133.2, 133.1, 129.9, 129.8, 128.6, 128.1, 128.1, 127.9, 127.8, 102.5, 83.3, 77.4, 73.2, 70.5, 64.0, 55.6, 26.8, 21.4, 19.3. HRMS (ESI) m/z: $[M+Na]^+$ Calcd for $C_{31}H_{38}O_6SiNa$ 557.2330; Found 557.2330.

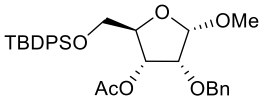

1-α-*O*-methyl-2-*O*-benzyl-3-*O*-acetyl-D-ribofuranoside (37) Compound **36** (78 mg, 0.15 mmol) was dissolved in dry pyridine (1.5 mL, 0.1 M) and stirred at 0 °C under a nitrogen atmosphere when HF in pyridine (70% w/w) (0.15 mL) was added. The resulting mixture was allowed to stir for 2 hours when TLC analysis indicated full consumption of the starting material. The reaction was quenched by the careful addition of sat. aq. sodium bicarbonate solution after which it was diluted by the addition of ethyl acetate. The organic phase was washed with sat. aq. sodium bicarbonate solution twice and with brine once. The organic phase was then dried over $MgSO_4$, filtered off and concentrated *in vacuo*. Purification by silica column chromatography (ethyl acetate/pentane, 3/7 → 4/1, v/v) gave compound **37** (28 mg, 0.094 mmol, 64%). R_f 0.3 (pentane/ethyl acetate, 2/3, v/v). ^{1}H NMR (400 MHz, $CDCl_3$) δ 7.39 – 7.28 (m, 5H), 5.10 (dd, *J* = 7.1, 3.2 Hz, 1H), 4.85 (d, *J* = 4.4 Hz, 1H), 4.67 (d, *J* = 12.3 Hz, 1H), 4.58 (d, *J* = 12.4 Hz, 1H), 4.12 (q, *J* = 3.4 Hz, 1H), 3.85 (dd, *J* = 7.1, 4.4 Hz, 1H), 3.81 – 3.68 (m, 2H), 3.45 (s, 3H), 2.14 (s, 3H), 2.08 (bs, 1H). ^{13}C NMR (101 MHz, $CDCl_3$) δ 171.4, 137.41, 128.6, 128.2, 102.4, 82.9, 77.2, 73.3, 70.2, 62.6, 55.5, 21.3. HRMS (ESI) m/z: $[M+Na]^+$ Calcd for $C_{15}H_{20}O_6Na$ 319.1152; Found 319.1154.

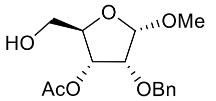

1-α-*O*-methyl-2-*O*-benzyl-3-*O*-acetyl-5-*O*-(di(9*H*-fluoren-9-yl))-phosphoryl-D-ribofuranoside (38) To compound **37** (28 mg, 0.094 mmol) was added DCI (33 mg, 0.28 mmol, 3.0 eq.) after which the mixture was co-evaporated thrice with dry and distilled toluene. Next, the mixture was dissolved in dry acetonitrile (0.62 mL, 0.15 M) and stirred under a nitrogen atmosphere. In another flask, a solution of $(FmO)_2PN(iPr)_2$ was prepared (0.32 M in acetonitrile) and some of this (0.44 mL, 0.14 mmol, 1.5 eq.) was added to the stirring mixture dropwise. The resulting reaction mixture was then allowed to stir at room temperature for 1.5 hours. Upon completion, to the stirring mixture was added *t*BuOOH (5.5 M in decanes) (85 µL, 0.47 mmol, 5.0 eq.) and the resulting solution was stirred at room temperature for 35 minutes, after which the reaction was quenched by the addition of water. The solution was diluted using dichloromethane, and the organic phase was washed with brine, after which it was dried over Na_2SO_4, filtered off and concentrated *in vacuo*. Purification by silica column chromatography (ethyl acetate/pentane, 3/7 → 3/1, v/v) gave compound **38** (51 mg, 0.069 mmol, 74%). R_f 0.6 (pentane/ethyl acetate, 3/7, v/v). ^{1}H NMR (400 MHz, $CDCl_3$) δ 7.75 – 7.68 (m, 4H), 7.58 – 7.44 (m, 4H), 7.42 – 7.31 (m, 4H), 7.30 – 7.19 (m, 9H), 4.97 (dd, *J* = 7.1, 3.2 Hz, 1H), 4.68 (d, *J* = 4.4 Hz, 1H), 4.53 (d, *J* = 12.1 Hz, 1H), 4.47 (d, *J* = 12.1 Hz, 1H), 4.32 – 4.18 (m, 4H), 4.17 – 4.00 (m, 5H), 3.71 (dd, *J* = 7.2, 4.5 Hz, 1H), 3.36 (s, 3H), 2.08 (s, 3H). ^{13}C NMR (101 MHz, $CDCl_3$) δ 171.0, 143.1, 143.0, 143.02, 143.00, 141.48, 141.46, 141.43, 137.2, 128.6, 128.3, 128.2, 128.1, 127.3, 125.20, 125.18, 125.15, 120.2, 120.1, 102.3, 80.7 (d, *J* = 7.3 Hz), 77.0, 73.4, 69.6 (d, *J* = 2.5 Hz), 69.51, 69.49, 69.48, 67.2 (d, *J* = 5.8 Hz), 55.5, 48.0 (d, *J* = 3.3 Hz), 47.9 (d, *J* = 3.3 Hz), 21.1. ^{31}P NMR (162 MHz, $CDCl_3$) δ -1.9. HRMS (ESI) m/z: $[M+Na]^+$ Calcd for $C_{43}H_{41}O_9PNa$ 755.2380; Found 755.2383.

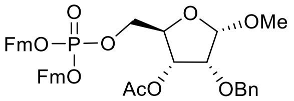

1-α-*O*-methyl-2-*O*-benzyl-3-*O*-acetyl-5-*O*-phosphate-D-ribofuranoside (39) Compound **38** (51 mg, 0.069 mmol) was dissolved in dry acetonitrile (1.6 mL, 0.044 M) and brought under a nitrogen atmosphere. To the stirring mixture was then added triethylamine (0.17 mL, 1.2 mmol, 17 eq.) and the mixture was allowed to stir at room temperature for 48 hours, after which it was directly concentrated in vacuo. The crude product was used directly in the next reaction without any further purification.

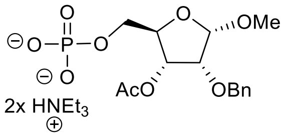

1”-α-*O*-methyl-2”-*O*-benzyl-ADPr (6) Crude compound **39** (~0.069 mmol) was co-evaporated thrice using a solvent system consisting of 1/1 dry acetonitrile/dry pyridine after which it was co-evaporated once using dry acetonitrile and to it was added DCI (21 mg, 0.17 mmol, 2.5 eq.). The mixture was co-evaporated thrice with dry acetonitrile after which it was dissolved in dry acetonitrile (1.3 mL, 0.055 M) and stirred vigorously under a nitrogen atmosphere. In another flask, phosphoramidite **18** was co-evaporated thrice with dry and distilled toluene after which it was dissolved in dry acetonitrile to create a stock solution (0.22 M in acetonitrile). A part of this stock solution (0.32 mL, 0.069 mmol, 1.0 eq.) was then added dropwise to the solution containing the phosphate. The resulting mixture was stirred at room temperature for 1 hour and 20 minutes at which point TLC analysis indicated full consumption of the phosphoramidite. Subsequently, *t*BuOOH (5.5 M in decanes) (26 µL, 0.15 mmol, 2.1 eq.) was added to the mixture and the solution was stirred at room temperature for 1 hour, at which point another and equal batch of *t*BuOOH (26 µL, 0.15 mmol, 2.1 eq.) was added to the mixture. After another hour and 15 minutes, ^{31}P-NMR indicated full conversion of the phosphite and to the reaction mixture was added DBU (0.52 µL, 0.35 mmol, 5.0 eq.). The resulting solution was stirred at room temperature for 1 hour and 10 minutes at which point aqueous ammonium hydroxide (28% ammonia, w/w) (1.3 mL) was added.

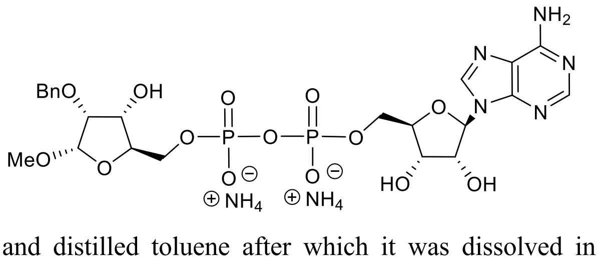

The resulting yellow clear solution was stirred at room temperature for 48 hours, at which point LCMS indicated full deprotection of the construct. The solution was then diluted with water and ethyl acetate and the aqueous phase was washed multiple times with ethyl acetate until the organic phase returned clear. The aqueous phase was directly concentrated *in vacuo*. Purification by gel filtration (HW-40, NH_4OAc buffer) afforded compound **6** (11.4 mg, 16.3 µmol, 24% (over 5 steps)) as the ammonium salt as a white foam. ^1H NMR (500 MHz, D_2O) δ 8.52 – 8.46 (m, 1H), 8.16 (m, 1H), 7.39 – 7.27 (m, 5H), 6.10 – 6.05 (m, 1H), 4.78 – 4.74 (m, 1H), 4.73 – 4.67 (m, 1H), 4.56 (d, *J* = 11.6 Hz, 1H), 4.50 (dd, *J* = 5.1, 3.8 Hz, 1H), 4.42 (d, *J* = 11.6 Hz, 1H), 4.37 (s, 1H), 4.30 – 4.17 (m, 4H), 4.05 – 3.92 (m, 2H), 3.88 (dd, *J* = 6.1, 4.5 Hz, 1H), 3.29 (s, 3H). ^{13}C NMR (126 MHz, D_2O) δ 155.3, 152.6, 148.9, 139.7, 136.7, 128.5, 128.3, 128.2, 118.5, 102.3, 86.9, 83.9 (d, *J* = 7.1 Hz), 83.7 (d, *J* = 4.8 Hz), 77.8, 74.3, 72.4, 70.2, 68.5, 65.6 (d, *J* = 3.9 Hz), 65.2 (d, *J* = 4.3 Hz), 55.1. ^{31}P NMR (202 MHz, D_2O) δ -11.3. HRMS (ESI) m/z: $[M+H]^+$ Calcd for $C_{23}H_{32}N_5O_{14}P_2$ 664.1416; Found 664.1415.

Scheme S4: Synthesis of 1”-α-azido-2”-*O*-benzyl-ADPr

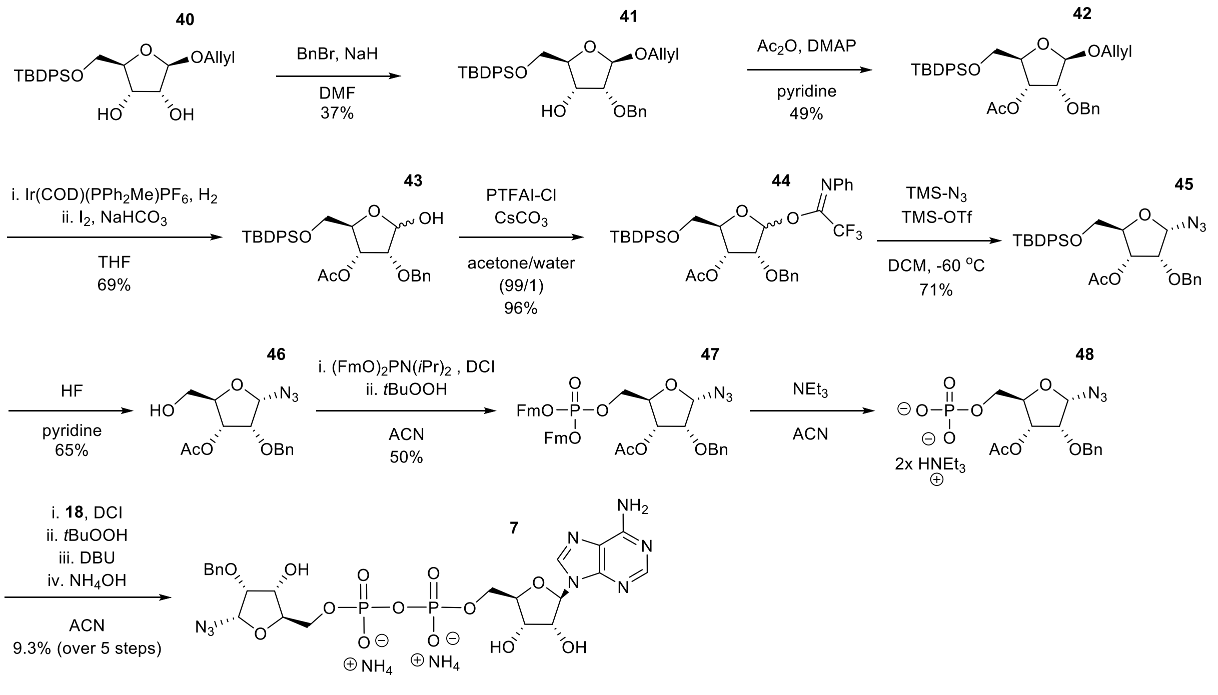

1-β-*O*-allyl-5-*O*-(*tert*-butyl-diphenylsilyl)-D-ribofuranoside (40) Compound **40** was prepared following a procedure previously described.[6] All spectra are in full accordance with literary precedence. ^{1}H NMR (400 MHz, $CDCl_3$) δ 7.73 – 7.64 (m, 4H), 7.47 – 7.34 (m, 6H), 5.81 (dddd, *J* = 17.2, 10.4, 6.2, 5.2 Hz, 1H), 5.20 (dq, *J* = 17.2, 1.6 Hz, 1H), 5.13 (dq, *J* = 10.4, 1.4 Hz, 1H), 4.98 (d, *J* = 1.0 Hz, 1H), 4.39 – 4.27 (m, 1H), 4.19 – 4.10 (m, 1H), 4.08 (dd, *J* = 4.8, 2.6 Hz, 1H), 4.03 (dd, *J* = 6.1, 5.1 Hz, 1H), 3.92 (ddt, *J* = 12.8, 6.2, 1.4 Hz, 1H), 3.86 – 3.73 (m, 2H), 2.81 (d, *J* = 3.3 Hz, 1H), 2.46 (d, *J* = 5.5 Hz, 1H), 1.07 (s, 9H). ^{13}C NMR (101 MHz, $CDCl_3$) δ 135.7, 134.1, 133.34, 133.30, 130.0, 129.9, 127.91, 127.90, 117.5, 106.3, 83.2, 75.5, 72.9, 68.6, 65.4, 27.0, 19.4. HRMS (ESI) m/z: $[M+Na]^+$ Calcd for $C_{24}H_{32}O_5SiNa$ 451.1911; Found 451.1909.

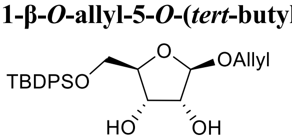

1-β-*O*-allyl-2-*O*-benzyl-5-*O*-(*tert*-butyl-diphenylsilyl)-D-ribofuranoside (41) Compound **40** (14.2 grams, 33.2 mmol) was co-evaporated with anhydrous toluene and then dissolved in anhydrous DMF (200 mL, 0.166 M) and subsequently cooled to 0 °C. Benzyl bromide (7.9 mL, 66 mmol, 2.0 eq.) was slowly added to the solution, followed by sodium hydride (60% w/w dispersion in mineral oil) (1.5 grams, 37 mmol, 1.1 eq.). The resulting solution was stirred for two hours at room temperature when TLC analysis indicated full conversion of the starting material into a mixture of both mono-benzylated constructs as well as the di-benzylated construct, after which the reaction mixture was quenched using water (100 mL) and extracted with ethyl acetate (100 mL). The combined organic fractions were dried over $MgSO_4$, filtered off and concentrated *in vacuo*. Purification via silica gel chromatography (pentane/diethyl ether, 19/1 → 7/3, v/v) yielded compound **41** (6.40 grams, 12.3 mmol, 37%) as a colourless clear oil. R_f 0.4 (pentane/diethyl ether, 4/1, v/v). ^{1}H NMR (400 MHz, $CDCl_3$) δ 7.74 – 7.66 (m, 4H), 7.45 – 7.29 (m, 11H), 5.82 (dddd, *J* = 16.8, 10.4, 6.2, 5.0 Hz, 1H), 5.21 (dq, *J* = 17.2, 1.6 Hz, 1H), 5.14 (dq, *J* = 10.4, 1.4 Hz, 1H), 5.08 (d, *J* = 1.6 Hz, 1H), 4.74 (d, *J* = 11.7 Hz, 1H), 4.63 (d, *J* = 11.7 Hz, 1H), 4.30 (dt, *J* = 8.1, 5.4 Hz, 1H), 4.18 (ddt, *J* = 12.9, 5.1, 1.6 Hz, 1H), 4.04 (q, *J* = 4.9 Hz, 1H), 4.00 – 3.89 (m, 2H), 3.82 (dd, *J* = 11.0, 4.1 Hz, 1H), 3.76 – 3.65 (m, 1H), 2.55 (d, *J* = 8.1 Hz, 1H), 1.05 (s, 9H). ^{13}C NMR (101 MHz, $CDCl_3$) δ 137.3, z135.8, 134.2, 133.5, 129.8, 129.8, 128.7, 128.3, 128.1, 127.82, 127.80, 117.3, 104.1, 84.9, 82.5, 72.9, 71.4, 68.7, 64.9, 26.9, 19.4. HRMS (ESI) m/z: $[M+Na]^+$ Calcd for $C_{31}H_{38}O_5SiNa$ 541.2381; Found 541.2377.

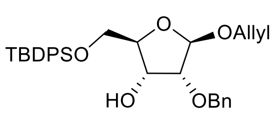

1-β-*O*-allyl-2-*O*-benzyl-3-*O*-acetyl-5-*O*-(*tert*-butyl-diphenylsilyl)-D-ribofuranoside (42) Compound **41** (2.6 grams, 5.0 mmol) was co-evaporated with anhydrous toluene and then dissolved in anhydrous pyridine (30 mL, 0.17 M). Acetic anhydride (1.4 mL, 15 mmol, 3.0 eq.) and 4-(dimethylamino)pyridine (DMAP) (0.12 grams, 1.0 mmol, 0.20 eq.) were added and the resulting solution was allowed to stir overnight at room temperature. The reaction mixture was then cooled to 0 °C and diluted with water (30 mL) and washed with diethyl ether (50 mL)

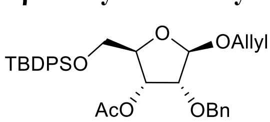

and an aqueous solution of HCl (1.0 M) (50 mL). The combined organic fractions were dried over $MgSO_4$, filtered off and concentrated *in vacuo*. Purification via silica gel chromatography (pentane/diethyl ether, 19/1 → 17/3, v/v) yielded compound **42** (1.37 grams, 2.45 mmol, 49%) as colourless clear oil. Rf 0.55 (pentane/diethyl ether, 4/1, v/v); ^{1}H NMR (400 MHz, $CDCl_3$) δ 7.72 – 7.61 (m, 4H), 7.45 – 7.26 (m, 11H), 5.82 (dddd, *J* = 17.3, 10.4, 6.0, 5.0 Hz, 1H), 5.27 (t, *J* = 5.1 Hz, 1H), 5.22 (dq, *J* = 17.2, 1.7 Hz, 1H), 5.13 (dq, *J* = 10.4, 1.4 Hz, 1H), 5.05 (d, *J* = 2.3 Hz, 1H), 4.58 (dd, *J* = 12.3, 1.0 Hz, 2H), 4.24 (q, *J* = 5.0 Hz, 1H), 4.20 – 4.14 (m, 2H), 3.95 (ddt, *J* = 12.9, 6.0, 1.4 Hz, 1H), 3.73 (dd, *J* = 4.9, 1.2 Hz, 2H), 2.07 (s, 3H), 1.03 (s, 9H). ^{13}C NMR (101 MHz, $CDCl_3$) δ 170.5, 137.8, 135.79, 135.76, 134.1, 133.4, 133.3, 129.9, 129.8, 128.6, 128.0, 127.9, 127.8, 127.8, 117.2, 105.2, 81.8, 81.3, 73.6, 73.2, 68.9, 64.8, 26.9, 21.0, 19.3. HRMS (ESI) m/z: $[M+Na]^+$ Calcd for $C_{33}H_{40}O_6SiNa$ 583.2486; Found 583.2484.

2-*O*-benzyl-3-*O*-acetyl-5-*O*-(*tert*-butyl-diphenylsilyl)-D-ribofuranoside (43) Compound **42** (0.56 grams, 1.0 mmol) was co-evaporated with anhydrous toluene and dissolved in anhydrous tetrahydrofuran (5.0 mL, 0.20 M) under an argon atmosphere. In another flask, Ir(COD)(PPh_2Me)PF_6 (4.2 mg, 5.0 μmol, 0.0050 eq.) was dissolved in anhydrous tetrahydrofuran (5.0 mL, 0.0010 M) and flushed with argon. The solution containing the catalyst was first flushed with H_2 until the solution became completely colourless and was subsequently flushed with argon. This process was repeated until the solution stayed completely clear after flushing with argon. The solution containing the catalyst was then added dropwise to the solution of compound **42** after which the resulting mixture was allowed to stir at room temperature. Isomerization of the starting material was followed via TLC analysis. When the starting material had fully isomerized, aq. sat. sodium bicarbonate solution (7 mL) and solid iodine (0.31 grams, 1.2 mmol, 1.2 eq.) were added to the reaction mixture, respectively. After stirring for 30 minutes at room temperature the reaction was quenched using a solution of sodium thiosulphate (20 mL) and diluted with dichloromethane (30 mL) which was washed with a solution of sodium thiosulphate until the organic phase had fully discoloured. The organic phase was dried over $MgSO_4$, filtered off and concentrated *in vacuo*. Purification via silica gel chromatography (pentane/ethyl acetate, 9/1 → 7/3, v/v) yielded compound **43** (0.357 grams. 0.686 mmol, 69%) as a slightly yellow clear oil as 1:1 mixture of anomers. Rf 0.25 (pentane/ethyl acetate, 4:1, v/v). Both anomers are reported here. As a consequence, the total integral for e.g. δ 7.70 – 7.59 is doubled and given as 8H, as opposed to the expected 4H for a single anomer due to the mixture being 1:1. With this in mind, ^{13}C-NMR also reports both anomers; ^{1}H NMR (400 MHz, $CDCl_3$) δ 7.70 – 7.59 (m, 8H), 7.47 – 7.27 (m, 22H), 5.47 – 5.34 (m, 3H), 5.32 (dd, *J* = 7.1, 1.9 Hz, 1H), 4.62 (m, 2x2H), 4.24 (dt, *J* = 5.6, 3.0 Hz, 2H), 4.20 (dd, *J* = 5.8, 4.5 Hz, 1H), 4.15 – 4.06 (m, 1H), 3.82 (dd, *J* = 11.3, 3.3 Hz, 1H), 3.77 – 3.72 (m, 2H), 3.68 (dd, *J* = 11.2, 2.9 Hz, 1H), 3.60 (d, *J* = 8.8 Hz, 1H), 3.26 (d, *J* = 7.2 Hz, 1H), 2.10 (s, 3H), 2.07 (s, 3H), 1.05 (s, 9H), 0.98 (s, 9H). ^{13}C NMR (101 MHz, $CDCl_3$) δ 170.4, 170.3, 137.7, 137.2, 135.9, 135.74, 135.72, 135.67, 130.2, 130.1, 130.0, 129.9, 128.7, 128.6, 128.3, 128.0, 127.9, 127.9, 100.9, 96.2, 82.4, 82.4, 82.1, 77.2, 73.3, 73.2, 72.7, 72.2, 64.0, 63.9, 26.9, 26.9, 21.1, 21.0, 19.3, 19.2. HRMS (ESI) m/z: $[M+Na]^+$ Calcd for $C_{30}H_{36}O_6SiNa$ 543.2173; Found 543.2173.

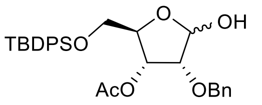

1-α,β-*O*-((*N*-phenyl)-2,2,2-trifluoroacetamido)-2-*O*-benzyl-3-*O*-acetyl-5-*O*-(*tert*-butyl-diphenylsilyl)-D-ribofuranoside (44) Compound **43** (2.65 grams, 5.08 mmol) was dissolved in a mixture of acetone and water (51 mL, 0.1 M, 50/1, acetone/water, v/v) and subsequently cooled to 0 °C. Next, Cs_2CO_3 (2.5 grams, 7.6 mmol, 1.5 eq.) and *N*-phenyltrifluoroacetimidoyl chloride (PTFAI-Cl) (1.2 mL, 7.6 mmol, 1.5 eq.) were added and the resulting solution was stirred at 0 °C for 20 hours. The reaction mixture was filtered off and concentrated *in vacuo*. Purification via silica gel chromatography (pentane/diethyl ether, 1/0 → 9/1, v/v) (with 1% triethylamine, v/v) yielded compound **44** (3.38 grams, 4.89 mmol, 96%) as a yellow oil. Rf 0.45 (pentane/diethyl ether, 9/1, v/v); ^{1}H NMR (400 MHz, $CDCl_3$) δ 7.71 – 7.54 (m, 4H), 7.46 – 7.36 (m, 6H), 7.35 – 7.25 (m, 7H), 7.10 (td, *J* = 7.4, 1.0 Hz, 1H), 6.86 – 6.79 (m, 2H), 6.30 (bs, 1H), 5.28 (t, *J* = 5.2 Hz, 1H), 4.63 (q, *J* = 12.2, 8.9 Hz, 2H), 4.50 (dd, *J* = 5.2, 2.2 Hz, 1H), 4.38 (q, *J* = 4.5 Hz, 1H), 3.80 (qd, *J* = 11.3, 4.3 Hz, 2H), 2.09 (s, 3H), 1.03 (s, 9H). ^{13}C NMR (101 MHz, $CDCl_3$) δ 170.4, 143.8, 137.3, 135.8, 135.7, 135.7, 133.1, 132.9, 130.0, 129.9, 128.8, 128.7, 128.6, 128.2, 127.99, 127.95, 127.93, 127.90, 127.87, 127.5, 124.4, 119.6, 102.9, 83.5, 80.3, 73.3, 72.3, 63.9, 46.3, 26.84, 26.78, 20.9, 19.3. HRMS (ESI) m/z: $[M\text{-imidate}+OH+Na]^+$ Calcd for $C_{30}H_{36}O_6SiNa$ 543.2173; Found 543.2176.

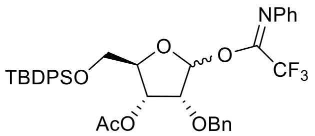

1-α-azido-2-*O*-benzyl-3-*O*-acetyl-5-*O*-(*tert*-butyl-diphenylsilyl)-D-ribofuranoside (45) Compound **44** (0.138 grams, 0.200 mmol) was co-evaporated with anhydrous toluene and dissolved in anhydrous dichloromethane (6.0 mL, 0.033 M) in a flame-dried flask containing activated molecular sieves (3Å, rods). Next, trimethylsilyl azide (TMS-N_3) (0.10 mL, 0.80 mmol, 4.0 eq.) was added and the resulting solution was stirred for 90 minutes before cooling to

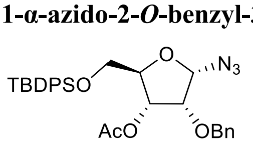

-60 °C. Once fully cooled, a stock solution containing trimethylsilyl triflate (TMS-OTf) (0.1 M in dry dichloromethane) (0.50 mL, 0.050 mmol, 0.25 eq.) was added in one go and the resulting solution was stirred for 20 hours at -60 °C. The reaction mixture was then quenched with triethylamine (0.06 mL), filtered off and concentrated *in vacuo*. Purification via silica gel chromatography (pentane/diethyl ether, 1/0 → 9/1, v/v) gave compound **45** (77 mg, 0.14 mmol, 71%) as white cloudy oil. Rf 0.5 (pentane/diethyl ether, 17:3, v/v); ^{1}H NMR (400 MHz, $CDCl_3$) δ 7.62 (t, *J* = 5.8 Hz, 4H), 7.48 – 7.25 (m, 11H), 5.40 (dd, *J* = 6.1, 2.2 Hz, 1H), 5.28 (d, *J* = 5.2 Hz, 1H), 4.68 (d, *J* = 12.2 Hz, 1H), 4.60 (d, *J* = 12.2 Hz, 1H), 4.28 (q, *J* = 2.5 Hz, 1H), 4.24 (dd, *J* = 6.0, 5.3 Hz, 1H), 3.76 (t, *J* = 2.6 Hz, 2H), 2.14 (s, 3H), 0.96 (s, 9H). ^{13}C NMR (101 MHz, $CDCl_3$) δ 170.8, 137.2, 135.8, 135.7, 135.6, 132.9, 132.7, 130.1, 130.0, 129.9, 128.7, 128.7, 128.3, 128.2, 128.0, 127.94, 127.92, 90.5, 84.8, 78.1, 73.4, 70.9, 63.9, 29.8, 26.8, 21.2, 19.2. HRMS (ESI) m/z: $[M+Na]^+$ Calcd for $C_{30}H_{35}N_3O_5SiNa$ 568.2238; Found 568.2236.

1-α-azido-2-*O*-benzyl-3-*O*-acetyl-D-ribofuranoside (46) Compound **45** (0.88 grams, 1.6 mmol) was dissolved in anhydrous pyridine (16 mL, 0.10 M) and to it was added HF-pyridine (70%, w/w) (1.6 mL). The resulting solution was stirrzed at room temperature for 1 hour. The reaction mixture was quenched with aq. sat. sodium bicarbonate solution until bubbling seized. The resulting solution was then diluted with ethyl acetate (10 mL) and washed twice with aq. sat. sodium bicarbonate solution (20 mL) and brine (20 mL). The organic phase was dried over $MgSO_4$, filtered off and concentrated *in vacuo*. Purification via silica gel chromatography (pentane/ethyl acetate, 7/3 → 1/1, v/v) afforded compound **46** (0.322 grams, 1.05 mmol, 65%) as a slightly cloudy oil. Rf 0.45 (pentane/ethyl acetate, 1/1, v/v); ^{1}H NMR (400 MHz, $CDCl_3$) δ 7.42 – 7.27 (m, 5H), 5.28 (d, *J* = 5.0 Hz, 1H), 5.19 (dd, *J* = 6.4, 3.4 Hz, 1H), 4.66 (d, *J* = 12.0 Hz, 1H), 4.60 (d, *J* = 12.0 Hz, 1H), 4.29 (q, *J* = 3.2 Hz, 1H), 4.09 (dd, *J* = 6.4, 5.0 Hz, 1H), 3.87 – 3.66 (m, 2H), 2.13 (s, 3H), 2.09 (bs, *J* = 8.4, 3.1 Hz, 1H). ^{13}C NMR (101 MHz, $CDCl_3$) δ 171.0, 137.1, 128.7, 128.3, 128.1, 90.5, 84.2, 77.9, 73.6, 70.3, 62.2, 21.1. HRMS (ESI) m/z: $[M+Na]^+$ Calcd for $C_{14}H_{17}N_3O_5Na$ 330.1060; Found 330.1058.

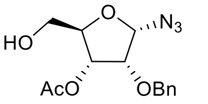

1-α-azido-2-*O*-benzyl-3-*O*-acetyl-5-*O*-(di(9*H*-fluoren-9-yl))-phosphoryl-D-ribofuranoside (47) Compound **46** (0.32 grams, 1.0 mmol) and DCI (0.37 grams, 3.1 mmol, 3.1 eq.) were combined in a flask and co-evaporated with anhydrous toluene and subsequently dissolved in anhydrous acetonitrile (6.3 mL, 0.15 M). In another flask, $(FmO)_2PN(iPr)_2$ (0.82 grams, 1.6 mmol, 1.5 eq.) was dissolved in anhydrous acetonitrile (5.0 mL, 0.32 M) and was added dropwise to the stirring solution of compound **46** and DCI. The resulting mixture was stirred at room temperature for 90 minutes. Then, to it was added *t*BuOOH (5.5 M in decanes) (1.0 mL, 5.5 mmol, 5.5 eq.) and the reaction was monitored via ^{31}P-NMR. After complete oxidation of the phosphite into the corresponding phosphate, the reaction mixture was quenched with water (10 mL) and diluted dichloromethane (20 mL). The organic phase was separated and dried over $MgSO_4$, filtered off and concentrated *in vacuo*. Purification via silica gel chromatography (pentane/ethyl acetate, 7/3 → 3/7, v/v) yielded an inseparable 3:2 (mol:mol) mixture of respectively compound **47** and the FmO H-phosphonate (0.520 mmol, 50%) as a clear oil which was used without further purification in the next reaction. Rf 0.55 (pentane/ethyl acetate, 3/3, v/v); ^{1}H NMR (400 MHz, $CDCl_3$) δ 7.78 – 7.66 (m, 4H), 7.57 – 7.25 (m, 17H), 5.04 – 4.95 (m, 2H), 4.47 (d, *J* = 1.9 Hz, 2H), 4.34 – 4.18 (m, 5H), 4.16 – 4.06 (m, 4H), 3.86 (dd, *J* = 6.3, 5.0 Hz, 1H), 2.07 (s, 3H). ^{13}C NMR (101 MHz, $CDCl_3$) δ 170.6, 143.1, 143.05, 143.03, 142.96, 141.51, 141.48, 141.45, 137.0, 128.7, 128.6, 128.3, 128.09, 128.08, 128.06, 127.99, 127.9, 127.3, 127.27, 127.2, 125.18, 125.15, 125.12, 125.10, 120.2, 120.1, 120.0, 90.3, 82.0, 81.9 (d, *J* = 7.3 Hz), 77.6, 73.7, 69.9, 69.4 (t, *J* = 6.2 Hz), 67.1 (d, *J* = 6.2 Hz), 48.0 (d, *J* = 2.7 Hz), 47.9 (d, *J* = 3.4 Hz), 21.0. ^{31}P NMR (122 MHz, aceton-capil) δ 9.7 (H-phosphonate), -1.2. IR: 2110, 1740, 1450; HRMS (ESI) m/z: $[M+Na]^+$ Calcd for $C_{42}H_{38}N_3O_8PNa$ 766.2289; Found 766.2282.

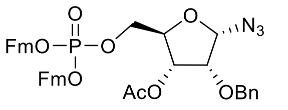

1-α-azido-2-*O*-benzyl-3-*O*-acetyl-5-*O*-phosphate-D-ribofuranoside (48) Compound **47** (0.20 mmol) was dissolved in anhydrous acetonitrile (5.0 mL, 0.044 M) and brought under nitrogen atmosphere. Then, triethylamine (0.47 mL, 3.4 mmol, 17 eq.) was added and the solution was allowed to stir at room temperature for 72 hours. The reaction mixture was then directly concentrated *in vacuo*. The crude product was co-evaporated with 1/1 anhydrous pyridine/anhydrous acetonitrile (2:2 mL) for a total of three times. The resulting crude product was used directly in the next reaction without any further purification.

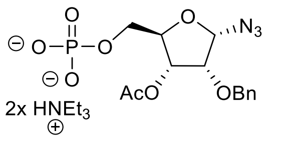

1”-α-azido-2”-*O*-benzyl-ADPr (7) To crude compound **48** (~0.20 mmol) was added DCI (59 mg, 0.50 mmol, 2.5 eq.) and the resulting mixture was co-evaporated thrice with anhydrous acetonitrile after which the mix was suspended in anhydrous acetonitrile (4.0 mL, 0.050 M) and stirred under an argon atmosphere. In another flask, compound **18** (142 mg, 0.200 mol, 1.0 eq.) was co-evaporated twice with anhydrous toluene after which it was dissolved in anhydrous acetonitrile (1.0 mL, 0.20 M) which was added dropwise to the suspension containing compound **48** and DCI. The resulting mixture was allowed to stir for one hour at room temperature after which *t*BuOOH (5.5 M in decanes) (76 µL, 0.42 mmol, 2.1 eq.) was added. The reaction mixture was then allowed to stir at room temperature for one hour, after which a second portion of *t*BuOOH (76 µL, 0.42 mmol, 2.1 eq.) was added and the reaction was monitored via ^{31}P-NMR. Upon complete conversion of the phosphite species to the corresponding protected phosphates, to the mixture was added DBU (0.15 mL, 1.0 mmol, 5.0 eq.) agfetr which the mixture was allowed to stir at room temperature for 30 minutes. At this point to the reaction mixture was added an aqueous solution of ammonium hydroxide (28% NH_3, w/w) (3.6 mL) and the resulting yellow suspension was allowed to stir for 19 hours after which it was diluted with water (10 mL) and the aqueous phase washed with diethyl ether until the organic phase returned clear. The aqueous phase was concentrated *in vacuo* and purification via gel filtration (HW-40, NH_4OAc buffer) and subsequent HPLC purification afforded compound **7** (13.1 mg, 18.5 µmol, 9.3% (over 5 steps)) as the ammonium salt as a white foam. ^{1}H NMR (500 MHz, D_2O) δ 8.40 (s, 1H), 8.08 (s, 1H), 7.35 – 7.12 (m, 5H), 5.99 (d, *J* = 5.8 Hz, 1H), 5.26 (d, *J* = 5.1 Hz, 1H), 4.51 (d, *J* = 11.3 Hz, 1H), 4.45 – 4.37 (m, 2H), 4.31 – 4.17 (m, 4H), 4.14 (bs, 2H), 3.99 (td, *J* = 5.4, 1.7 Hz, 1H), 3.95 – 3.87 (m, 2H). ^{13}C NMR (126 MHz, D_2O) δ 155.3, 152.5, 148.9, 139.8, 136.7, 128.6, 128.3, 118.6, 90.3, 86.9, 84.8 (d, *J* = 7.8 Hz), 83.8 (d, *J* = 9.3 Hz), 78.3, 74.3, 72.9, 70.3, 68.7, 65.3 (d, *J* = 4.0 Hz), 65.2 (d, *J* = 4.2 Hz). ^{31}P NMR (202 MHz, D_2O) δ -11.3. HRMS (ESI) m/z: $[M+H]^+$ Calcd for $C_{22}H_{29}N_8O_{13}P_2$ 675.1324; Found 675.1322.

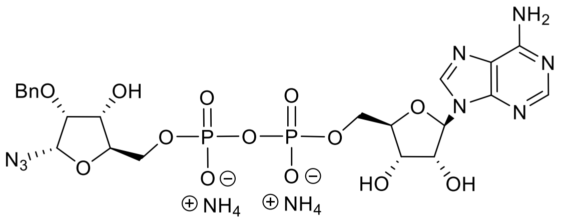

Scheme S5: Synthesis of Fluorinated ADPr

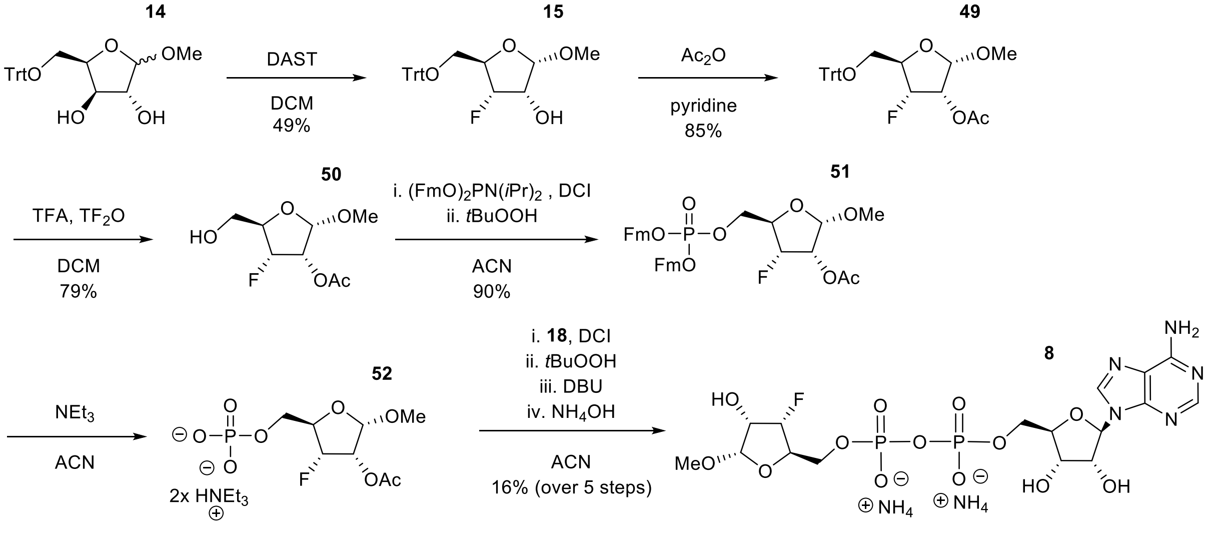

1-α-*O*-methyl-3-deoxy-3-fluoro-5-*O*-trityl-α-D-ribofuranoside (15) Trans vicinal D-lyxofuranoside 2,3-diol **14**

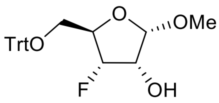

(1.9 g, 4.6 mmol, 1.0 eq. α/β = 13/7) was co-evaporated thrice with anhydrous pyridine, dissolved in anhydrous dichloromethane (12.0 mL, 0.17 M) and warmed to 28 °C. Diethylaminosulfur trifluoride (DAST) (2.7 mL, 20.7 mmol, 4.5 eq.) was added dropwise and the resulting solution was stirred for six hours, whereafter it was quenched by the careful addition of sat. aq. sodium bicarbonate solution (4 mL). The mixture was poured into a separatory funnel containing additional sat. aq. sodium bicarbonate solution (50 mL) and the aqueous layer was extracted thrice with dichloromethane (3 x 100 mL). The combined organic layers were dried over Na_2SO_4, filtered off and concentrated *in vacuo*. Purification by silica gel column chromatography (pentane/ethyl acetate, 99/1 → 7/3, v/v with 1% triethylamine) furnished title compound **15** (0.92 g, 2.25 mmol, 49%, 75% based on the α anomer) as a yellow oil. R_f 0.32 (ethyl acetate/pentane, 2/8, v/v). ^1H NMR (500 MHz, $CDCl_3$) δ 7.46 – 7.39 (m, 6H), 7.34 – 7.23 (m, 9H), 5.07 (d, 1H, J = 4.7 Hz), 4.81 (ddd, 1H, J= 55.0 Hz, J = 5.4, 1.1 Hz), 4.46 – 4.24 (m, 2H), 3.53 (s, 3H), 3.41 (dd, 1H, J = 10.3, 3.8 Hz), 3.15 (dd, 1H, J= 10, 3.1 Hz), 2.76 (d, 1H, J = 11.9 Hz). ^{13}C NMR (126 MHz, $CDCl_3$) δ 143.7, 128.7, 128.1, 127.4. 102.6, 91.4 (d, J = 183.6 Hz), 87.1, 82.2 (d, J = 23.9 Hz), 72.6 (d, J = 16.3 Hz), 63.4 (d, J= 10.1 Hz), 56.0. ^{19}F NMR (471 MHz, $CDCl_3$, proton-decoupled) δ -195.0. HRMS (ESI) m/z: $[M+Na]^+$ Calcd for $C_{25}H_{25}FO_4Na$ 431.1629; Found 431.1629.

1-α-*O*-methyl-2-*O*-acetyl-3-deoxy-3-fluoro-5-*O*-trityl-D-ribofuranoside (49) To a solution of compound **15**

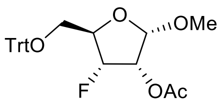

(0.29 g, 0.70 mmol, 1.0 eq.) in anhydrous pyridine/acetic anhydride (3/1, v/v, 3.5 mL, 0.20 M) was added a catalytic amount of DMAP (17 mg, 0.14 mmol, 0.20 eq.). The reaction was stirred until TLC analysis indicated full consumption of the starting material. The mixture was poured into a separatory funnel containing sat. aq. sodium bicarbonate solution and the aqueous phase was extracted thrice with dichloromethane (3 x 20 mL). The resulting organic layers were combined, dried over $MgSO_4$, filtered off and concentrated *in vacuo*. Purification by silica gel column chromatography (pentane/ethyl acetate, 99/1 → 7/3, v/v with 1% triethylamine) afforded title compound **49** (0.27 g, 0.59 mmol, 85%) as a yellow oil. R_f 0.65 (pentane/ethyl acetate, 9/1, v/v). ^1H NMR (300 MHz, $CDCl_3$): δ 7.50 – 7.43 (m, 6H), 7.33 – 7.20 (m, 9H), 5.21 (dt, 1H, J = 51.0 Hz, J = 4.2 Hz), 5.17 – 5.11 (m, 1H), 5.03 (dd, 1H, J = 3.9 Hz, J = 1.2 Hz), 4.36 (dq, 1H, J = 21.6 Hz, J = 4.5 Hz), 3.41 (s, 3H), 3.32 (dd, 1H, J = 10.2, 4.5 Hz), 3.21 (dd, 1H, J= 10.2, 4.8 Hz), 2.14 (s, 3H). ^{13}C NMR (75 MHz, $CDCl_3$) δ 170.0, 143.7, 128.8, 128.2, 127.2, 106.1 (d, J = 2.3 Hz), 90.5 (d, J = 190.2 Hz), 87.0, 81.2 (d, J = 24.1 Hz), 75.5 (d, J = 13.6 Hz), 63.8 (d, J = 6.0 Hz), 56.1, 20.7. HRMS (ESI) m/z: $[M+Na]^+$ Calcd for $C_{27}H_{27}FO_5Na$ 473.1735; Found 473.1732.

1-α-*O*-methyl-2-*O*-acetyl-3-deoxy-3-fluoro-D-ribofuranoside (50) Compound **49** (0.27 g, 0.61 mmol, 1.0 eq.)

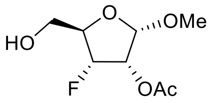

was co-evaporated thrice with anhydrous toluene, dissolved in anhydrous dichloromethane (5.1 mL, 0.12 M) and cooled to 0 °C using an ice bath. To this was added trifluoroacetic acid (0.19 mL, 2.4 mmol, 4.0 eq.) and trifluoroacetic anhydride (0.24 mL, 1.83 mmol, 3.0 eq.) successively, and cooling was removed. The solution was allowed to warm up to room temperature. After TLC analysis indicated complete consumption of the starting material, the reaction mixture was cooled to 0 °C, quenched by the addition of triethylamine (2 mL) and methanol (1 mL) and stirred until gas formation ceased. The quenched mixture was poured into a separatory funnel containing water (10 mL) and the

aqueous phase was extracted thrice with dichloromethane (3 x 10 mL). The resulting organic layers were combined, dried over Na_2SO_4, filtered and concentrated under reduced pressure. Purification by silica gel column chromatography (pentane/ethyl acetate, 99/1 → 1/1, v/v) furnished title compound **50** (100 mg, 0.48 mmol, 79%) as a yellow oil. R_f 0.33 (pentane/ethyl acetate, 7/3, v/v). ^{1}H NMR (500 MHz, $CDCl_3$) δ 5.16 (ddd, *J* = 52.8, 4.7, 3.3 Hz, 1H), 5.02 – 4.97 (m, 2H), 4.36-4.28 (ddd, 1H, *J* = 21.9 Hz, *J* = 6.5, 3.0 Hz), 3.75 (ddd, 1H, *J* = 12.3, 3.0, *J* = 1.2 Hz), 3.61 (dd, 1H, *J* = 12.5 Hz, 3.6 Hz), 3.40 (s, 3H), 2.48 (bs, 1H), 2.08 (s, 3H). ^{13}C NMR (126 MHz, $CDCl_3$) δ 170.0, 106.8, 91.0 (d, *J* = 108.0 Hz), 83.9 (d, *J* = 13.5 Hz), 76.4 (d, *J* = 9.0 Hz), 62.5, 56.5, 20.7. ^{19}F NMR (376 MHz, $CDCl_3$) δ -207.02 (ddd, *J* = 52.8, 21.7, 11.0 Hz). HRMS (ESI) m/z: $[M+Na]^+$ Calcd for $C_8H_{13}FO_5Na$ 231.0639; Found 231.0639.

1-α-*O*-methyl-2-*O*-acetyl-3-deoxy-3-fluoro-5-*O*-(di(9*H*-fluoren-9-yl))-D-ribofuranoside (51) Compound **50** (20 mg, 0.10 mmol, 1.0 eq.) and $(FmO)_2PN(iPr)_2$ (80 mg, 0.15 mmol, 1.5 eq.) were co-evaportaed thrice with anhydrous acetonitrile, suspended in anhydrous acetonitrile (2 mL, 0.05 M) and stirred until all was dissolved. Phosphitylation was initiated by the addition of DCI (21 mg, 0.17 mmol, 1.7 eq.) and the solution was stirred until ^{31}P-NMR indicated complete conversion into the corresponding phosphite triester intermediate ($δ_P$ 140.9). Hereafter, oxidation was effected by the addition of *t*BuOOH (5.5 M in nonane) (50 µL, 0.28 mmol, 2.8 eq.) and stirring commenced until ^{31}P-NMR analysis inferred complete oxidation into the P^V phosphate triester ($δ_P$ -1.86). Subsequently, the reaction mixture was quenched by the addition of water, further diluted with water (10 mL), extracted thrice with dichloromethane (3 x 10 mL) and the resulting organic layers were combined, dried over Na_2SO_4, filtered off and concentrated *in vacuo*. Subjection of the crude to flash column chromatography (dichloromethane/acetone, 1/0 → 19/1, v/v) furnished title compound **51** (50 mg, 90.0 µmol, 90%) as a white waxy oil. R_f 0.5 (pentane/ethyl acetate, 6/4, v/v). ^{1}H NMR (300 MHz, $CDCl_3$) δ 7.72 – 7.63 (m, 4H), 7.52 – 7.43 (m, 4H), 7.36, 7.20 (m, 8H), 5.08 (dt, 1H, *J* = 53.1 Hz, *J* = 4.7 Hz), 5.00 (ddd, 1H, *J* = 6.8, 4.9, 2.1 Hz), 4.93 – 4.84 (m, 1H), 4.31 – 4.20 (m, 5H) 4.10 (t, 2H, *J* = 6.3 Hz), 3.86 (dd, 2H, *J* = 6.5, 4.9 Hz), 3.20 (s, 3H), 2.10 (s, 3H). ^{13}C NMR (75 MHz, $CDCl_3$) δ 169.7, 142.83, 142.81, 141.39, 141.37, 127.97, 127.95, 127.2, 124.94, 124.93, 120.07, 120.05, 106.0 (d, *J* = 3.0 Hz), 89.5 (d, *J* = 201.7 Hz), 79.5 (d, *J* = 25.3 Hz), 74.7 (d, *J* = 14.3 Hz), 69.49 (d, *J* = 6.0 Hz), 69.46 (d, *J* = 6.0 Hz), 66.7 (appears as t, *J* = 5.7 Hz), 55.7, 47.8, 47.7, 20.5. ^{31}P NMR (122 MHz, $CDCl_3$) δ -1.8. HRMS (ESI) m/z: $[M+H]^+$ Calcd for $C_{36}H_{35}FO_8P$ 645.2048; Found 645.2046.

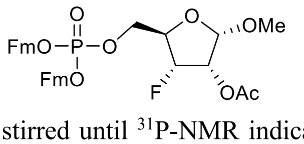

1-α-*O*-methyl-2-*O*-acetyl-3-deoxy-3-fluoro-5-*O*-phosphate-D-ribofuranoside (52) Compound **51** (0.25 g, 0.40 mmol, 1.0 eq.) was co-evaporated with anhydrous acetonitrile thrice and redissolved in anhydrous acetonitrile (8.0 mL, 0.050 M). To this, triethylamine (2.8 mL, 20 mmol, 50 eq.) was added. The reaction was monitored through LC-MS analysis and full deprotection was detected after stirring overnight, whereafter the volatiles were removed under reduced pressure. Subsequent co-evaporations with anhydrous pyridine and a final co-evaporation with anhydrous acetonitrile. The resulting crude product was used directly in the next reaction without any further purification.

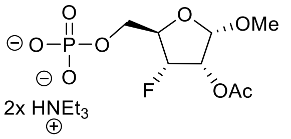

1”-α-*O*-methyl-3”-deoxy-3”-fluoro ADPr (8) Compound **18** (0.36 g, 0.44 mmol, 1.1 eq.) was co-evaporated with anhydrous acetonitrile (3 x 5 mL), dissolved in anhydrous acetonitrile (8.0 mL, 0.050 M) and added to a stirring suspension of crude compound **52** (0.11 g, 0.40 mmol, 1.0 eq.). Phosphitylation was initiated by the addition of DCI (0.12 grams, 1.0 mmol, 2.5 eq.), whereupon after ^{31}P-NMR spectroscopy indicated complete formation of the phosphite-phosphate P^{III}-P^V intermediate ($δ_P$ 129.4, 127.6 (P^{III}), -10.2 (P^V)), oxidation was facilitated by the addition of *t*BuOOH (5.5 M in nonane) (0.18 mL, 1.0 mmol, 2.5 eq.). Stirring commenced until ^{31}P-NMR analysis indicated complete oxidation into the corresponding protected P^V-P^V pyrophosphate linkage ($δ_P$ -11.09 to -12.63), whereafter DBU (0.30 mL, 2.0 mmol, 5.0 eq.) was added. After one hour, global deprotection was mediated by treatment with an aqueous ammonium hydroxide solution (28 wt. % NH_4OH water, 8.0 mL, 50 eq.) and the resulting mixture was left to stir overnight and monitored using LCMS. Upon completion, excess ammonia was purged by stirring under vacuum and the resulting residue was subjected to an extractive work-up by partitioning in a mixture of water (10 mL) and diethyl ether (10 mL). The organic layer was back-extracted thrice with water (3 x 10 mL) and the resulting aqueous layers were combined and concentrated *in vacuo*. The crude material was purified using size-exclusion chromatography (HW-40, NH_4OAc buffer) and HPLC. The colourless oil obtained was co-evaporated thrice with a 1/1 (v/v) mixture of acetonitrile/water (3 x 2 mL) to remove excess NH_4OAc and subsequently lyophilized to furnish title compound **8** (37.0 mg, 0.064 mmol, 16%) as the ammonia salt as a white powder. ^{1}H NMR (500 MHz,

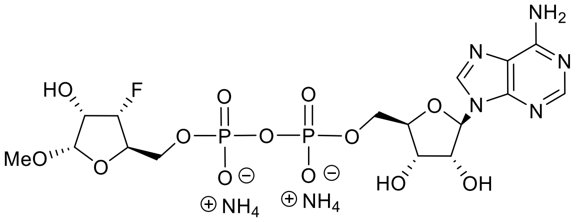

D_2O) δ 8.48 (s, 1H), 8.21 (s, 1H), 6.11 (d, 1H, *J* = 5.8 Hz), 5.03 (ddd, 1H, *J* = 53.4 Hz, *J* = 4.6, 3.0 Hz), 4.91 (dd, 1H, *J* = 4.0 Hz, *J* = 1.4 Hz), 4.75 (app. t, 1H, *J* = 5.5 Hz), 4.51 (dd, 1H, *J* = 5.2, 3.7 Hz), 4.39 – 4.35 (m, 1H), 4.34 (dq, 1H, *J* = 23.5 Hz, *J* = 4.4 Hz), 4.21 (bs, 2H), 4.14 (dt, 1H, *J* = 15.2 Hz, *J* = 4.3 Hz), 3.98 (q, 2H, *J* = 4.3 Hz, *J* = 3.7 Hz), 3.38 (s, 3H). ^{13}C NMR (126 MHz, D_2O) δ 155.6, 152.8, 149.1, 139.3 (signal taken from HSQC), 119.6, 107.6, 92.0 (d, *J* = 183.6 Hz), 87.0, 83.9, 80.3 (d, *J* = 21.4 Hz), 74.3, 73.2 (d, *J* = 15.1 Hz), 70.4, 65.5 (C-P coupling not resolved), 65.2 (C-P coupling not resolved), 56.1. ^{31}P NMR (202 MHz, D_2O) δ -10.73 (d, *J* = 22.3 Hz). ^{31}P{^{1}H} NMR (202 MHz, D_2O) δ -10.7 (br. s). ^{19}F NMR (471 MHz, D_2O, proton and carbon-decoupled) δ -205.6. HRMS (ESI) m/z: $[M+H]^+$ Calcd for $C_{16}H_{25}FN_5O_{13}P_2$ 576.0903; Found 576.0901.

Scheme S6: Synthesis of 1”-α-*O*-methyl-D-erythro-pentofuran-3”-uloside *O*-methyl oxime ADPr

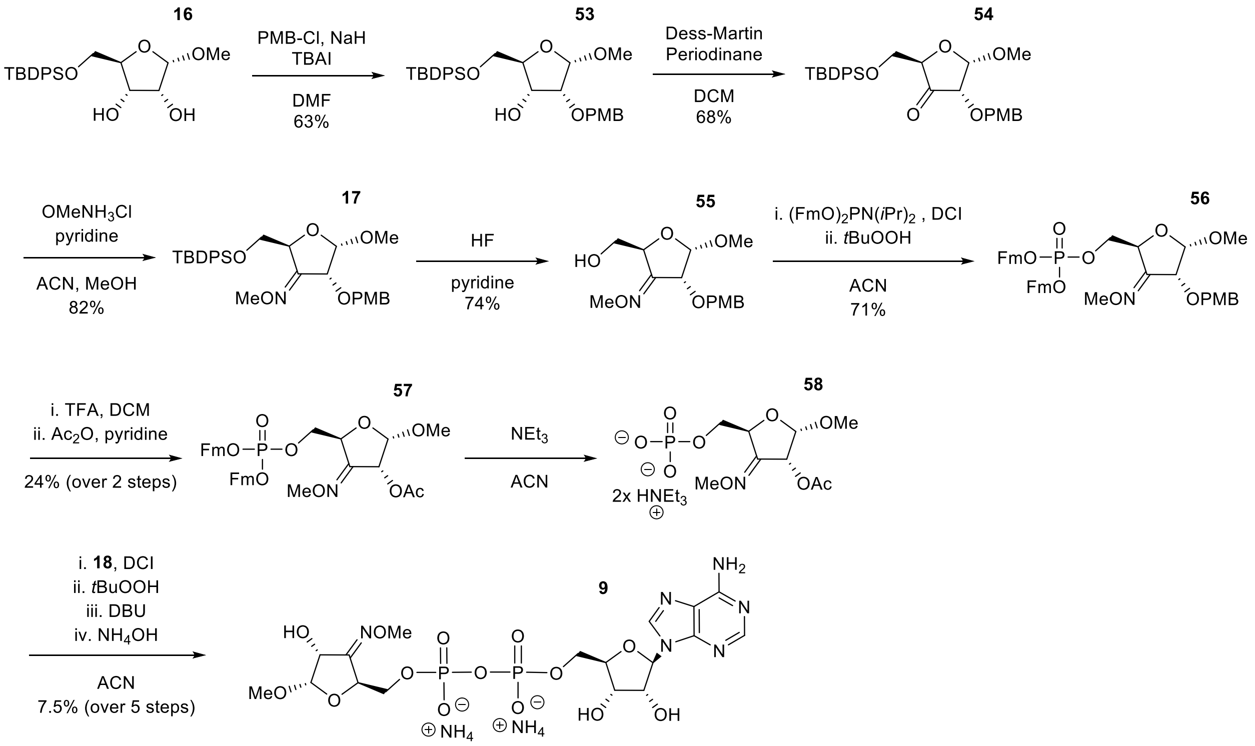

1-α-*O*-methyl-5-*O*-(*tert*-butyl-di-phenylsilyl)-D-ribofuranoside (16) Compound **31** (3.89 grams, 23.7 mmol) was dissolved in anhydrous DMF (118 mL, 0.20 M) and flushed with nitrogen over a period of 30 minutes. While stirring, imidazole (3.2 grams, 47 mmol, 2.0 eq.), DMAP (0.29 grams, 2.4 mmol, 0.1 eq.) and TBDPS-Cl (7.2 grams, 26 mmol, 1.1 eq.) were added. The reaction mixture was then allowed to stir for 24 hours at room temperature at which point TLC analysis indicated full consumption of the starting material. The reaction was then quenched with water and diluted with diethyl ether. The organic phase was washed three times with water, once with brine and was dried over $MgSO_4$, filtered off concentrated *in vacuo*. Purification by column chromatography (ethyl acetate/pentane, 1/9 → 3/7, v/v) afforded compound **16** (6.97 grams, 17.3 mmol, 73%) as clear colourless oil. TLC: R_f 0.4 (ethyl acetate/pentane, 1/1, v/v). ^1H NMR (400 MHz, $CDCl_3$) δ 7.70 – 7.62 (m, 4H), 7.47 – 7.34 (m, 6H), 4.94 (d, *J* = 4.4 Hz, 1H), 4.22 (ddd, *J* = 9.5, 5.9, 4.4 Hz, 1H), 4.18 – 4.06 (m, 2H), 3.77 (d, *J* = 3.2 Hz, 2H), 3.47 (s, 3H), 3.07 (d, *J* = 9.5 Hz, 1H), 2.76 (d, *J* = 8.0 Hz, 1H), 1.05 (s, 9H). ^{13}C NMR (101 MHz, $CDCl_3$) δ 135.7, 135.6, 133.2, 133.0, 129.9, 129.8, 127.9, 127.8, 103.1, 85.8, 72.3, 71.4, 64.2, 55.7, 26.9, 19.3. HRMS (ESI) m/z: $[M+NH_4]^+$ Calcd for $C_{22}H_{30}O_5SiNH_4$ 420.2206; Found 420.2201.

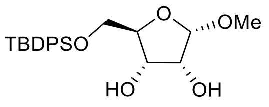

1-α-*O*-methyl-2-*O*-(*para*-methoxybenzyl)-5-*O*-(*tert*-butyl-diphenylsilyl)-D-ribofuranoside (53) Compound **16** (4.65 grams, 11.5 mmol) was co-evaporated with anhydrous toluene for three times after which it was dissolved in anhydrous DMF (68 mL, 0.17 M) and flushed with nitrogen at 0 °C. To the reaction mixture, tetra-n-butylammonium iodide (TBAI) (0.43 grams, 1.2 mmol, 0.10 eq.) and 4-methoxybenzyl chloride (PMB-Cl) (2.0 grams, 13 mmol, 1.1 eq.) were added and the resulting reaction mixture was stirred for 10 minutes. Subsequently, sodium hydride (60% w/w dispersion in mineral oil) was slowly added (0.31 grams, 13 mmol, 1.1 eq.) and the resulting grey suspension was stirred at room temperature over a period of 20 hours. At this point TLC analysis indicated absence of the starting material and the reaction was quenched with ice and diluted with diethyl ether. The organic phase was washed three times with water and was dried over $MgSO_4$, filtered off concentrated *in vacuo*. Purification by column chromatography (ethyl acetate/pentane, 1/9 → 3/7, v/v) gave a 3/2 (mol/mol) inseparable mixture of compound **53** and anisyl alcohol respectively (3.56 grams, 6.85 mmol, 63%) as cloudy light-yellow oil.z TLC: R_f 0.5 (ethyl acetate/pentane, 1/1, v/v). ^1H NMR (400 MHz, $CDCl_3$) δ 7.72 – 7.59 (m, 4H), 7.47 – 7.34 (m, 6H), 7.33 – 7.28 (m, 2H), 6.93 – 6.78 (m, 2H), 4.89 (d, *J* = 4.1 Hz, 1H), 4.70 (d, *J* = 11.8 Hz, 1H), 4.63 – 4.53 (m, 1H), 4.25 – 4.16 (m, 2H), 3.98 (dd, *J* = 5.8, 4.1 Hz, 1H), 3.79 (s, 3H), 3.72 (qd, *J* = 11.3, 3.0 Hz, 2H), 3.43 (s, 3H), 3.01 (d, *J* = 8.8 Hz, 1H), 0.98 (s, 9H). ^{13}C NMR (101 MHz, $CDCl_3$) δ 159.6, 135.8, 135.7, 135.7, 133.3, 133.1, 130.0, 129.91, 129.86, 129.7, 129.5, 128.8, 127.9, 127.9, 127.83, 127.79, 114.1, 113.9,

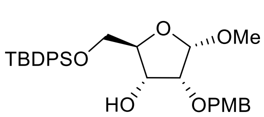

113.8, 102.7, 87.0, 78.1, 72.2, 70.1, 65.2, 64.3, 55.4, 55.4, 55.2, 26.9, 19.3. HRMS (ESI) m/z: $[M+NH_4]^+$ Calcd for $C_{30}H_{38}O_6SiNH_4$ 540.2781; Found 540.2776.

1-α-*O*-methyl-2-*O*-(*para*-methoxybenzyl)-5-*O*-(*tert*-butyl-diphenylsilyl)-D-erythro-pentofuran-3-uloside (54) Compound **53** (2.22 grams, 4.25 mmol) was dissolved in dichloromethane (47 mL, 0.090 M) and Dess-Martin Periodinane (DMP) (3.1 grams, 7.2 mmol, 1.7 eq.) was added to the solution. Subsequently, the resulting white suspension was stirred over a period of 17 hours at room temperature. At this point TLC analysis indicated full conversion of the starting material and the reaction was diluted with sat. aq. sodium bicarbonate solution and sat.aq. sodium thiosulphate solution. The product was extracted with dichloromethane and the combined organic phases were dried over $MgSO_4$ filtered off and concentrated *in vacuo*. Purification by column chromatography (diethyl ether/pentane, 0/1 → 1/4, v/v) afforded compound **54** (1.50 grams, 2.88 mmol, 68%) as clear yellow oil. TLC: R_f 0.4 (diethyl ether/pentane, 2/3, v/v). ^1H NMR (400 MHz, $CDCl_3$) δ 7.61-7.57 (m, 4H), 7.46 – 7.35 (m, 6H), 7.32 (d, *J* = 8.6 Hz, 2H), 6.89 – 6.83 (m, 2H), 5.04 (d, *J* = 4.7 Hz, 1H), 4.84 (d, *J* = 12.0 Hz, 1H), 4.75 (d, *J* = 12.1 Hz, 1H), 4.24 (dd, *J* = 4.7, 0.9 Hz, 1H), 3.98 (t, *J* = 2.2 Hz, 1H), 3.95 – 3.88 (m, 1H), 3.82 (dd, *J* = 11.4, 2.1 Hz, 1H), 3.78 (s, 3H), 3.45 (s, 3H), 0.92 (s, 9H). ^{13}C NMR (101 MHz, $CDCl_3$) δ 210.0, 159.8, 135.7, 135.7, 132.8, 132.6, 130.3, 130.0, 129.9, 128.8, 127.9, 127.9, 127.9, 114.1, 99.8, 79.8, 77.5, 72.6, 62.5, 55.4, 29.8, 26.8, 19.3. HRMS (ESI) m/z: $[M+NH_4]^+$ Calcd for $C_{30}H_{36}O_6SiNH_4$ 538.2625; Found 538.2619.

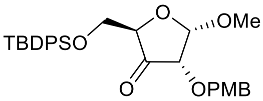

1-α-*O*-methyl-2-*O*-(*para*-methoxybenzyl)-5-*O*-(*tert*-butyl-diphenylsilyl)-D-erythro-pentofuran-3-uloside *O*-methyl oxime (17) Compound **54** (0.52 grams, 1.0 mmol) was dissolved in a solvent system of methanol (2.9 mL), pyridine (0.4 mL) and acetonitrile (0.6 mL) (0.26 M). While stirring, $MeONH_3Cl$ (0.17 grams, 2.0 mmol, 2.0 eq.) was added at room temperature and the resulting solution was allowed to stir over a period of 2 hours. Upon completion, the reaction was quenched with water and diluted with ethyl acetate. The organic phase was washed with brine and dried over $MgSO_4$, filtered off and concentrated *in vacuo*. Purification by column chromatography (ethyl acetate/pentane, 0/1 → 1/4, v/v) gave compound **17** (0.45 grams, 0.82 mmol, 82%) as clear, colourless oil. TLC: R_f 0.5 (ethyl acetate/pentane, 1/4, v/v). ^1H NMR (400 MHz, $CDCl_3$) δ 7.71 – 7.58 (m, 4H), 7.47 – 7.28 (m, 8H), 6.90 – 6.82 (m, 2H), 4.86 (d, *J* = 12.1 Hz, 1H), 4.81 – 4.67 (m, 3H), 4.57 (dd, *J* = 4.4, 1.8 Hz, 1H), 4.10 (dd, *J* = 11.0, 2.5 Hz, 1H), 3.91 (s, 3H), 3.78 (s, 3H), 3.76 (dd, *J* = 11.1, 1.9 Hz, 1H), 3.37 (s, 3H), 0.96 (s, 9H). ^{13}C NMR (101 MHz, $CDCl_3$) δ 159.6, 157.4, 135.80, 135.75, 135.7, 133.3, 133.2, 130.3, 129.9, 129.79, 129.76, 129.4, 127.8, 127.7, 113.9, 113.8, 100.3, 77.1, 75.6, 72.8, 72.6, 62.4, 62.3, 55.4, 54.9, 26.8, 19.3. HRMS (ESI) m/z: $[M+Na]^+$ Calcd for $C_{31}H_{39}NO_6SiNa$ 572.2444; Found 572.2439.

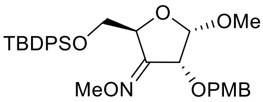

1-α-*O*-methyl-2-*O*-(*para*-methoxybenzyl)-D-erythro-pentofuran-3-uloside *O*-methyl oxime (55) Compound **17** (0.11 grams, 0.20 mmol) was co-evaporated with anhydrous toluene thrice and dissolved in pyridine (2.0 mL, 0.1 M). The resulting solution was brought under a nitrogen atmosphere and while on ice, HF-pyridine (70% w/w) (0.20 mL, 6.9 mmol, 34 eq.) was carefully added. The resulting light-yellow solution was allowed to stir at room temperature for two hours. Upon completion, the reaction was quenched with sat. aq. sodium bicarbonate solution and the product was extracted with dichloromethane. The combined organic phases were dried over $MgSO_4$, filtered off and concentrated *in vacuo*. Purification by column (ethyl acetate/pentane, 2/3 → 1/1, v/v) gave compound **55** (46 mg, 0.15 mmol, 74%) as clear colourless oil. TLC: R_f 0.5 (ethyl acetate/pentane, 7/3, v/v). ^1H NMR (400 MHz, $CDCl_3$) δ 7.34 (d, *J* = 8.6 Hz, 2H), 6.92 – 6.84 (m, 2H), 4.85 (d, *J* = 12.0 Hz, 1H), 4.76 (td, *J* = 3.3, 1.9 Hz, 1H), 4.73 – 4.64 (m, 2H), 4.47 – 4.38 (m, 1H), 4.03 – 3.91 (m, 4H), 3.83 – 3.79 (m, 4H), 3.36 (s, 3H). ^{13}C NMR (101 MHz, $CDCl_3$) δ 159.7, 157.1, 130.2, 130.0, 129.3, 113.9, 113.8, 102.0, 100.4, 77.0, 75.7, 75.6, 72.9, 72.7, 62.7, 61.4, 55.4, 55.0. HRMS (ESI) m/z: $[M+Na]^+$ Calcd for $C_{15}H_{21}NO_6Na$ 334.1267; Found 334.1261.

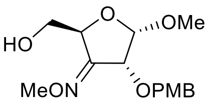

1-α-*O*-methyl-2-*O*-(*para*-methoxybenzyl)-5-*O*-(di(9*H*-fluoren-9-yl))-phosphoryl-D-erythro-pentofuran-3-uloside *O*-methyl oxime (56) Compound **55** (46 mg, 0.15 mmol) was co-evaporated three times with anhydrous toluene and dissolved in anhydrous acetonitrile (1.0 mL, 0.15 M). While stirring, DCI (52 mg, 0.44 mmol, 3.0 eq.) was added and the resulting white suspension was stirred vigorously while flushing with nitrogen for ten minutes. $(FmO)_2PN(iPr)_2$ (0.12 grams, 0.22 mmol, 1.5 eq.) was co-evaporated thrice with anhydrous toluene and dissolved in anhydrous acetonitrile (0.7 mL, 0.31 M). This solution was added dropwise to the stirring suspension containing compound **55**. After final addition, the mixture was allowed to stir at room temperature over a period of 90 minutes. At this point TLC analysis indicated full conversion of the starting material and *t*BuOOH (5.5 M in decanes) (67 mg, 0.74 mmol, 5.0 eq.) was added and the progression of the reaction was followed by ^{31}P NMR. When this indicated full conversion, the reaction was quenched with water and the product was extracted using dichloromethane. The combined organic phases were dried over $MgSO_4$, filtered off and concentrated *in*

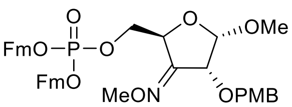

vacuo. Purification by column chromatography (acetone/pentane, 0/1 → 1/4, v/v) gave compound **56** (78 mg, 0.11 mmol, 71%) as light-yellow oil. TLC: R_f 0.4 (acetone/pentane, 3/7, v/v). ^{1}H NMR (400 MHz, $CDCl_3$) δ 7.79 – 7.69 (m, 4H), 7.57 – 7.46 (m, 4H), 7.45 – 7.32 (m, 4H), 7.31 – 7.22 (m, 4H), 7.22 – 7.16 (m, 2H), 6.78 – 6.73 (m, 2H), 4.78 – 4.70 (m, 2H), 4.53 (d, *J* = 4.3 Hz, 1H), 4.45 (d, *J* = 11.8 Hz, 1H), 4.35 – 4.07 (m, 9H), 3.83 (d, *J* = 1.7 Hz, 3H), 3.70 (s, 3H), 3.26 (s, 3H). ^{13}C NMR (101 MHz, $CDCl_3$) δ 159.6, 155.5, 143.2, 143.1, 143.0, 141.5, 141.43, 141.40, 130.1, 129.9, 129.2, 128.06, 128.04, 127.99, 127.91, 127.88, 127.3, 127.24, 127.20, 127.17, 125.31, 125.28, 125.25, 125.1, 125.0, 120.14, 120.11, 120.08, 120.0, 113.8, 100.3, 77.0, 73.1 (d, *J* = 8.2 Hz), 72.8, 69.5 (d, *J* = 6.0 Hz), 65.6 (d, *J* = 5.4 Hz), 62.7, 55.3, 55.1, 53.9, 48.0 (d, *J* = 2.5 Hz), 47.9 (d, *J* = 2.6 Hz), 29.8, 29.4. HRMS (ESI) m/z: $[M+NH_4]^+$ Calcd for $C_{43}H_{42}NO_9PNH_4$ 765.2941; Found 765.2935.

1-α-*O*-methyl-2-*O*-acetyl-5-*O*-(di(9*H*-fluoren-9-yl))-phosphoryl-D-erythro-pentofuran-3-uloside *O*-methyl oxime (57) Compound **56** (75 mg, 0.10 mmol) was dissolved in anhydrous dichloromethane (1.8 mL, 0.055 M) and cooled on ice. The mixture was brought under a nitrogen atmosphere and while stirring, triethyl silane hydride (TES-H) (0.16 mL, 1.0 mmol, 10 eq.) and TFA (0.2 mL, 10% v/v) were added. Subsequently, the light-yellow solution was allowed to stir for 4.5 hours at room temperature. Upon completion, the reaction was quenched with pyridine until a neutral pH was obtained. Subsequently, the solution was carefully concentrated *in vacuo*. Purification by flash column chromatography (acetone/pentane, 1/4 → 1/1, v/v) gave the corresponding secondary alcohol which was co-evaporated three times with pyridine and was dissolved in pyridine (0.10 mL, 1.0 M). The mixture was cooled on ice and acetic anhydride (14 µL, 0.15 mmol, 3.0 eq.) was added. The resulting colourless solution was stirred at room temperature for three hours after which full consumption of the intermediate was observed via TLC analysis. The reaction was quenched with water and the product was extracted using dichloromethane. The combined organic phases were dried over $MgSO_4$, filtered off and concentrated *in vacuo*. Purification via silica column chromatography (acetone/pentane, 1/4 → 2/3, v/v) gave compound **57** (16 mg, 0.024 mmol, 24%) as clear yellow oil. TLC: R_f 0.5 (acetone/pentane, 2/3, v/v). ^{1}H NMR (400 MHz, $CDCl_3$) δ 7.78 – 7.66 (m, 4H), 7.58 – 7.47 (m, 4H), 7.43 – 7.32 (m, 4H), 7.31 – 7.22 (m, 4H), 5.35 (dd, *J* = 4.5, 2.0 Hz, 1H), 5.14 (d, *J* = 4.5 Hz, 1H), 4.81 (p, *J* = 3.9, 2.2, 2.1 Hz, 1H), 4.39 – 4.19 (m, 6H), 4.16 (t, *J* = 6.9 Hz, 2H), 3.82 (s, 3H), 3.35 (s, 3H), 2.18 (s, 3H). ^{13}C NMR (101 MHz, $CDCl_3$) δ 170.0, 152.9, 143.3, 143.2, 141.5, 128.1, 128.0, 127.3, 127.3, 125.4, 125.31, 125.27, 120.2, 120.1, 99.8, 73.3 (d, *J* = 8.5 Hz), 71.8, 69.5 (t, *J* = 6.2 Hz), 65.4 (d, *J* = 5.5 Hz), 62.9, 55.4, 48.03 (no C-P coupling observed), 47.96 (no C-P coupling observed), 20.8. HRMS (ESI) m/z: $[M+NH_4]^+$ Calcd for $C_{37}H_{36}NO_9PNH_4$ 687.2471; Found 687.2466.

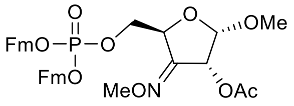

1”-α-*O*-methyl-D-erythro-pentofuran-3”-uloside *O*-methyl oxime ADPr (9) Compound **57** (16 mg, 0.024 mmol) was dissolved in a mix of triethylamine (0.17 mL, 1.2 mmol, 50 eq.) and anhydrous acetonitrile (0.5 mL, 0.05 M) and the resulting light-yellow solution was allowed to stir at room temperature for 60 hours. At this point TLC analysis indicated absence of the starting material or its mono-deprotected counterpart and the solution was directly concentrated *in vacuo* and subsequently co-evaporated thrice with pyridine. Then, to it DCI (7.1 mg, 0.060 mmol, 2.5 eq.) was added and the mixture was again co-evaporated thrice but this time with anhydrous acetonitrile. The resulting white mixture was suspended in anhydrous acetonitrile (0.6 mL, 0.04 M). In another flask, compound **18** (21 mg, 0.029 mmol, 1.2 eq) was co-evaporated thrice with anhydrous toluene and dissolved in anhydrous acetonitrile (0.3 mL, 0.1 M), before it was added dropwise to the suspension containing the phosphate **58**. The mixture was brought under an atmosphere of argon gas and stirred vigorously for one hour at room temperature. At this point *t*BuOOH (5.5 M in decanes) (22 µL, 0.12 mmol, 5.1 eq.) was added dropwise to the suspension which was then allowed to stir for 90 minutes at room temperature. At this point ^{31}P NMR showed full conversion of all phosphite species into their corresponding oxidated counterparts and to the reaction mixture was added DBU (18 µL, 0.12 mmol, 5.1 eq.). The resulting suspension was allowed to stir at room temperature for half an hour after which ammonium hydroxide (28% NH_3 w/w, 0.4 mL) was added and the resulting solution was allowed to stir at room temperature for six days when LCMS analysis indicated full deprotection of the construct. The reaction mixture was diluted with water and the aqueous phases were washed thrice with ethyl acetate after which the aqueous phase was concentrated *in vacuo.* Subsequent purification by size exclusion chromatography (HW-40, NH_4OAc buffer) and HPLC afforded compound **9** (1.1 mg, 1.8 µmol, 7.5% (over 5 steps)) as the ammonium salt as a white foam. ^{1}H NMR (500 MHz, D_2O) δ 8.56 – 8.45 (m, 1H), 8.29 – 8.18 (m, 1H), 6.15 – 6.11 (m, 1H), 5.01 (d, *J* = 4.6 Hz, 1H), 4.89 – 4.84 (m, 1H), 4.82 (dd, *J* = 4.7, 1.8 Hz, 1H), 4.78 – 4.75 (m, 1H), 4.53 (dd, *J* = 5.2, 3.6 Hz, 1H), 4.39 (q, *J* = 3.4 Hz, 1H), 4.27 – 4.19 (m, 3H), 4.13 (dq, *J* = 11.3, 2.5 Hz, 1H), 3.84 (s, 3H), 3.38 (s, 3H). ^{13}C NMR (126 MHz, D_2O) δ 158.6, 155.4, 152.6, 149.0,139.9, 118.6, 101.0, 86.9, 83.8 (C-P coupling unresolved), 74.2, 73.8 (ap. t, *J* = 4.4 Hz), 70.5, 70.3, 65.1 (C-P coupling

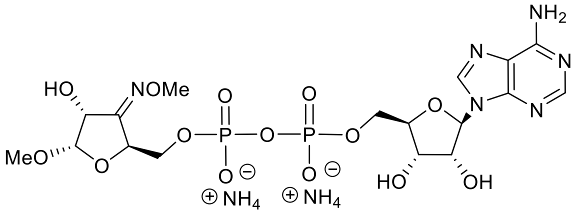

unresolved), 63.9 (C-P coupling unresolved), 62.1, 54.8. ^{31}P NMR (202 MHz, D_2O) δ -11.4. HRMS (ESI) m/z: $[M+H]^+$ Calcd for $C_{17}H_{27}N_6O_{14}P_2$ 601.1055; Found 601.1060.

Scheme S7: Synthesis of acid-labile P(V) donors

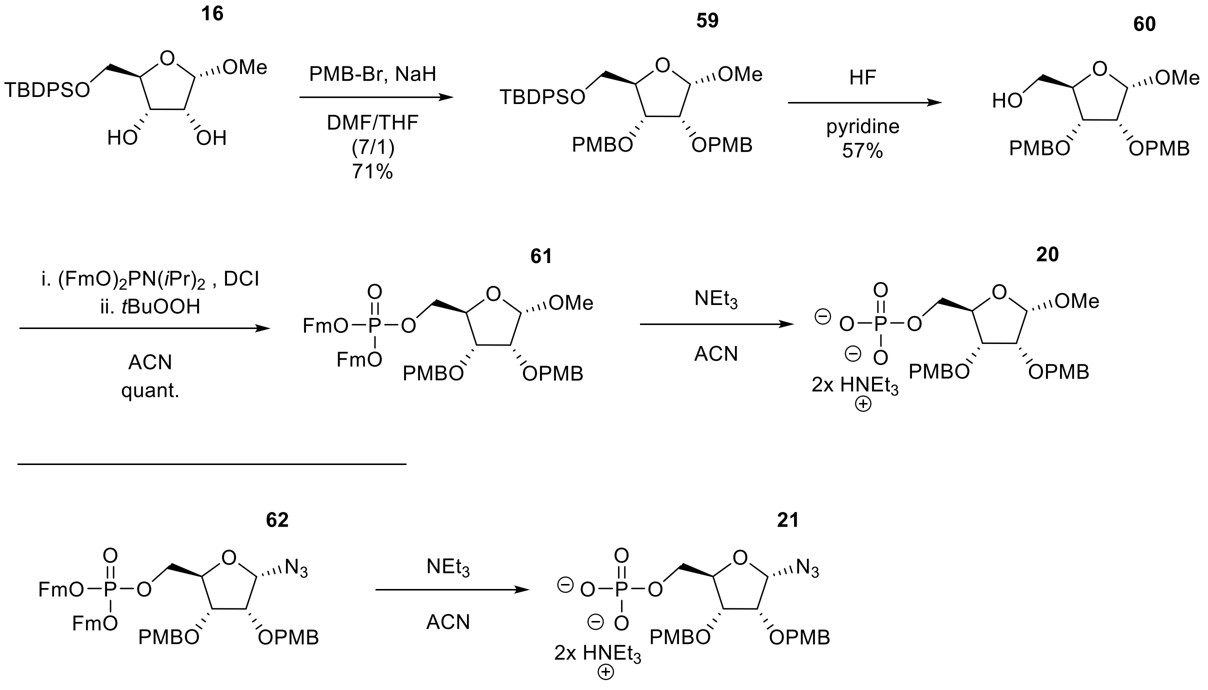

1-α-*O*-methyl-2,3-*O*-(*para*-methoxybenzyl)-5-*O*-(*tert*-butyl-diphenylsilyl)-D-ribofuranoside (59) Compound

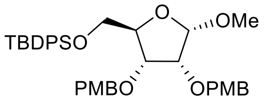

16 (90 mg, 0.22 mmol) was co-evaporated with dry distilled toluene thrice to remove residual water content after which it was brought under a nitrogen atmosphere. The starting material was then dissolved in a solvent system consisting of a 1/7 (v/v) mixture of tetrahydrofuran/DMF respectively (1.1 mL, 0.20 M) and to the resulting solution was added *para*-methoxybenzyl bromide (PMB-Br) (0.13 mL, 0.90 mmol, 4.0 eq.) after which the mixture was cooled to 0 °C using an icebath. Then, sodium hydride (60% w/w in mineral oil) (27 mg, 0.66 mmol, 3.0 eq.) was added and the resulting whitish suspension was allowed to warm to room temperature while stirring vigorously. After two hours of stirring TLC analysis indicated full consumption of the starting material and the reaction was quenched by the addition of water and diluted with ethyl acetate after which the biphasic mixture was stirred for five minutes. The product was extracted using ethyl acetate and the combined organic phases were washed with brine. The organic phase was then dried over Na_2SO_4, filtered off and concentrated *in vacuo*. Purification by silica column chromatography (ethyl acetate/pentane, 1/9 → 2/3, v/v) gave compound **59** (101 mg, 0.156 mmol, 71%). R_f 0.35 (pentane/ethyl acetate, 7/3, v/v). ^1H NMR (400 MHz, $CDCl_3$) δ 7.70 – 7.56 (m, 4H), 7.48 – 7.35 (m, 6H), 7.31 – 7.22 (m, 4H), 6.89 – 6.83 (m, 4H), 4.87 (d, *J* = 4.4 Hz, 1H), 4.65 – 4.59 (m, 2H), 4.55 (dd, *J* = 12.2, 5.3 Hz, 2H), 4.17 (q, *J* = 3.1 Hz, 1H), 3.96 (dd, *J* = 6.6, 2.5 Hz, 1H), 3.86 – 3.77 (m, 7H), 3.62 (dd, *J* = 11.1, 3.6 Hz, 1H), 3.54 – 3.45 (m, 4H), 0.97 (s, 9H). ^{13}C NMR (101 MHz, $CDCl_3$) δ 159.4, 159.3, 135.74, 135.67, 133.4, 133.2, 130.5, 130.0, 130.0, 129.9, 129.8, 129.7, 128.0, 127.93, 127.89, 127.83, 127.79, 113.9, 113.8, 102.7, 83.9, 77.9, 74.6, 72.2, 72.1, 64.3, 55.7, 55.4, 26.9, 26.9, 19.3. HRMS (ESI) m/z: $[M+Na]^+$ Calcd for $C_{38}H_{46}O_7SiNa$ 665.2905; Found 665.2903.

1-α-*O*-methyl-2,3-*O*-(*para*-methoxybenzyl)-D-ribofuranoside (60) Compound **59** (189 mg, 0.294 mmol) was

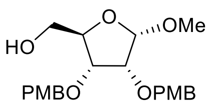

dissolved in pyridine (5.9 mL, 0.050 M) and transferred to a 15 mL falcon tube where it was brought under a nitrogen atmosphere and stirred vigorously. The solution was cooled to 0 °C using an icebath and to it was added HF-pyridine (70% HF, w/w) (0.3 mL) after which the mixture was removed from the icebath and it was allowed to warm to room temperature where it was stirred for four hours. At this point TLC analysis indicated full consumption of the starting material and the reaction mixture was poured out into an Erlenmeyer flask containing dichloromethane and sat. aq. sodium bicarbonate solution. The resulting bi-phasic system was vigorously stirred after which the product was extracted using dichloromethane (3x). The combined organic phases were dried over $MgSO_4$, filtered off and concentrated *in vacuo*. Purification by silica column chromatography (ethyl acetate/pentane, 3/7 → 3/1, v/v) gave compound **60** (68 mg, 0.17 mmol, 57%). R_f 0.45 (pentane/ethyl acetate, 1/4, v/v). ^1H NMR (400 MHz, $CDCl_3$) δ 7.28 (dd, *J* = 8.7, 2.6 Hz, 4H), 6.87 (dd, *J* = 8.7, 2.8 Hz, 4H), 4.83 (d, *J* = 4.2 Hz, 1H), 4.67 (d, *J* = 12.4 Hz, 1H), 4.60 (d, *J* = 11.9 Hz, 1H), 4.57 – 4.47 (m, 2H), 4.13 (q, *J* = 3.5 Hz, 1H), 3.84 – 3.77 (m, 7H), 3.71 – 3.61 (m, 2H), 3.45 (s, 3H), 3.38 (ddd, *J* = 11.9, 8.2, 3.8 Hz, 1H). ^{13}C NMR (101 MHz, $CDCl_3$) δ 159.5, 159.4, 130.4, 129.9, 129.8, 113.9,

102.9, 83.3, 77.8, 74.3, 72.4, 72.3, 62.9, 55.7, 55.4. HRMS (ESI) m/z: $[M+Na]^+$ Calcd for $C_{22}H_{28}O_7Na$ 427.1727; Found 427.1727.

1-α-*O*-methyl-2,3-*O*-(*para*-methoxybenzyl)-5-*O*-(di(9*H*-fluoren-9-yl))-phosphoryl--D-ribofuranoside (61)

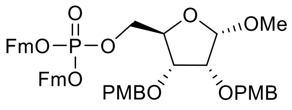

Compound **60** (68 mg, 0.17 mmol) was co-evaporated thrice with dry and distilled toluene, whereafter DCI (60 mg, 0.50 mmol, 3.0 eq.) was added and the mixture was co-evaporated with dry and distilled toluene once more. The resulting mixture was then dissolved in acetonitrile (2.4 mL, 0.071 M) and brought under a nitrogen atmosphere where it was stirred vigorously. In another flask, thrice co-evaporated (dry distilled toluene) $(FmO)_2PN(iPr)_2$ was dissolved in acetonitrile (0.32 M) and some of the stock solution (0.79 mL, 0.25 mmol, 1.5 eq.) is added to the initial flask dropwise. The reaction mixture was allowed to stir at room temperature for 100 minutes, after which TLC analysis indicated full consumption of the starting material. At this point to the mixture was added *t*BuOOH (5.5 M in decanes) (92 µl, 0.50 mmol, 3.0 eq.) and reaction progress was tracked via ^{31}P-NMR which indicated full transformation of the phosphite into the generated protected phosphates after 20 minutes. The reaction was then quenched by the addition of water and the product was extracted using dichloromethane. The combined organic phases were dried over Na_2SO_4, filtered off and concentrated *in vacuo*. Purification by silica column chromatography (ethyl acetate/pentane, 1/1 → 9/1, v/v) gave compound **61** (144 mg, 0.168 mmol, quant.). R_f 0.55 (pentane/ethyl acetate, 1/4, v/v). ^{1}H NMR (400 MHz, $CDCl_3$) δ 7.75 – 7.66 (m, 4H), 7.54 – 7.44 (m, 4H), 7.42 – 7.32 (m, 4H), 7.31 – 7.21 (m, 4H), 7.21 – 7.08 (m, 4H), 6.83 – 6.72 (m, 4H), 4.70 (d, *J* = 4.3 Hz, 1H), 4.51 – 4.34 (m, 4H), 4.29 – 4.17 (m, 4H), 4.17 – 4.07 (m, 3H), 3.86 (ddd, *J* = 11.1, 6.1, 3.6 Hz, 1H), 3.76 – 3.68 (m, 8H), 3.63 (dd, *J* = 7.0, 4.2 Hz, 1H), 3.33 (s, 3H). ^{13}C NMR (101 MHz, $CDCl_3$) δ 159.7, 159.5, 143.1, 143.04, 143.03, 143.01, 141.5, 141.4, 139.5, 129.93, 129.90, 129.3, 129.1, 128.1, 127.3, 125.2, 125.1, 125.1, 120.2, 120.1, 114.0, 113.9, 102.3, 81.2 (d, *J* = 7.7 Hz), 77.7, 74.6, 72.61, 72.55, 69.4 (d, *J* = 5.8 Hz), 67.3 (d, *J* = 5.8 Hz), 55.5, 55.4, 55.3, 48.0 (d, *J* = 2.2 Hz), 47.9 (d, *J* = 2.2 Hz). ^{31}P NMR (162 MHz, $CDCl_3$) δ -1.7 (hept, *J* = 6.1 Hz). HRMS (ESI) m/z: $[M+Na]^+$ Calcd for $C_{50}H_{49}O_{10}PNa$ 863.2956; Found 863.2948.

1-α-*O*-methyl-2,3-*O*-(*para*-methoxybenzyl)-5-*O*-phosphate-D-ribofuranoside (20) Compound **61** (84 mg, 0.098 mmol) was dissolved in dry acetonitrile (2.0 mL, 0.049 M) after which a stirring bar was added and the solution was brought under a nitrogen atmosphere while stirring. To this solution was then added triethylamine (0.3 mL, 2 mmol, 20 eq.), the flask was outfitted with a glass stopper and the resulting mixture was allowed to stir at room temperature 70 hours at which point LCMS indicated full deprotection of the phosphate. The mixture was directly concentrated *in* vacuo and was subsequently co-evaporated thrice with a solvent system consisting of 1/1 dry acetonitrile/dry pyridine whereafter it was co-evaporated with dry acetonitrile once more. The resulting crude mixture was used as-is in the next reaction.

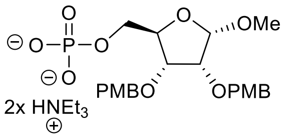

1-α-azido-2,3-*O*-(*para*-methoxybenzyl)-5-*O*-(di(9*H*-fluoren-9-yl))-phosphoryl--D-ribofuranoside (62)

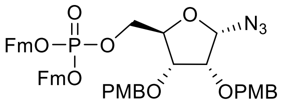

Compound **62** was prepared following procedures previously described.[7,8] All spectra are in full accordance with literary precedence. ^{1}H NMR (400 MHz, $CDCl_3$) δ 7.76 – 7.65 (m, 4H), 7.55 – 7.42 (m, 4H), 7.41 – 7.31 (m, 4H), 7.30 – 7.20 (m, 6H), 7.17 – 7.12 (m, 2H), 6.85 – 6.74 (m, 4H), 4.93 – 4.86 (m, 1H), 4.50 (s, 2H), 4.46 (d, *J* = 11.9 Hz, 1H), 4.33 (d, *J* = 11.8 Hz, 1H), 4.30 – 4.16 (m, 6H), 4.13 – 4.04 (m, 3H), 3.88 (ddd, *J* = 11.3, 6.1, 3.2 Hz, 1H), 3.75 (s, 3H), 3.71 (m, 4H). ^{13}C NMR (101 MHz, $CDCl_3$) δ 159.6, 159.5, 143.11, 143.09, 143.04, 142.95, 141.49, 141.47, 141.45, 129.8, 129.7, 129.5, 129.3, 128.1, 128.0, 127.29, 127.27, 127.25, 125.2, 125.14, 125.11, 125.09, 120.2, 120.1, 114.0, 113.9, 90.5, 81.7 (d, *J* = 7.5 Hz), 77.7, 75.1, 72.9, 72., 69.5 – 69.2 (m, C-P coupling unresolved), 66.6 (d, *J* = 5.7 Hz), 55.4, 55.3, 48.0 (d, *J* = 2.6 Hz), 47.9 (d, *J* = 2.7 Hz). HRMS (ESI) m/z: $[M+Na]^+$ Calcd for $C_{49}H_{46}N_3O_9PNa$ 874.2864; Found 874.2865.

1-α-azido-2,3-*O*-(*para*-methoxybenzyl)-5-*O*-phosphate-D-ribofuranoside (21) Compound **21** was prepared following a procedure previously published.[7,8] The crude product was used directly in the next reaction.

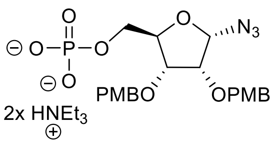

Scheme S8: Synthesis of GS-441524 P(III) donors and NDPr molecules

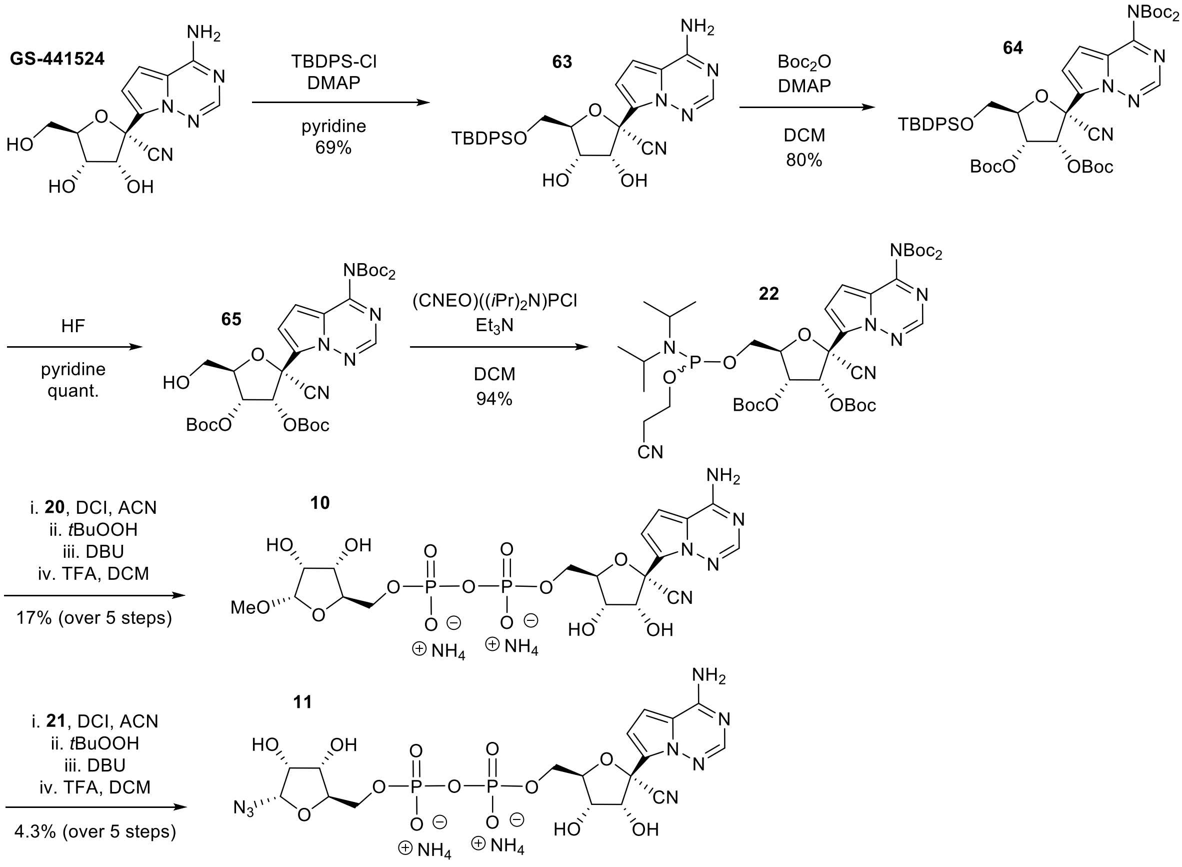

GS-441524 GS-441524 was prepared following procedures previously described.[9–11] All spectra are in full accordance with literary precedence. ^{1}H NMR (400 MHz, DMSO) δ 8.03 – 7.76 (m, 3H), 6.92 (d, *J* = 4.5 Hz, 1H), 6.88 (d, *J* = 4.6 Hz, 1H), 6.14 (d, *J* = 6.2 Hz, 1H), 5.23 (d, *J* = 5.1 Hz, 1H), 4.95 (t, *J* = 5.8 Hz, 1H), 4.63 (t, *J* = 5.7 Hz, 1H), 4.05 (q, *J* = 4.4 Hz, 1H), 3.95 (q, *J* = 5.2 Hz, 1H), 3.68 – 3.58 (m, 1H), 3.55 – 3.45 (m, 1H). ^{13}C NMR (101 MHz, DMSO) δ 155.6, 147.9, 123.9, 117.4, 116.5, 110.8, 100.9, 85.4, 78.5, 74.3, 70.1, 60.9. HRMS (ESI) m/z: $[M+H]^+$ Calcd for $C_{12}H_{14}N_5O_4$ 292.1040; Found 292.1042.

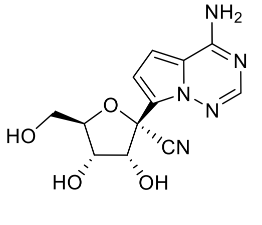

5-*O*-(*tert*-butyl-diphenylsilyl) GS-441524 (63) GS-441524 (29 mg, 0.10 mmol) was co-evaporated thrice with dry pyridine and subsequently dissolved in dry pyridine (2.0 mL, 0.050 M) while under an atmosphere of nitrogen gas. To this mixture were then added flame dried molecular sieves (3Å rods) and DMAP (cat.) and the resulting mixture was stirred for 15 minutes. The mixture was then cooled to 0 °C and TBDPS-Cl (62 µL, 0.24 mmol, 2.4 eq.) was added and the reaction was allowed to warm to room temperature where it was stirred overnight. The next day, TLC analysis indicated full consumption of the starting material and the reaction was subsequently quenched by the addition of water. The product was then extracted using dichloromethane as the organic phase and the combined organic phases were washed with sat. aq. sodium bicarbonate solution and brine, respectively. The organic phase was then dried over Na_2SO_4, filtered off and concentrated *in vacuo*. Purification by silica column chromatography (ethyl acetate/pentane, 3/2 → 1/0, v/v) gave compound **63** (37 mg, 69 µmol, 69%). R_f 0.6 (ethyl acetate). ^{1}H NMR (400 MHz, Acetone) δ 7.88 (s, 1H), 7.69 (d, *J* = 1.5 Hz, 2H), 7.67 (d, *J* = 1.5 Hz, 2H), 7.47 – 7.41 (m, 2H), 7.40 – 7.34 (m, 4H), 7.26 (bs, 1H), 6.91 (d, *J* = 4.6 Hz, 1H), 6.89 (d, *J* = 4.5 Hz, 1H), 5.63 (d, *J* = 5.3 Hz, 1H), 4.94 (t, *J* = 4.2 Hz, 1H), 4.45 (q, *J* = 4.4, 3.9 Hz, 2H), 4.37 (q, *J* = 3.9 Hz, 1H), 4.00 (dd, *J* = 11.5, 3.3 Hz, 1H), 3.90 (dd, *J* = 11.5, 4.0 Hz, 1H), 2.88 (s, 1H), 0.98 (s, 9H). ^{13}C NMR (101 MHz, Acetone) δ 157.0, 148.9, 136.43, 136.40, 134.0, 130.8, 130.7, 128.7, 117.6, 111.7, 101.4, 86.8, 76.0, 71.9, 64.4, 27.2, 19.8. HRMS (ESI) m/z: $[M+H]^+$ Calcd for $C_{28}H_{32}N_5O_4Si$ 530.2218; Found 530.2229.

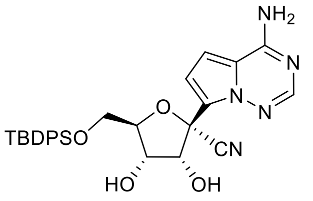

***N,N*-di-(*tert*-butyloxycarbonyl)-2,3-O-di-(*tert*-butyloxycarbonyl)-5-*O*-(*tert*-butyl-diphenylsilyl) GS-441524 (64)**

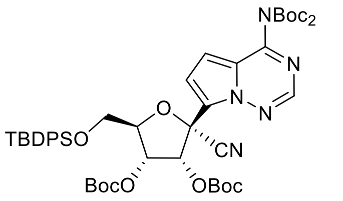

Compound **63** (656 mg, 1.24 mmol) was dissolved in dry dichloromethane (25 mL, 0.050 M) the resulting solution was stirred under an atmosphere of nitrogen gas. To the stirring mixture was added DMAP (cat.) and di-*tert*-butyl dicarbonate (2.8 mL, 12 mmol, 10 eq.) and the resulting mixture was allowed to stir overnight. The next day, TLC analysis indicated full conversion and triethylamine (0.5 mL) and celite were added after which the mixture was directly concentrated *in vacuo*. Purification by silica column chromatography (diethyl ether/pentane, 1/19 → 1/1, v/v) gave desired compound **64** (0.919 grams, 0.989 mmol, 80%) as a white foam. R_f 0.5 (diethyl ether/pentane, 3/7, v/v). ^{1}H NMR (400 MHz, $CDCl_3$) δ 8.39 (s, 1H), 7.63 – 7.54 (m, 4H), 7.44 – 7.35 (m, 2H), 7.33 – 7.27 (m, 4H), 7.17 (d, *J* = 4.8 Hz, 1H), 6.73 (d, *J* = 4.8 Hz, 1H), 6.02 (d, *J* = 5.8 Hz, 1H), 5.53 (t, *J* = 5.6 Hz, 1H), 4.58 (dt, *J* = 6.0, 3.2 Hz, 1H), 3.98 (dd, *J* = 11.8, 3.2 Hz, 1H), 3.88 (dd, *J* = 11.8, 3.3 Hz, 1H), 1.52 (s, 9H), 1.49 (s, 9H), 1.43 (s, 18H), 1.01 (s, 9H). ^{13}C NMR (101 MHz, $CDCl_3$) δ 153.3, 152.5, 151.8, 149.7, 146.6, 135.7, 135.6, 132.8, 132.6, 130.0, 129.9, 127.9, 127.8, 123.7, 122.5, 115.2, 114.7, 103.1, 84.7, 83.9, 83.4, 83.1, 76.5, 74.9, 72.2, 62.3, 27.8, 27.8, 27.7, 26.9, 19.3. HRMS (ESI) m/z: $[M+H]^+$ Calcd for $C_{48}H_{64}N_5O_{12}Si$ 930.4315; Found 930.4330.

***N,N*-di-(*tert*-butyloxycarbonyl)-2,3-O-di-(*tert*-butyloxycarbonyl) GS-441524 (65)**

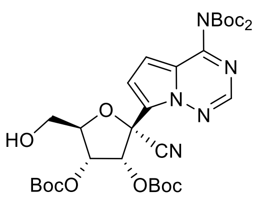

Compound **64** (0.315 grams, 0.338 mmol) was dissolved in pyridine and brought to a 15 mL falcon tube where it was stirred and cooled to 0 °C using an ice bath. To this stirring mixture was added HF-pyridine (70% HF, w/w) and the resulting reaction mixture was allowed to stir at 4 °C overnight. The next day, TLC analysis indicated full conversion of the starting material and the reaction mixture was successively cooled to 0 °C and carefully quenched with sat. aq. sodium bicarbonate solution. The mixture was diluted with dichloromethane and the organic phase was washed with water and brine. The organic phase was then dried over Na_2SO_4, filtered off and concentrated *in vacuo*. Purification by silica column chromatography (ethyl acetate/pentane, 1/9 → 35/65, v/v) gave compound **65** (0.241 grams, 0.338 mmol, quant.) as a colourless oil. TLC: R_f 0.45 (ethyl acetate/pentane, 3/7, v/v). ^{1}H NMR (400 MHz, $CDCl_3$) δ 8.46 (s, 1H), 7.36 (d, *J* = 4.9 Hz, 1H), 6.82 (d, *J* = 4.8 Hz, 1H), 5.99 (d, *J* = 6.1 Hz, 1H), 5.53 (dd, *J* = 6.1, 2.9 Hz, 1H), 4.63 (dt, *J* = 2.9, 1.4 Hz, 1H), 4.01 (dd, *J* = 10.1, 2.0 Hz, 1H), 3.96 – 3.86 (m, 2H), 1.52 (s, 9H), 1.44 (s, 18H), 1.37 (s, 9H). ^{13}C NMR (101 MHz, $CDCl_3$) δ 154.0, 152.6, 151.4, 149.5, 146.8, 123.3, 122.7, 117.4, 115.2, 103.7, 85.8, 84.9, 83.9, 83.4, 78.1, 77.4, 73.6, 73.1, 62.1, 27.8, 27.5. HRMS (ESI) m/z: $[M+Na]^+$ Calcd for $C_{32}H_{45}N_5O_{12}Na$ 714.2957; Found 714.2958.

2-cyanoethoxy-*N'*,*N'*-di-*iso*-propylamino-(6-*N,N*-di-(*tert*-butyloxycarbonyl)-2',3'-*O*-di-(*tert*-butyloxycarbonyl)phosphine GS-441524 (22)

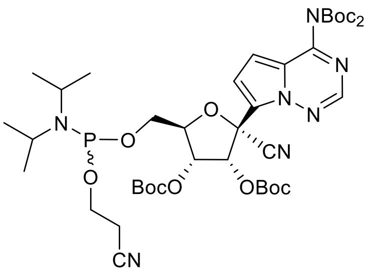

Compound **65** (240 mg, 0.338 mmol) was co-evaporated thrice with dry toluene, purged with argon gas and brought under an argon atmosphere. The compound was then dissolved in dry dichloromethane (7.0 mL, 0.048 M) and stirred vigorously. To the stirring solution were added triethylamine (161 µL, 1.16 mmol, 3.43 eq.) and 2-cyanoethyl *N,N*-di-*iso*-propylchlorophosphoramidite (94 µL, 0.42 mmol, 1.2 eq.), successively. The reaction was stirred for 90 minutes, when ^{31}P-NMR monitoring indicated full conversion of the chlorophosphoramidite into the corresponding phosphoramidite (180 ppm → 150 ppm). The reaction was then diluted with dichloromethane (14 mL) and quenched by the addition of aq. sodium bicarbonate solution (5% w/w). The organic phase was collected and dried over $MgSO_4$, filtered off and concentrated *in vacuo*. Purification by column chromatography using neutralized silica gel (ethyl acetate/pentane + 1% triethylamine, 1/19 → 2/3, v/v) gave compound **22** (293 mg, 0.328 mmol, 94%) as a mixture of P(III) diastereomers as a colourless oil. R_f 0.75 (ethyl acetate/pentane, 3/7, v/v). ^{1}H NMR (400 MHz, $CDCl_3$) δ 8.43 (ap. d, *J* = 10.8 Hz, 1H), 7.17 (ap. dd, *J* = 14.1, 4.8 Hz, 1H), 6.71 (ap. d, *J* = 4.8 Hz, 1H), 5.90 (ap. dd, *J* = 9.4, 5.7 Hz, 1H), 5.37 – 5.24 (m, 1H), 4.64 – 4.53 (m, 1H), 3.94 (ap. ddd, *J* = 11.2, 7.0, 3.9 Hz, 1H), 3.87 – 3.60 (m, 3H), 3.56 – 3.34 (m, 2H), 2.58 – 2.51 (m, 2H), 1.45 (m, 9H), 1.41 – 1.37 (m, 27H), 1.09 - 1.05 (m, 9H), 0.93 (d, *J* = 6.8 Hz, 3H). ^{13}C NMR (101 MHz, $CDCl_3$) δ 153.3, 152.6, 152.4, 151.8, 151.7, 149.8, 146.59, 146.55, 123.8, 123.6, 122.4, 117.8, 117.7, 115.3, 115.0, 114.6, 114.6, 103.2, 103.1, 84.8, 84.7, 83.94, 83.90, 83.5, 83.4, 82.83, 82.75, 82.2, 82.1, 77.4, 76.7, 75.9, 75.1, 74.7, 72.9, 72.7, 62.1, 62.1, 62.0, 61.9, 58.8, 58.68, 58.65, 58.5, 43.3, 43.23, 43.18, 43.1, 27.82, 27.76, 27.74, 27.66, 24.72, 24.68, 24.64, 24.61, 24.59, 24.52, 24.45, 20.5, 20.4, 20.3. ^{31}P NMR (162 MHz, $CDCl_3$) δ 149.4 (hept, *J* = 7.5 Hz), 149.0 (hept, *J* = 7.3 Hz). HRMS (ESI) m/z: $[M+O+Na]^+$ Calcd for $C_{41}H_{62}N_7O_{14}PNa$ 930.3985; Found 930.3986.

1”-α-*O*-methyl-RDPr (10) To crude compound **20** (~ 0.1 mmol) was added DCI (30 mg, 0.25 mmol, 2.5 eq.) and the resulting mixture was co-evaporated thrice with dry acetonitrile after which it was brought under a nitrogen atmosphere and suspended in dry acetonitrile (1.8 mL, 0.055 M). In another flask, phosphoramidite **22** was co-evaporated thrice with dry and distilled toluene after which it was brought under a nitrogen atmosphere and it was dissolved in dry acetonitrile to create a stock solution (0.22 M). Part of this stock solution (0.45 mL, 0.10 mmol, 1.0 eq.) was then added to the stirring suspension and the resulting yellow suspension was stirred for 80 minutes, after which to it was added *t*BuOOH (5.5 M in decanes) (38 µL, 0.21 mmol, 2.1 eq.) once and again after one hour of reaction time (38 µL, 0.21 mmol, 2.1 eq.). After stirring at room temperature for an additional 35 minutes reaction progress was checked by ^{31}P-NMR which indicated full oxidation of all P(III) species to P(V) (~140 ppm → ~5 ppm) and to the stirring solution was added DBU (75 µL, 0.50 mmol, 5.0 eq.) and the resulting solution was directly concentrated *in vacuo* whereafter it was suspended once more in dry dichloromethane (1.5 mL) and to it was added tri-*iso*-propylsilane hydride (TIS-H) (51 µL, 0.50 mmol, 5.0 eq.) and TFA (0.36 mL) in that order to create a solvent system consisting of TFA in dichloromethane (0.055 M, 20% TFA in dichloromethane, v/v). The reaction was monitored using LCMS to indicate full deprotection of the construct at which point the solution was directly concentrated *in vacuo*. The crude mixture was then extracted using ethyl acetate and water, washings of the water phase with ethyl acetate were continued until the ethyl acetate returned clear and colourless. The aqueous phase was then concentrated *in vacuo*. Purification by gel filtration (HW-40, NH_4OAc buffer) followed by HPLC and washing over an ion-exchange resin afforded compound **10** (10.5 mg, 16.7 µmol, 17% (over 5 steps)) as the ammonium salt as a white foam. ^{1}H NMR (400 MHz, D_2O) δ 7.91 (s, 1H), 7.00 (d, *J* = 4.7 Hz, 1H), 6.90 (d, *J* = 4.7 Hz, 1H), 4.95 (d, *J* = 5.4 Hz, 1H), 4.81 (d, *J* = 3.5 Hz, 1H), 4.50 (q, *J* = 3.6 Hz, 1H), 4.46 (dd, *J* = 5.4, 3.7 Hz, 1H), 4.16 – 3.99 (m, 5H), 3.86 (t, *J* = 4.7 Hz, 2H), 3.34 (s, 3H). ^{13}C NMR (101 MHz, D_2O) δ 155.5, 147.2, 123.0, 117.0, 116.6, 111.1, 103.2, 102.4, 85.0 (d, *J* = 8.9 Hz), 83.2 (d, *J* = 7.7 Hz), 76.7, 74.8, 70.8, 70.3, 69.6, 65.5 (d, *J* = 5.0 Hz), 64.8 (d, *J* = 3.6 Hz), 55.4. ^{31}P NMR (162 MHz, D_2O) δ -11.4 (q, *J* = 5.9 Hz). LCMS: $[M+H]^+$ Calcd for $C_{18}H_{26}N_5O_{14}P_2$ 598.0946; Found 598.0000. HRMS (ESI) m/z: $[M+H]^+$ Calcd for $C_{18}H_{26}N_5O_{14}P_2$ 598.0946; Found 598.0944.

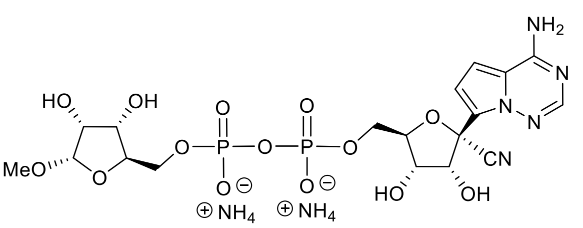

1”-α-azido-RDPr (11) Compound **21** (~98 µmol) was co-evaporated thrice with a 1:1 mixture of dry acetonitrile and pyridine, then once with just acetonitrile after which DCI (30 mg, 0.25 mmol, 2.5 eq.) was added. The resulting mixture was then co-evaporated three more times with dry acetonitrile after which it was brought under an argon atmosphere and suspended in dry acetonitrile (1.81 mL, 0.055 M). In a separate flask, compound **22** (87 mg, 98 µmol, 1.0 eq.) was co-evaporated three times with dry toluene, after which it was brought under an argon atmosphere and dissolved in dry acetonitrile (0.45 mL, 0.22 M). The clear solution of compound **22** was then added to the suspension containing crude compound **21** and DCI dropwise. The resulting solution was stirred for 1 hour and 20 minutes, after which TLC analysis indicated full consumption of the phosphoramidite. To the solution was then added *t*BuOOH (5.5 M in decanes) (38 µL, 0.21 mmol, 2.1 eq.) once and again after one hour of reaction time (38 µL, 0.21 mmol, 2.1 eq.). Reaction progress was checked after 40 more minutes of stirring by ^{31}P-NMR which indicated full oxidation of all P(III) species to P(V) (~140 ppm → ~5 ppm). To the mixture was then added DBU (75 µL, 0.49 mmol, 5.0 eq.) and the reaction was stirred for 75 minutes, after which ^{31}P-NMR indicated full conversion of the protected pyrophosphate into the unprotected pyrophosphate (~5 ppm → -10 ppm). The reaction mixture was then directly concentrated *in vacuo*, cooled to 0 °C and dissolved in dichloromethane (1.45 mL) to which were added TIS-H (51 µL, 0.25 mmol, 2.5 eq.). and TFA (0.36 mL) to obtain a solvent system consisting of 20% TFA in dichloromethane (1.86 mL total, 0.053 M). The reaction mixture was allowed to warm to room temperature and was stirred for five hours after which LCMS analysis of the mixture indicated full deprotection of the construct. The mixture was then directly concentrated *in vacuo*. The resulting red sludge was dissolved in a mixture of 1/1 ethyl acetate and water (7 mL + 7 mL) and the aqueous phase was washed with an equal amount of ethyl acetate until the organic phase came back as colourless. The aqueous phase was then separated and concentrated *in vacuo* to give a colourless syrup, which was subjected to HW-40 size exclusion chromatography (NH_4OAc buffer) and HPLC purification as well as washing over an ion-exchange resin, followed by lyophilization to afford compound **11** as the ammonium salt as a white foam. (2.73 mg, 4.25 µmol, 4.3% (over 5 steps)) as a white foam. ^{1}H NMR (500 MHz, D_2O) δ 7.92 (s, 1H), 7.01 (d, *J* = 4.7 Hz, 1H), 6.92 (d, *J* = 4.9 Hz, 1H), 5.29 (d, *J* = 4.8 Hz, 1H), 4.96 (d, *J* = 4.9 Hz, 1H), 4.53 – 4.47 (m, 1H), 4.47 – 4.42 (m, 1H), 4.18 (t, *J* = 5.0 Hz, 1H), 4.17 – 4.02 (m, 4H), 3.87 (m, 2H). ^{13}C NMR (126 MHz, D_2O) δ 155.6, 147.3, 123.0, 117.0, 116.6, 111.0,

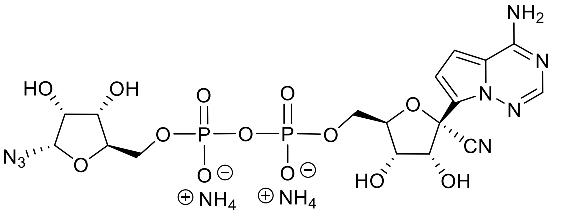

102.3, 91.3, 84.9 (d, *J* = 7.9 Hz), 83.9 (d, *J* = 7.8 Hz), 76.6, 74.7, 71.4, 70.3, 69.5, 65.1 (d, *J* = 5.2 Hz), 64.7 (d, *J* = 4.3 Hz). ^{31}P NMR (202 MHz, D_2O) δ -11.2 – -11.6 (m). LCMS: $[M+H]^+$ Calcd for $C_{17}H_{23}N_8O_{13}P_2$ 609.0854; Found 608.9167. HRMS (ESI) m/z: $[M+H]^+$ Calcd for $C_{17}H_{23}N_8O_{13}P_2$ 609.0854; Found 609.0846

References

(1) Gavel, M.; Courant, T.; Joosten, A. Y. P.; Lecourt, T. Regio- A Nd Chemoselective Deprotection of Primary Acetates by Zirconium Hydrides. *Org Lett*, **2019**, *21* (7), 1948–1952.

(2) Kistemaker, H. A. V.; Lameijer, L. N.; Meeuwenoord, N. J.; Overkleeft, H. S.; van der Marel, G. A.; Filippov, D. V. Synthesis of Well-Defined Adenosine Diphosphate Ribose Oligomers. *Angewandte Chemie*, **2015**, *127* (16), 4997–5000.

(3) Heinrich, M.; Murphy, J. J.; Ilg, M. K.; Letort, A.; Flasz, J. T.; Philipps, P.; Fü, A. Chagosensine: A Riddle Wrapped in a Mystery Inside an Enigma. *Journal of the American Chemical Society*, **2020**, *142* (13), 6409-6422.

(4) Bukownik, R. R.; Wilcox, C. S. Synthetic Receptors. 3,6-Anhydro-7-Benzenesulfonamido-1,7-Dideoxy-4,5-O-Isopropylidene-D-Altro-Hept-1-Ynitol: A Useful Component for the Preparation of Chiral Water-Soluble Cyclophanes Based on Carbohydrate Precursors. *Journal of Organic Chemistry*, **1988**, *53* (3), 463–471.

(5) Hananya, N.; Daley, S. K.; Bagert, J. D.; Muir, T. W. Synthesis of ADP-Ribosylated Histones Reveals Site-Specific Impacts on Chromatin Structure and Function. *J Am Chem Soc*, **2021**, *143* (29), 10847–10852.

(6) Kistemaker, H. A. V.; Van Der Heden Van Noort, G. J.; Overkleeft, H. S.; Van Der Marel, G. A.; Filippov, D. V. Stereoselective Ribosylation of Amino Acids. *Org Lett*, **2013**, *15* (9), 2306–2309.

(7) Minnee, H.; Chung, H.; Rack, J. G. M.; van der Marel, G. A.; Overkleeft, H. S.; Codée, J. D. C.; Ahel, I.; Filippov, D. V. Four of a Kind: A Complete Collection of ADP-Ribosylated Histidine Isosteres Using Cu(I)- and Ru(II)-Catalyzed Click Chemistry. *Journal of Organic Chemistry*, **2023**, *88* (15), 10801–10809.

(8) Minnee, H.; Rack, J. G. M.; Van Der Marel, G. A.; Overkleeft, H. S.; Codée, J. D. C.; Ahel, I.; Filippov, D. V. Mimetics of ADP-Ribosylated Histidine through Copper(I)-Catalyzed Click Chemistry. *Org Lett*, **2022**, *24* (21), 3776–3780.

(9) Vargas, D. F.; Larghi, E. L.; Kaufman, T. S. Evolution of the Synthesis of Remdesivir. Classical Approaches and Most Recent Advances. *ACS Omega*, **2021**, *6* (30), 19356–19363.

(10) Cardoza, S.; Shrivash, M. K.; Riva, L.; Chatterjee, A. K.; Mandal, A.; Tandon, V. Multistep Synthesis of Analogues of Remdesivir: Incorporating Heterocycles at the C-1′ Position. *Journal of Organic Chemistry*, **2023**, 88 (13), 9105–9122.

(11) Vieira, T.; Stevens, A. C.; Chtchemelinine, A.; Gao, D.; Badalov, P.; Heumann, L. Development of a Large-Scale Cyanation Process Using Continuous Flow Chemistry En Route to the Synthesis of Remdesivir. *Org Process Res Dev*, **2020**, *24* (10), 2113–2121.

(12) Schuller, M.; Correy, G. J.; Gahbauer, S.; Fearon, D.; Wu, T.; Díaz, R. E.; Young, I. D.; Carvalho Martins, L.; Smith, D. H.; Schulze-Gahmen, U.; Owens, T. W.; Deshpande, I.; Merz, G. E.; Thwin, A. C.; Biel, J. T.; Peters, J. K.; Moritz, M.; Herrera, N.; Kratochvil, H. T.; Aimon, A.; Bennett, J. M.; Brandao Neto, J.; Cohen, A. E.; Dias, A.; Douangamath, A.; Dunnett, L.; Fedorov, O.; Ferla, M. P.; Fuchs, M. R.; Gorrie-Stone, T. J.; Holton, J. M.; Johnson, M. G.; Krojer, T.; Meigs, G.; Powell, A. J.; Rack, J. G. M.; Rangel, V. L.; Russi, S.; Skyner, R. E.; Smith, C. A.; Soares, A. S.; Wierman, J. L.; Zhu, K.; O'Brien, P.; Jura, N.; Ashworth, A.; Irwin, J. J.; Thompson, M. C.; Gestwicki, J. E.; von Delft, F.; Shoichet, B. K.; Fraser, J. S.; Ahel, I. Fragment Binding to the Nsp3 Macrodomain of SARS-CoV-2 Identified through Crystallographic Screening and Computational Docking. *Sci Adv*, **2021**, *7* (16), 1–24.

NMR data

Compound **24**

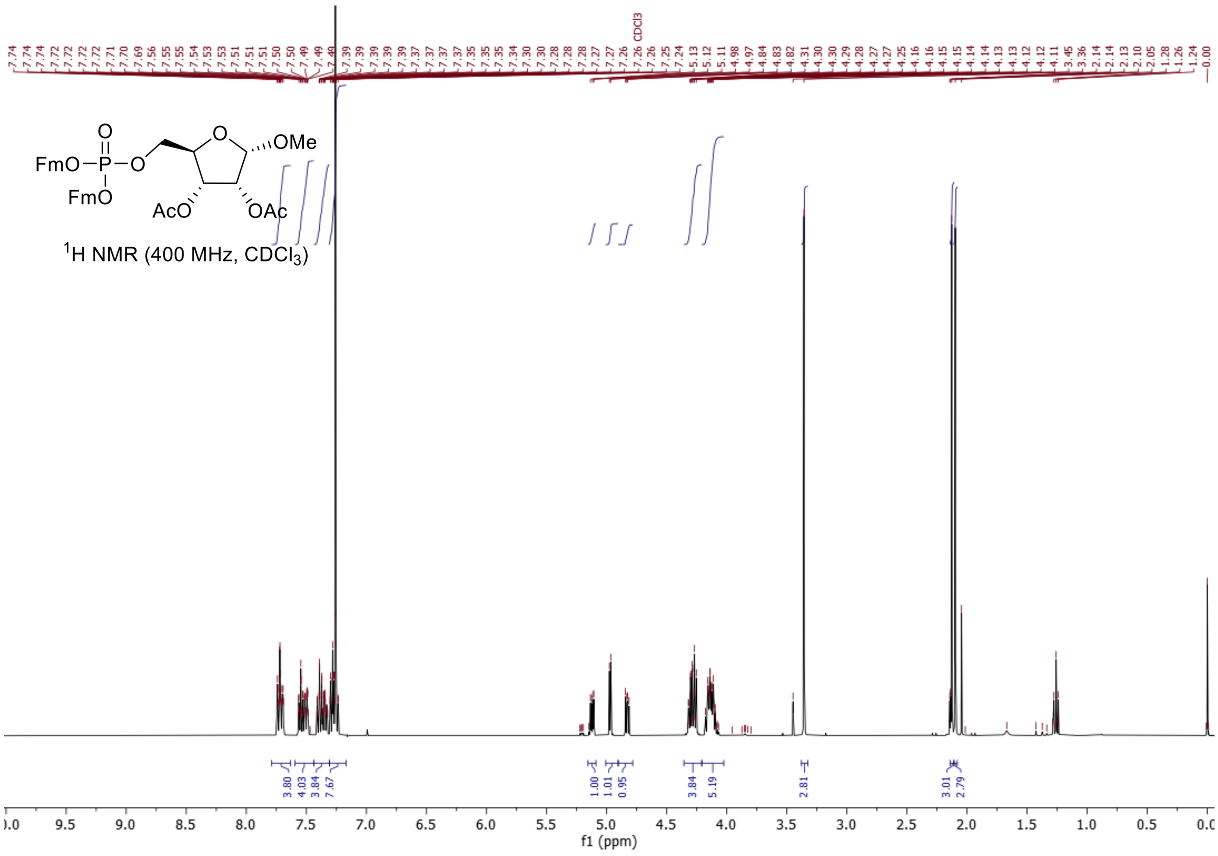

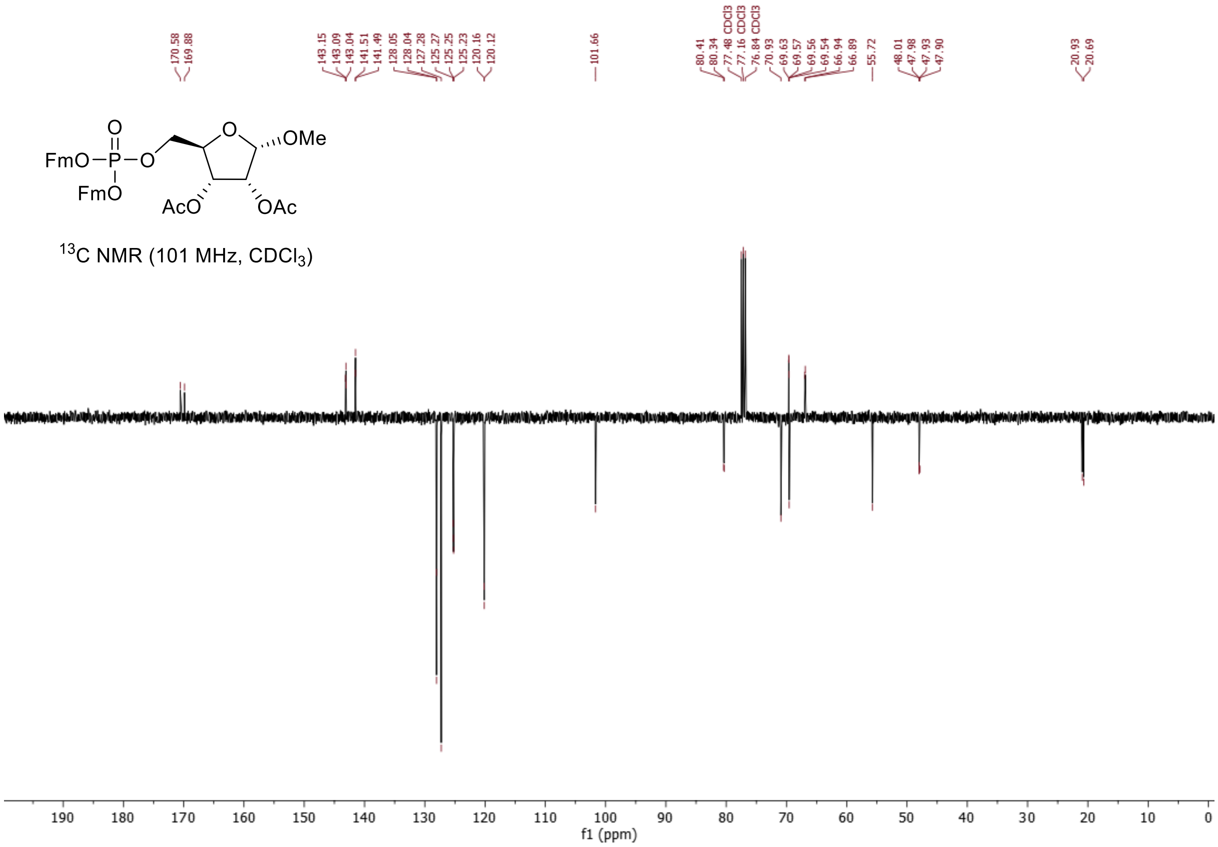

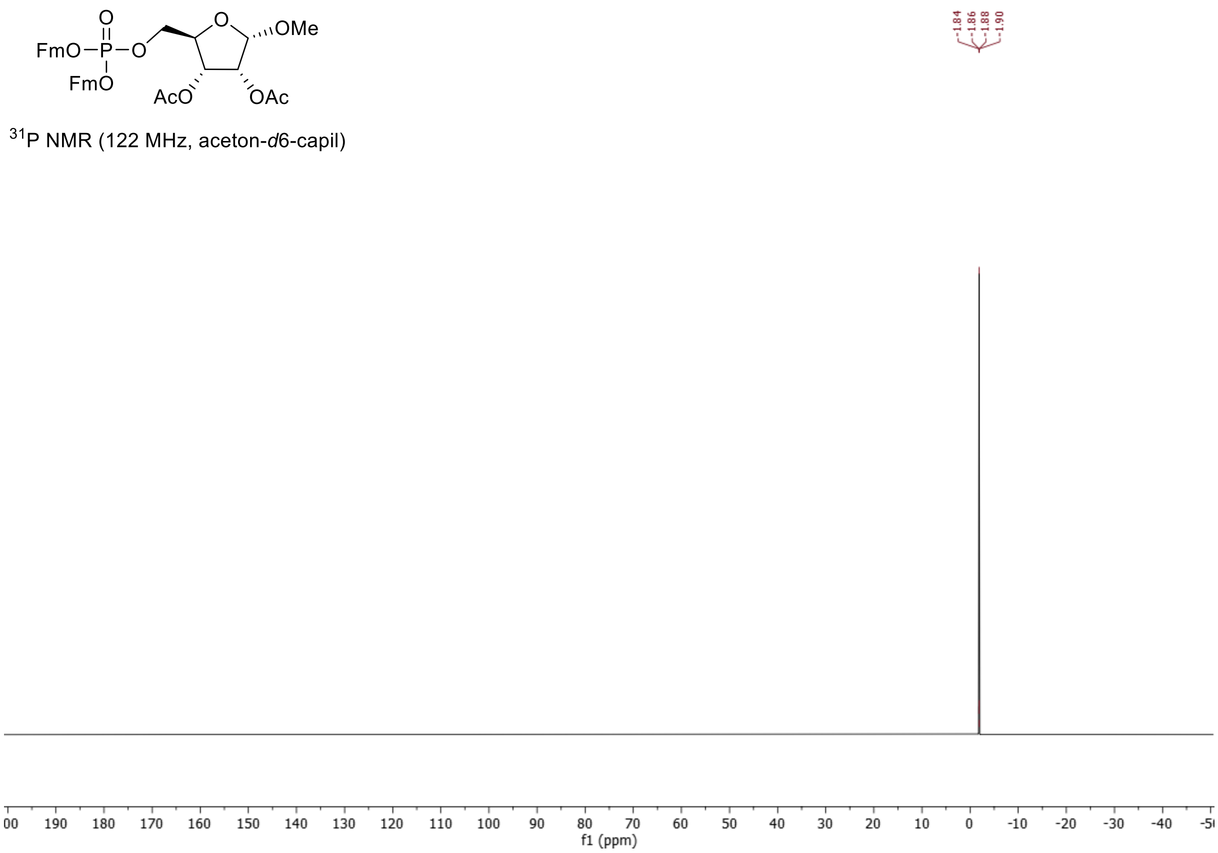
FmO
FmO
OMe
AcO
OAc
^{31}P NMR (122 MHz, aceton-*d*6-capil)
f1 (ppm)

Compound **1**

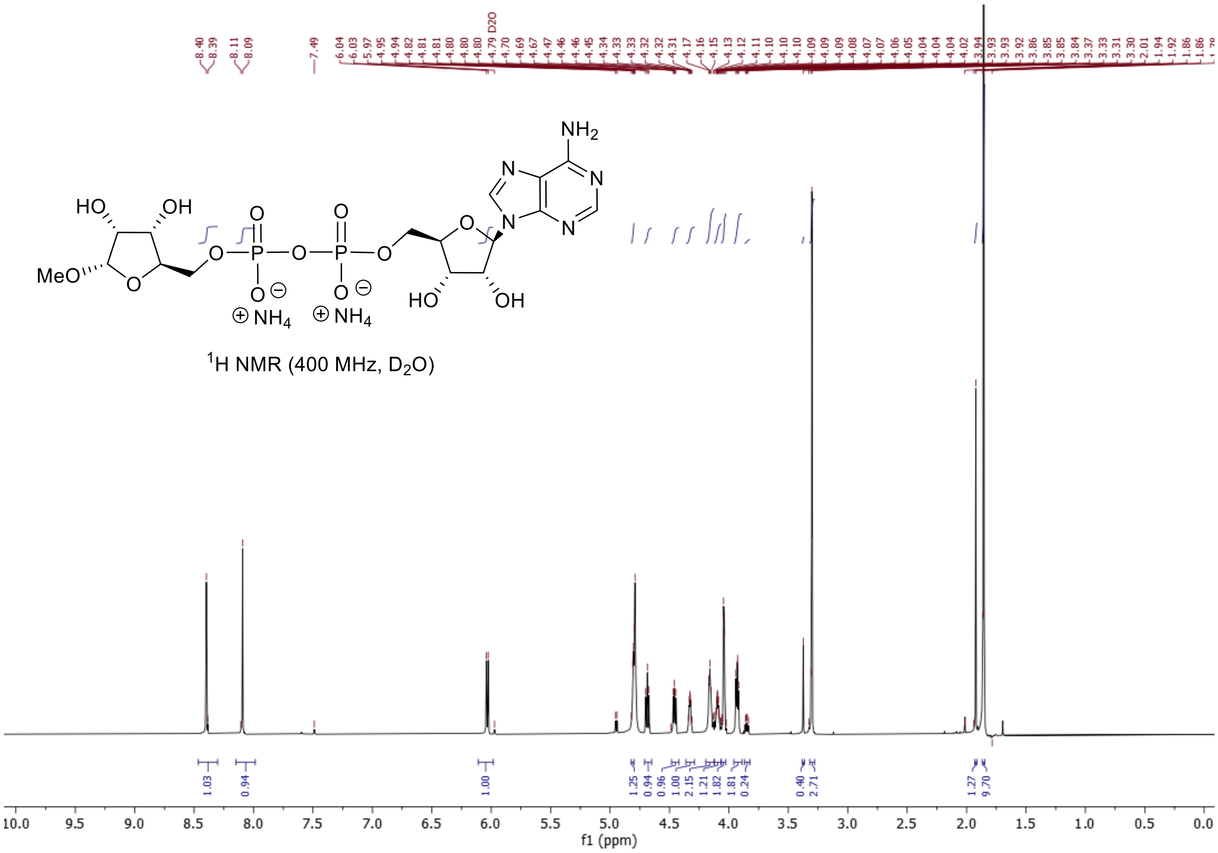
NH2
HO
OH
MeO
HO
OH
NH4
NH4
^{1}H NMR (400 MHz, D_2O)
f1 (ppm)

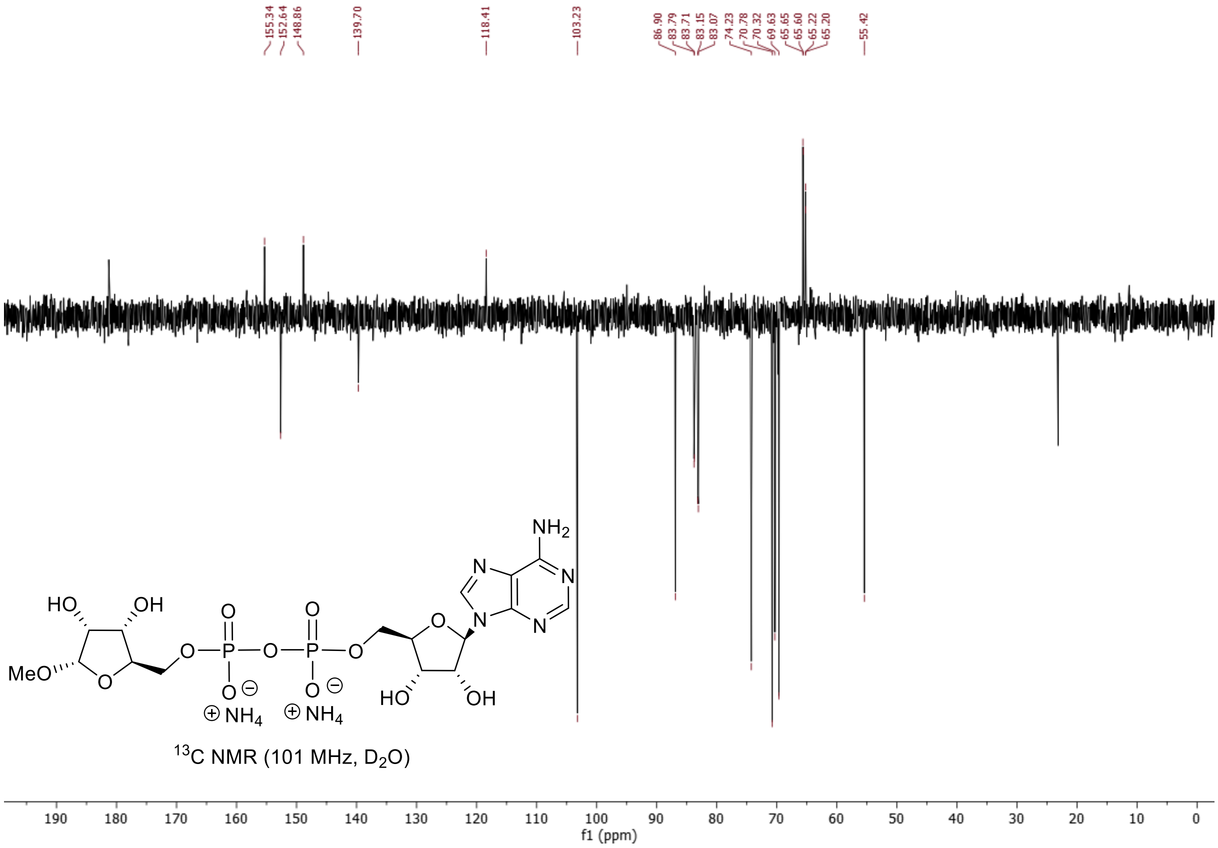
155.34
152.64
148.86
139.70
118.41
103.23
86.90
83.79
83.71
83.15
83.07
74.23
70.78
70.32
69.63
65.65
65.60
65.22
65.20
55.42
NH2
N
N
N
N
HO
OH
O
O
O
P
P
O
O
O
O
MeO
O
O
O
HO
OH
NH4
NH4
13C NMR (101 MHz, D2O)
f1 (ppm)

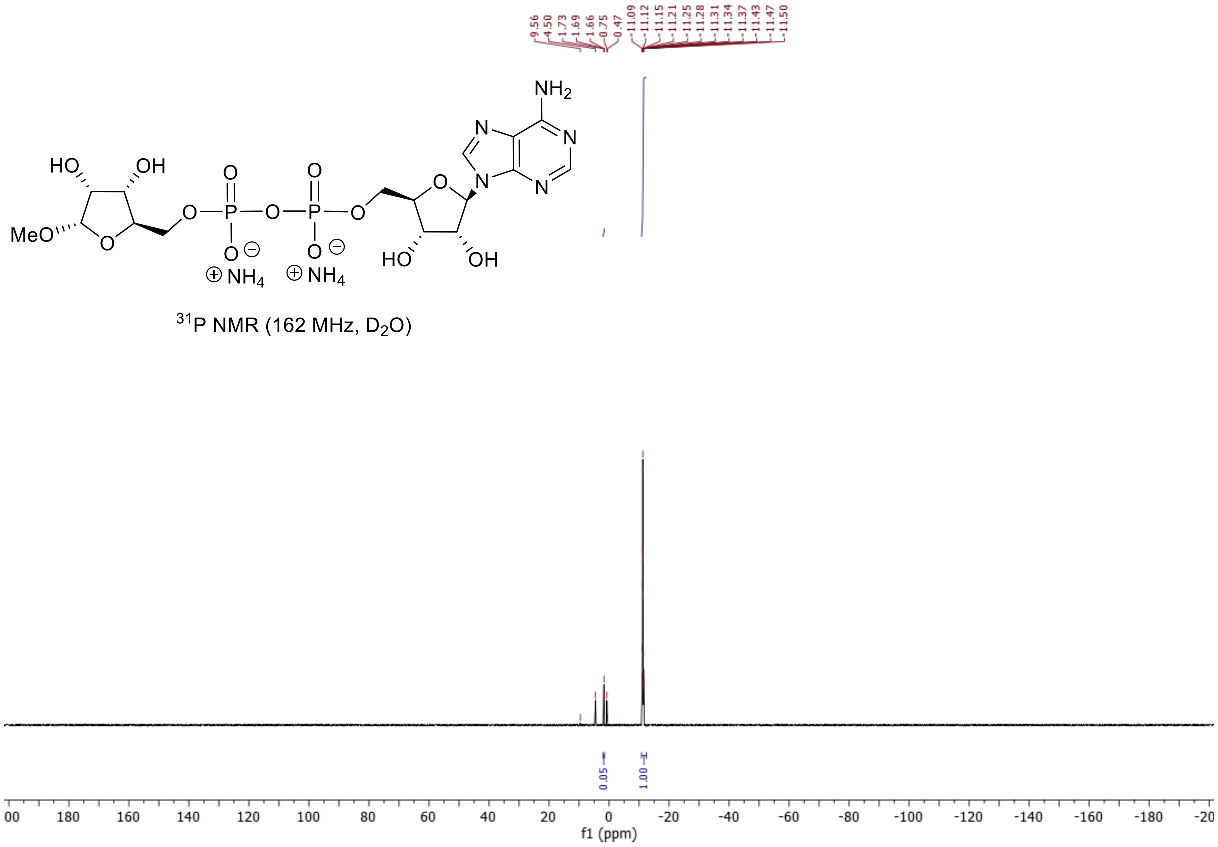
9.56
4.50
1.73
1.69
1.66
0.75
0.47
-11.09
-11.12
-11.15
-11.21
-11.25
-11.28
-11.31
-11.34
-11.37
-11.43
-11.47
-11.50
NH2
HO
OH
MeO
HO
OH
NH4
NH4
31P NMR (162 MHz, D2O)
0.05
1.00
f1 (ppm)

Compound **27**

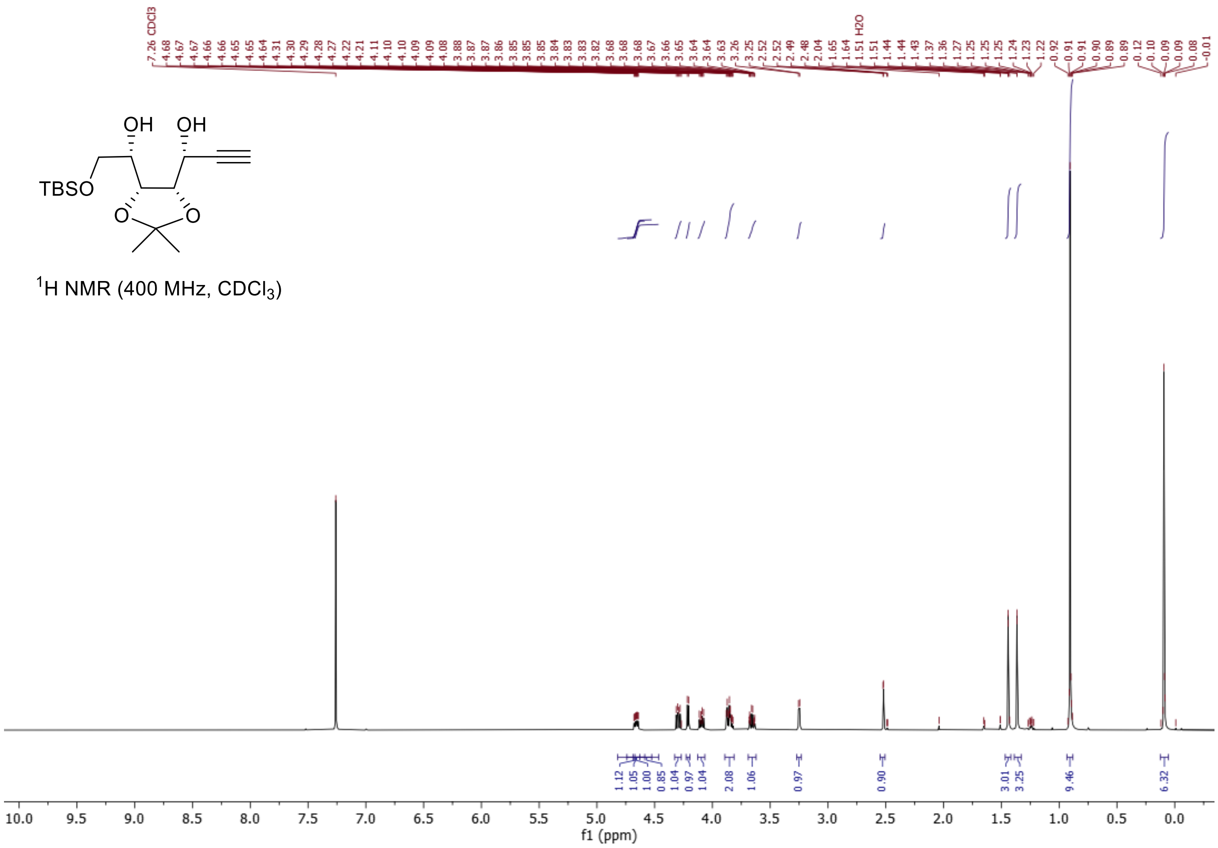

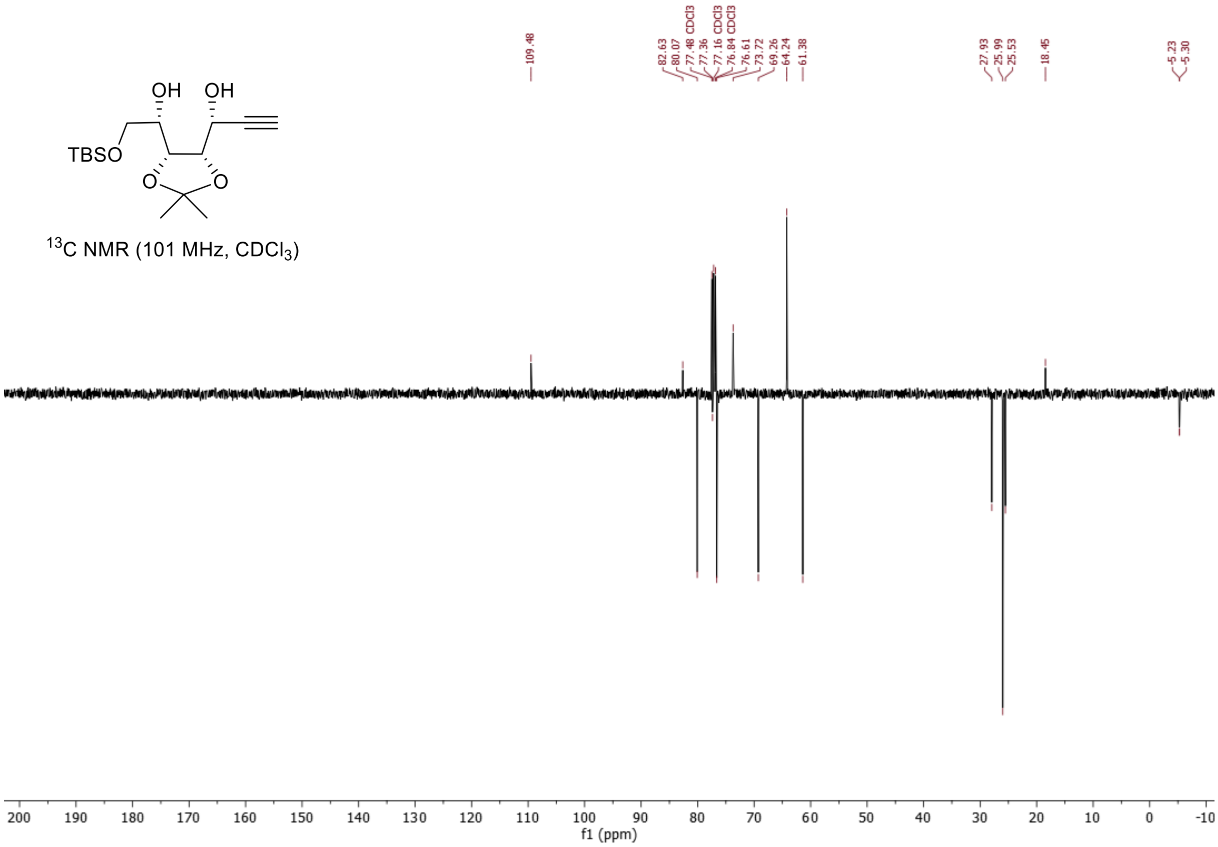

Compound **13**

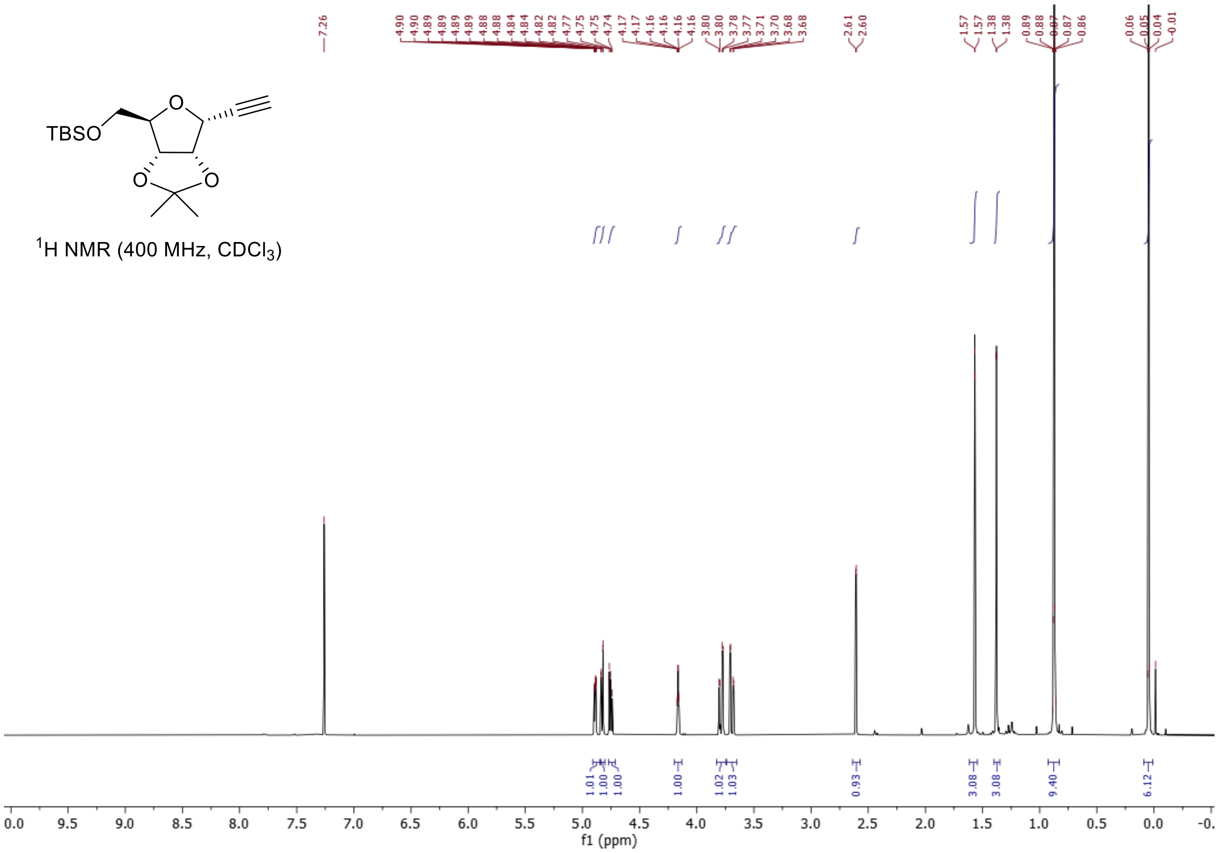

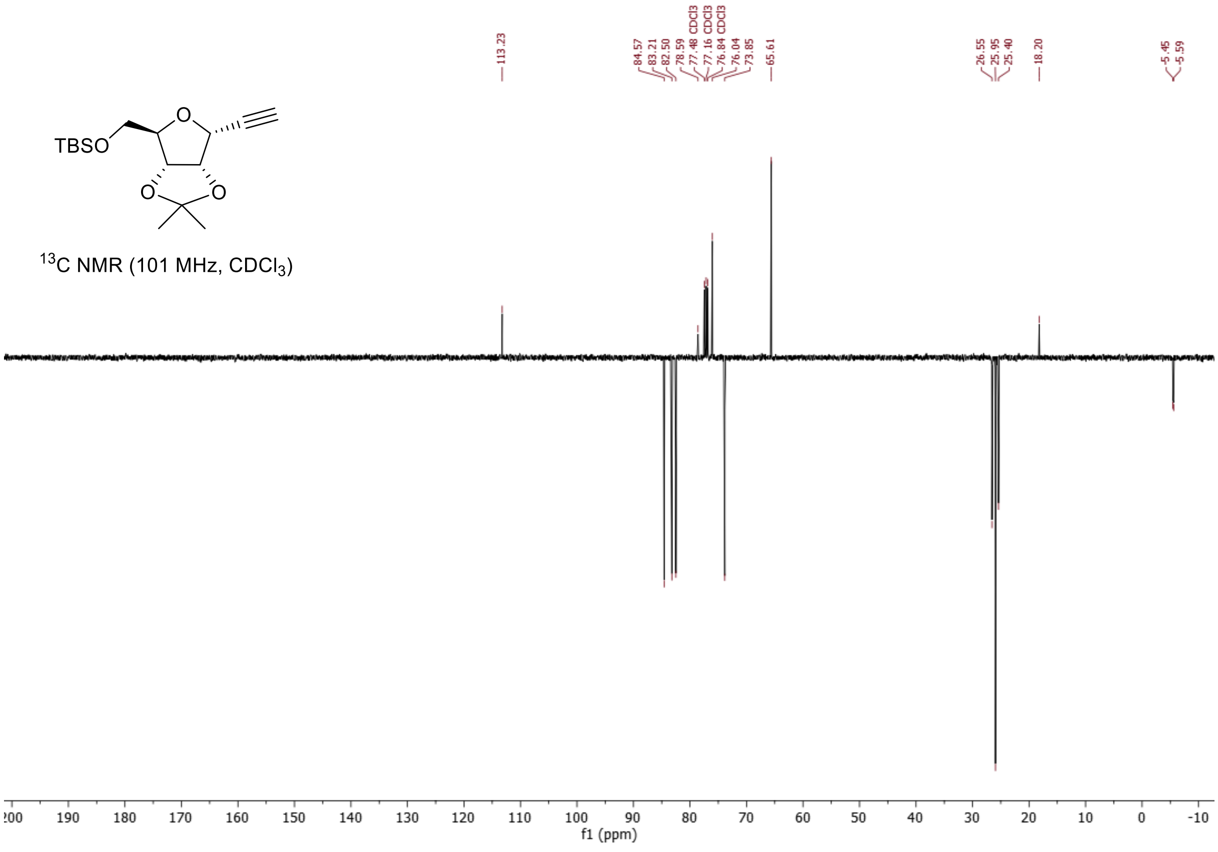

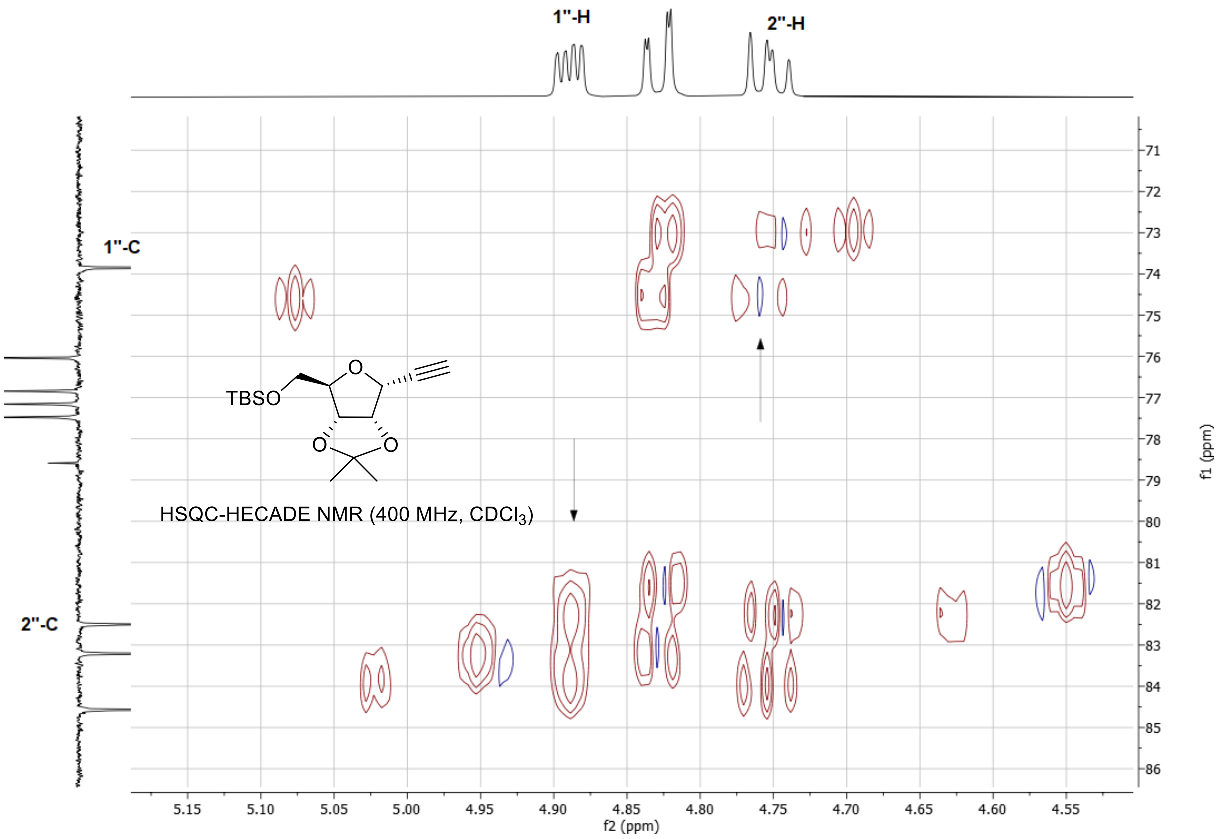
1''-H
2''-H
1''-C
2''-C
TBSO
HSQC-HECADE NMR (400 MHz, CDCl3)
f2 (ppm)
f1 (ppm)

Compound **29**

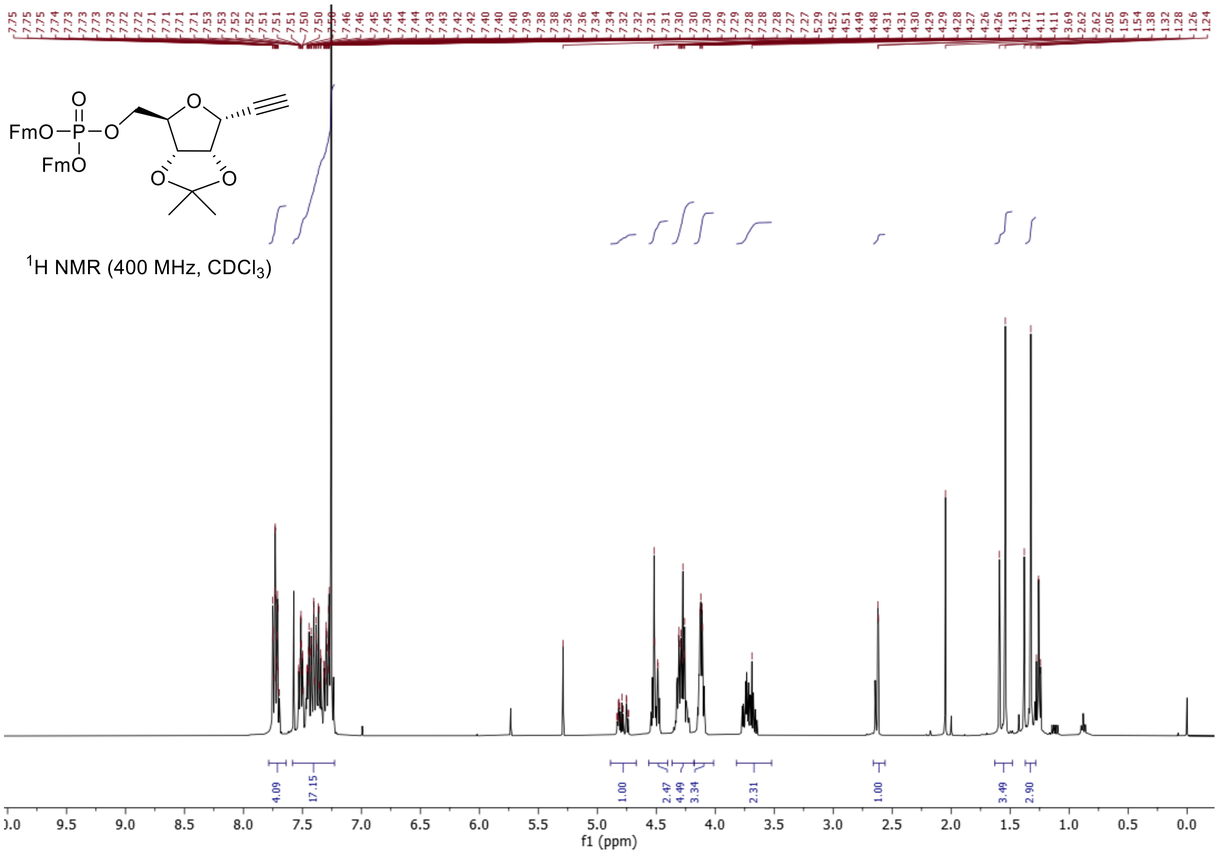
FmO
FmO
1H NMR (400 MHz, CDCl3)
f1 (ppm)

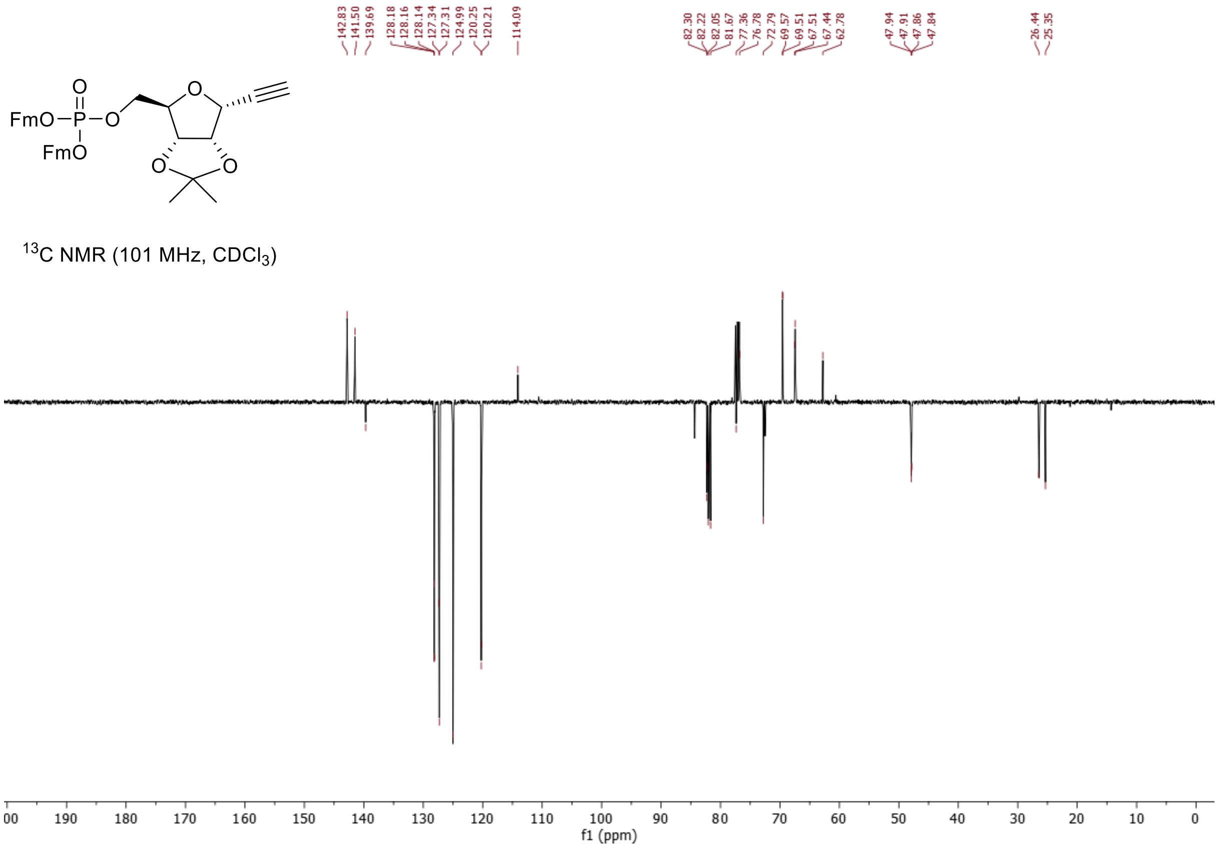

^{13}C NMR (101 MHz, $CDCl_3$)

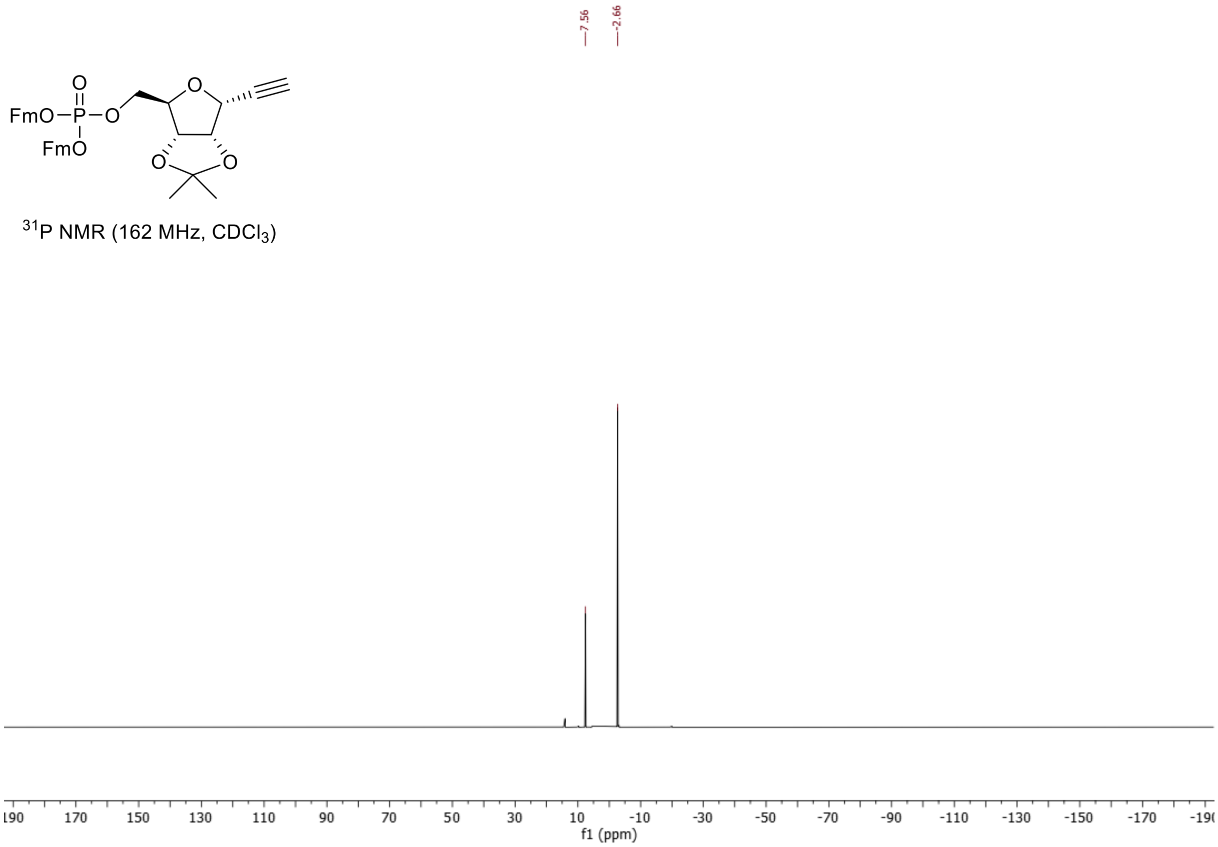

^{31}P NMR (162 MHz, $CDCl_3$)

Compound **5**

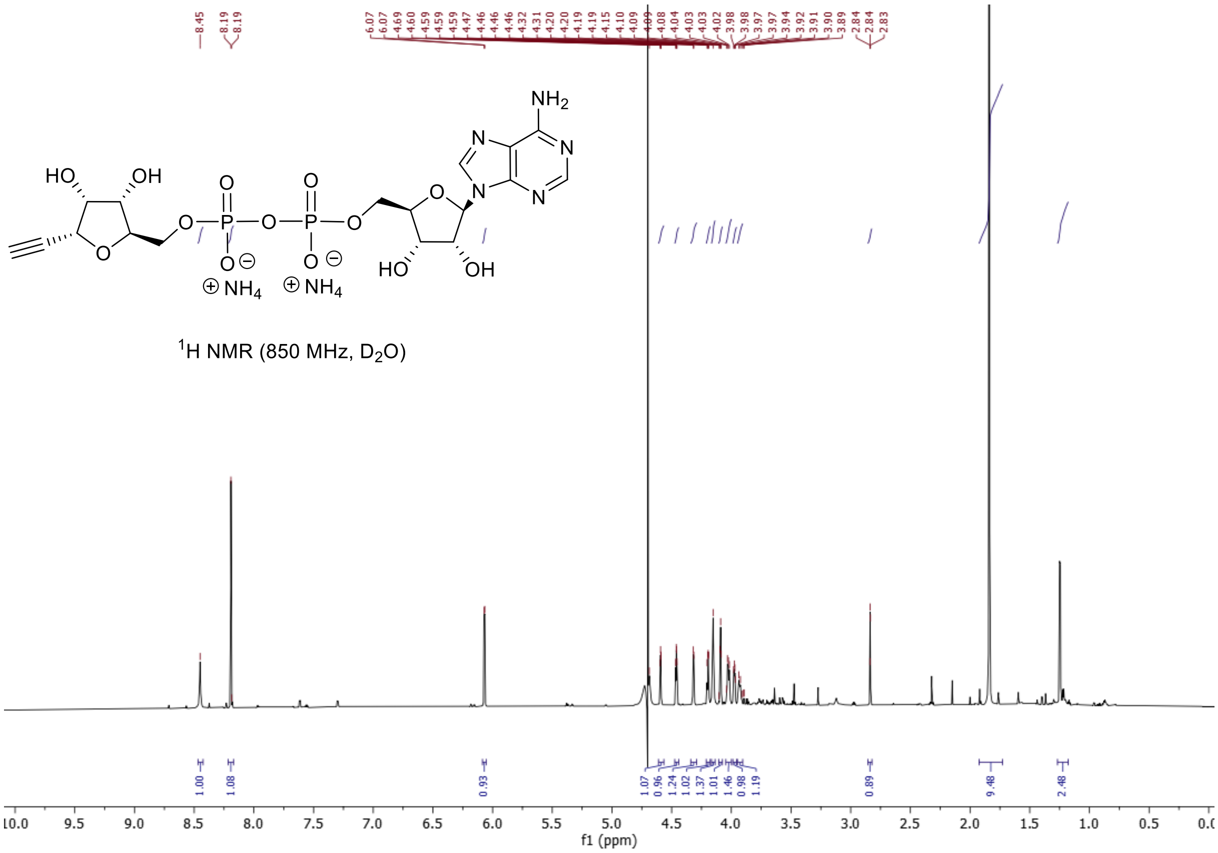

NH2
HO
OH
O
N
NH4
1H NMR (850 MHz, D2O)
f1 (ppm)

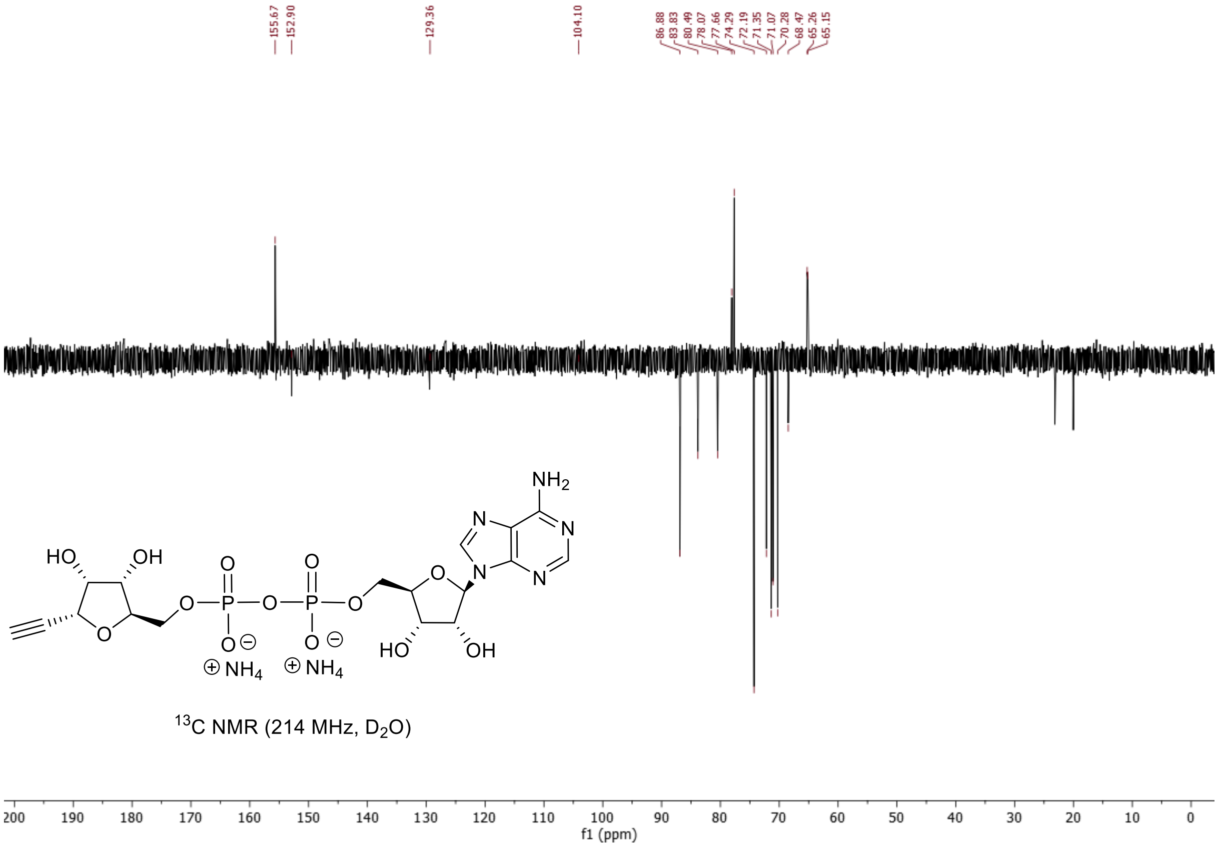

155.67
152.90
129.36
104.10
86.88
83.83
80.49
78.07
77.66
74.29
72.19
71.35
71.07
70.28
68.47
65.26
65.15
NH2
HO
OH
O
N
NH4
13C NMR (214 MHz, D2O)
f1 (ppm)

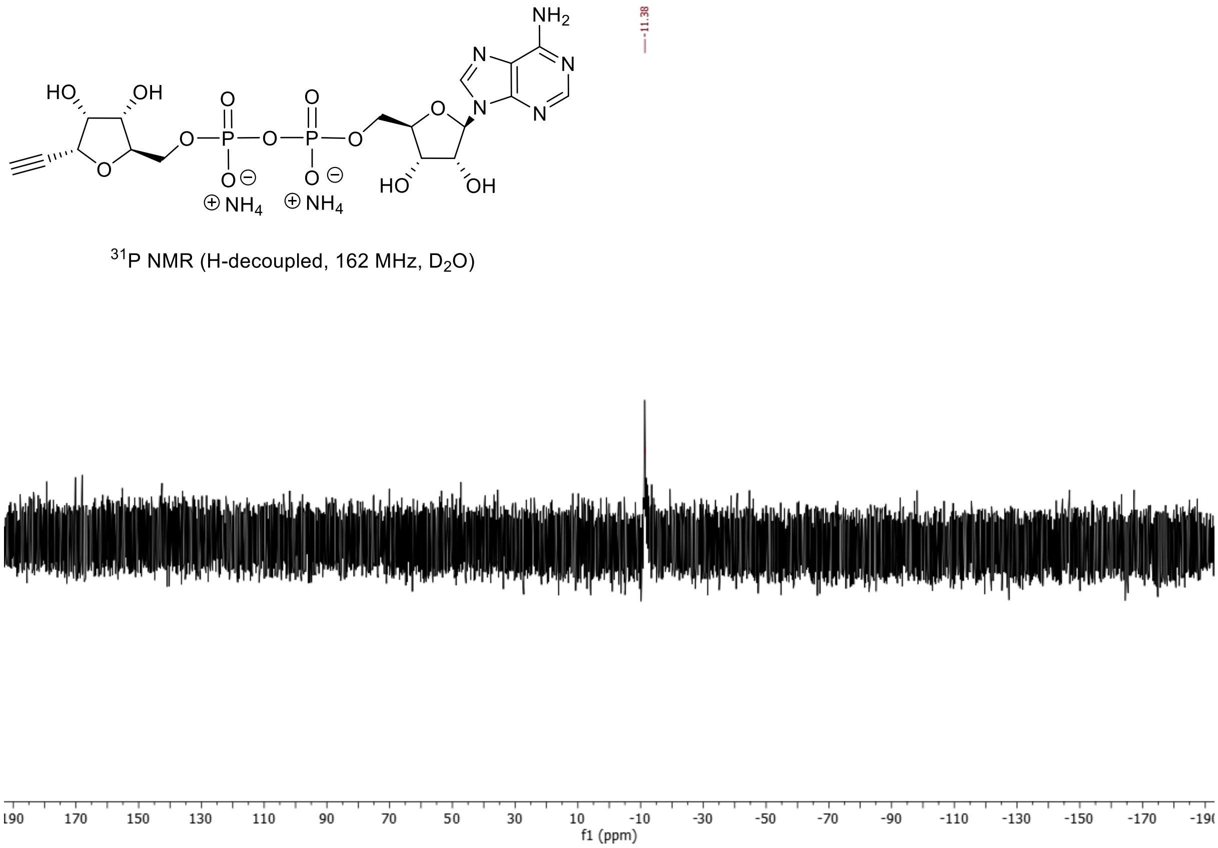
NH2
HO
OH
O
O
N
N
N
N
O
HO
OH
⊕ NH4
⊕ NH4
31P NMR (H-decoupled, 162 MHz, D2O)
-11.38
f1 (ppm)

Compound **33**

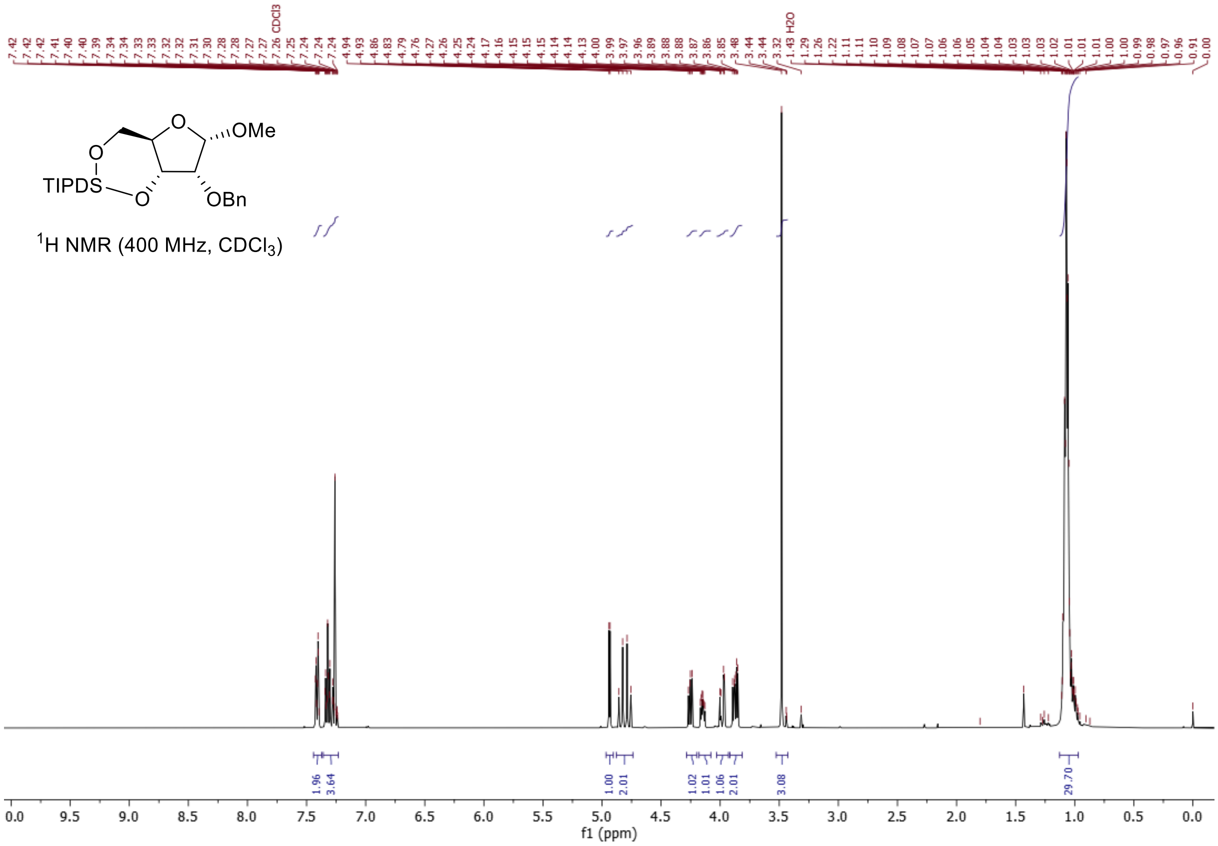
OMe
TIPDS
OBn
1H NMR (400 MHz, CDCl3)
CDCl3
H2O
1.96
3.64
1.00
2.01
1.02
1.01
1.06
2.01
3.08
29.70
f1 (ppm)

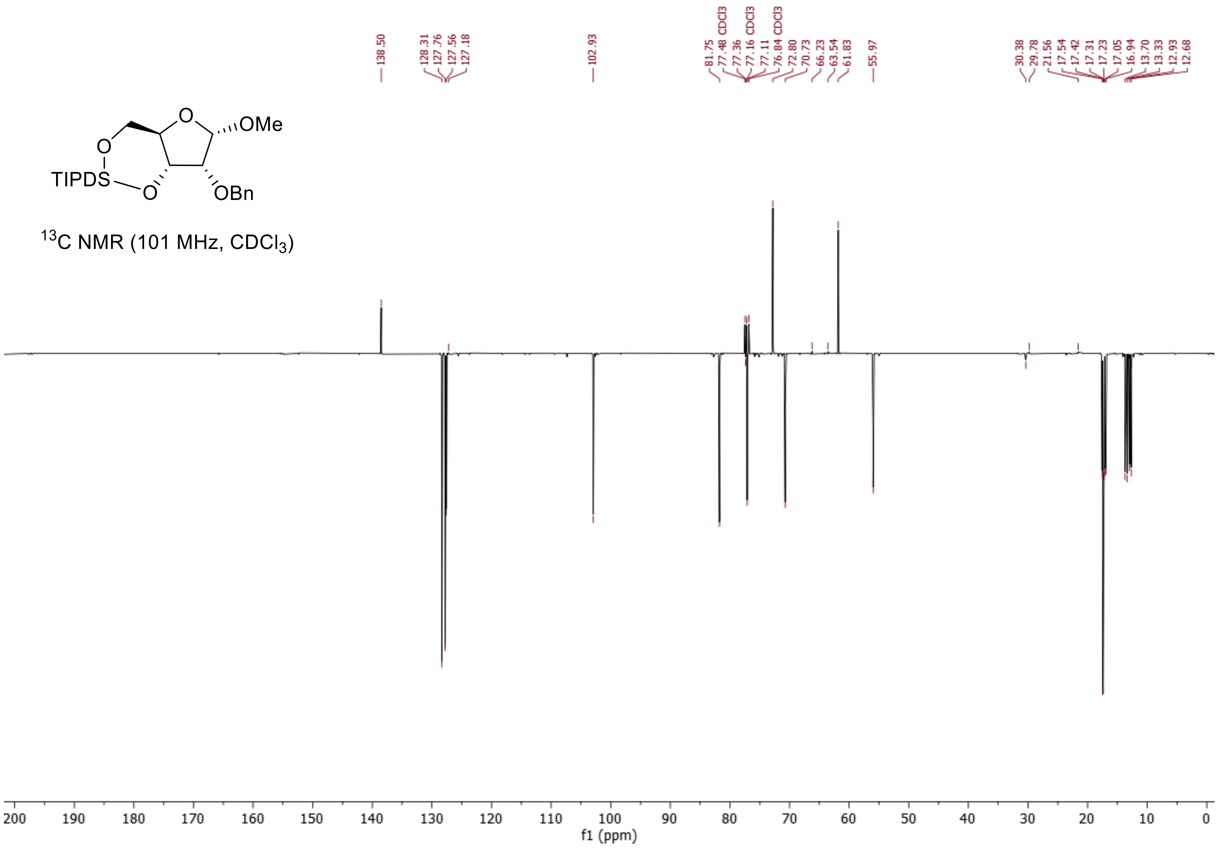
OMe
O
TIPDS
O
OBn
13C NMR (101 MHz, CDCl3)
138.50
128.31
127.76
127.56
127.18
102.93
81.75
77.48 CDCl3
77.36
77.16 CDCl3
77.11
76.84 CDCl3
72.80
70.73
66.23
63.54
61.83
55.97
30.38
29.78
21.56
17.54
17.42
17.31
17.23
17.05
16.94
13.70
13.33
12.93
12.68
f1 (ppm)

Compound **34**

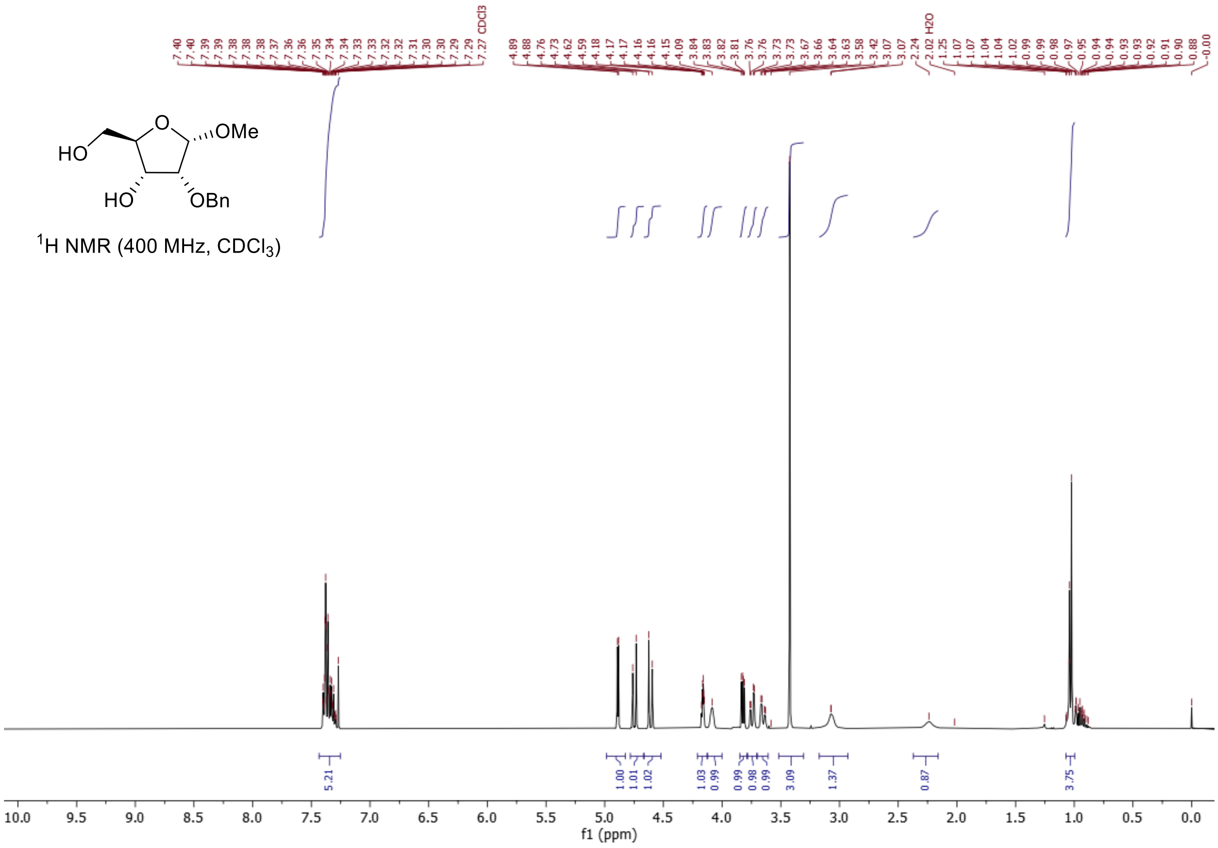
HO
O
OMe
HO
OBn
1H NMR (400 MHz, CDCl3)
7.27 CDCl3
2.02 H2O
5.21
1.00
1.01
1.02
1.03
0.99
0.99
0.98
0.99
3.09
1.37
0.87
3.75
f1 (ppm)

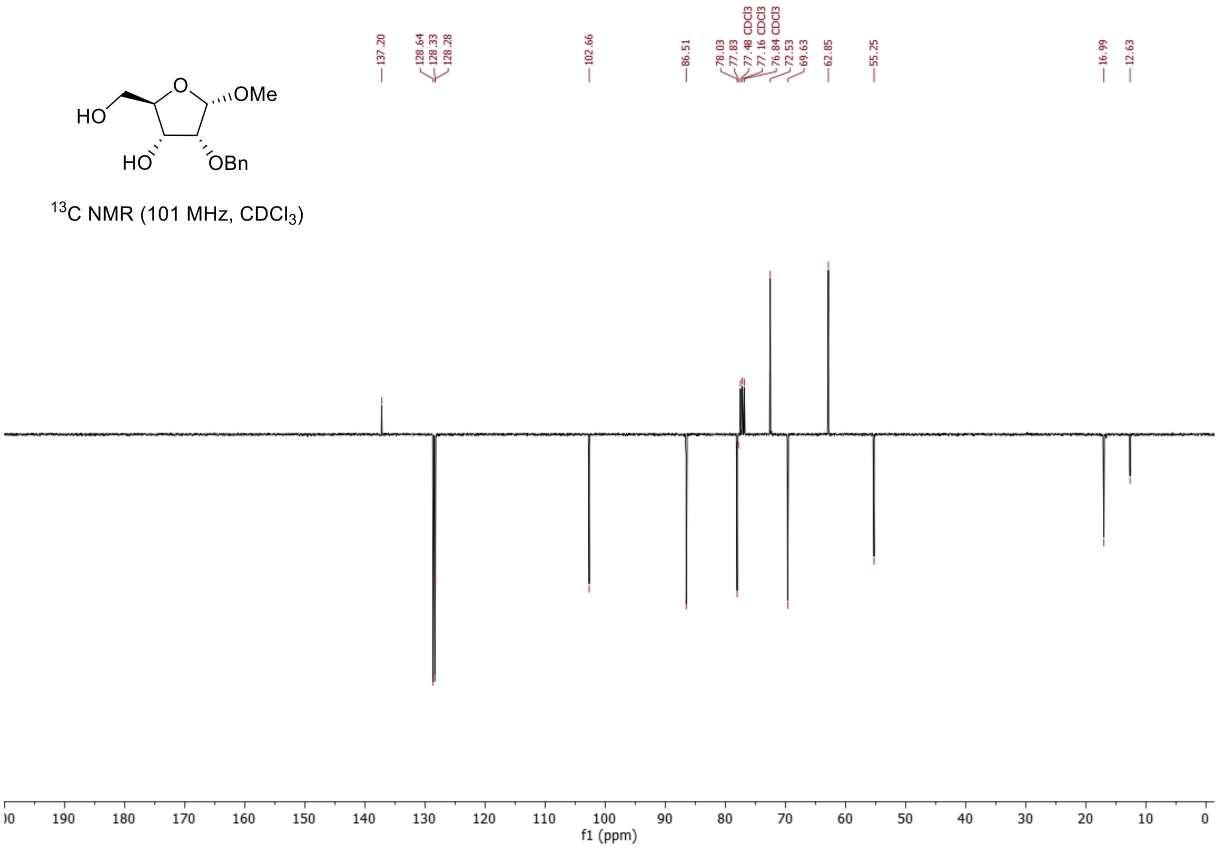
HO
O
OMe
HO
OBn
^{13}C NMR (101 MHz, $CDCl_3$)
137.20
128.64
128.33
128.28
102.66
86.51
78.03
77.83
77.48 CDCl3
77.16 CDCl3
76.84 CDCl3
72.53
69.63
62.85
55.25
16.99
12.63
f1 (ppm)

Compound **35**

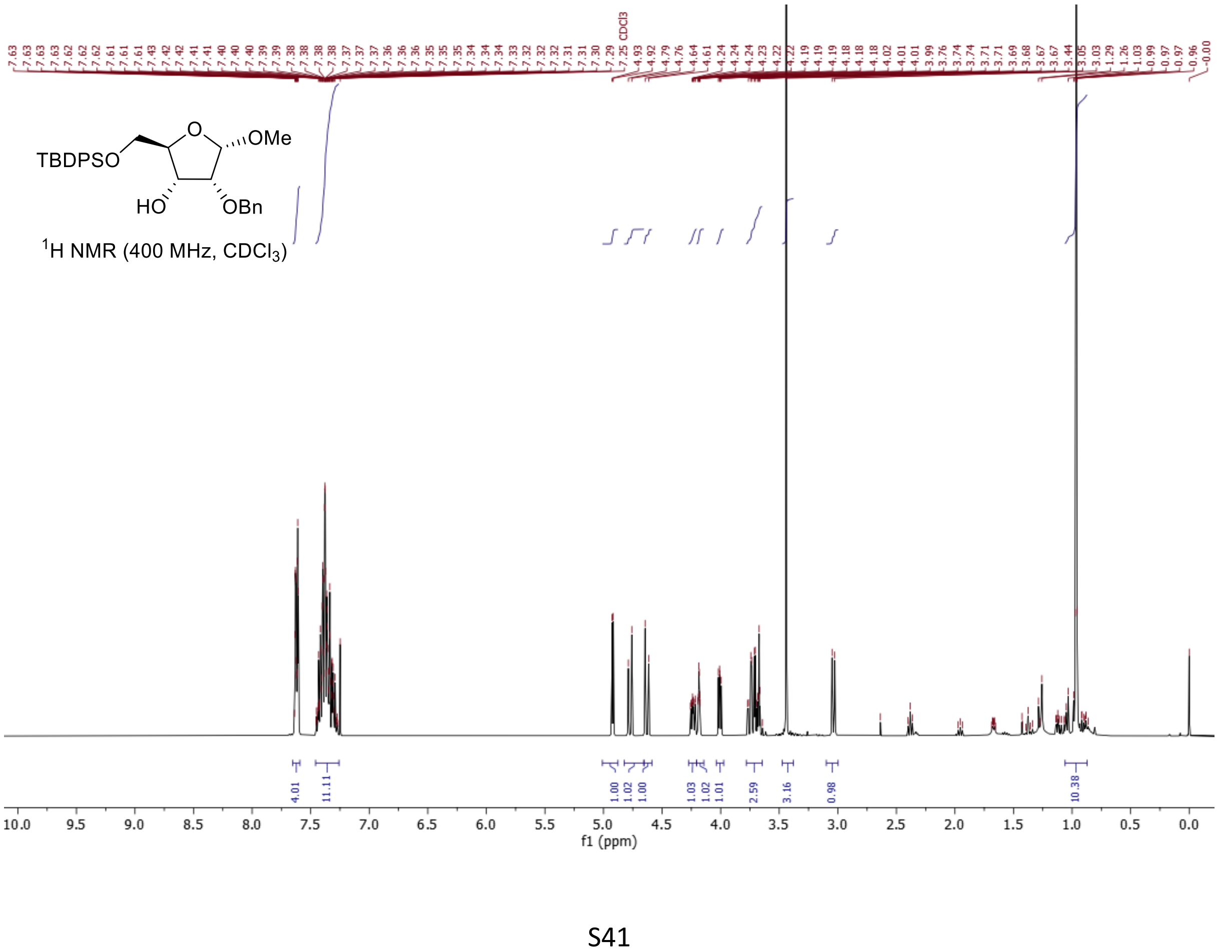
TBDPSO
O
OMe
HO
OBn
^{1}H NMR (400 MHz, $CDCl_3$)
CDCl3
4.01
11.11
1.00
1.02
1.00
1.03
1.02
1.01
2.59
3.16
0.98
10.38
f1 (ppm)

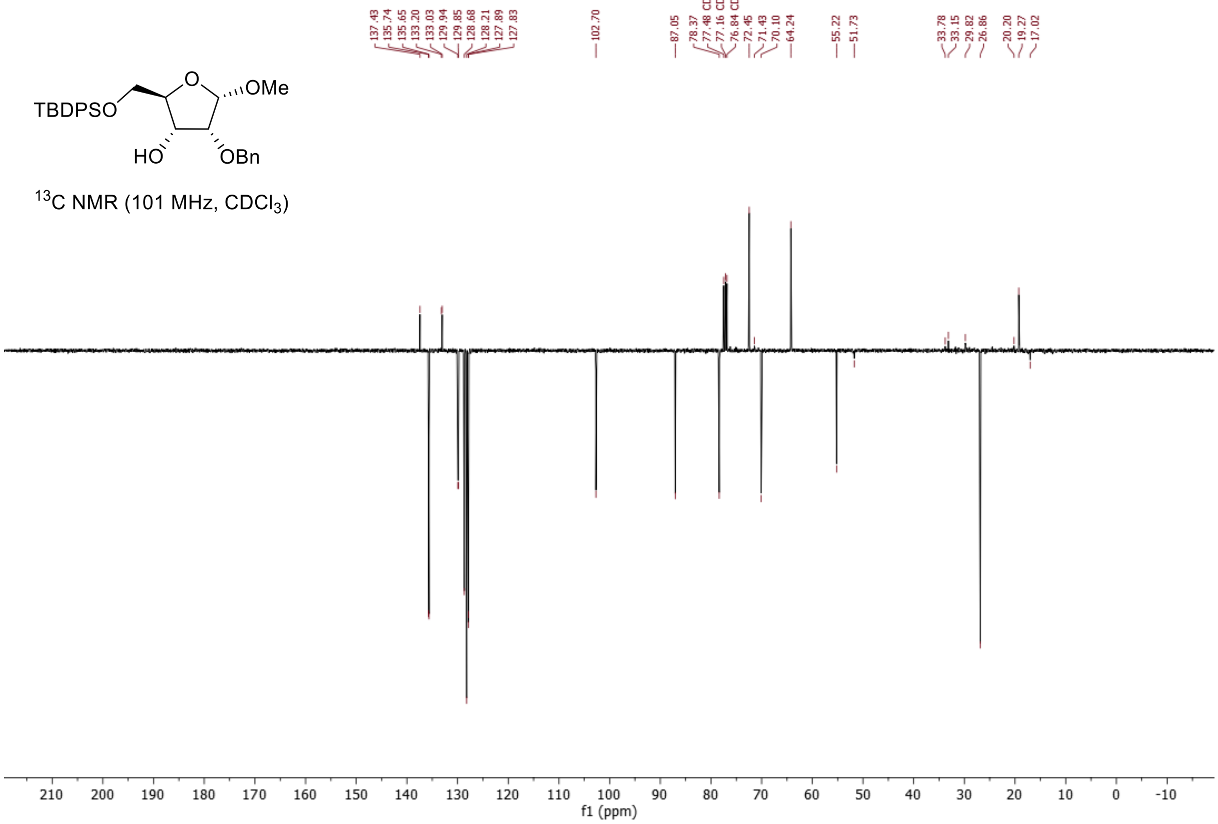
TBDPSO
OMe
HO
OBn
13C NMR (101 MHz, CDCl3)
f1 (ppm)

Compound **36**

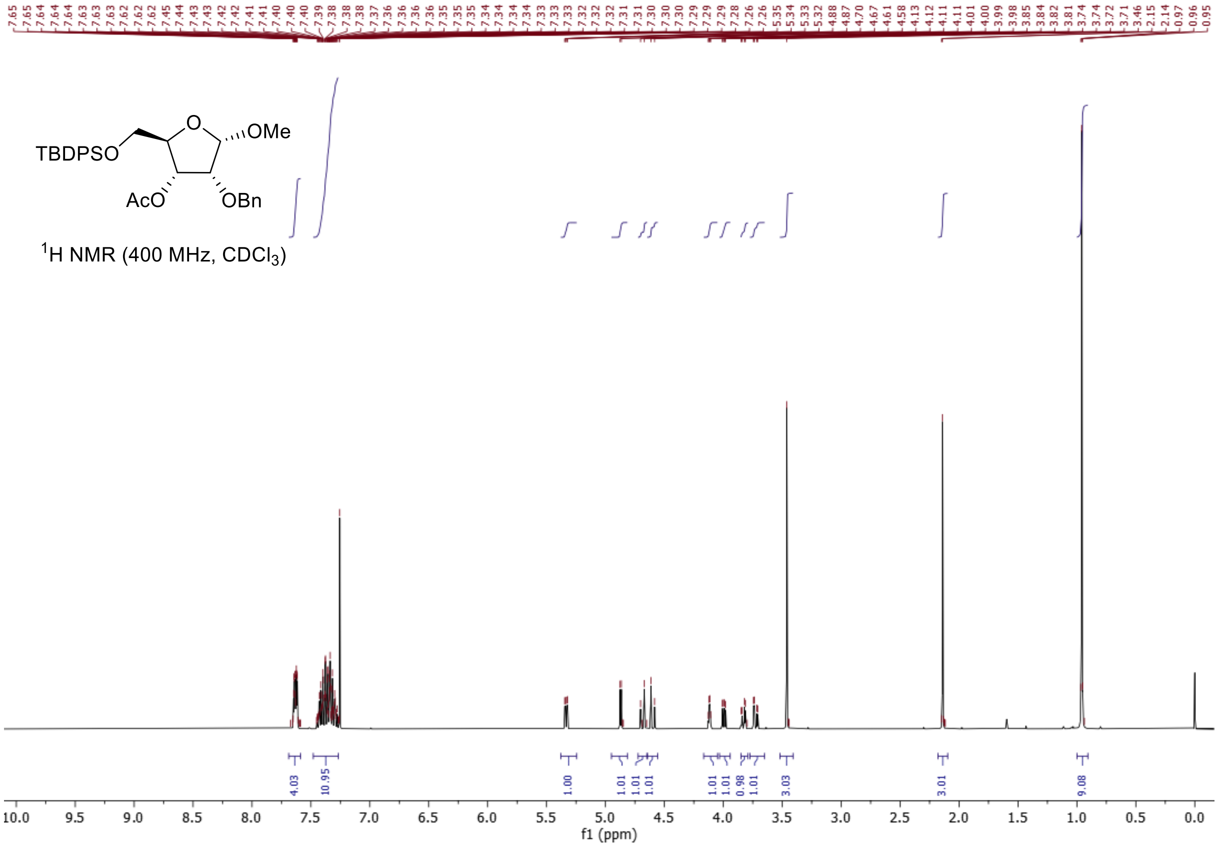
TBDPSO
OMe
AcO
OBn
1H NMR (400 MHz, CDCl3)
f1 (ppm)

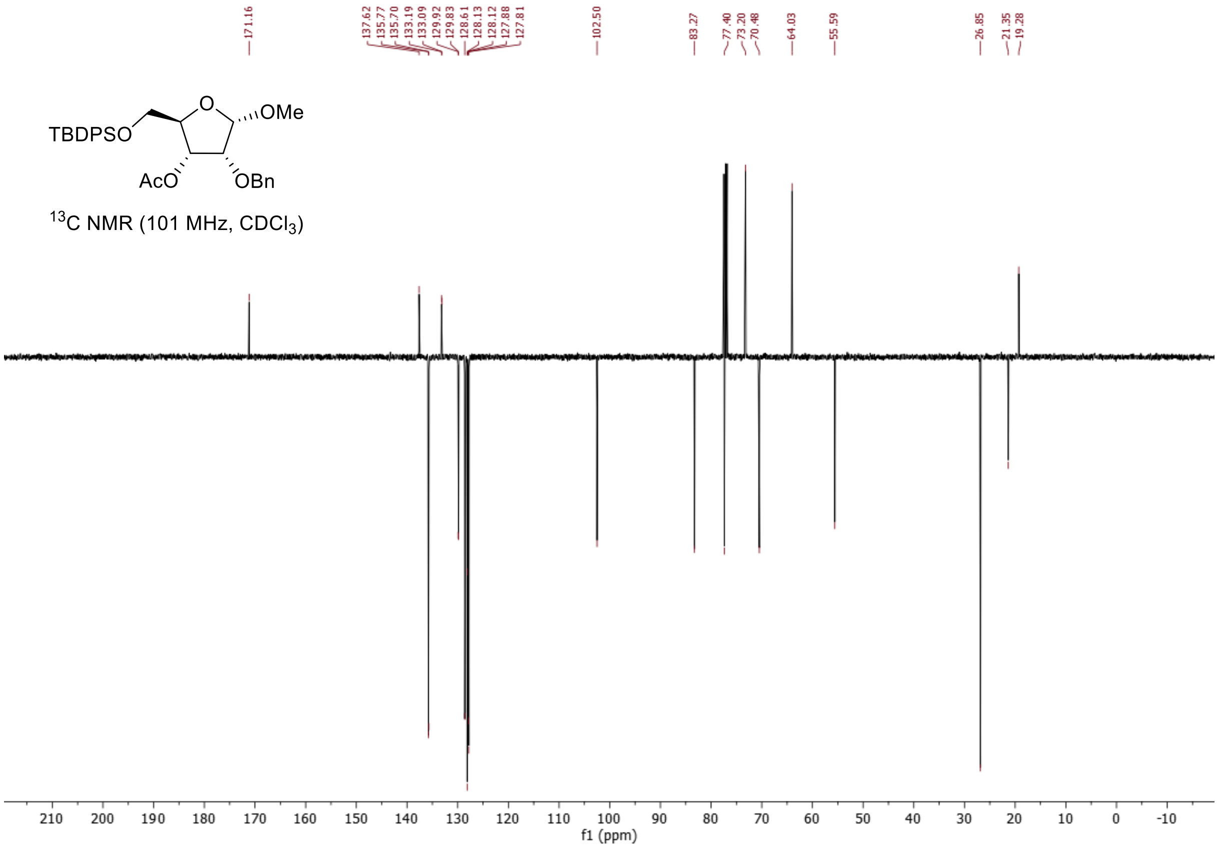
TBDPSO
OMe
AcO
OBn
13C NMR (101 MHz, CDCl3)
171.16
137.62
135.77
135.70
133.19
133.09
129.92
129.83
128.61
128.13
128.12
127.88
127.81
102.50
83.27
77.40
73.20
70.48
64.03
55.59
26.85
21.35
19.28
f1 (ppm)

Compound **37**

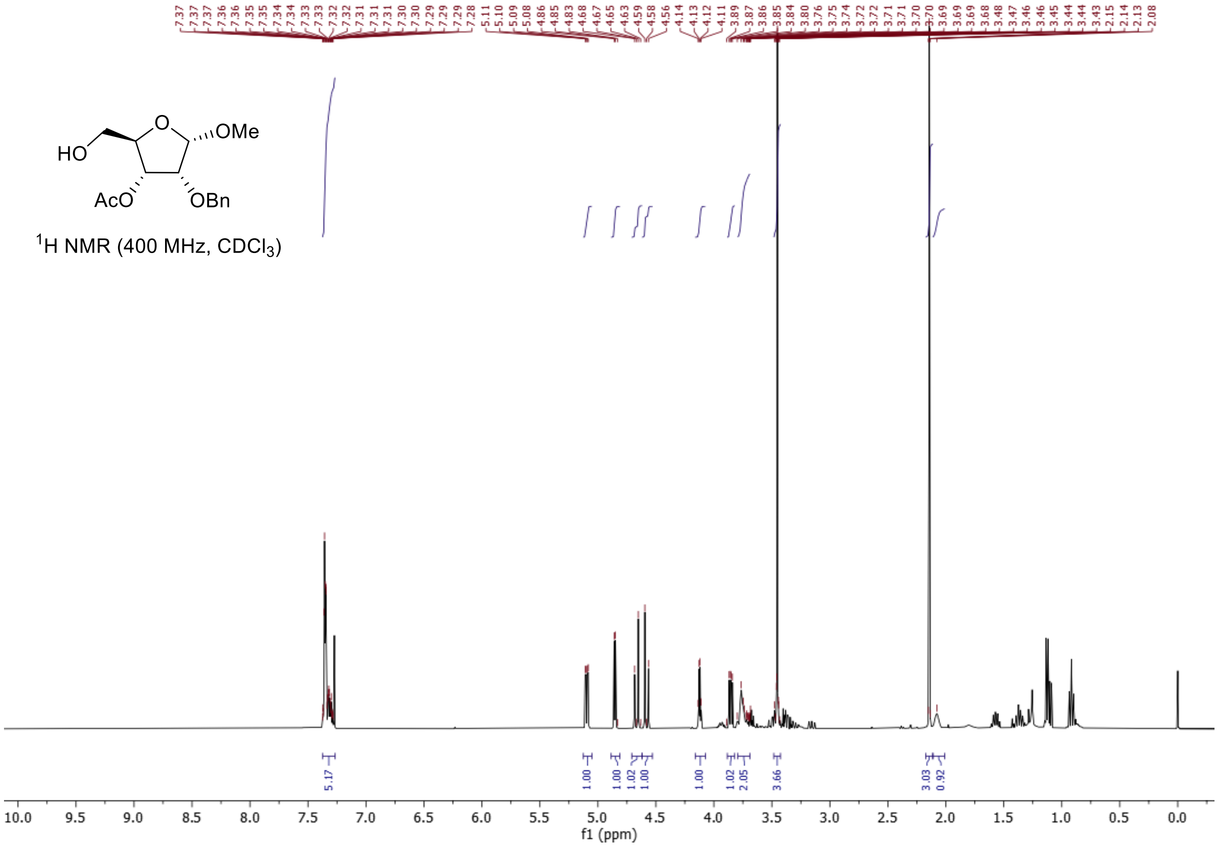
HO
OMe
AcO
OBn
1H NMR (400 MHz, CDCl3)
5.17
1.00
1.00
1.02
1.00
1.00
1.02
2.05
3.66
3.03
0.92
f1 (ppm)

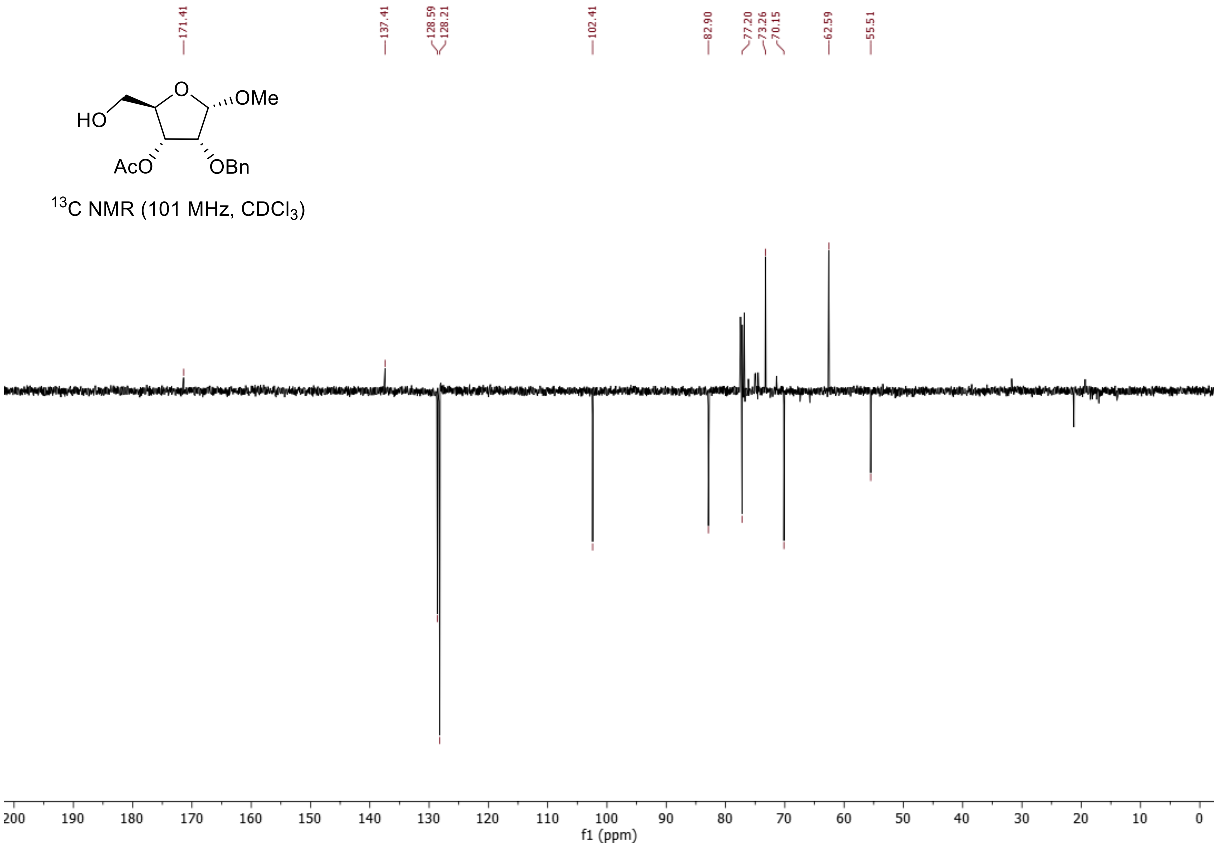
171.41
137.41
128.59
128.21
102.41
82.90
77.20
73.26
70.15
62.59
55.51
HO
O
OMe
AcO
OBn
^{13}C NMR (101 MHz, CDCl$_3$)
f1 (ppm)

Compound **38**

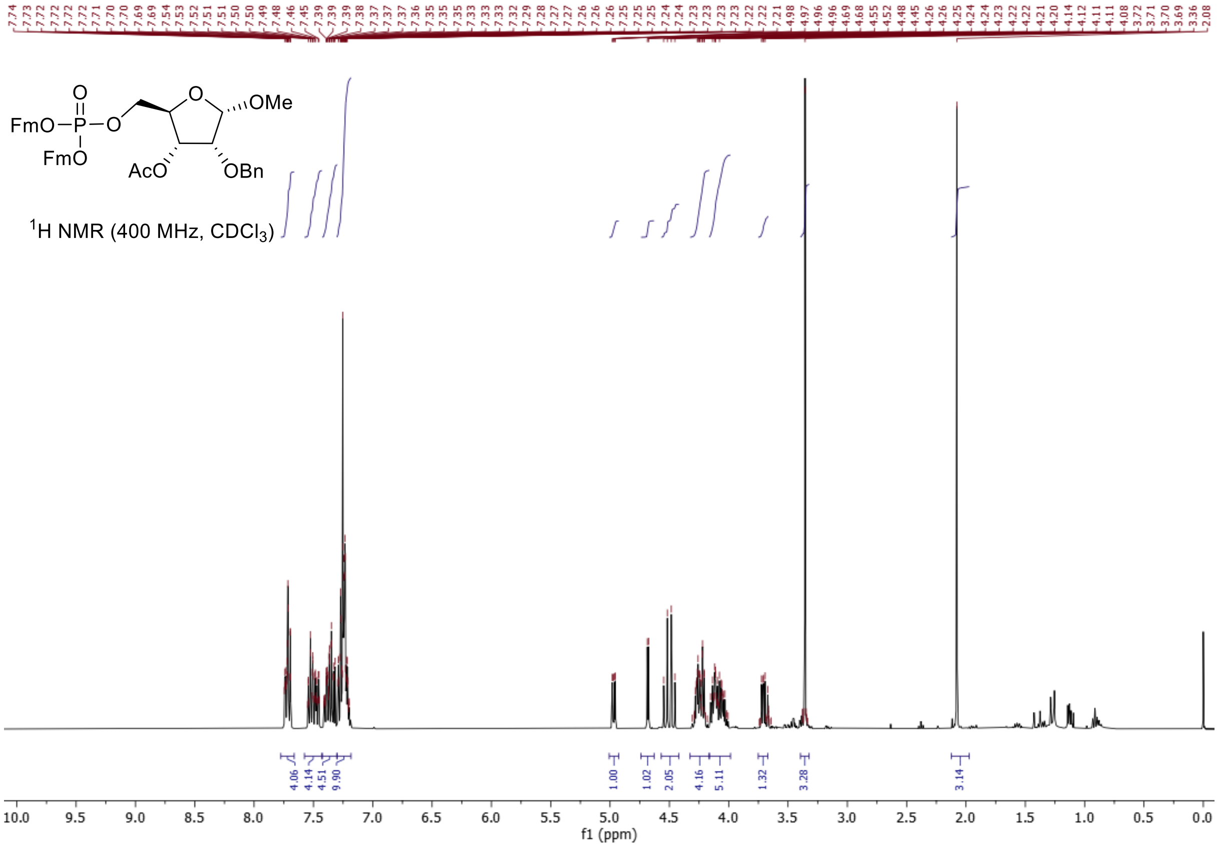
O
FmO
P
O
FmO
OMe
AcO
OBn
^{1}H NMR (400 MHz, CDCl$_3$)
4.06
4.14
4.51
9.90
1.00
1.02
2.05
4.16
5.11
1.32
3.28
3.14
f1 (ppm)

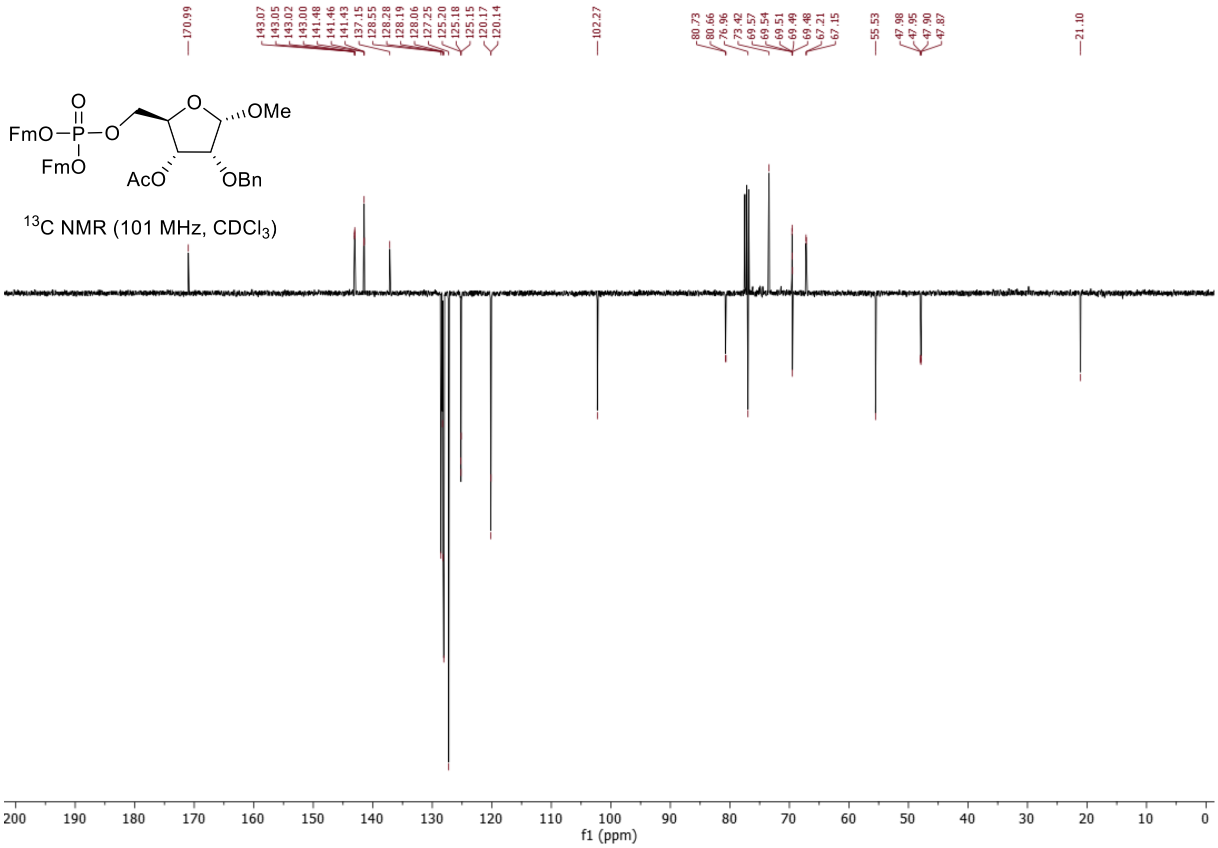
170.99
143.07
143.05
143.02
143.00
141.48
141.46
141.43
137.15
128.55
128.28
128.19
128.06
127.25
125.20
125.18
125.15
120.17
120.14
102.27
80.73
80.66
76.96
73.42
69.57
69.54
69.51
69.49
69.48
67.21
67.15
55.53
47.98
47.95
47.90
47.87
21.10
FmO
FmO
O
P
O
O
OMe
AcO
OBn
^{13}C NMR (101 MHz, $CDCl_3$)
f1 (ppm)

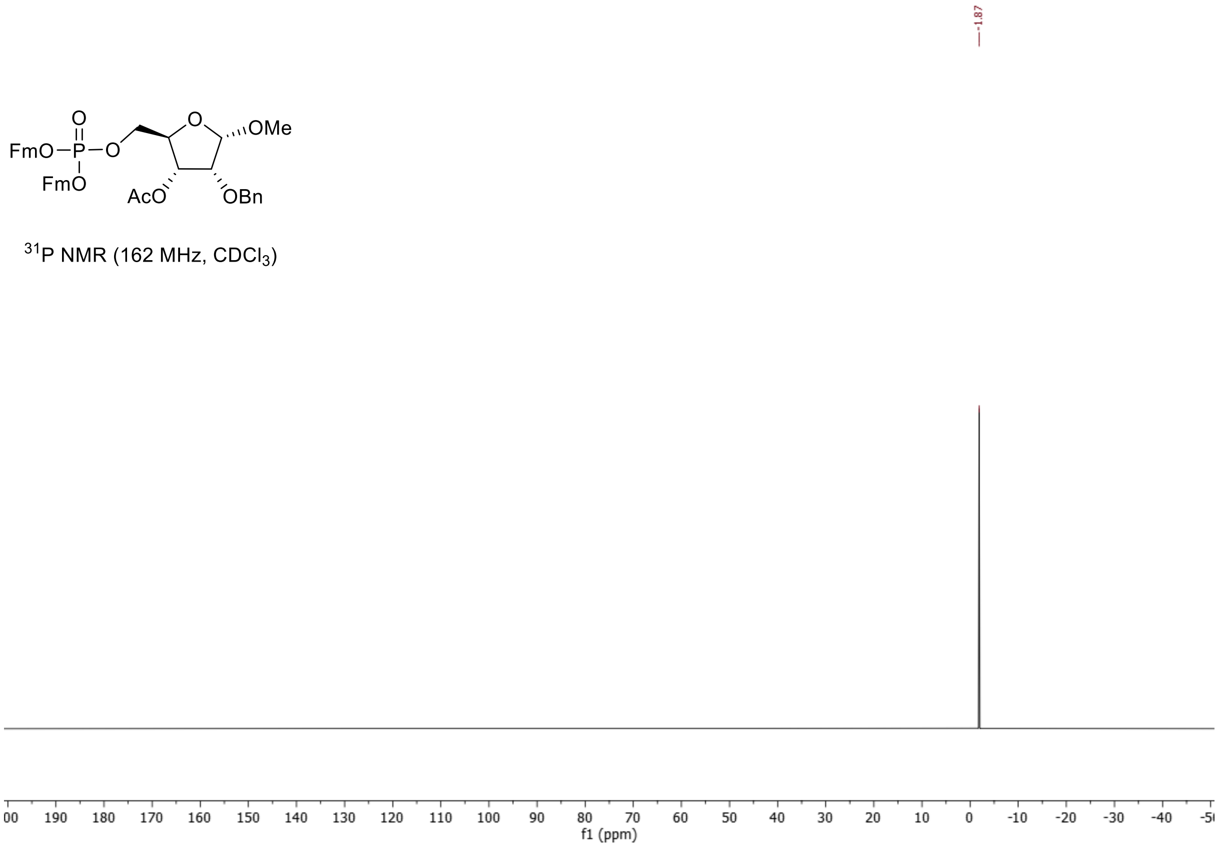
-1.87
FmO
FmO
O
P
O
O
OMe
AcO
OBn
^{31}P NMR (162 MHz, $CDCl_3$)
f1 (ppm)

Compound **6**

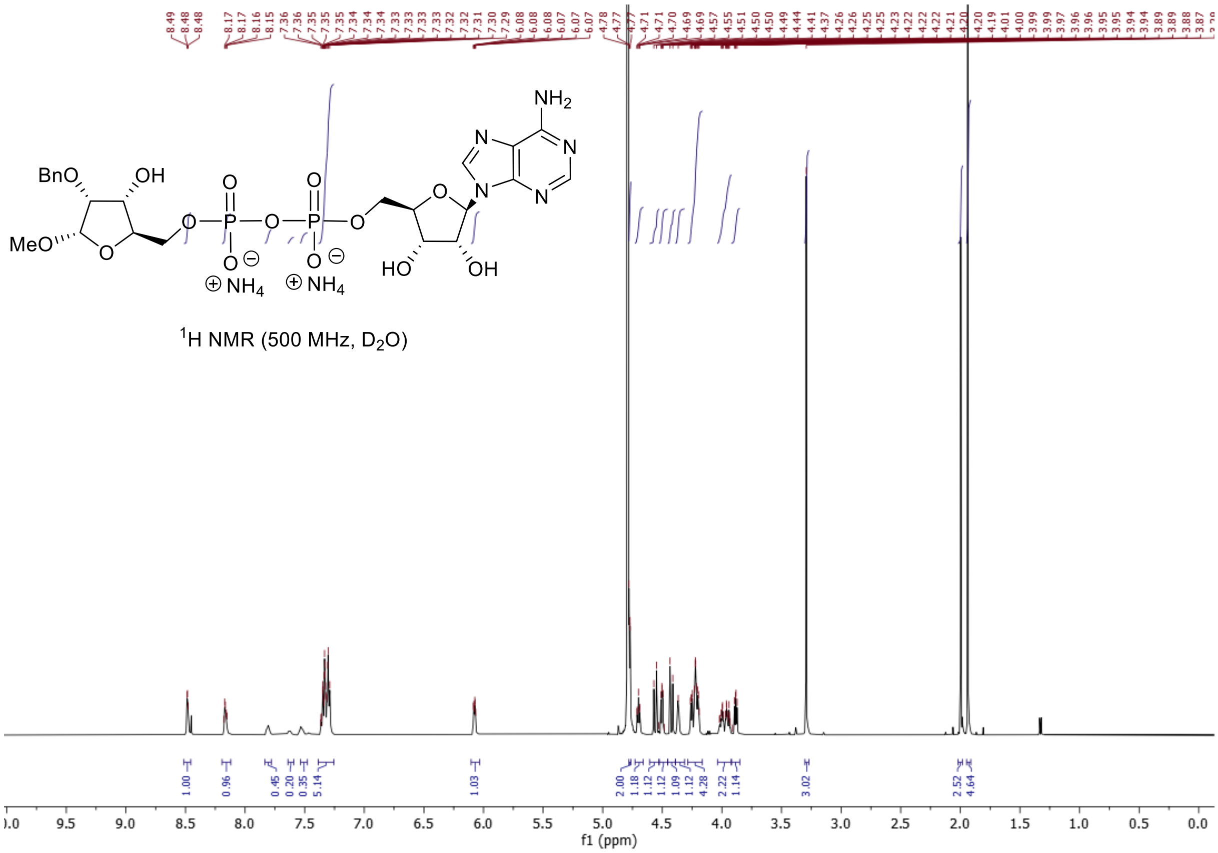

BnO
OH
MeO
O
P
NH2
N
HO
^{1}H NMR (500 MHz, D2O)
f1 (ppm)

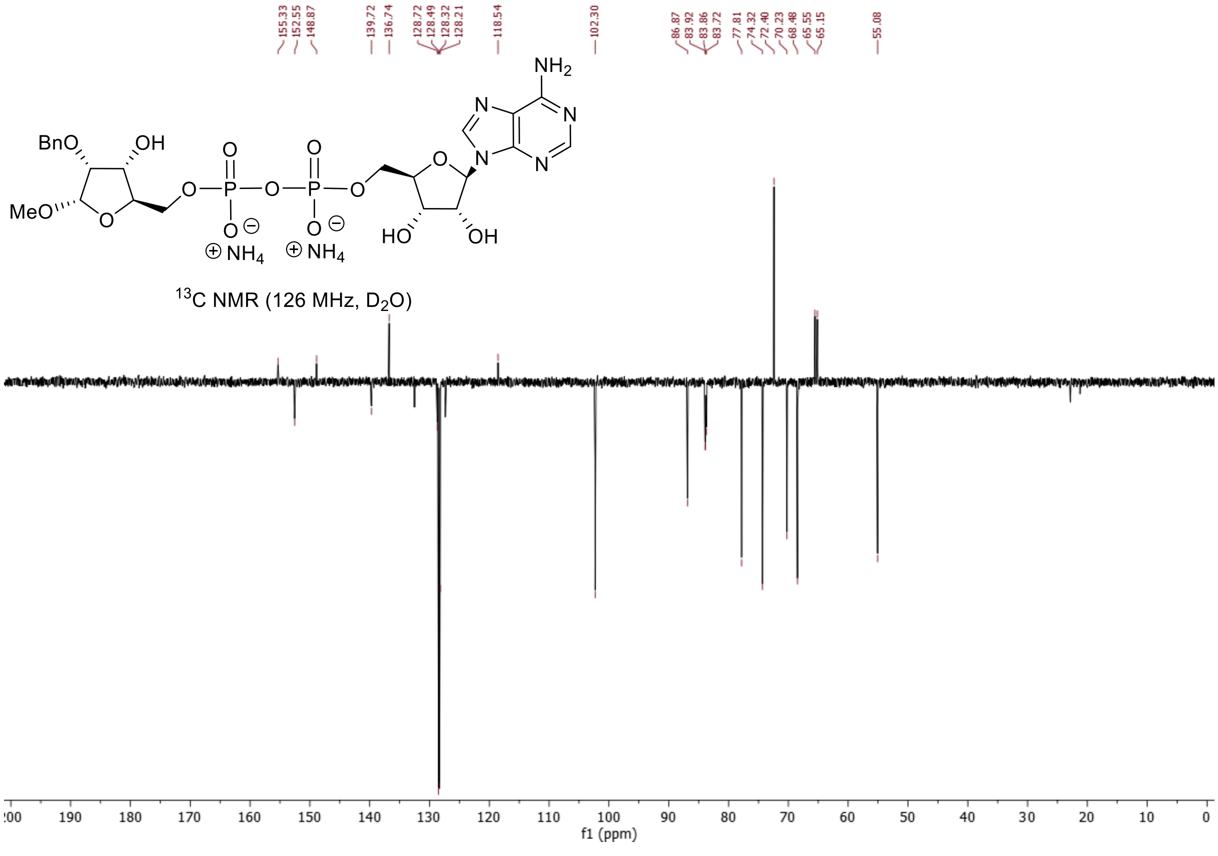

155.33
152.55
148.87
139.72
136.74
128.72
128.49
128.32
128.21
118.54
102.30
86.87
83.92
83.86
83.72
77.81
74.32
72.40
70.23
68.48
65.55
65.15
55.08
^{13}C NMR (126 MHz, D2O)
f1 (ppm)

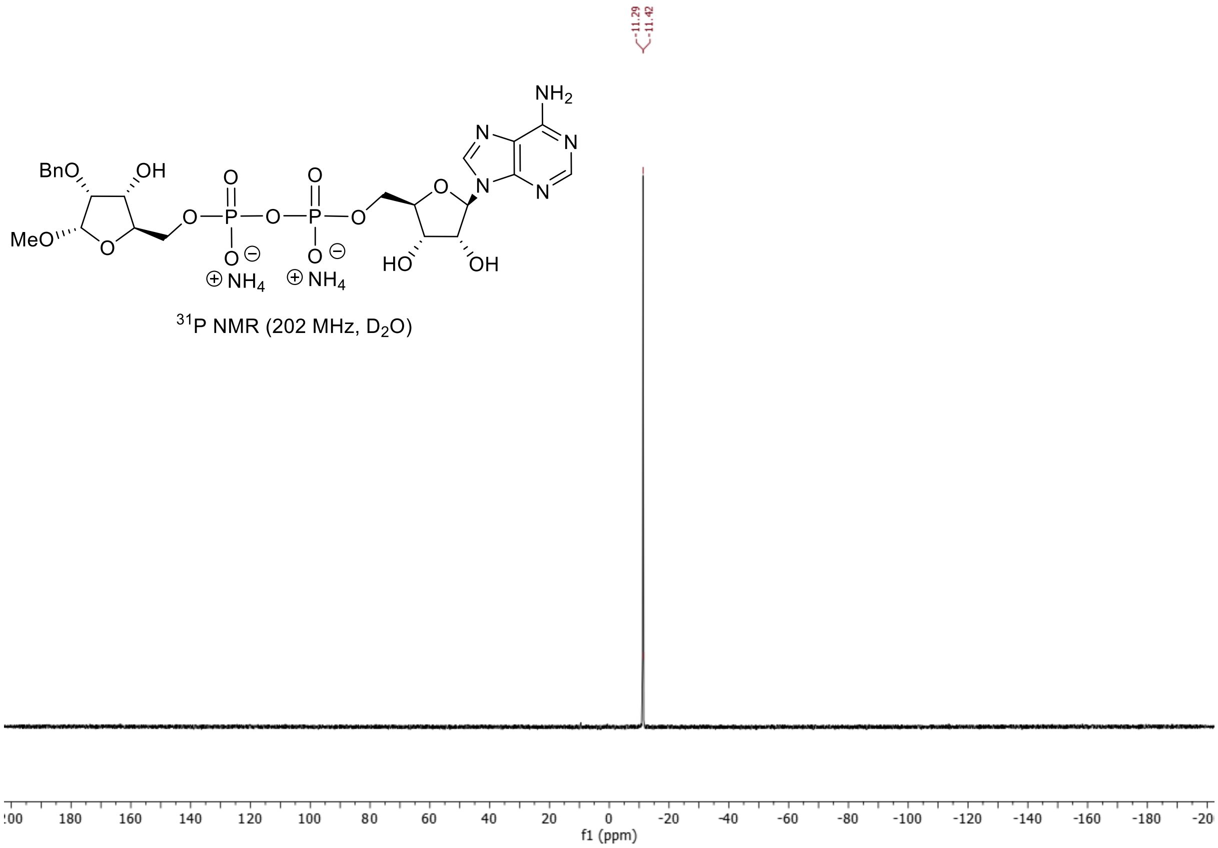
NH2
BnO
OH
MeO
^{31}P NMR (202 MHz, D$_2$O)
f1 (ppm)

Compound **41**

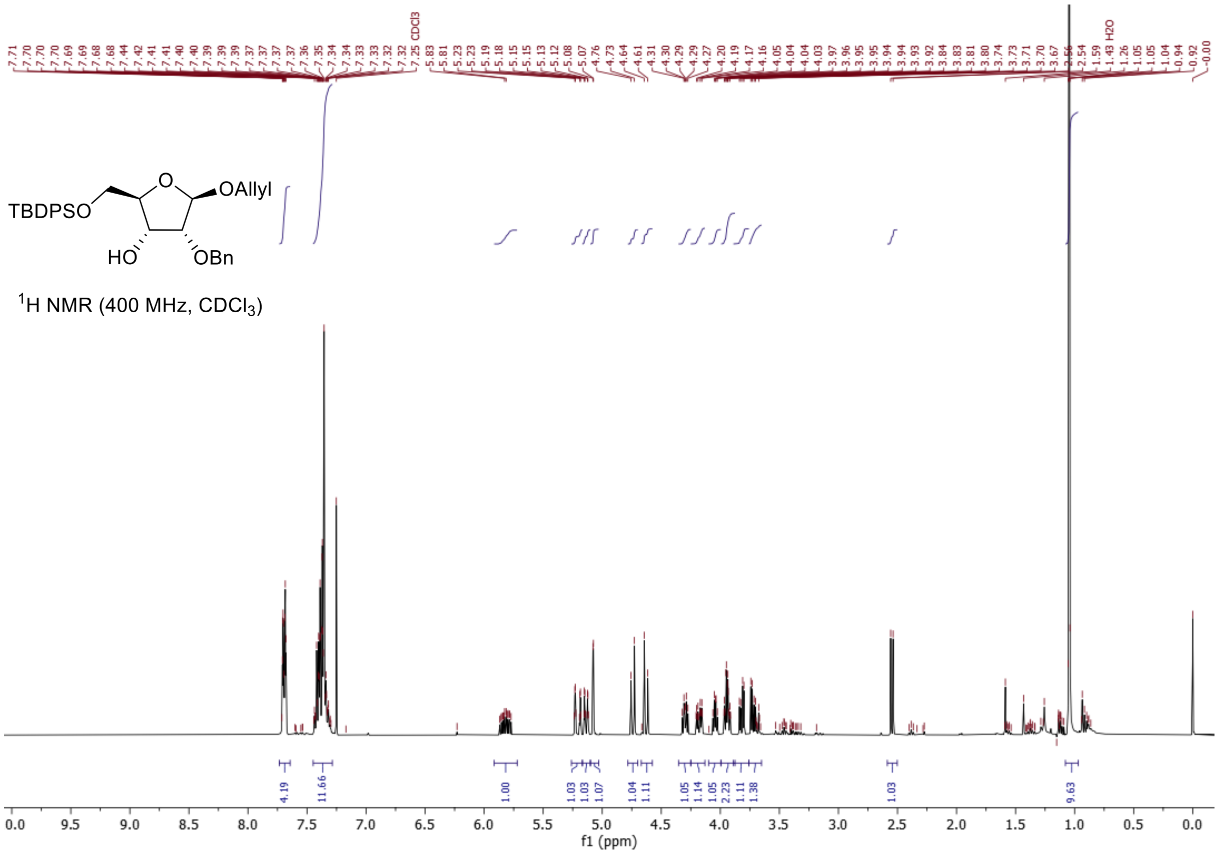
TBDPSO
OAllyl
HO
OBn
^{1}H NMR (400 MHz, CDCl$_3$)
f1 (ppm)

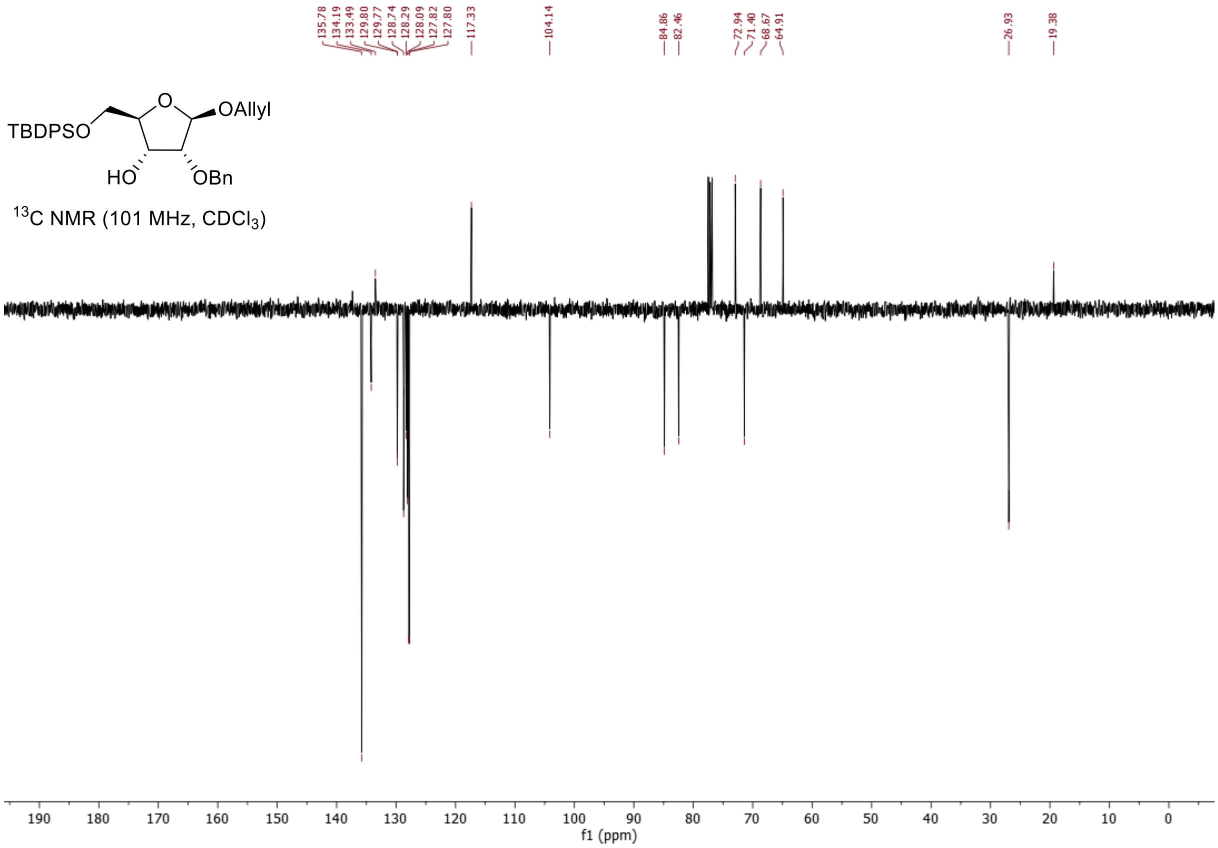

TBDPSO
OAllyl
HO
OBn
^{13}C NMR (101 MHz, CDCl$_3$)
f1 (ppm)

Compound **42**

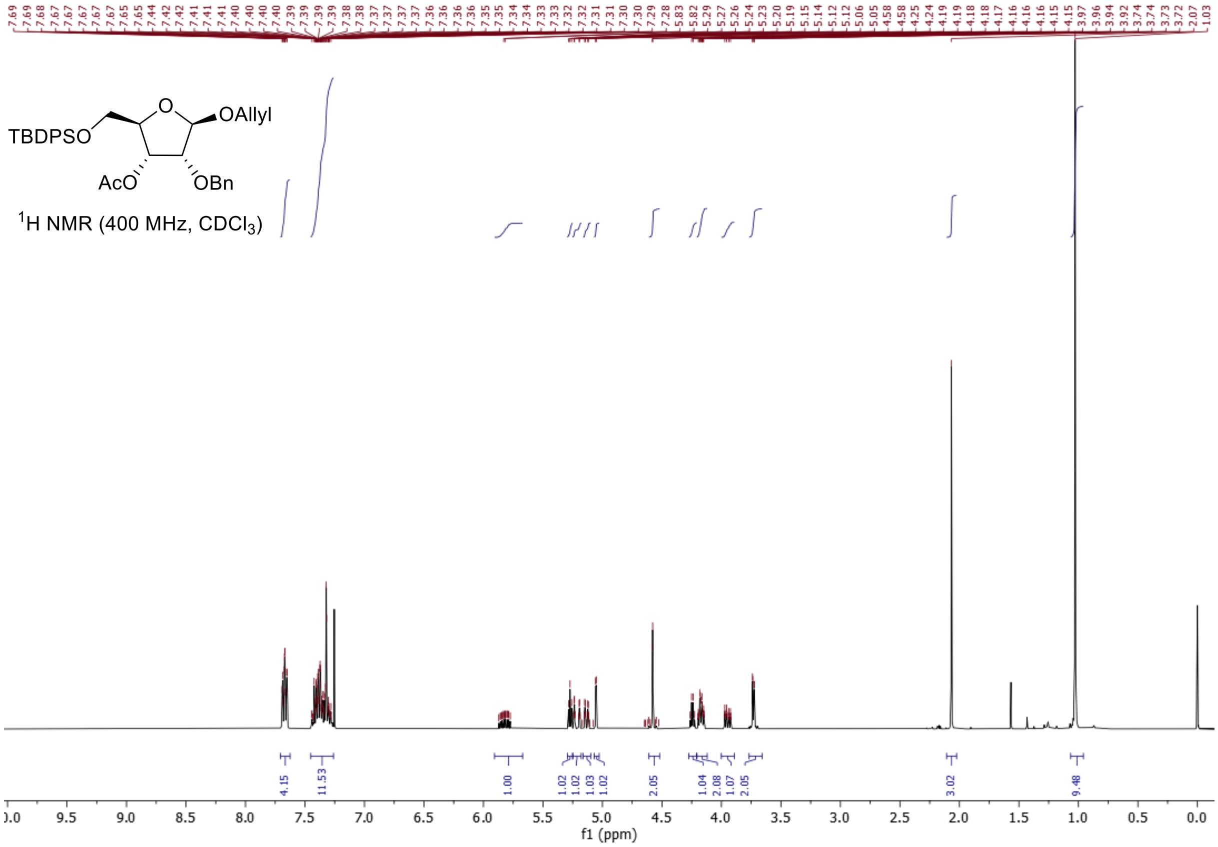

TBDPSO
OAllyl
AcO
OBn
^{1}H NMR (400 MHz, CDCl$_3$)
f1 (ppm)

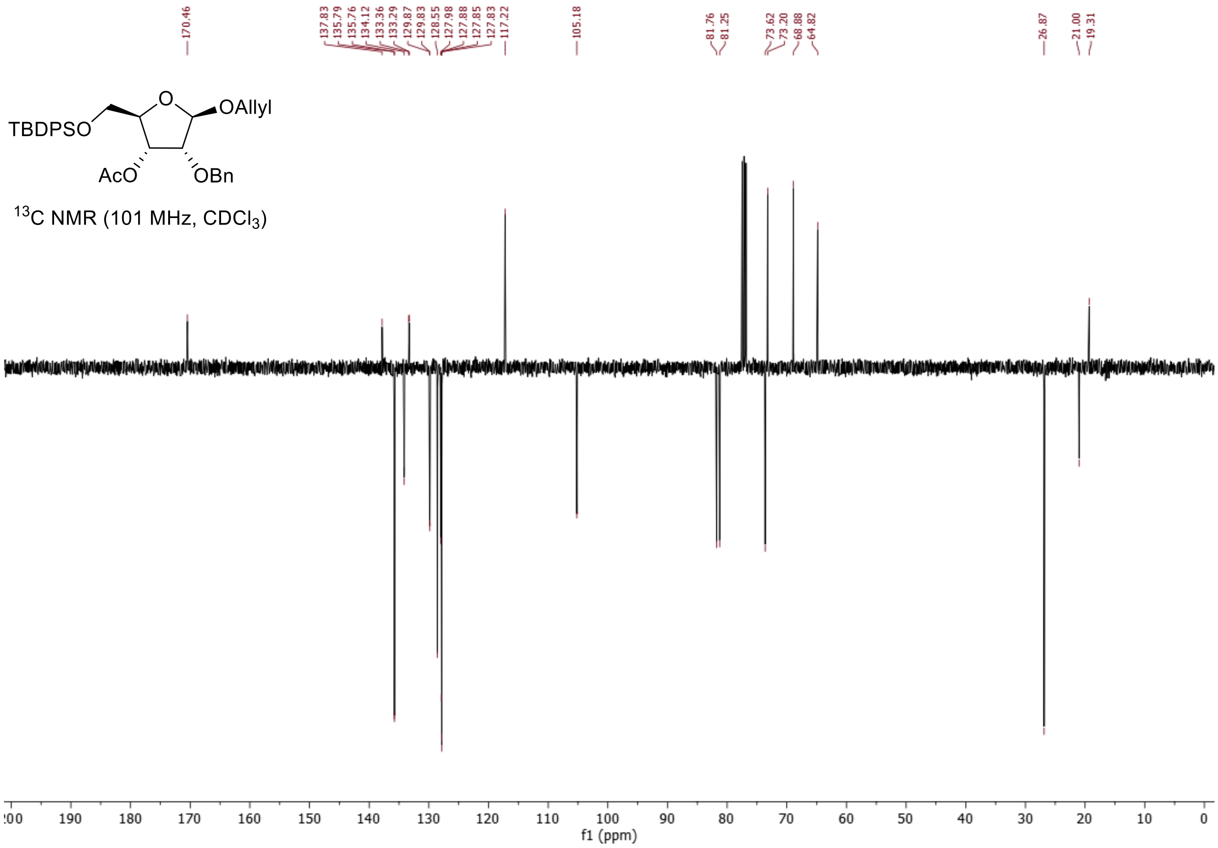

Compound **43**

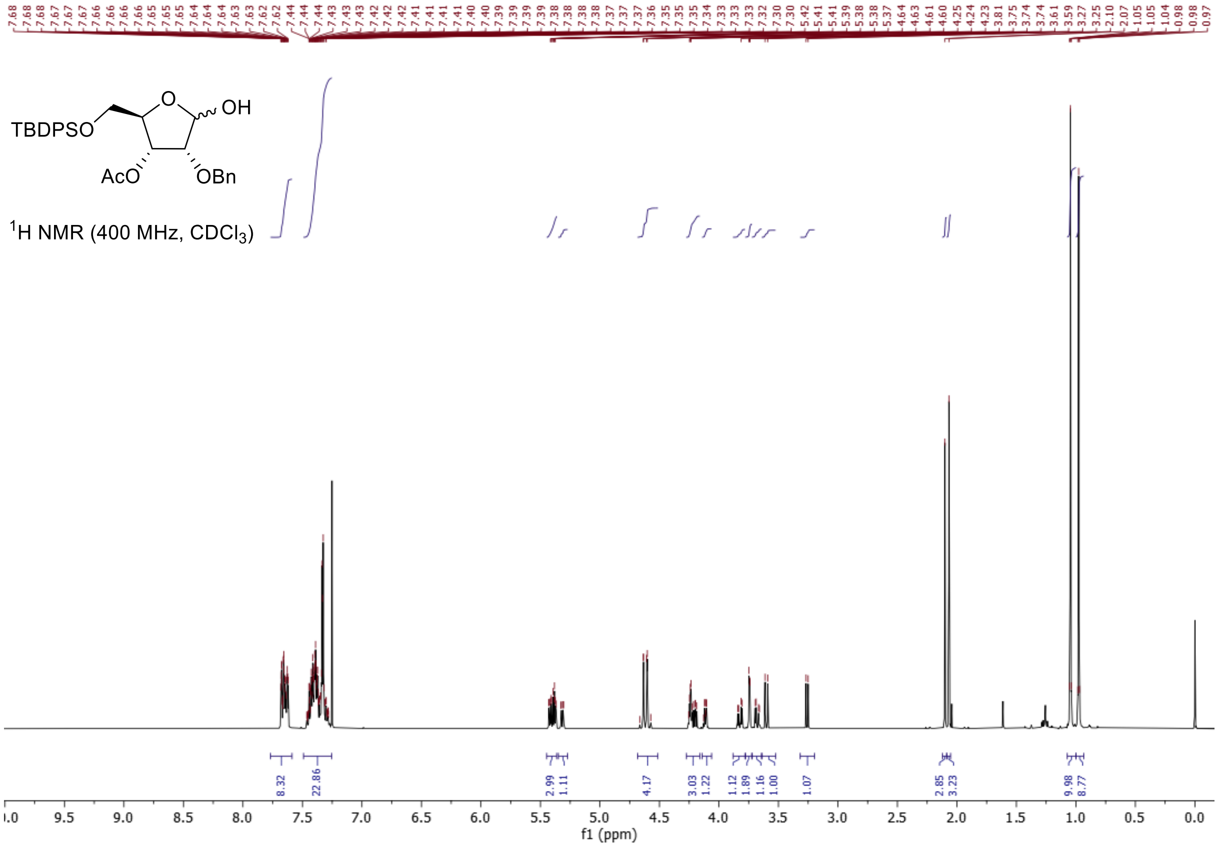

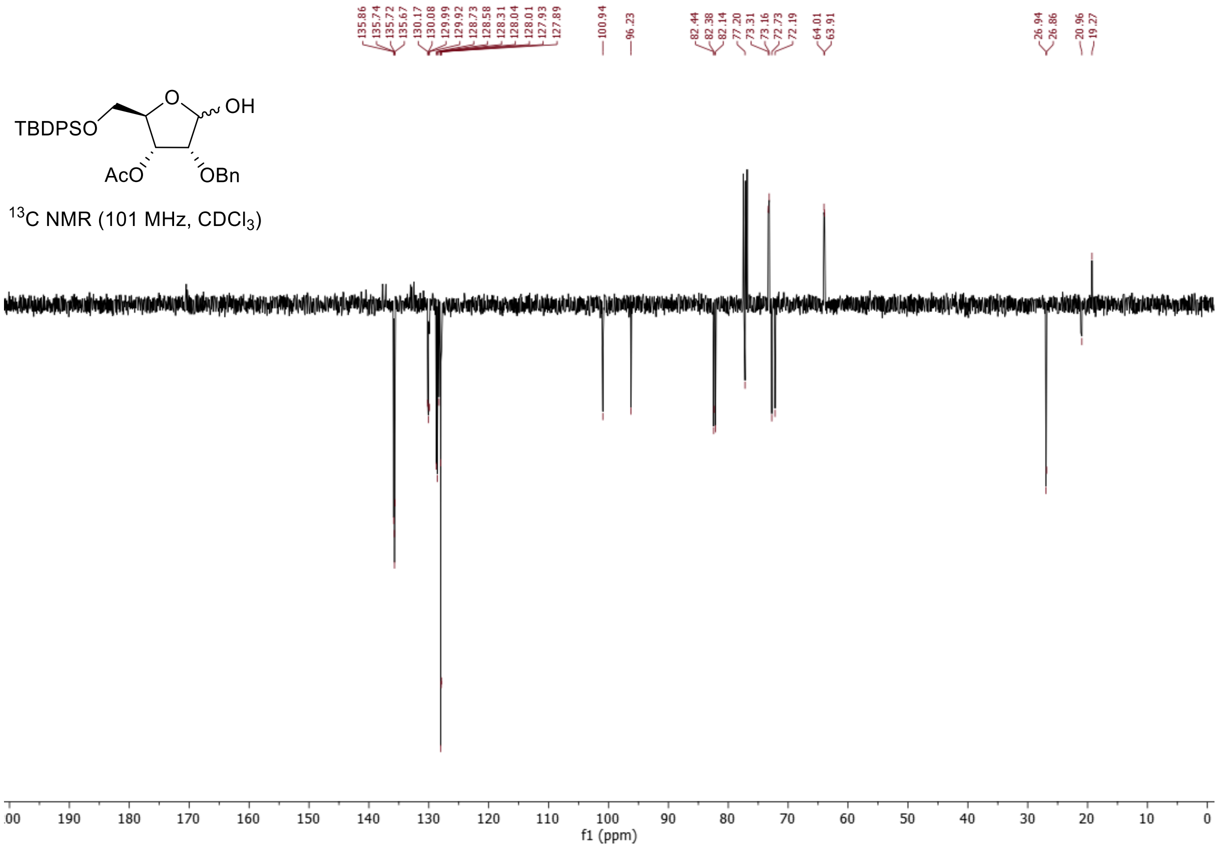
TBDPSO
OH
AcO
OBn
^{13}C NMR (101 MHz, CDCl$_3$)
f1 (ppm)

Compound **44**

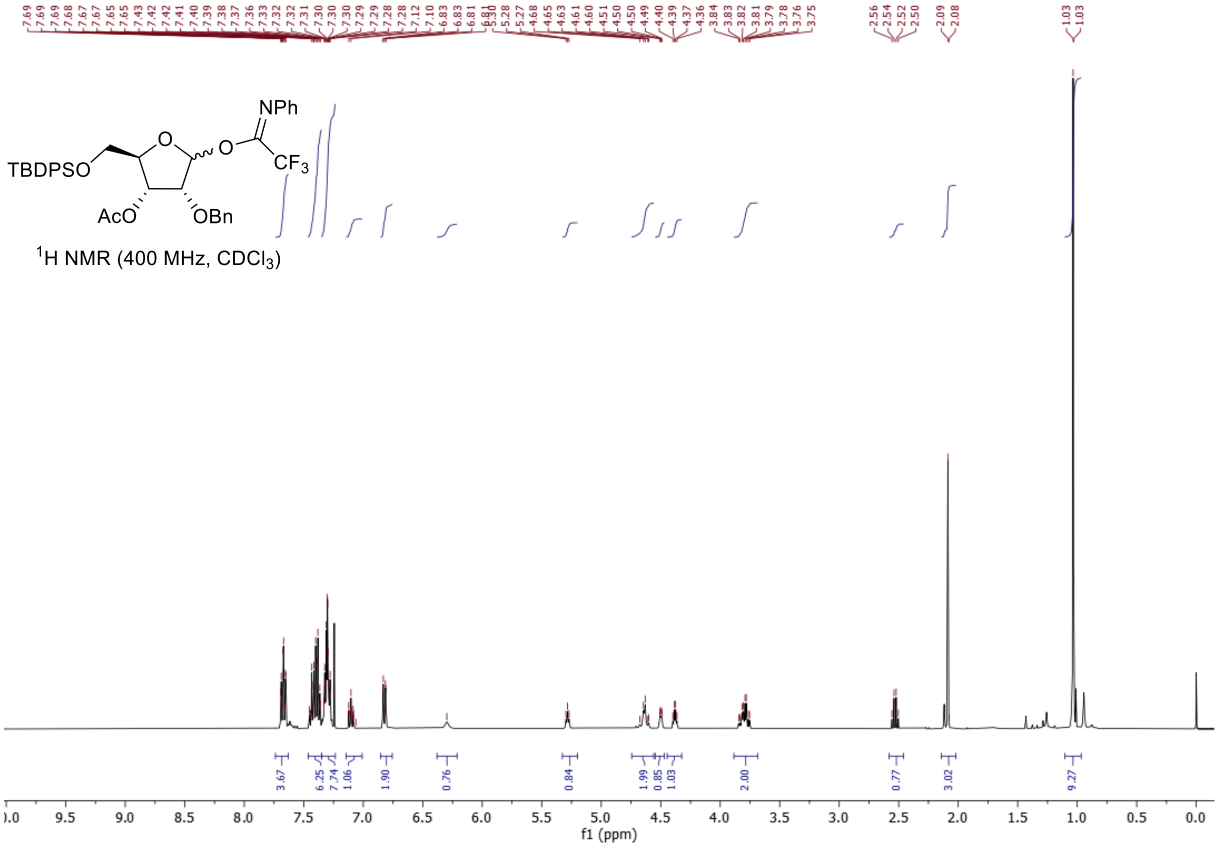
NPh
TBDPSO
O
CF_3
AcO
OBn
^{1}H NMR (400 MHz, CDCl$_3$)
f1 (ppm)

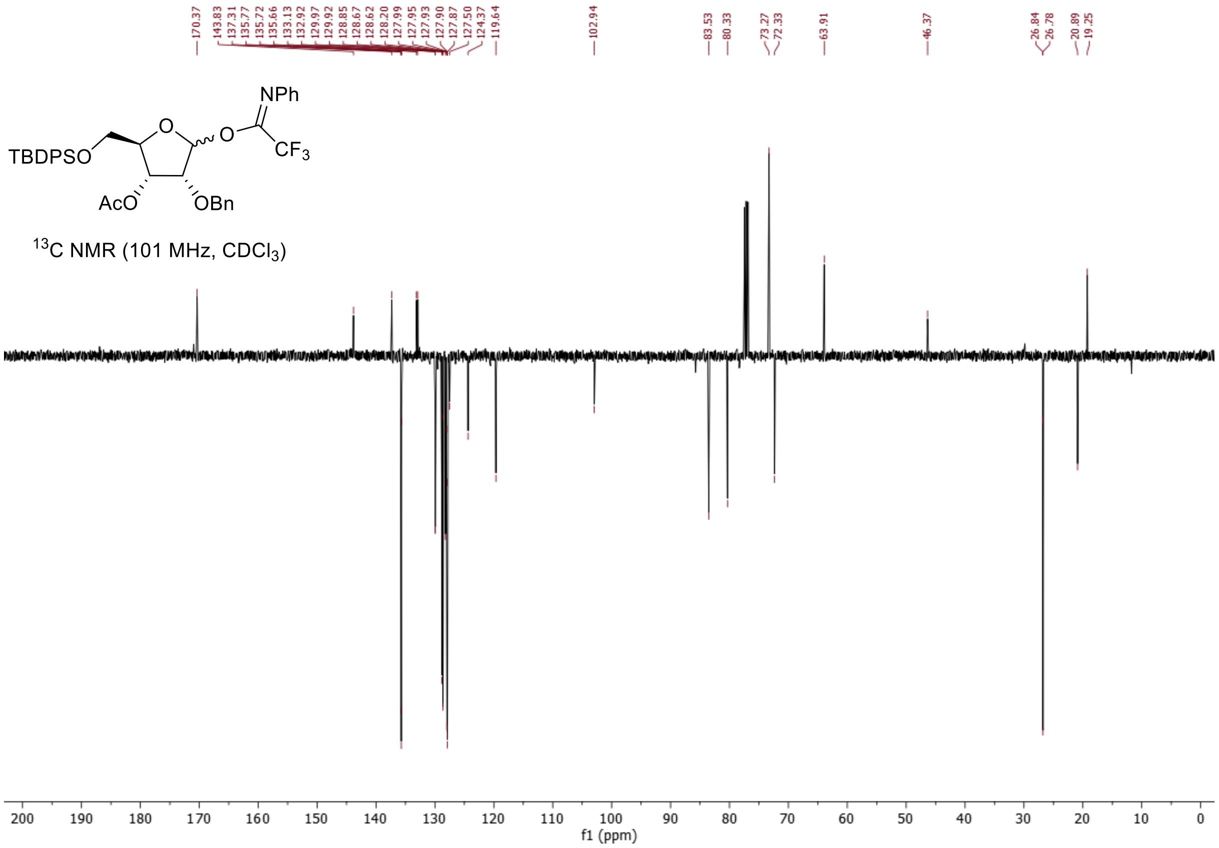
NPh
O
TBDPSO
CF3
AcO
OBn
13C NMR (101 MHz, CDCl3)
f1 (ppm)

Compound **45**

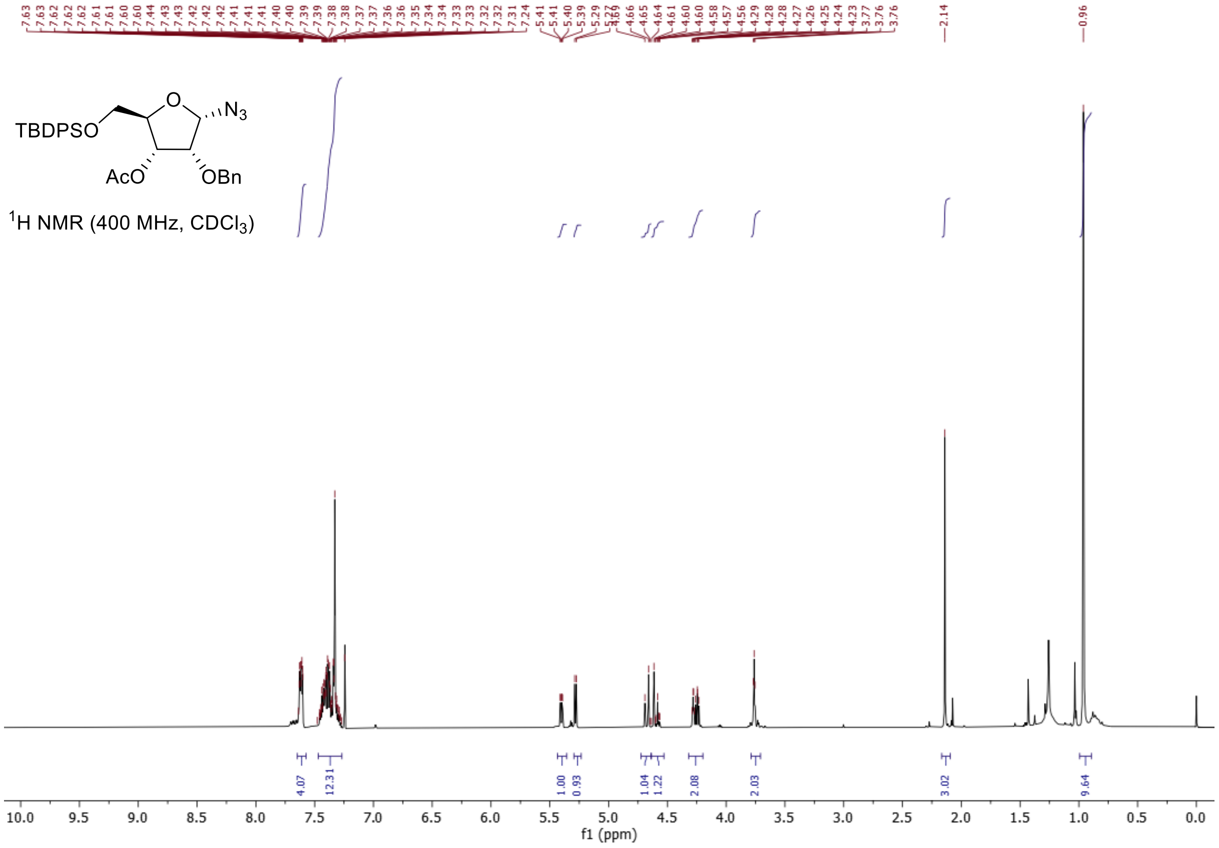
TBDPSO
O
N3
AcO
OBn
1H NMR (400 MHz, CDCl3)
f1 (ppm)

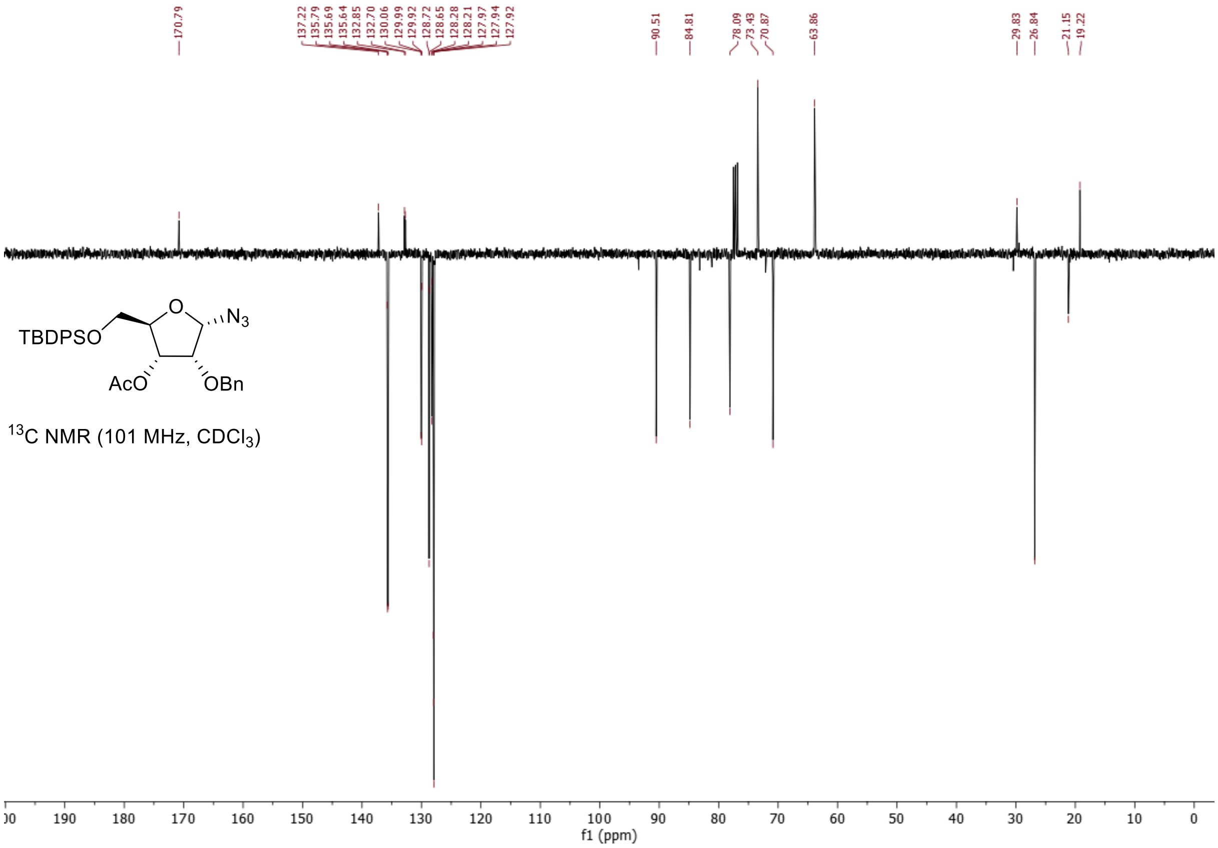
170.79
137.22 135.79 135.69 135.64 132.85 132.70 130.06 129.99 129.92 128.72 128.65 128.28 128.21 127.97 127.94 127.92
90.51
84.81
78.09 73.43 70.87
63.86
29.83 26.84 21.15 19.22
TBDPSO
O
N3
AcO
OBn
13C NMR (101 MHz, CDCl3)
f1 (ppm)

Compound **46**

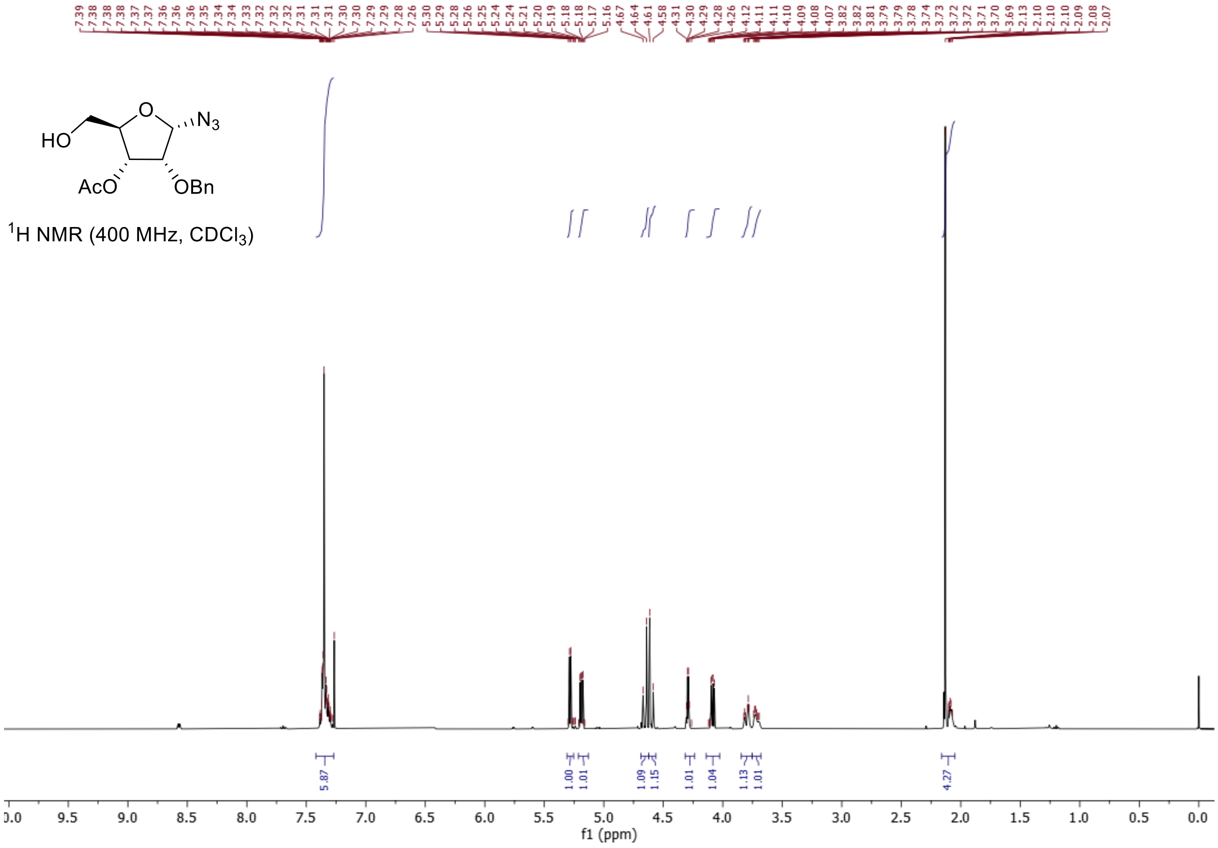
7.39 7.38 7.38 7.37 7.37 7.36 7.36 7.35 7.34 7.34 7.33 7.32 7.32 7.31 7.31 7.31 7.30 7.30 7.29 7.29 7.28 7.26 5.30 5.29 5.28 5.26 5.25 5.24 5.24 5.21 5.20 5.19 5.18 5.18 5.17 5.16 4.67 4.64 4.61 4.58 4.31 4.30 4.29 4.28 4.26 4.12 4.11 4.11 4.10 4.09 4.08 4.07 3.82 3.82 3.81 3.79 3.79 3.78 3.74 3.73 3.72 3.72 3.71 3.70 3.69 2.13 2.10 2.10 2.10 2.09 2.08 2.07
HO
O
N3
AcO
OBn
1H NMR (400 MHz, CDCl3)
5.87
1.00
1.01
1.09
1.15
1.01
1.04
1.13
1.01
4.27
f1 (ppm)

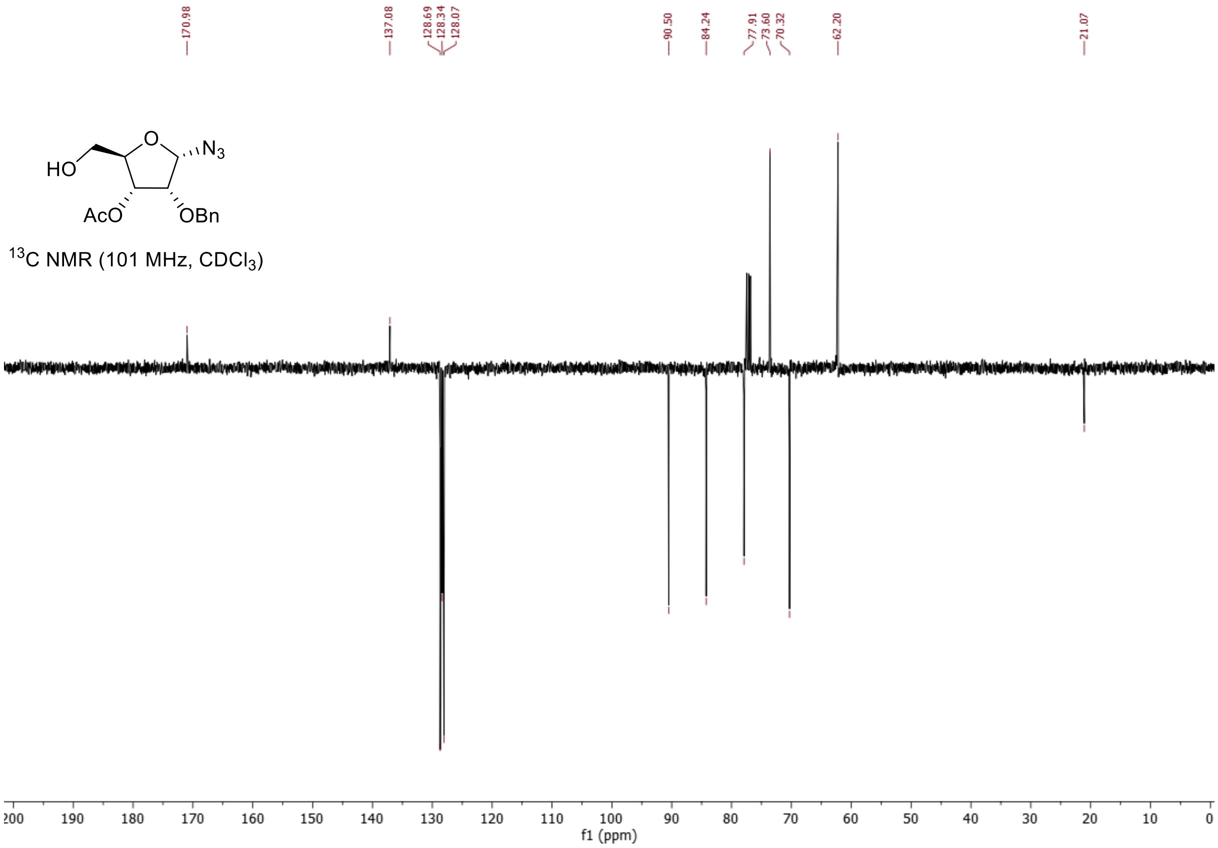
170.98
137.08
128.69
128.34
128.07
90.50
84.24
77.91
73.60
70.32
62.20
21.07
HO
O
N3
AcO
OBn
13C NMR (101 MHz, CDCl3)
f1 (ppm)

Compound **47**

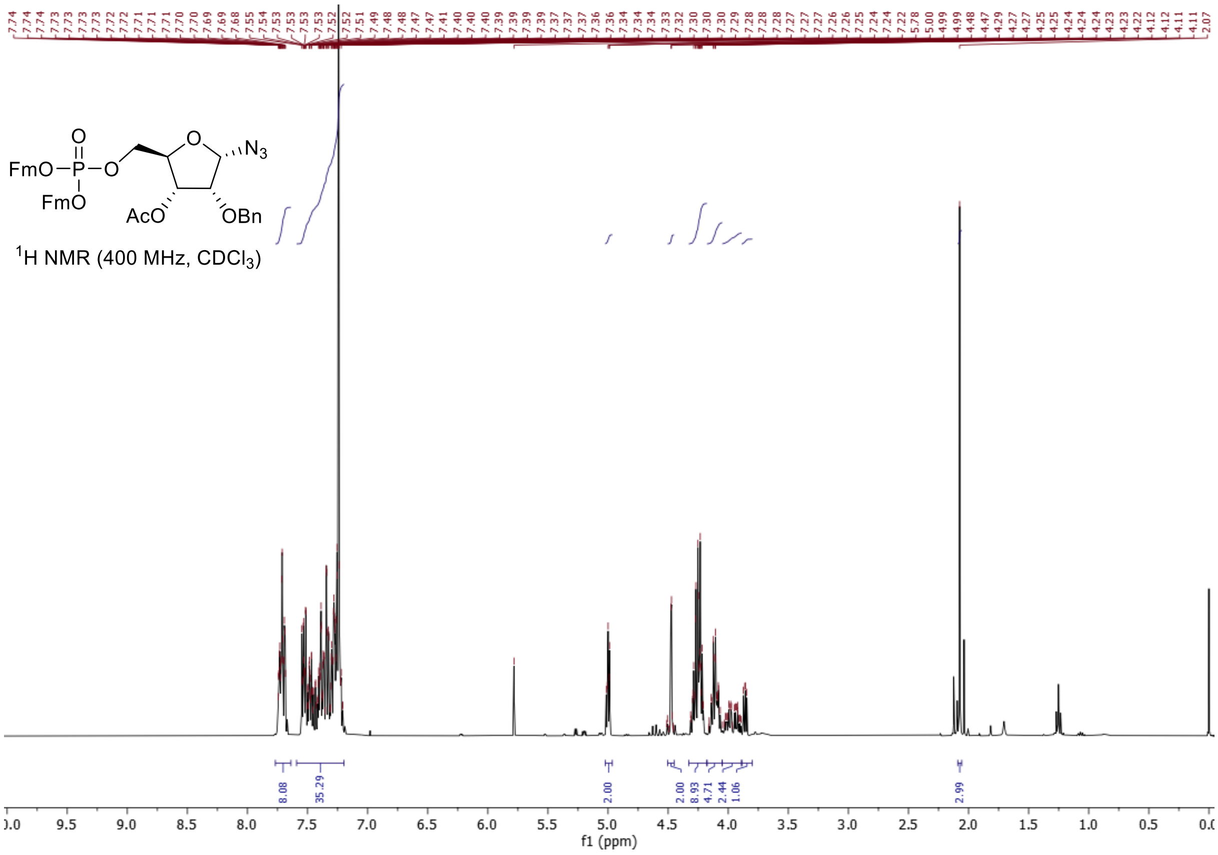
O
FmO
P
O
FmO
O
N3
AcO
OBn
1H NMR (400 MHz, CDCl3)
8.08
35.29
2.00
2.00
8.93
4.71
2.44
1.06
2.99
f1 (ppm)

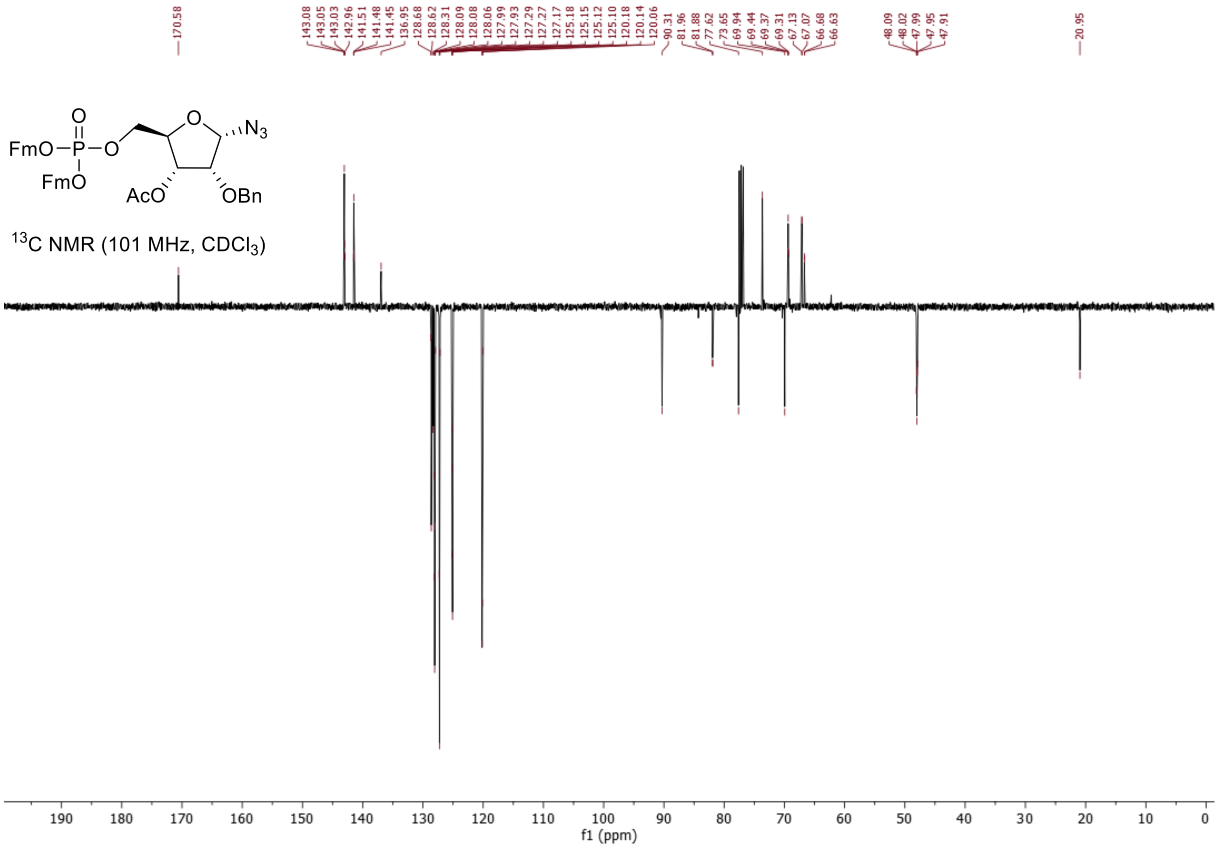
170.58
143.08 143.05 143.03 142.96 141.51 141.48 141.45 136.95 128.68 128.62 128.31 128.09 128.08 128.06 127.99 127.93 127.29 127.27 127.17 125.18 125.15 125.12 125.10 120.18 120.14 120.06
90.31 81.96 81.88 77.62 73.65 69.94 69.44 69.37 69.31 67.13 67.07 66.68 66.63
48.09 48.02 47.99 47.95 47.91
20.95
FmO
FmO
O
P
O
O
N3
AcO
OBn
13C NMR (101 MHz, CDCl3)
190 180 170 160 150 140 130 120 110 100 90 80 70 60 50 40 30 20 10 0
f1 (ppm)

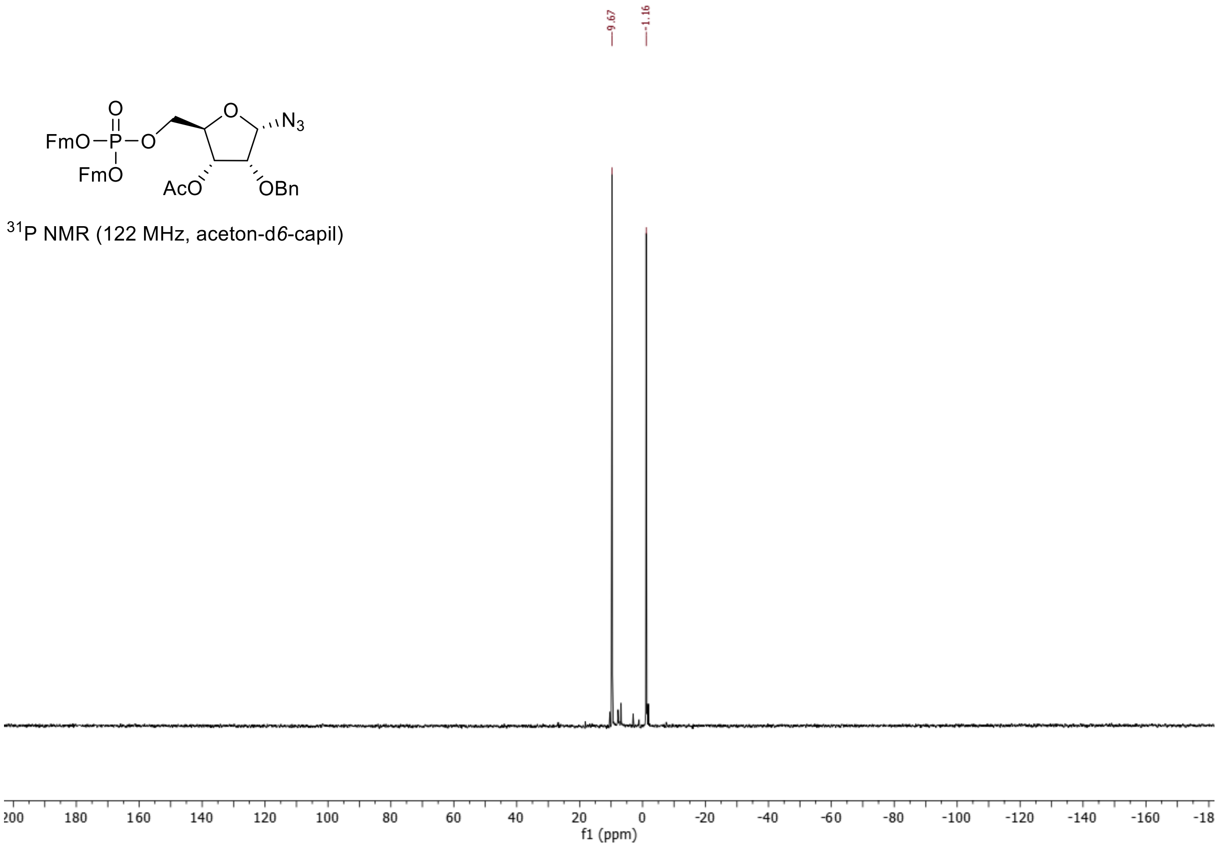
9.67
-1.16
FmO
FmO
O
P
O
O
N3
AcO
OBn
31P NMR (122 MHz, aceton-d6-capil)
200 180 160 140 120 100 80 60 40 20 0 -20 -40 -60 -80 -100 -120 -140 -160 -18
f1 (ppm)

Compound **7**

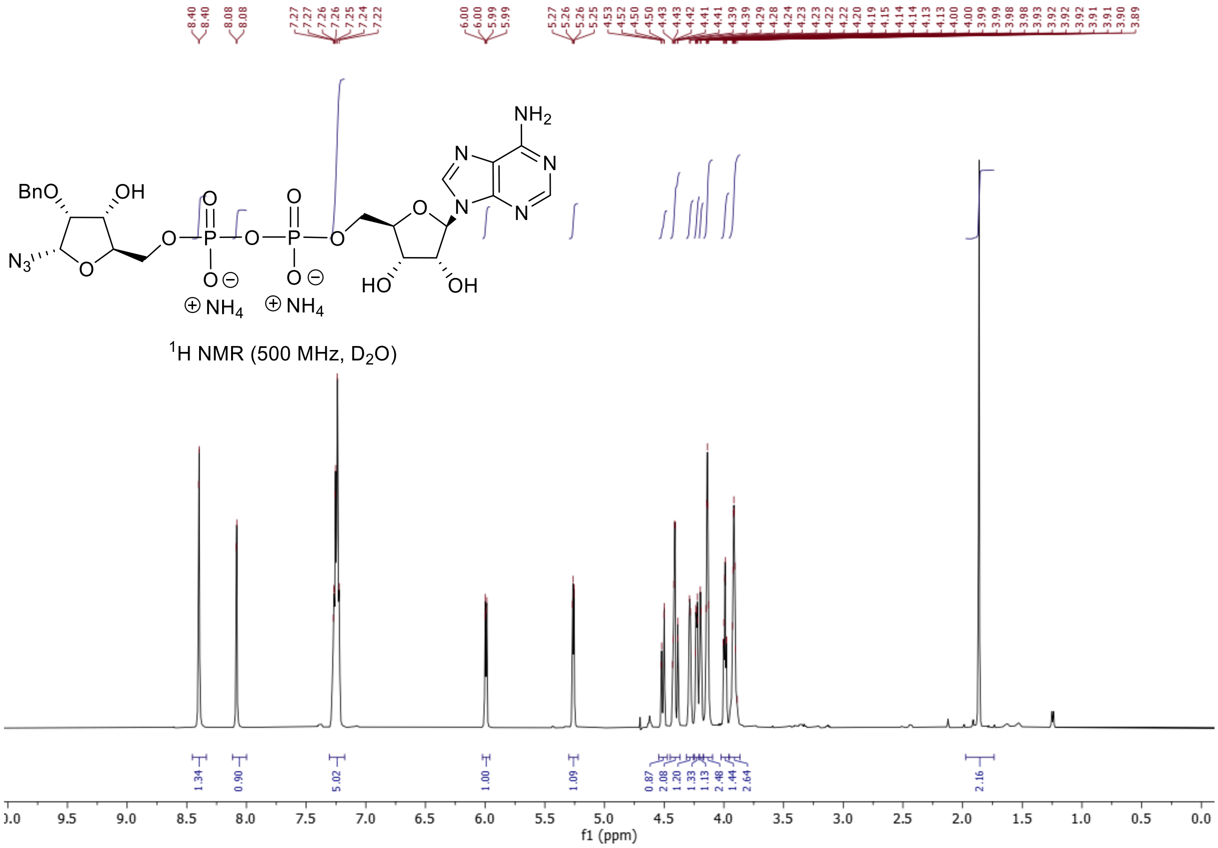

8.40
8.40
8.08
8.08
7.27
7.27
7.26
7.26
7.25
7.24
7.22
6.00
6.00
5.99
5.99
5.27
5.26
5.26
5.25
NH2
BnO
OH
N3
O
NH4
HO
OH
1H NMR (500 MHz, D2O)
1.34
0.90
5.02
1.00
1.09
0.87
2.08
1.20
1.33
1.13
2.48
1.44
2.64
2.16
f1 (ppm)

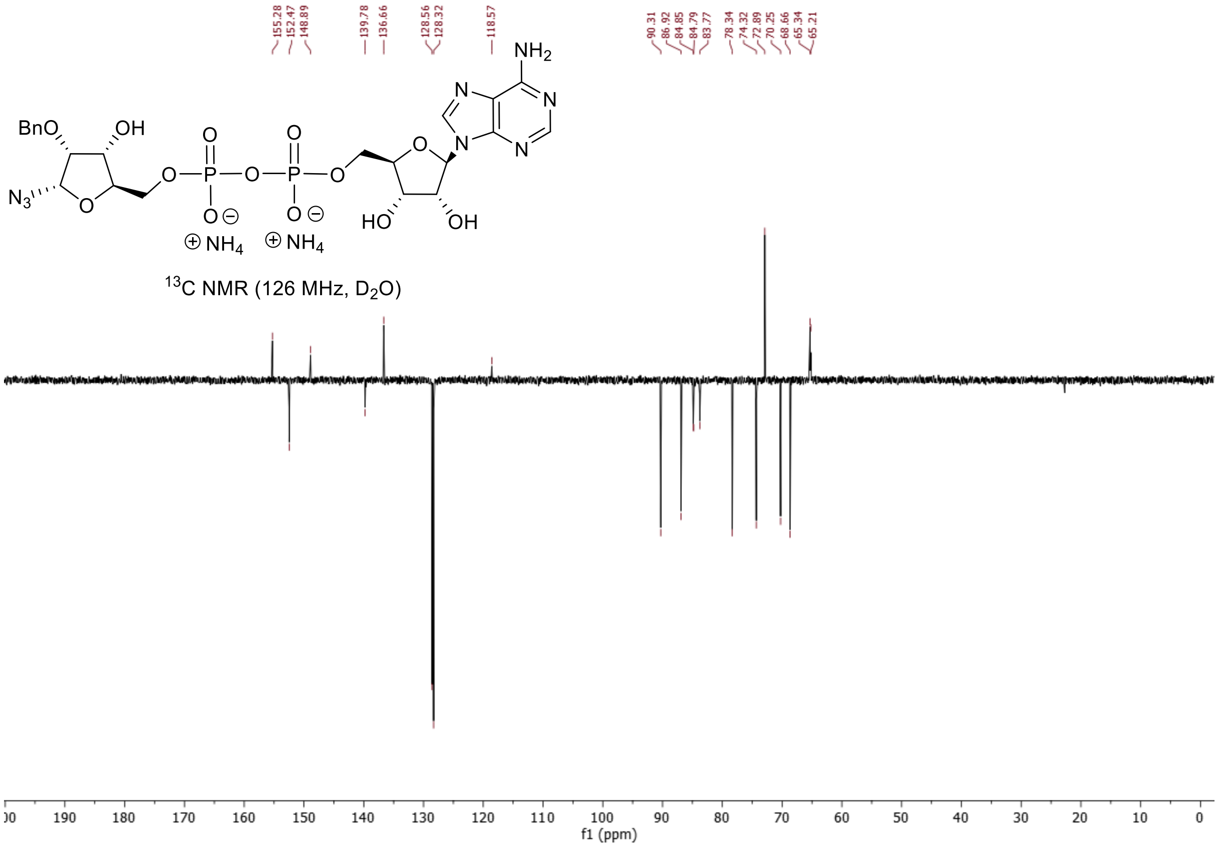

155.28
152.47
148.89
139.78
136.66
128.56
128.32
118.57
90.31
86.92
84.85
84.79
83.77
78.34
74.32
72.89
70.25
68.66
65.34
65.21
NH2
BnO
OH
N3
O
NH4
HO
OH
13C NMR (126 MHz, D2O)
f1 (ppm)

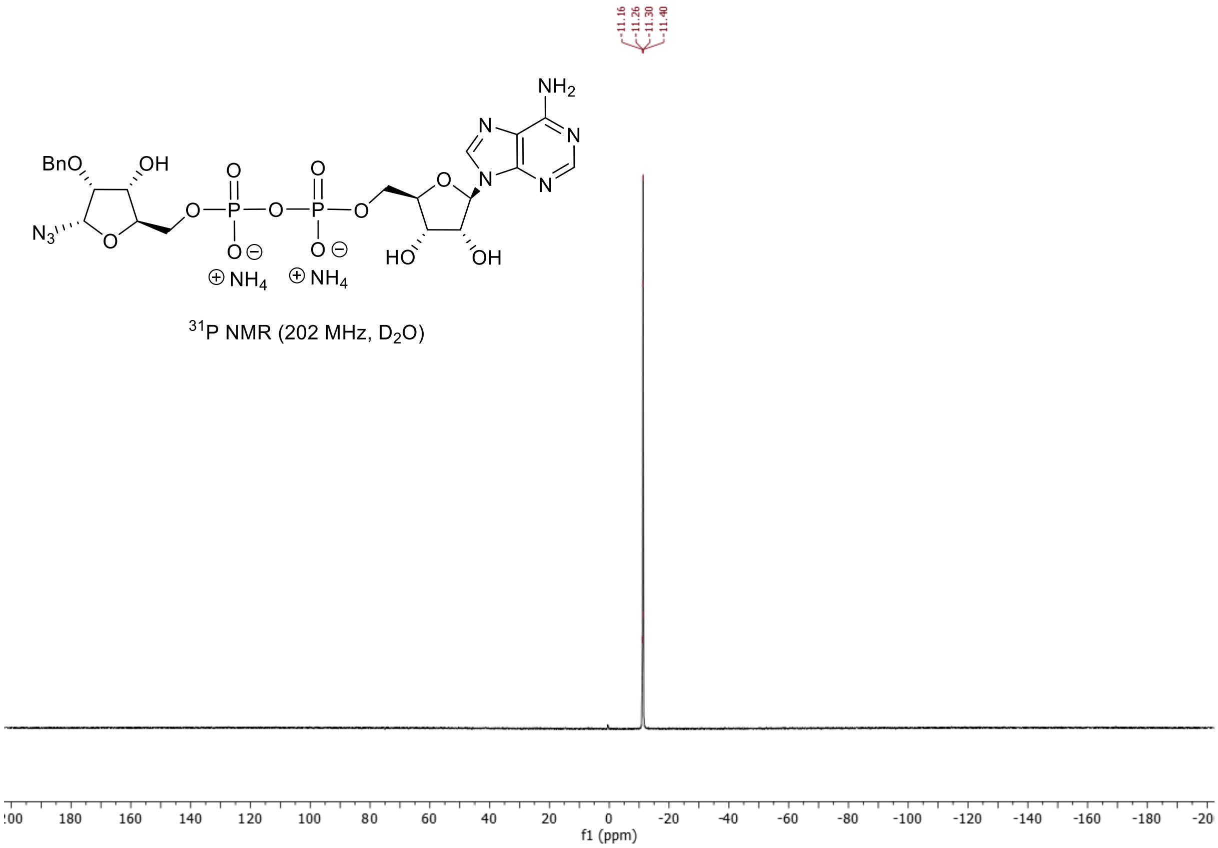
-11.16
-11.26
-11.30
-11.40
NH2
BnO
OH
N3
O
NH4
HO
OH
31P NMR (202 MHz, D2O)
f1 (ppm)

Compound **15**

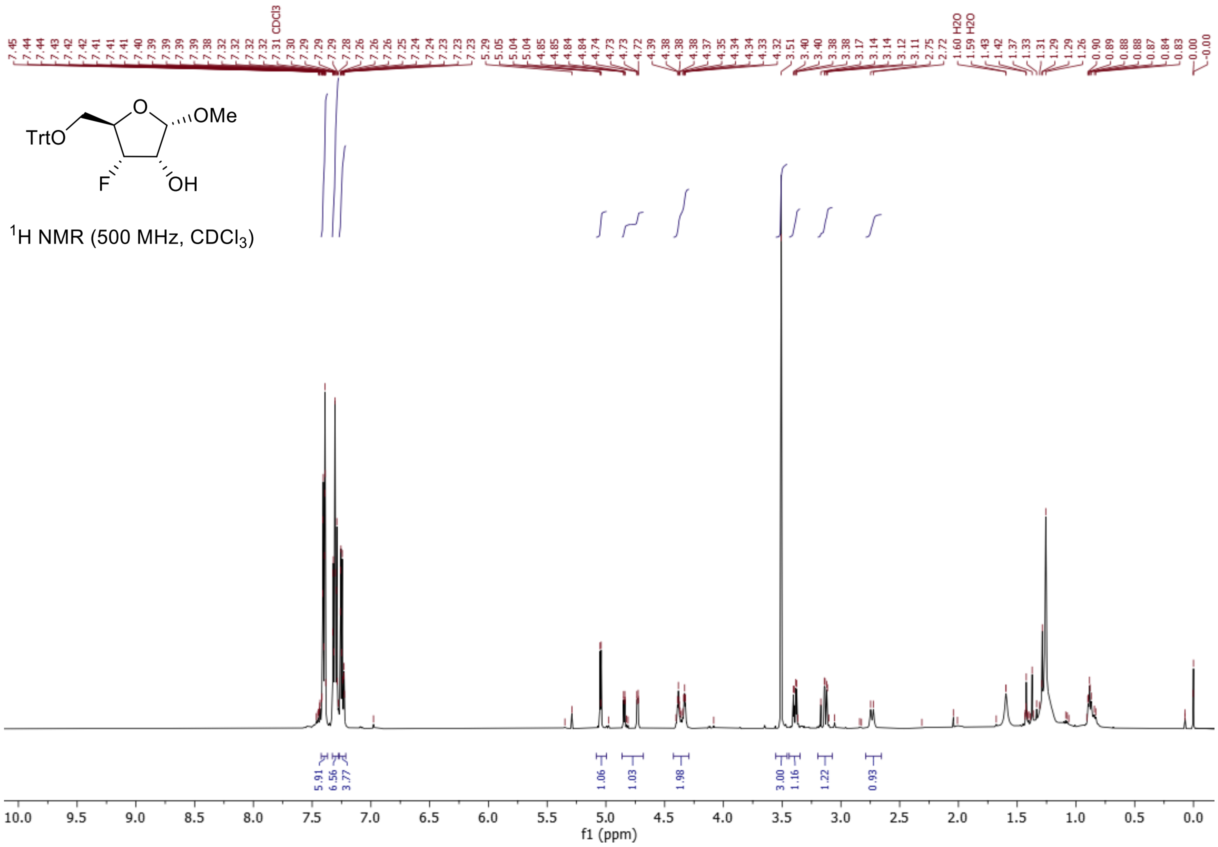
TrtO
OMe
F
OH
1H NMR (500 MHz, CDCl3)
f1 (ppm)

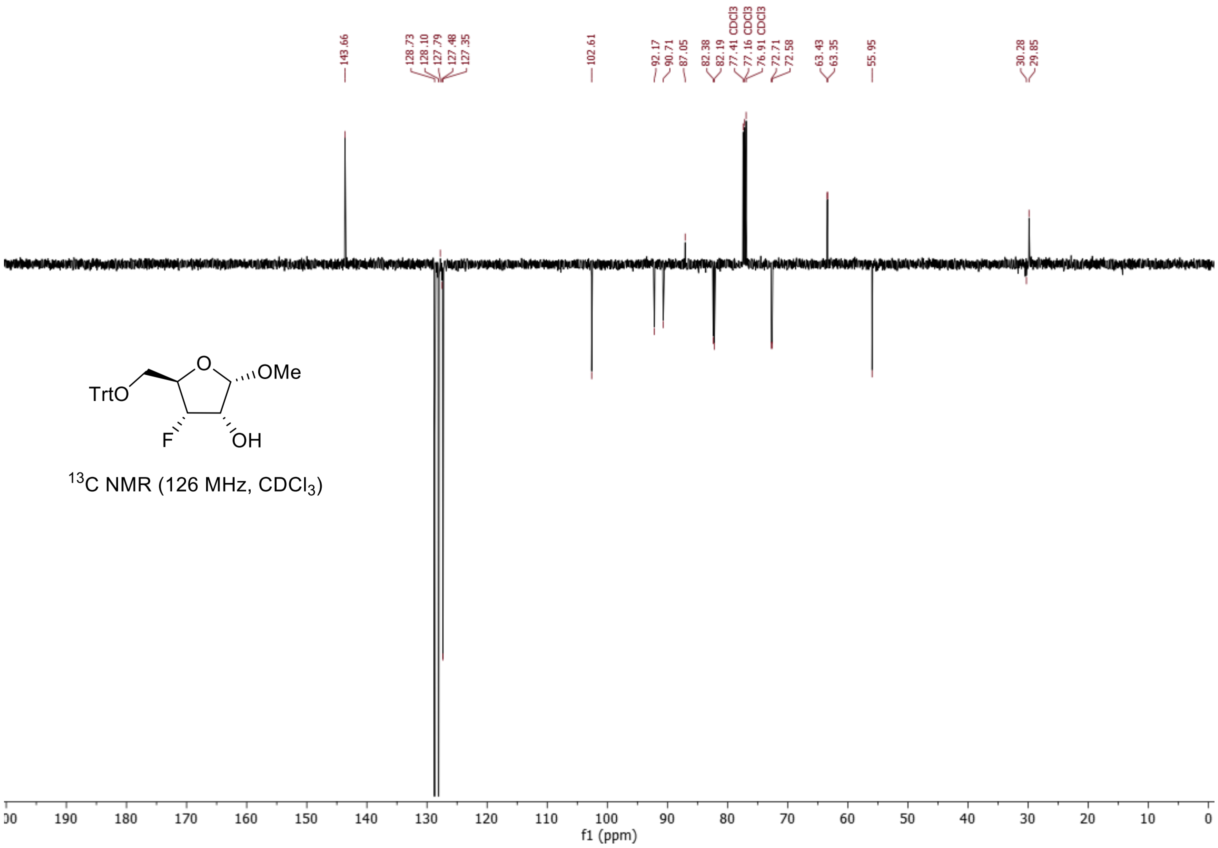
143.66
128.73
128.10
127.79
127.48
127.35
102.61
92.17
90.71
87.05
82.38
82.19
77.41 CDCl3
77.16 CDCl3
76.91 CDCl3
72.71
72.58
63.43
63.35
55.95
30.28
29.85
TrtO
O
OMe
F
OH
13C NMR (126 MHz, CDCl3)
f1 (ppm)

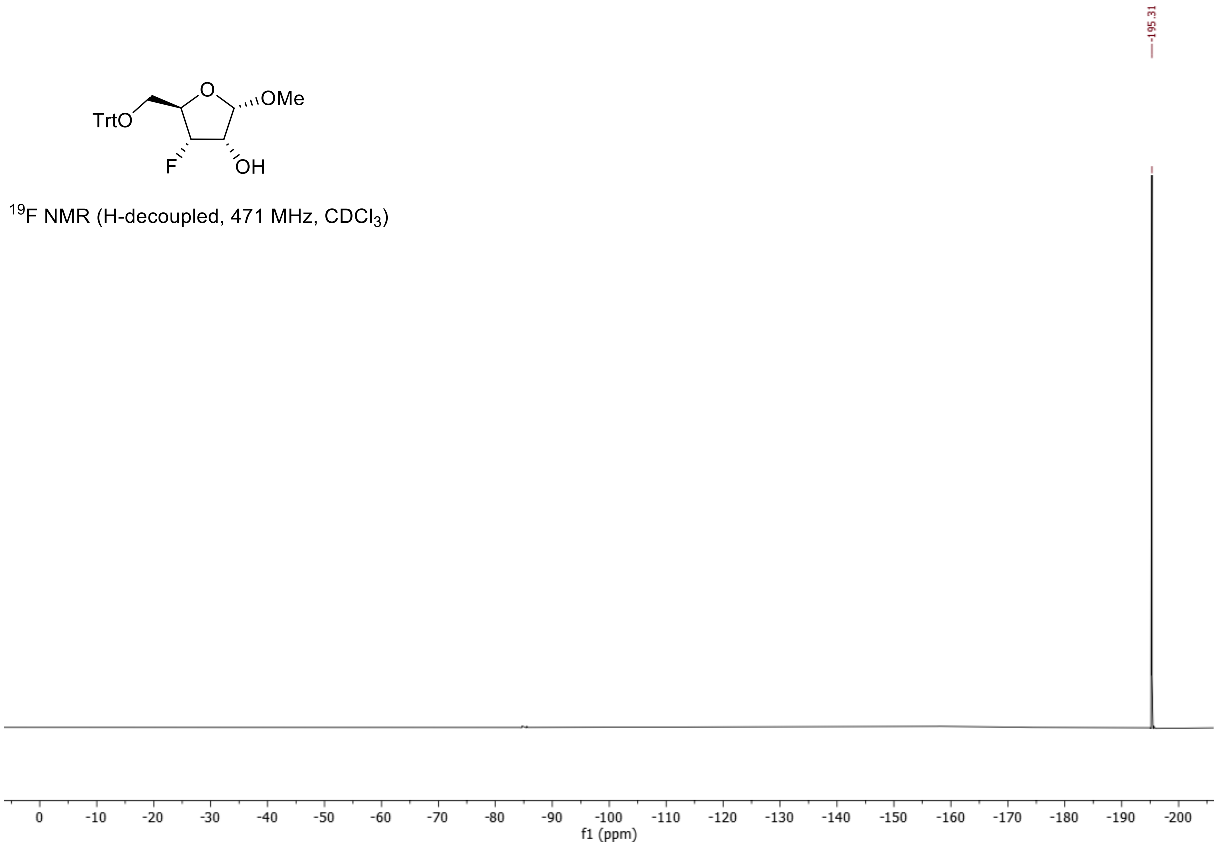
-195.31
TrtO
O
OMe
F
OH
19F NMR (H-decoupled, 471 MHz, CDCl3)
f1 (ppm)

Compound **49**

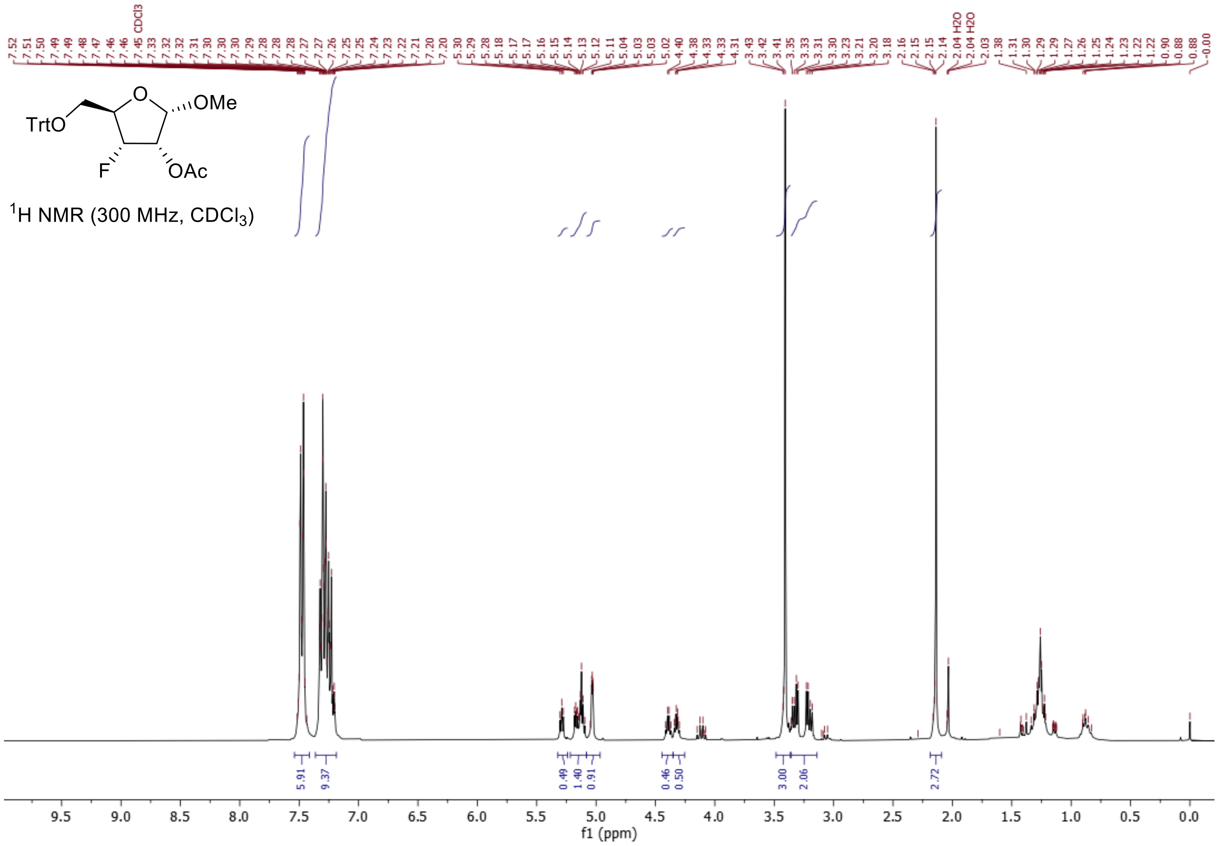
TrtO
OMe
F
OAc
1H NMR (300 MHz, CDCl3)
f1 (ppm)

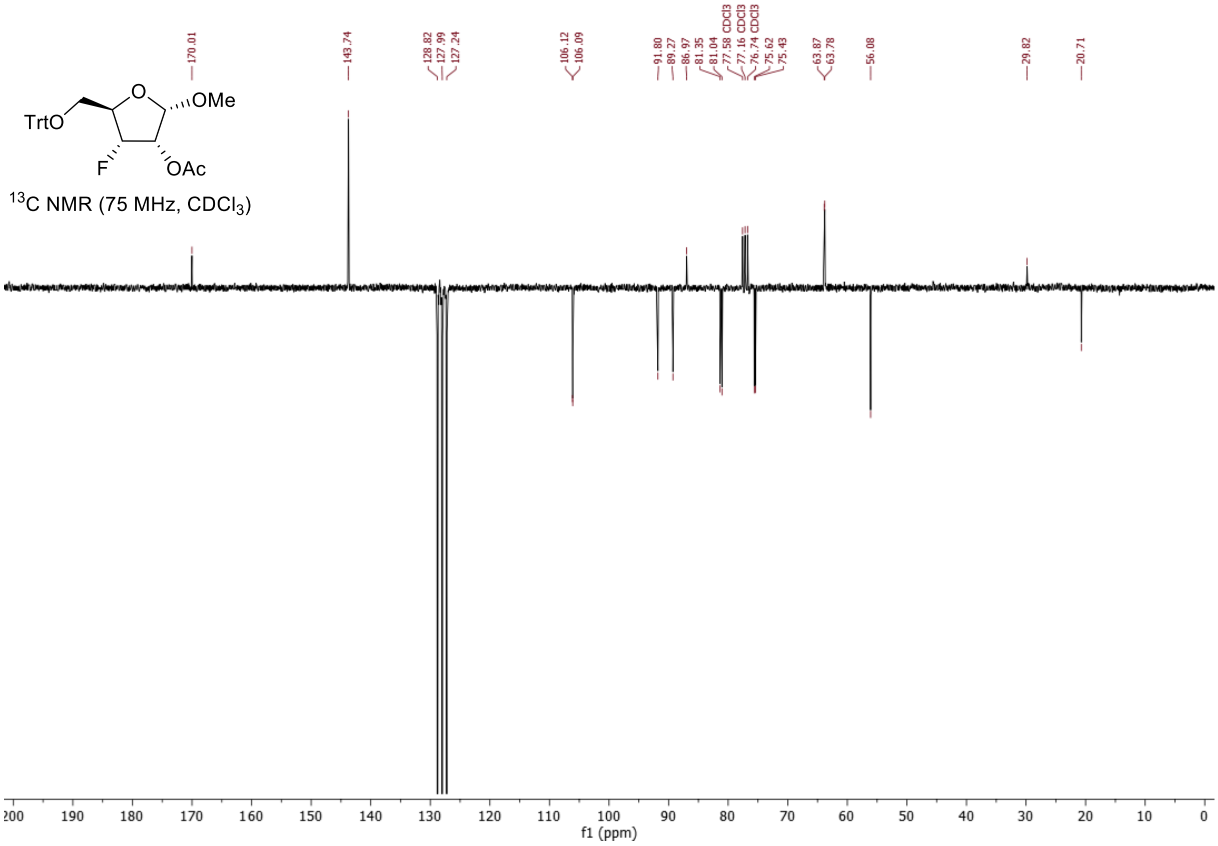
TrtO
OMe
F
OAc
13C NMR (75 MHz, CDCl3)
f1 (ppm)

Compound **50**

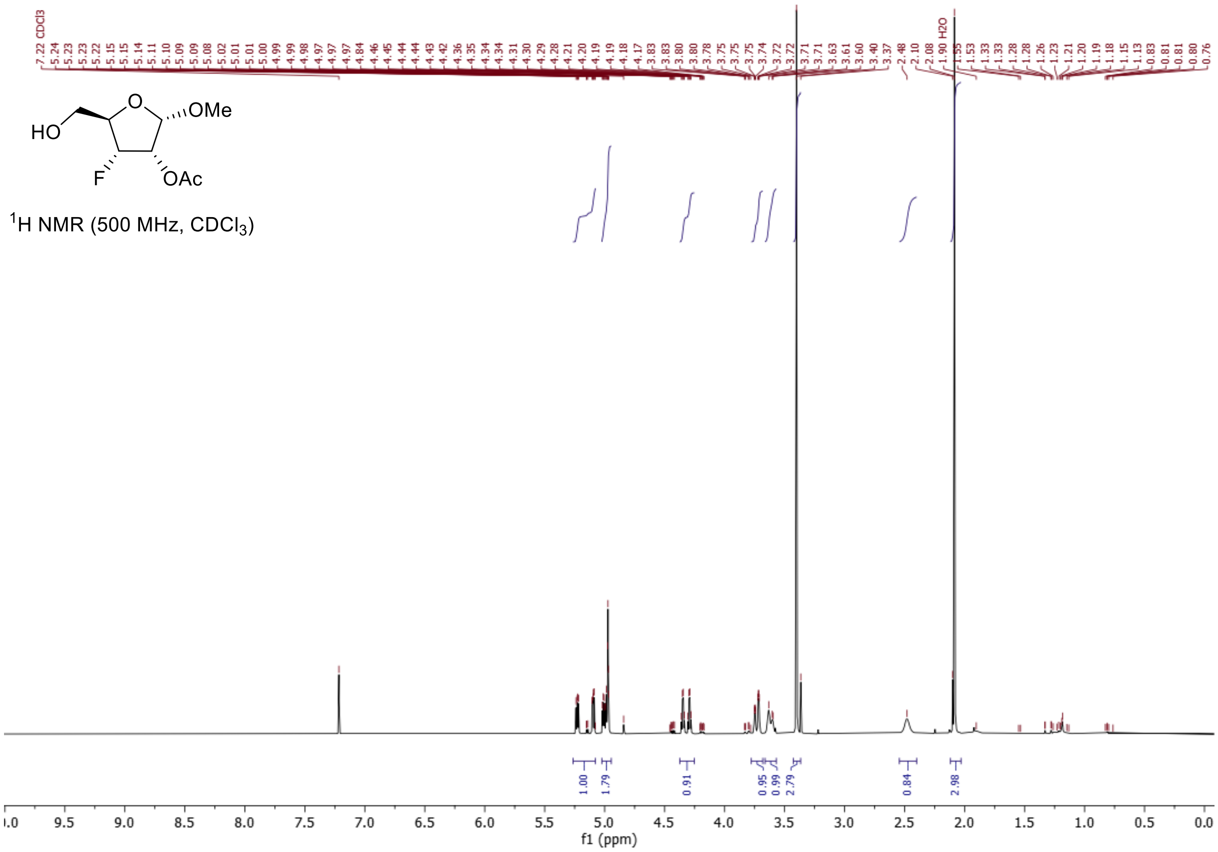

^{1}H NMR (500 MHz, $CDCl_3$)

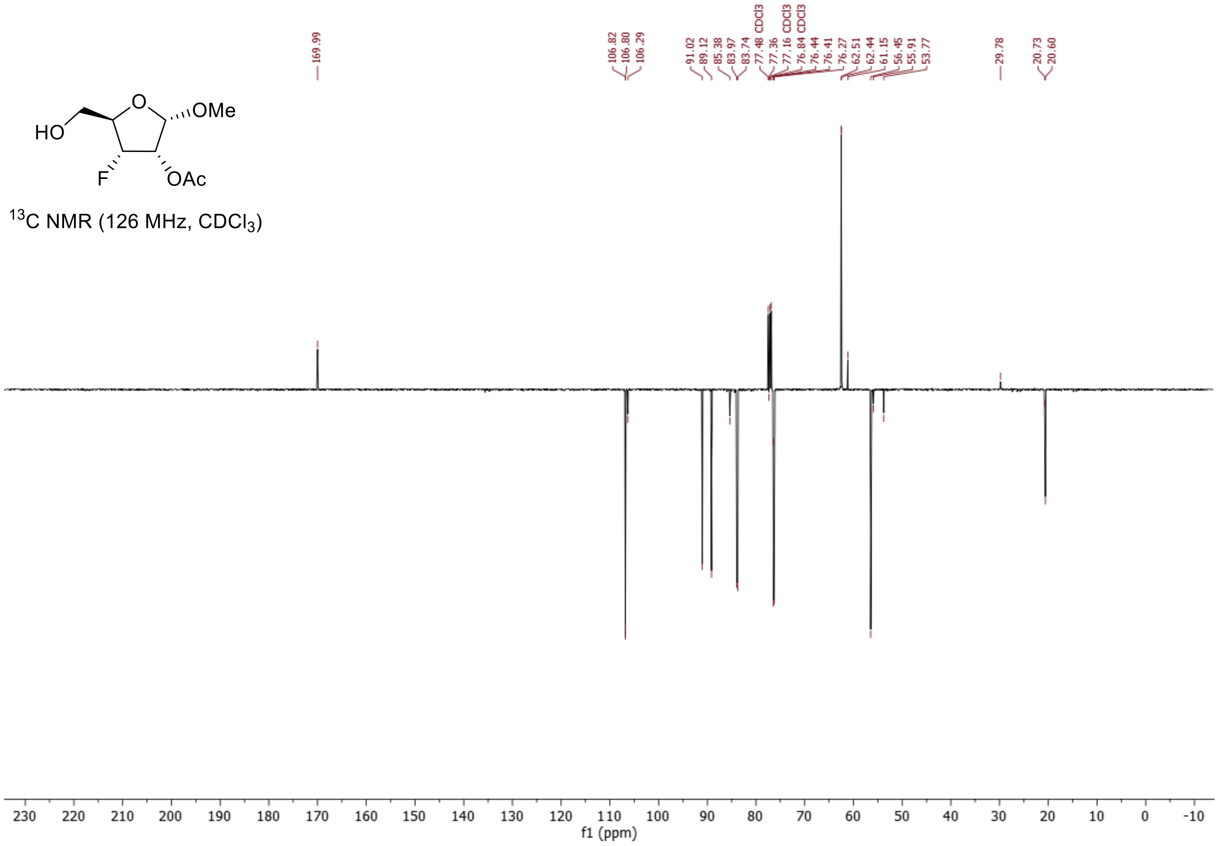

^{13}C NMR (126 MHz, $CDCl_3$)

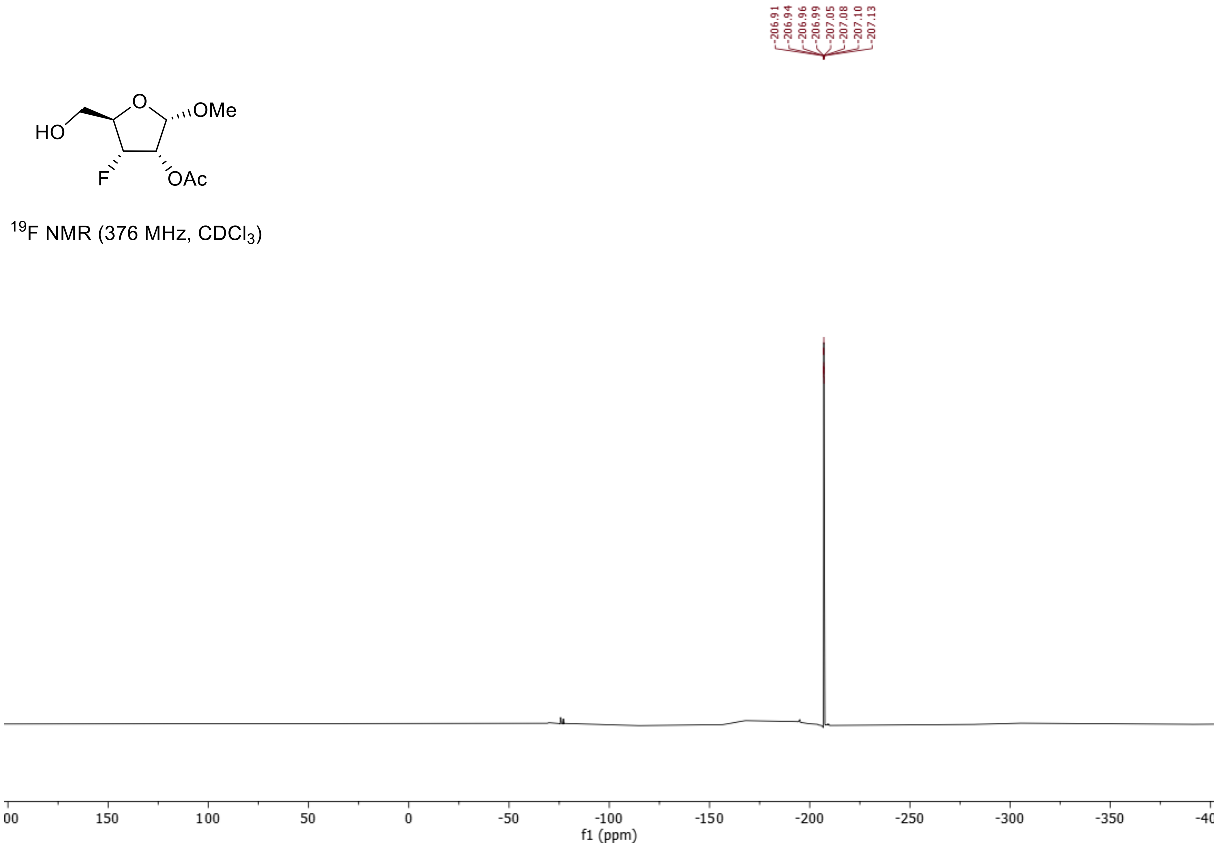

^{19}F NMR (376 MHz, $CDCl_3$)

Compound **51**

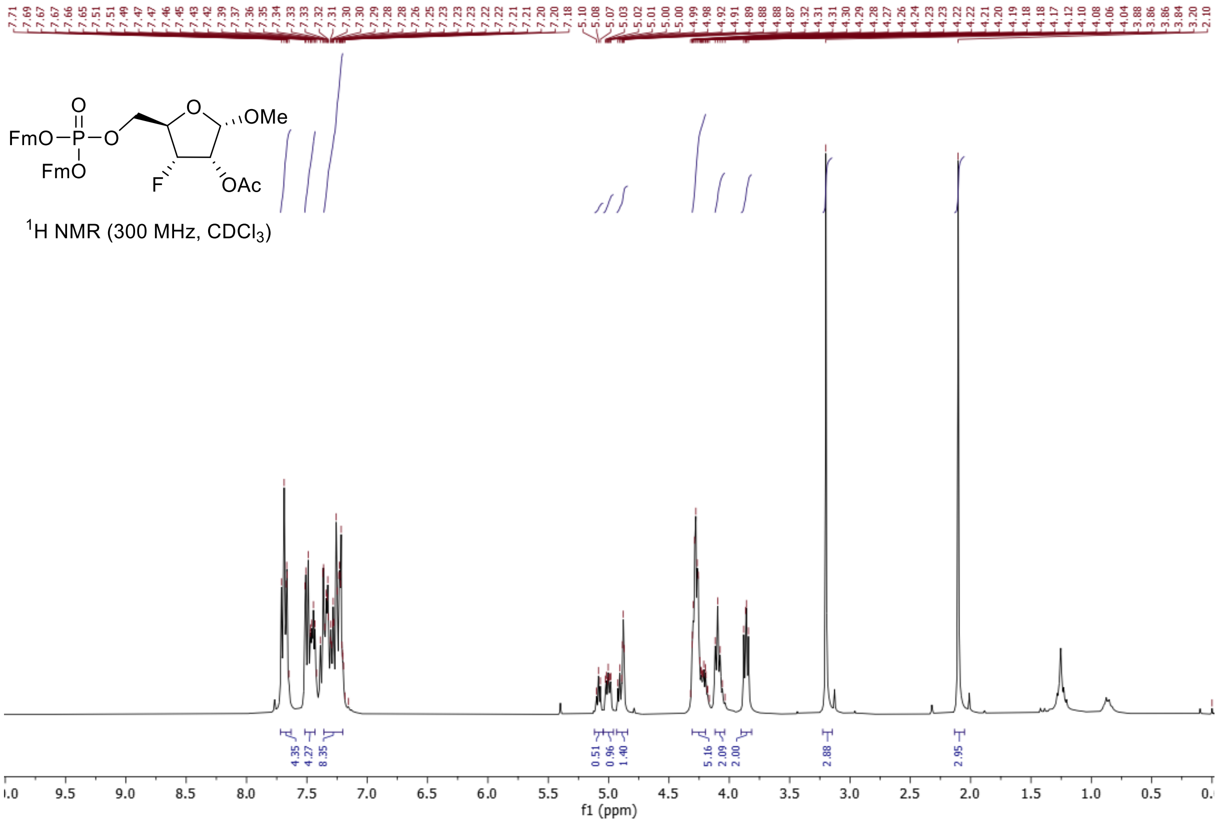

^1H NMR (300 MHz, $CDCl_3$)

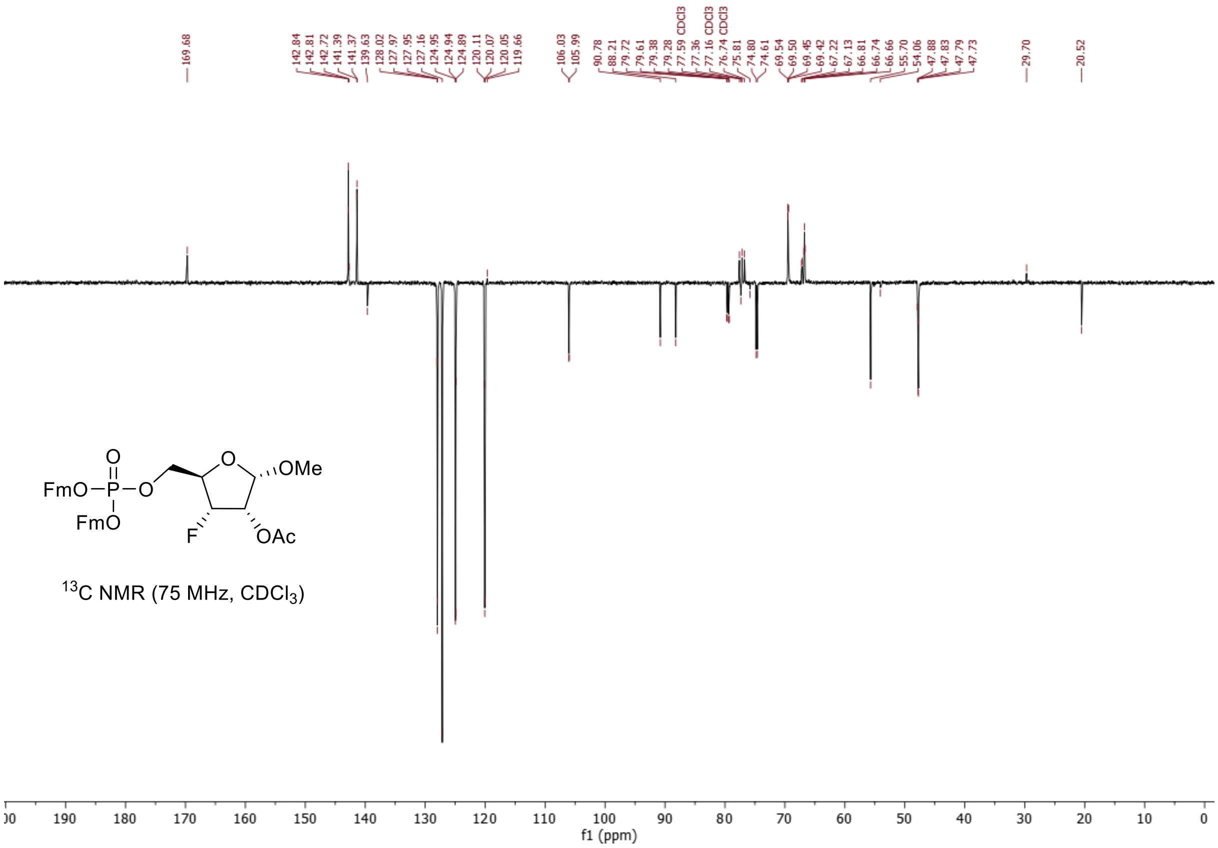
169.68
142.84
142.81
142.72
141.39
141.37
139.63
128.02
127.97
127.95
127.16
124.95
124.94
124.89
120.11
120.07
120.05
119.66
106.03
105.99
90.78
88.21
79.72
79.61
79.38
79.28
77.59 CDCl3
77.36
77.16 CDCl3
76.74 CDCl3
75.81
74.80
74.61
69.54
69.50
69.45
69.42
67.22
67.13
66.81
66.74
66.66
55.70
54.06
47.88
47.83
47.79
47.73
29.70
20.52
FmO
FmO
O
P
O
O
OMe
F
OAc
^{13}C NMR (75 MHz, CDCl$_3$)
f1 (ppm)

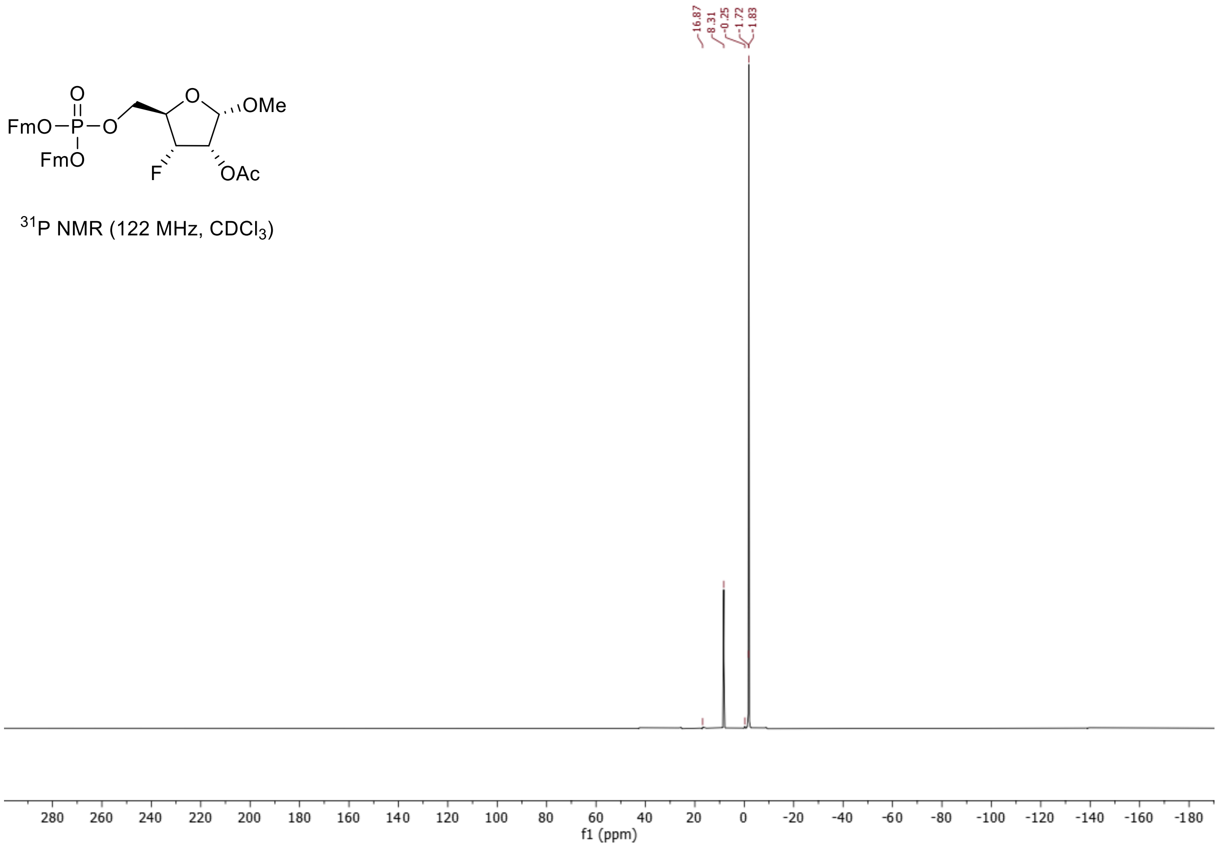
16.87
8.31
-0.25
-1.72
-1.83
FmO
FmO
O
P
O
O
OMe
F
OAc
^{31}P NMR (122 MHz, CDCl$_3$)
f1 (ppm)

Compound **8**

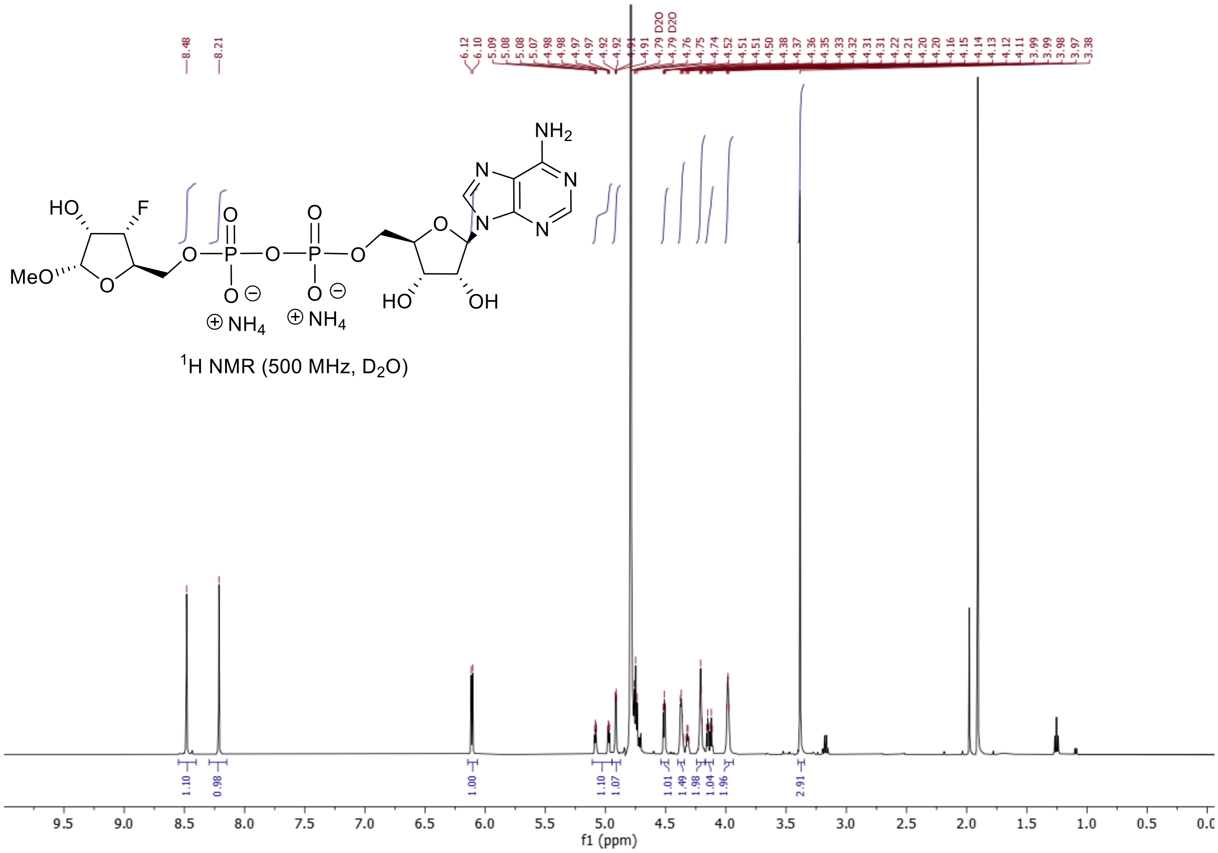

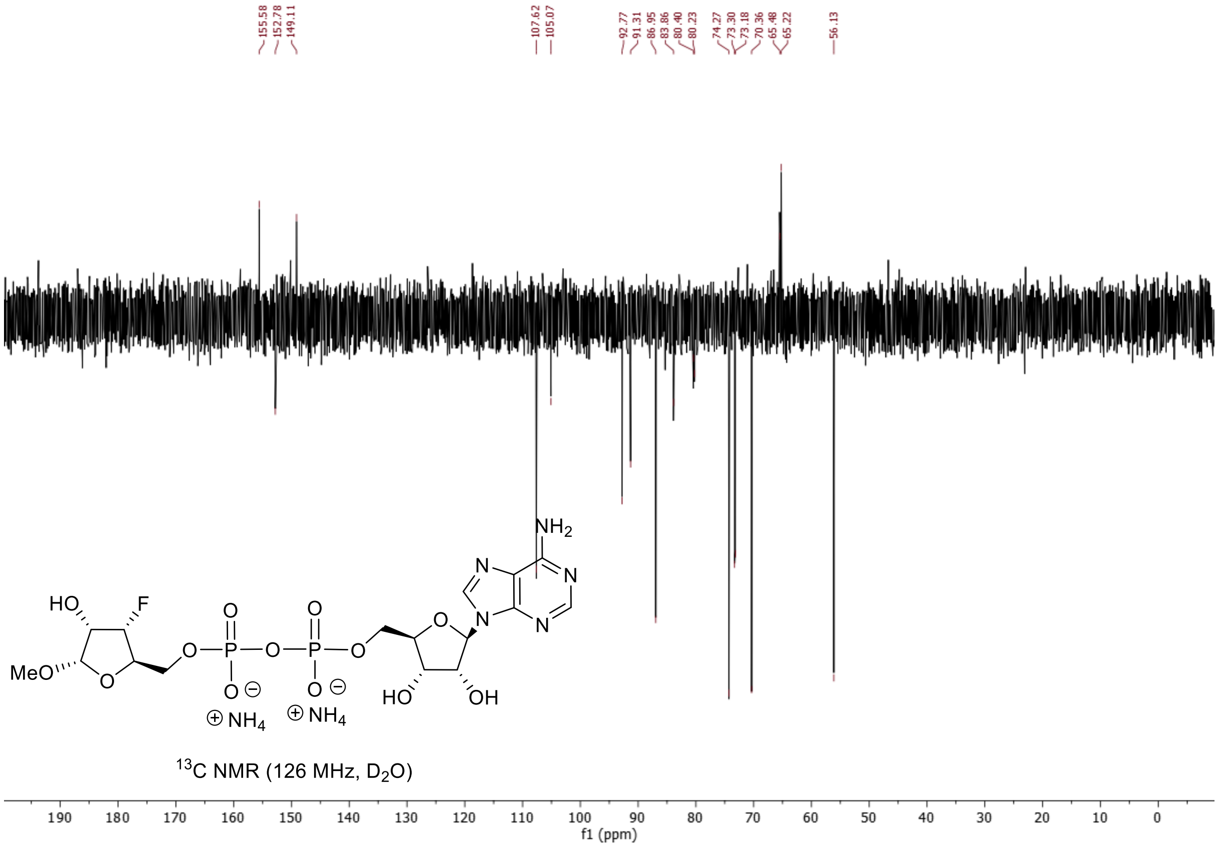

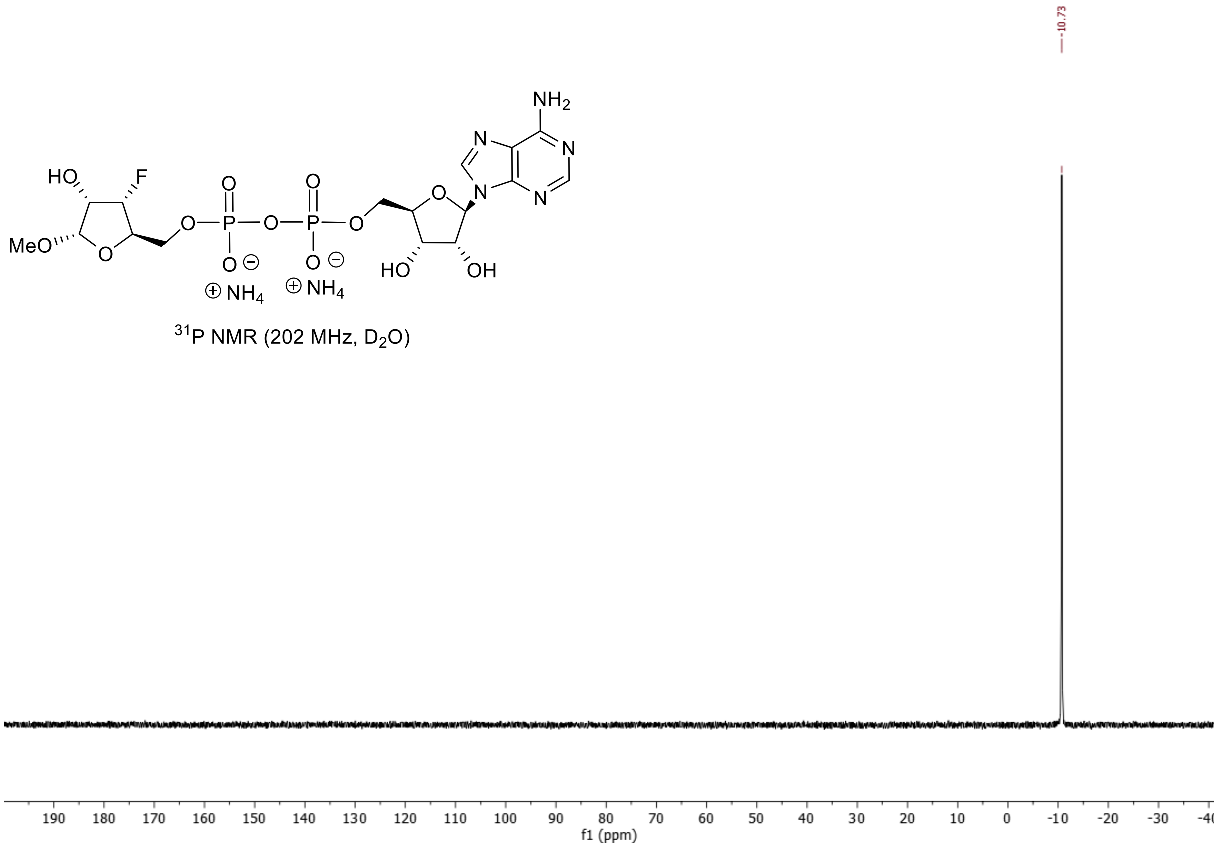

^{31}P NMR (202 MHz, D_2O)

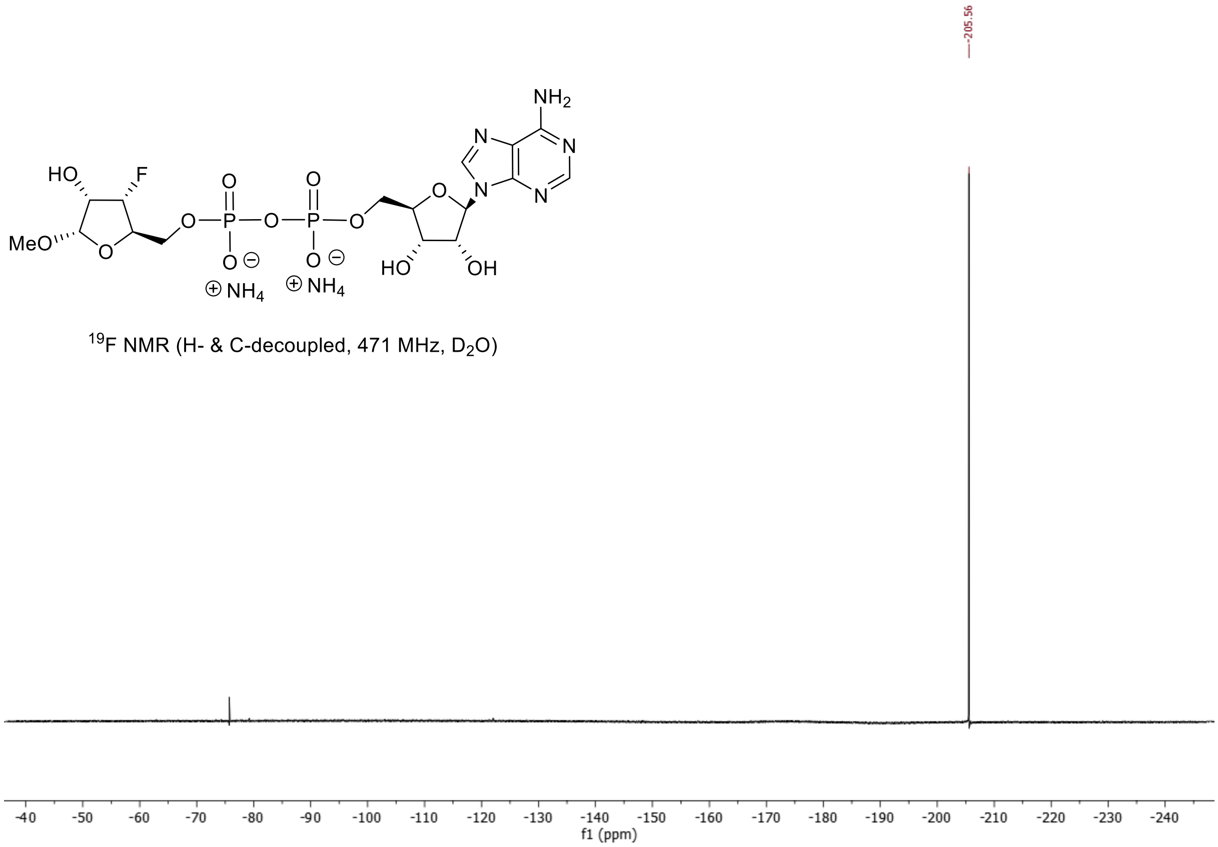

^{19}F NMR (H- & C-decoupled, 471 MHz, D_2O)

Compound **53**

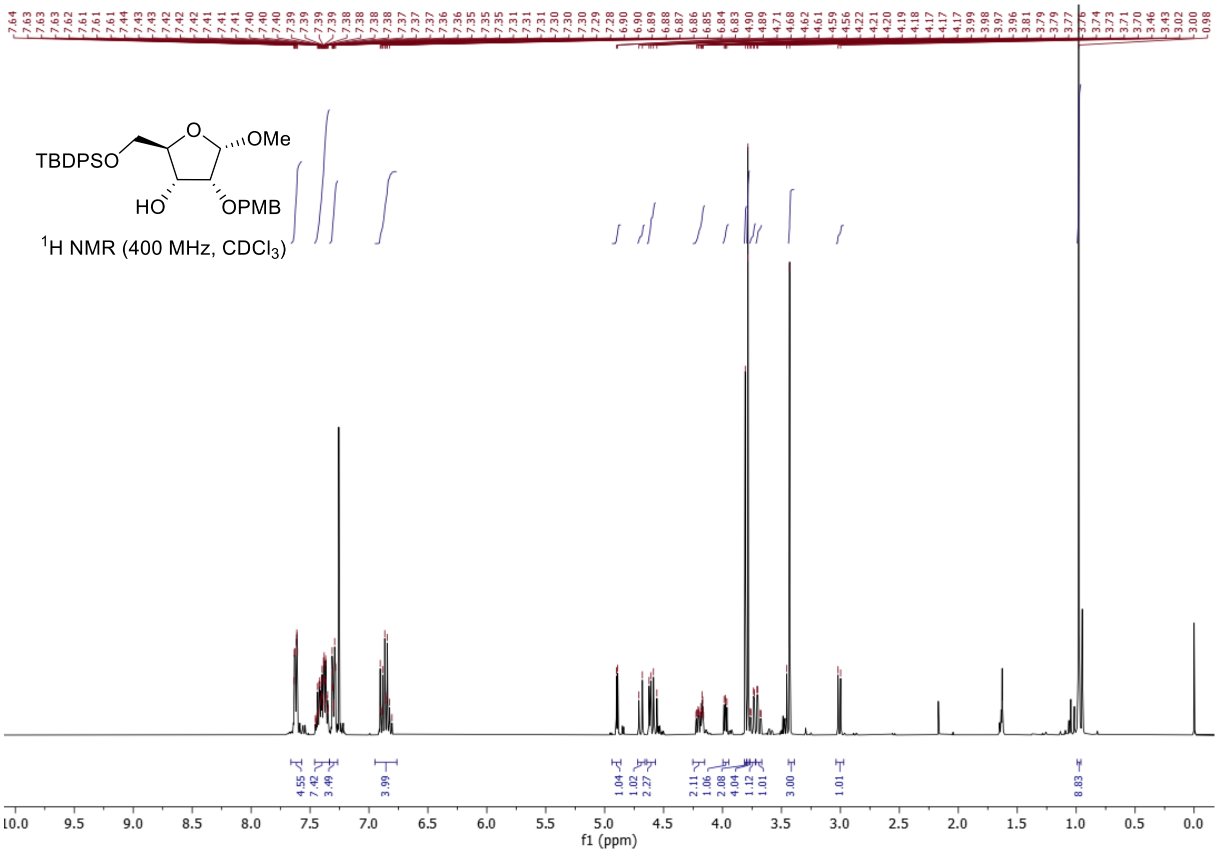

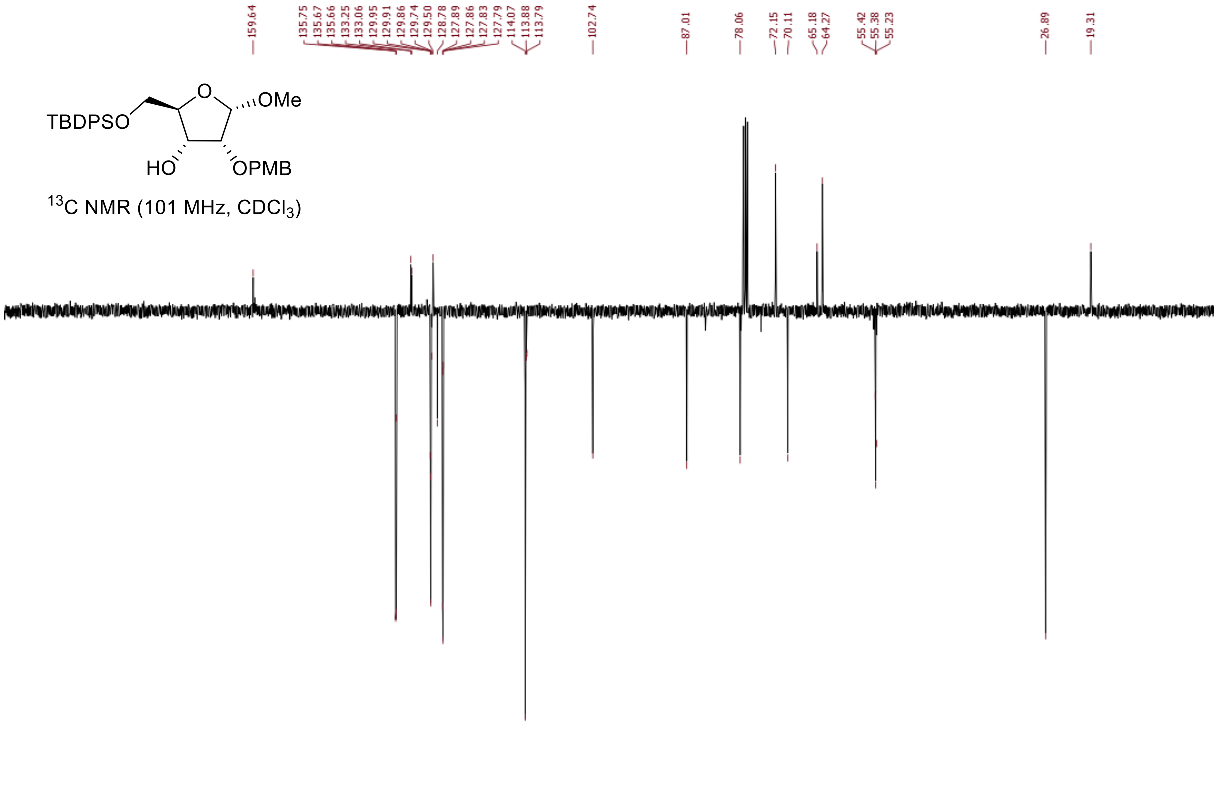

Compound **54**

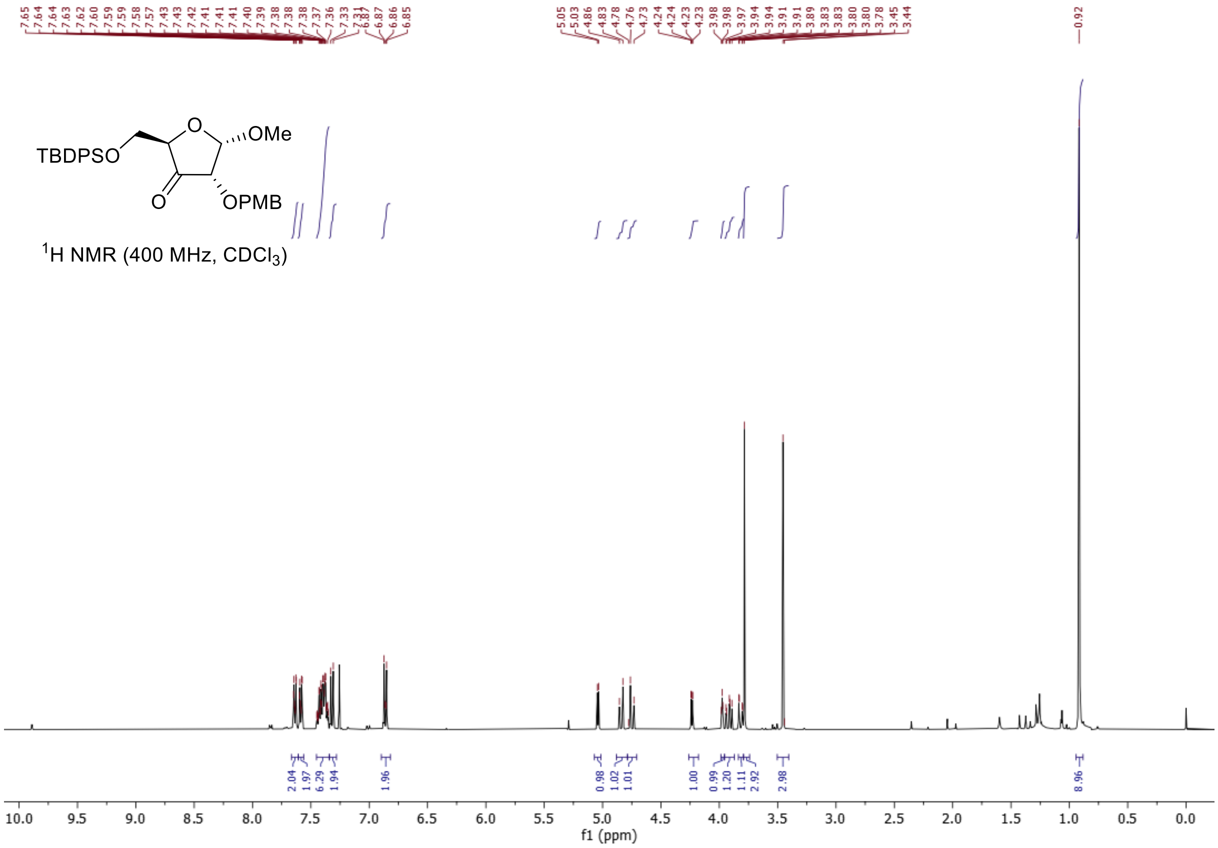
TBDPSO
OMe
OPMB
O
^{1}H NMR (400 MHz, CDCl$_3$)
f1 (ppm)

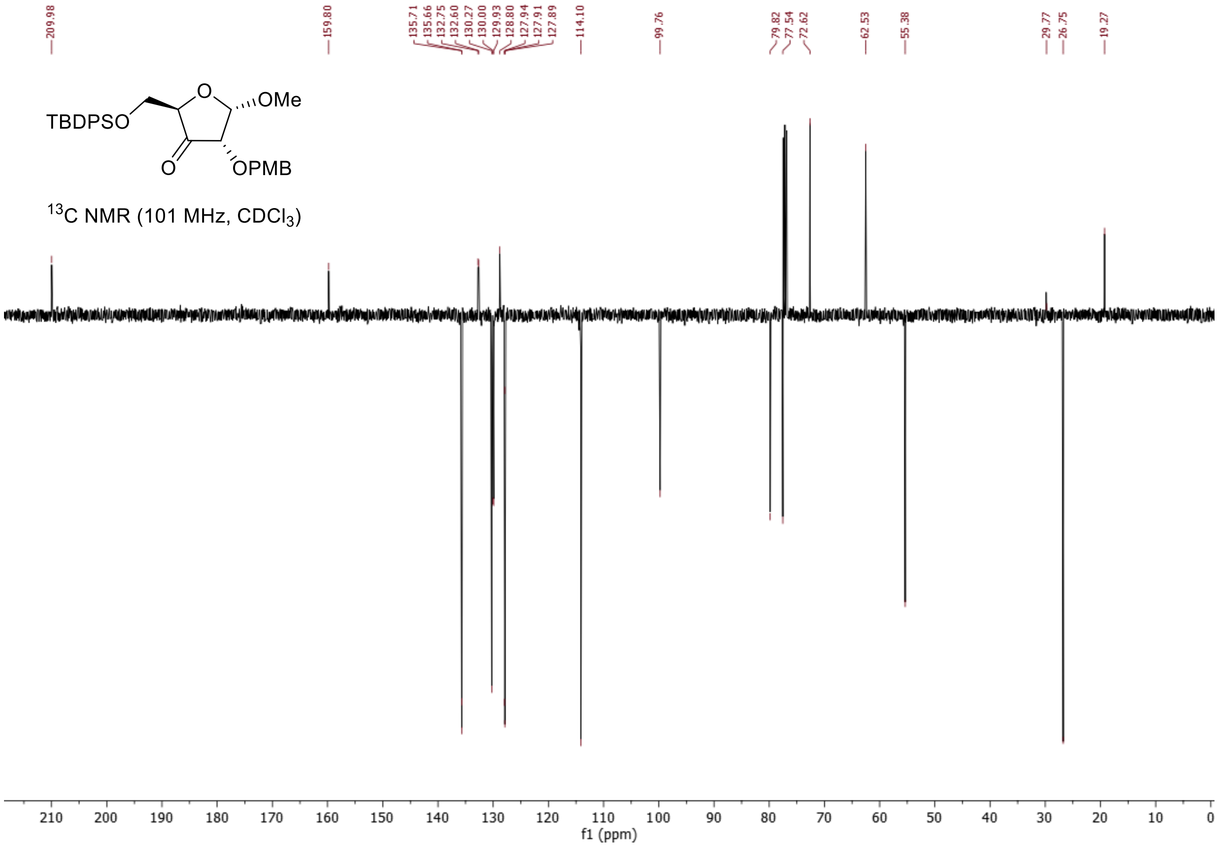
TBDPSO
OMe
OPMB
O
^{13}C NMR (101 MHz, CDCl$_3$)
f1 (ppm)

Compound **17**

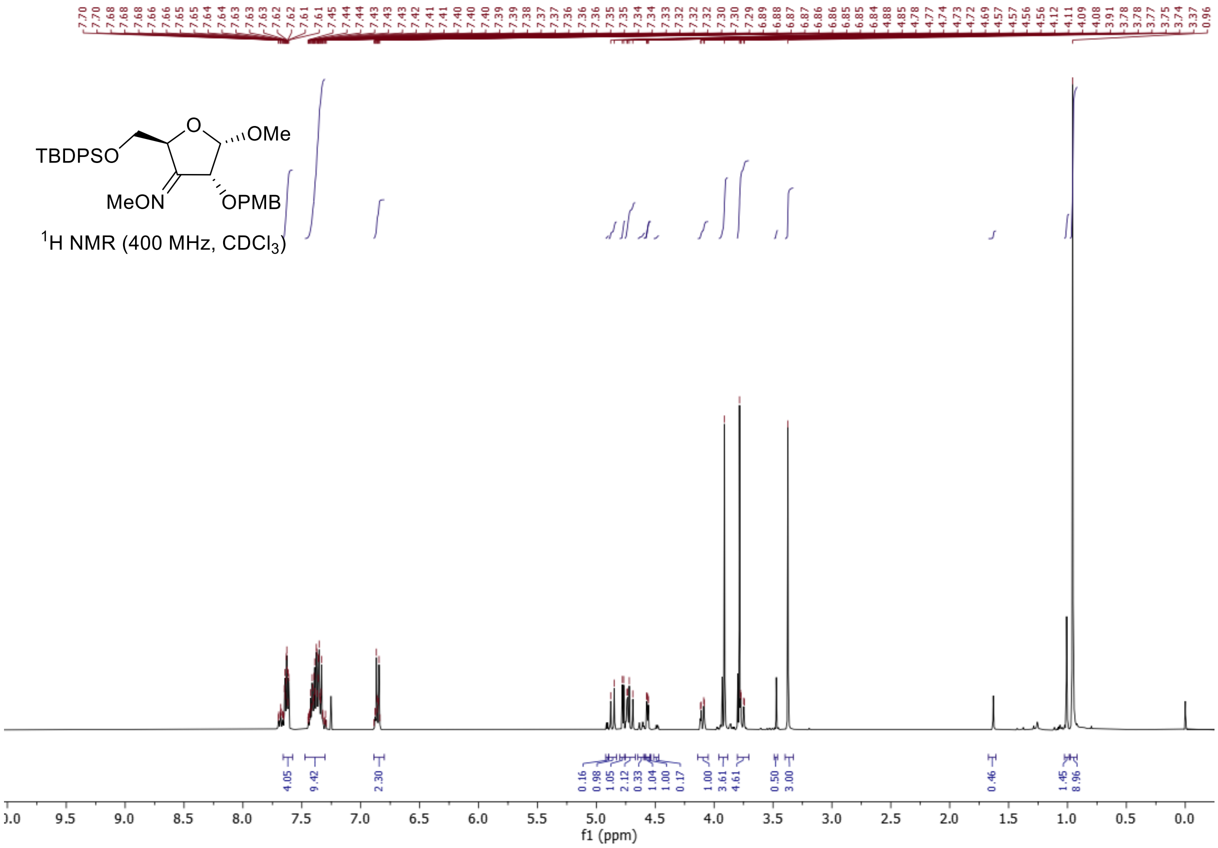

TBDPSO
OMe
MeON
OPMB
1H NMR (400 MHz, CDCl3)
f1 (ppm)

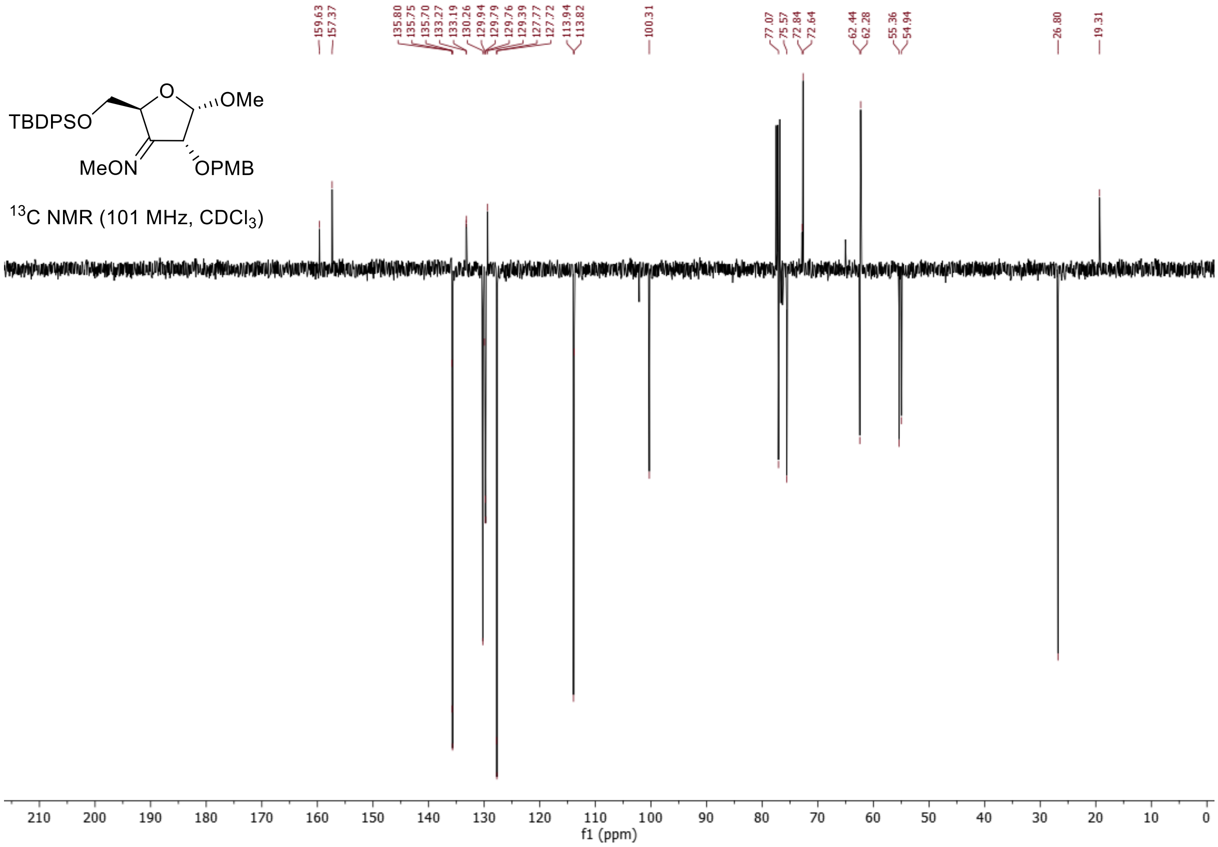

TBDPSO
OMe
MeON
OPMB
13C NMR (101 MHz, CDCl3)
f1 (ppm)

Compound **55**

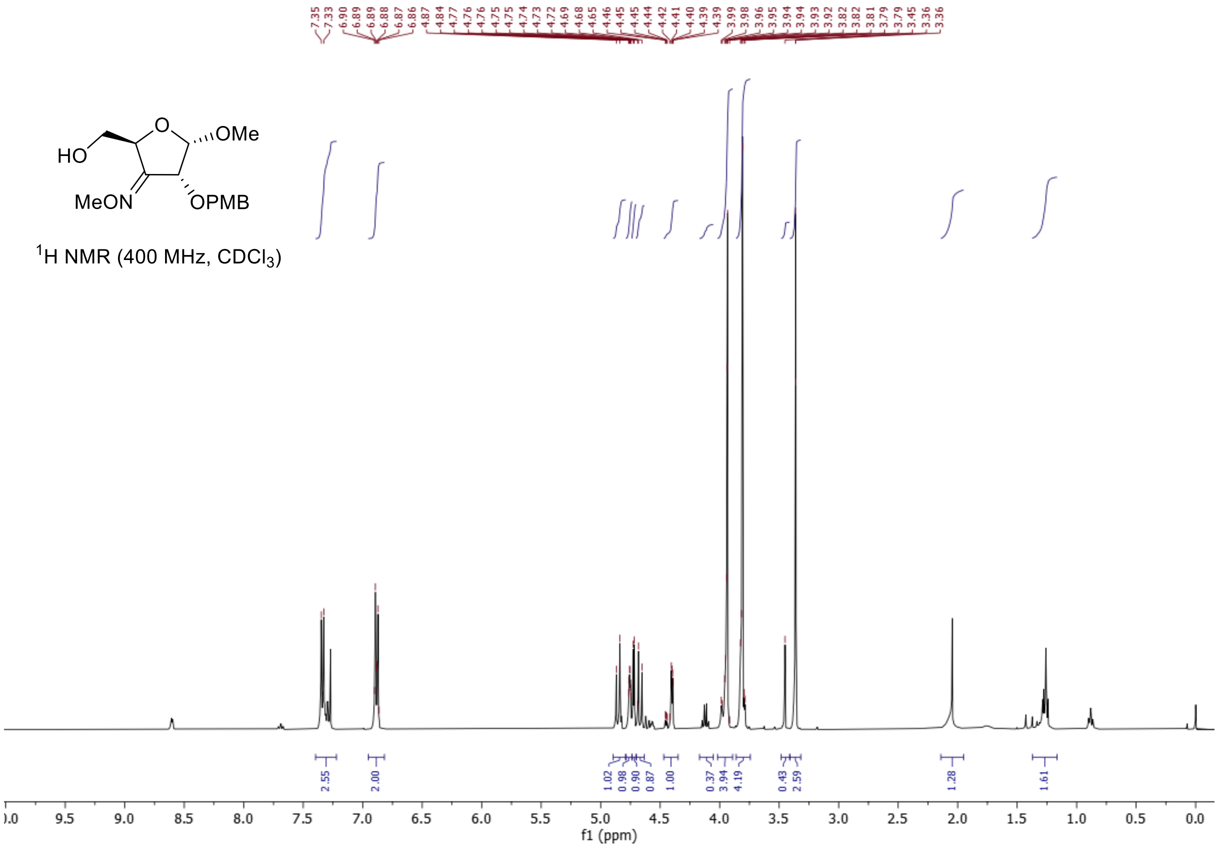
HO
OMe
MeON
OPMB
^{1}H NMR (400 MHz, $CDCl_3$)
f1 (ppm)

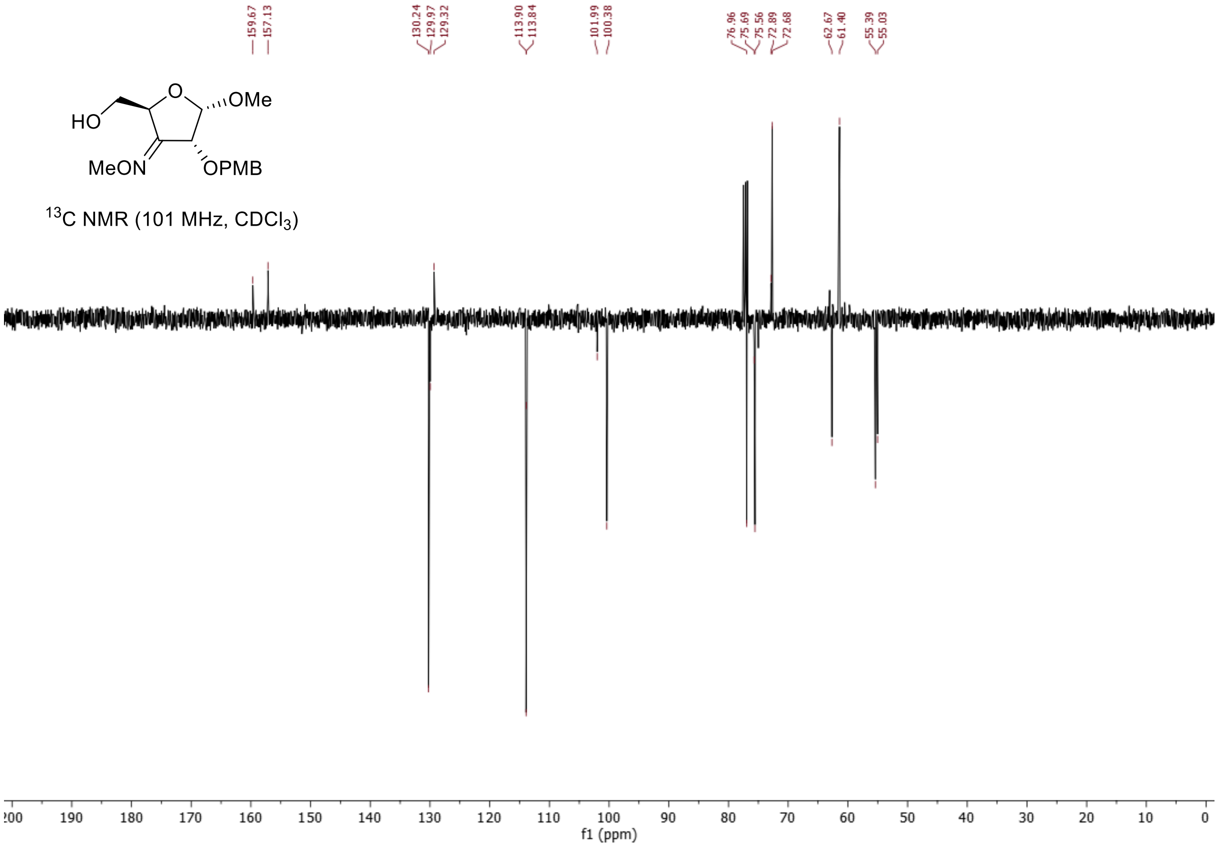
HO
OMe
MeON
OPMB
^{13}C NMR (101 MHz, $CDCl_3$)
f1 (ppm)

Compound **56**

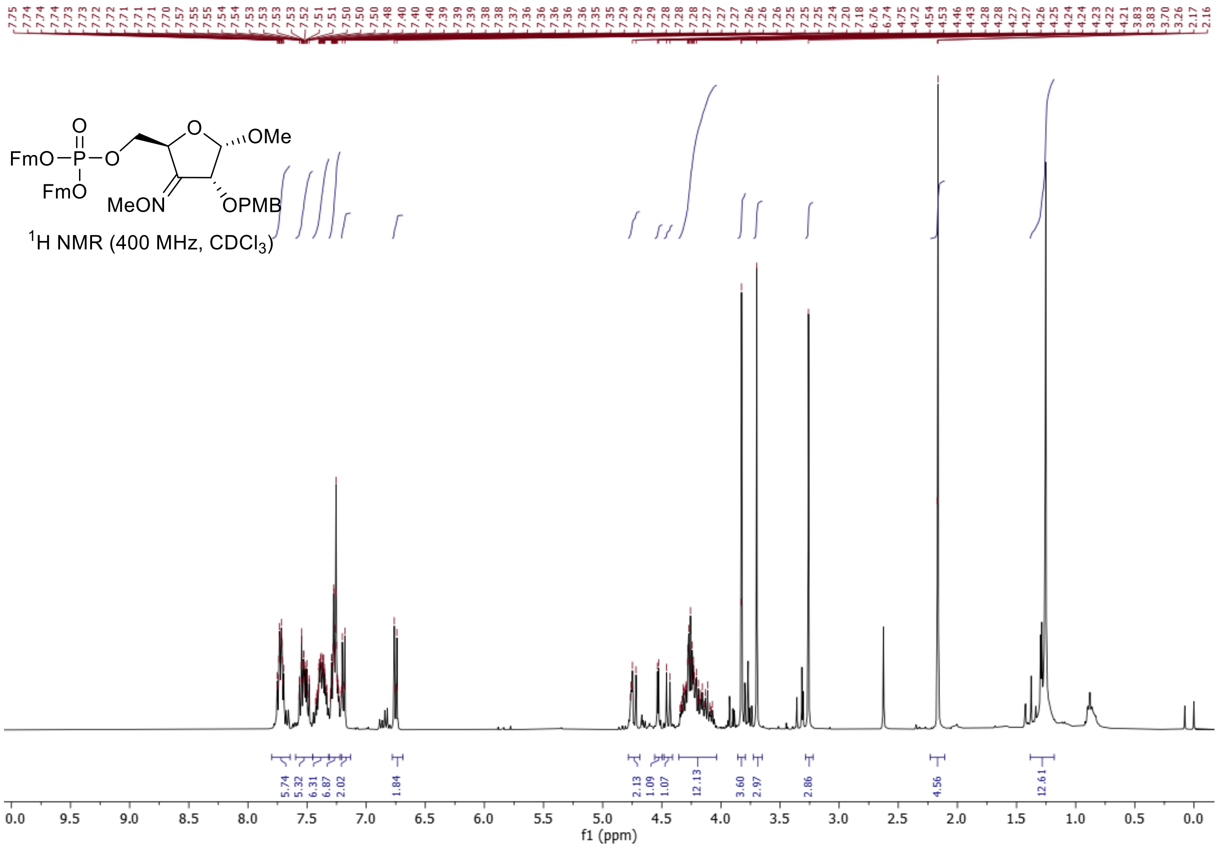

FmO
FmO
MeON
OMe
OPMB
^{1}H NMR (400 MHz, CDCl3)
f1 (ppm)

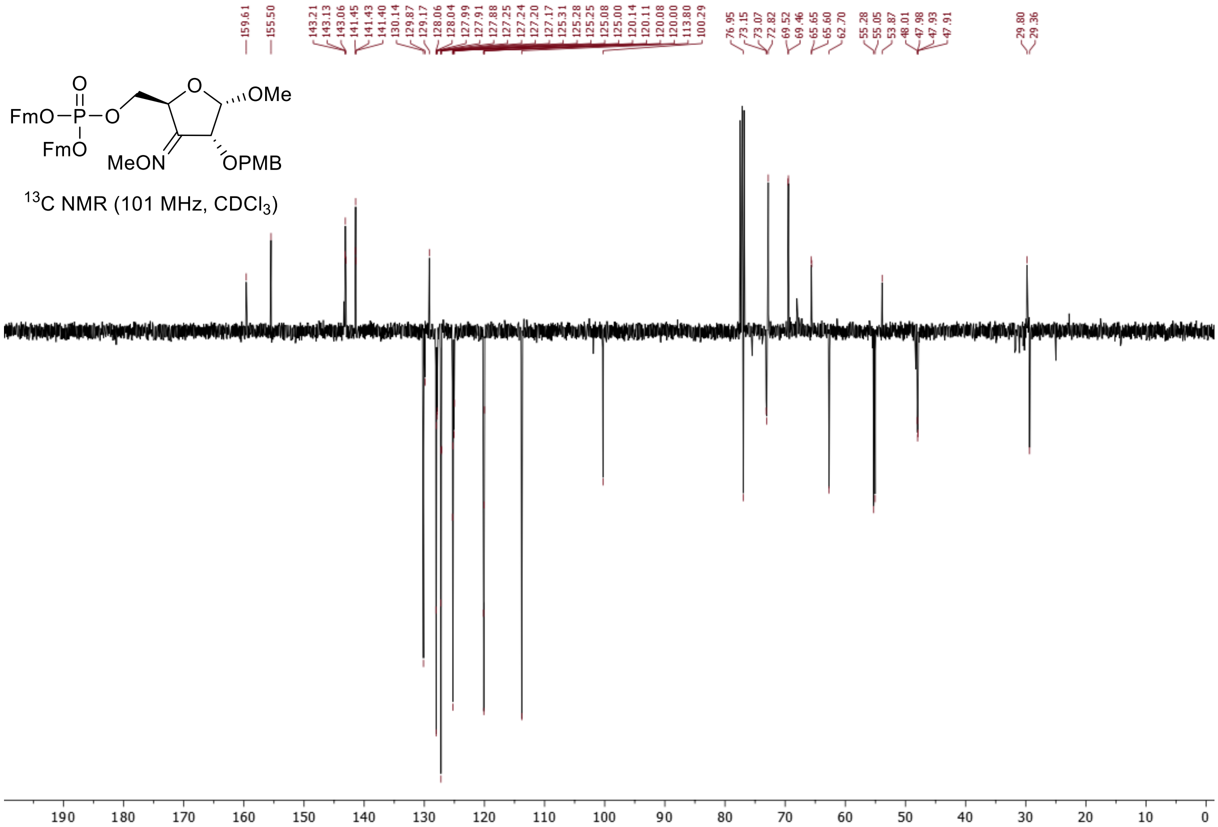

FmO
FmO
MeON
OMe
OPMB
^{13}C NMR (101 MHz, CDCl3)
f1 (ppm)

Compound **57**

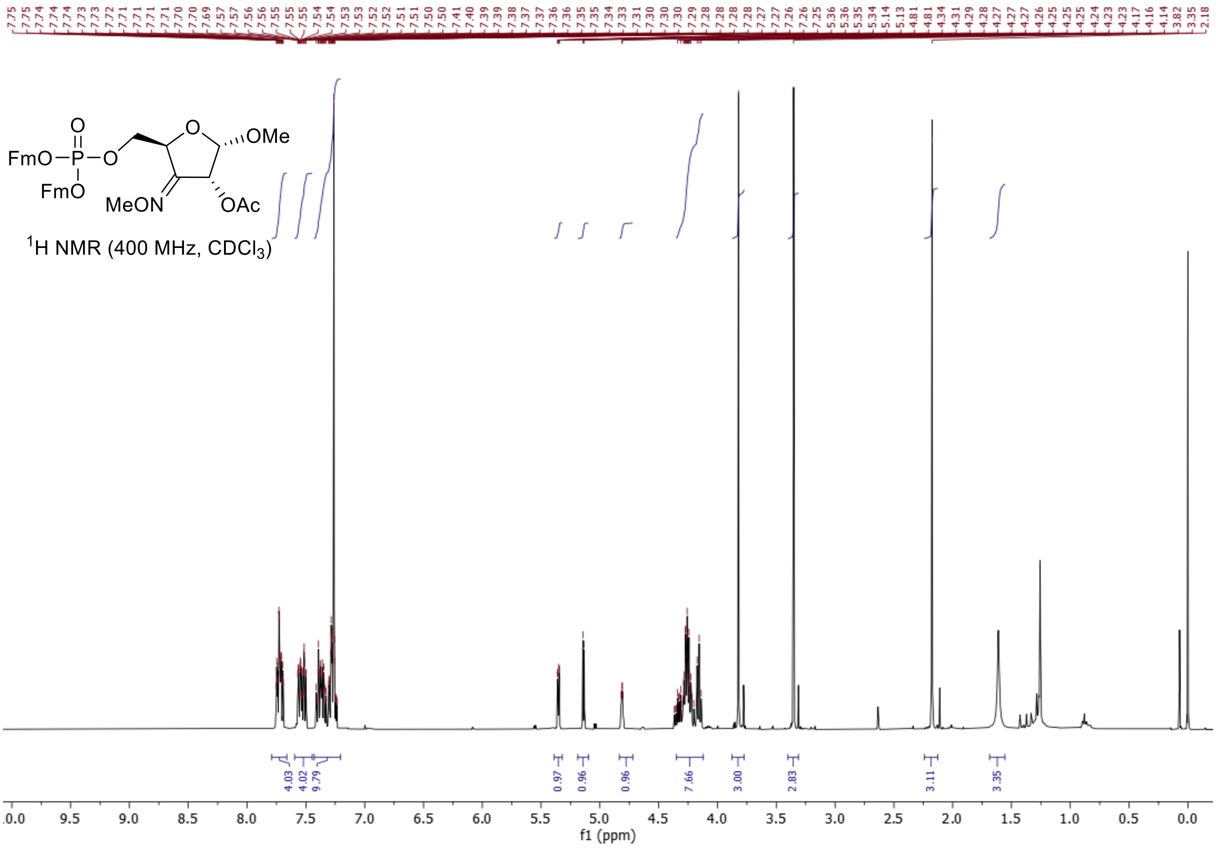
FmO–P–O
FmO
.,,OMe
MeON
OAc
1H NMR (400 MHz, CDCl3)
f1 (ppm)

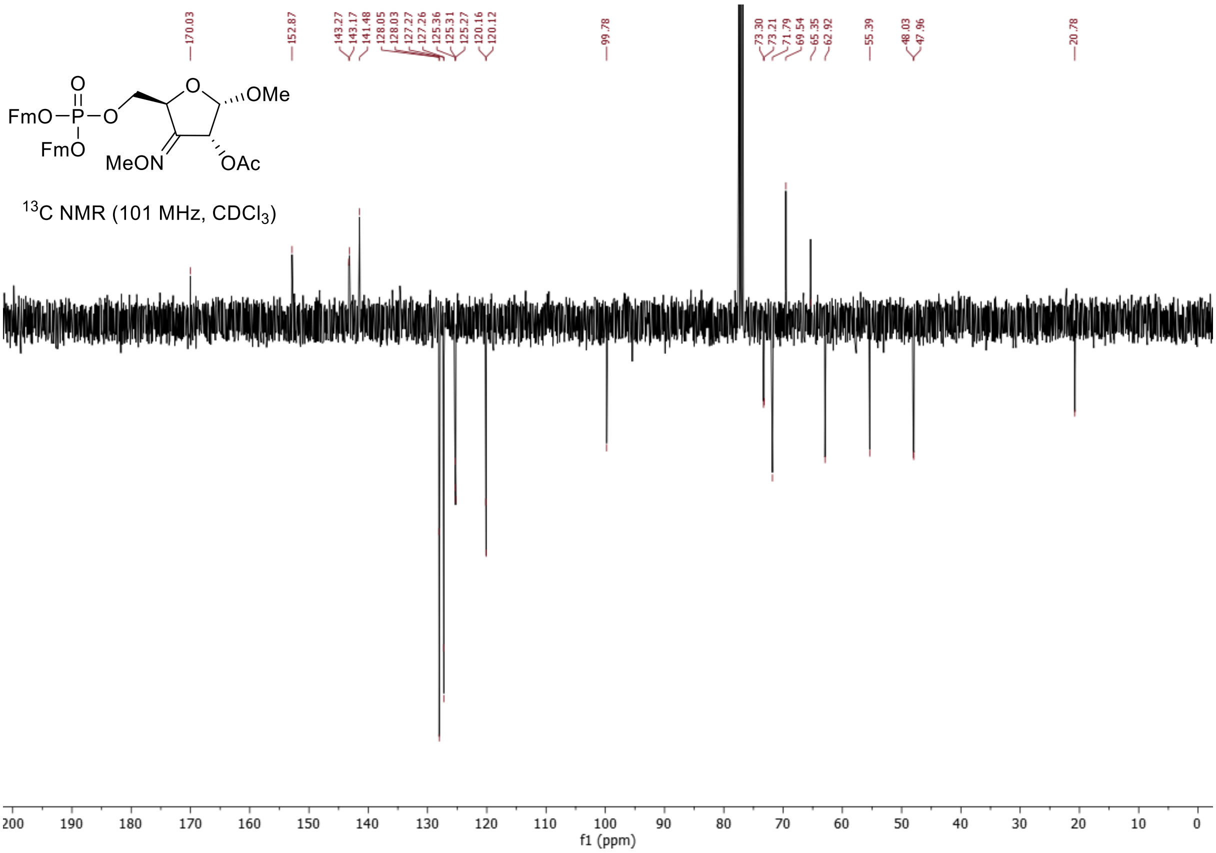
170.03
152.87
143.27
143.17
141.48
128.05
128.03
127.27
127.26
125.36
125.31
125.27
120.16
120.12
99.78
73.30
73.21
71.79
69.54
65.35
62.92
55.39
48.03
47.96
20.78
FmO–P–O
FmO
.,,OMe
MeON
OAc
13C NMR (101 MHz, CDCl3)
f1 (ppm)

Compound **9**

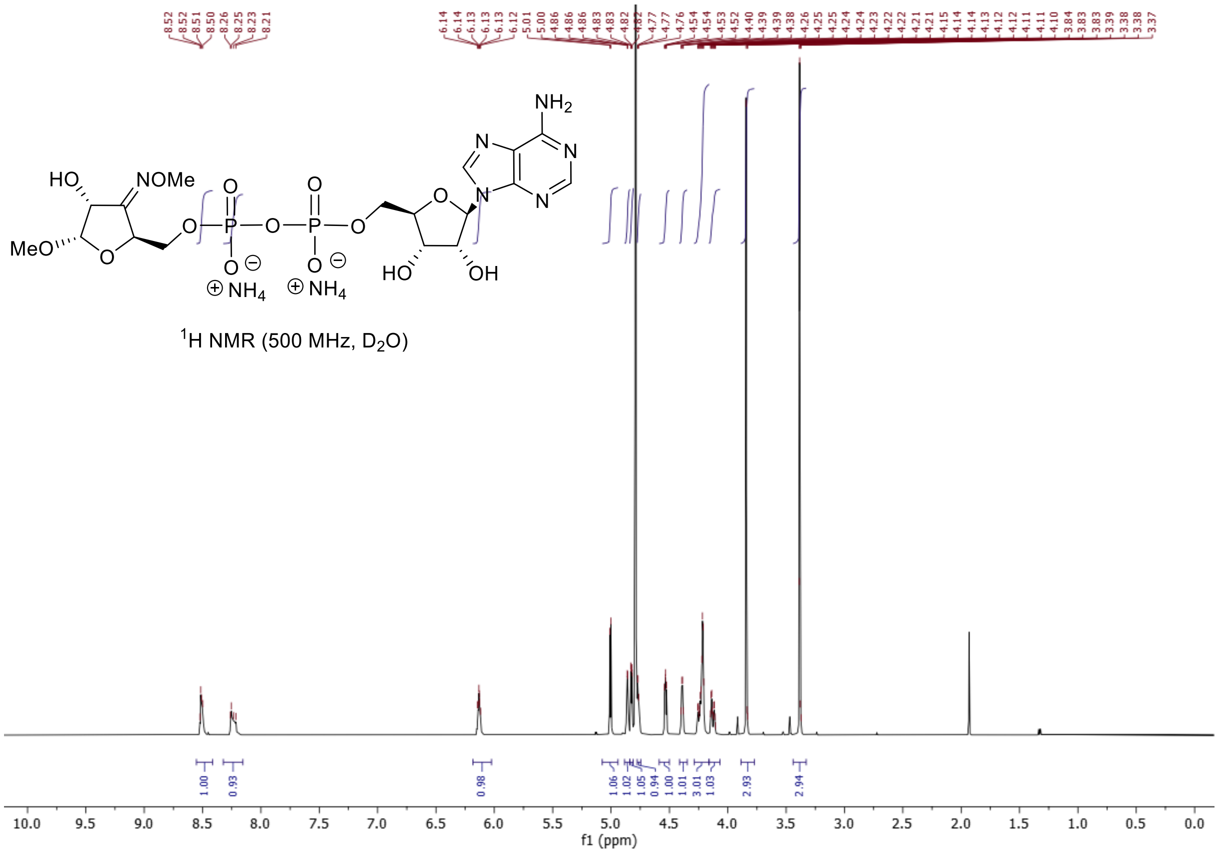
8.52 8.52 8.51 8.50 8.26 8.25 8.23 8.21
6.14 6.14 6.13 6.13 6.13 6.12 5.01 5.00 4.86 4.86 4.83 4.83 4.82 4.77 4.77 4.76 4.54 4.54 4.53 4.52 4.40 4.39 4.39 4.38 4.26 4.25 4.25 4.24 4.24 4.23 4.22 4.22 4.21 4.21 4.15 4.14 4.14 4.13 4.12 4.12 4.11 4.11 4.10 3.84 3.83 3.83 3.39 3.38 3.38 3.37
NH2
HO
NOMe
MeO
O
OH
NH4
1H NMR (500 MHz, D2O)
1.00 0.93 0.98 1.06 1.02 1.05 0.94 1.00 1.01 3.01 1.03 2.93 2.94
10.0 9.5 9.0 8.5 8.0 7.5 7.0 6.5 6.0 5.5 5.0 4.5 4.0 3.5 3.0 2.5 2.0 1.5 1.0 0.5 0.0
f1 (ppm)

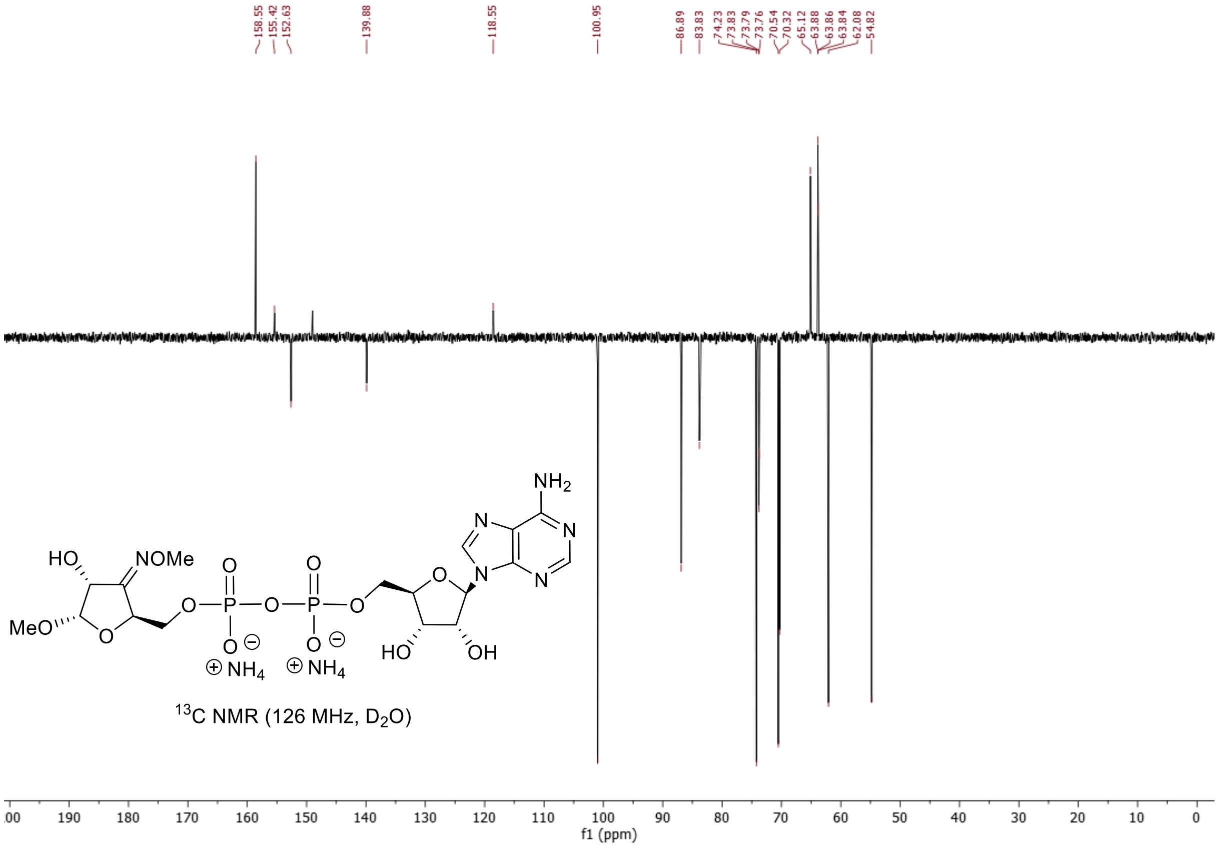
158.55 155.42 152.63 139.88 118.55 100.95 86.89 83.83 74.23 73.83 73.79 73.76 70.54 70.32 65.12 63.88 63.86 63.84 62.08 54.82
NH2
HO
NOMe
MeO
O
OH
NH4
13C NMR (126 MHz, D2O)
190 180 170 160 150 140 130 120 110 100 90 80 70 60 50 40 30 20 10 0
f1 (ppm)

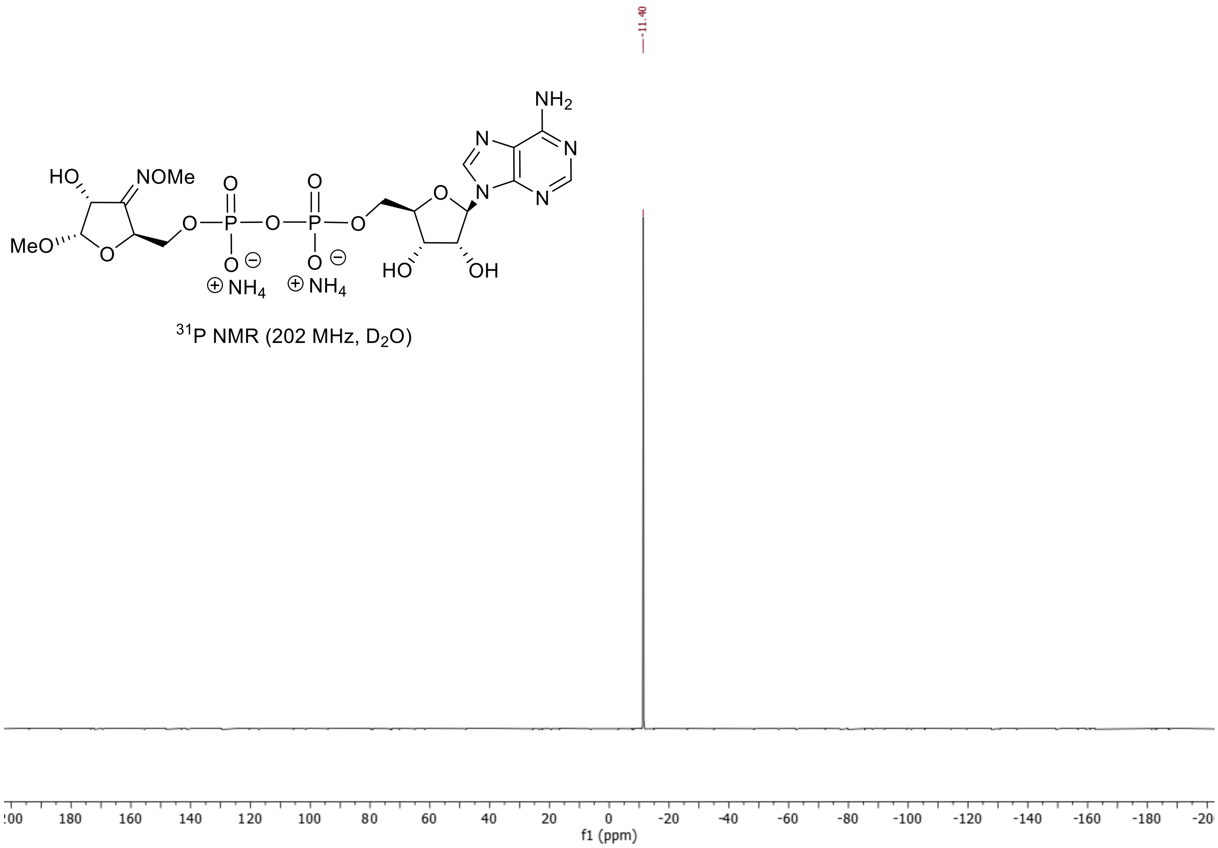
-11.40
NH2
HO
NOMe
MeO
O
P
NH4
HO
OH
31P NMR (202 MHz, D2O)
f1 (ppm)

Compound **59**

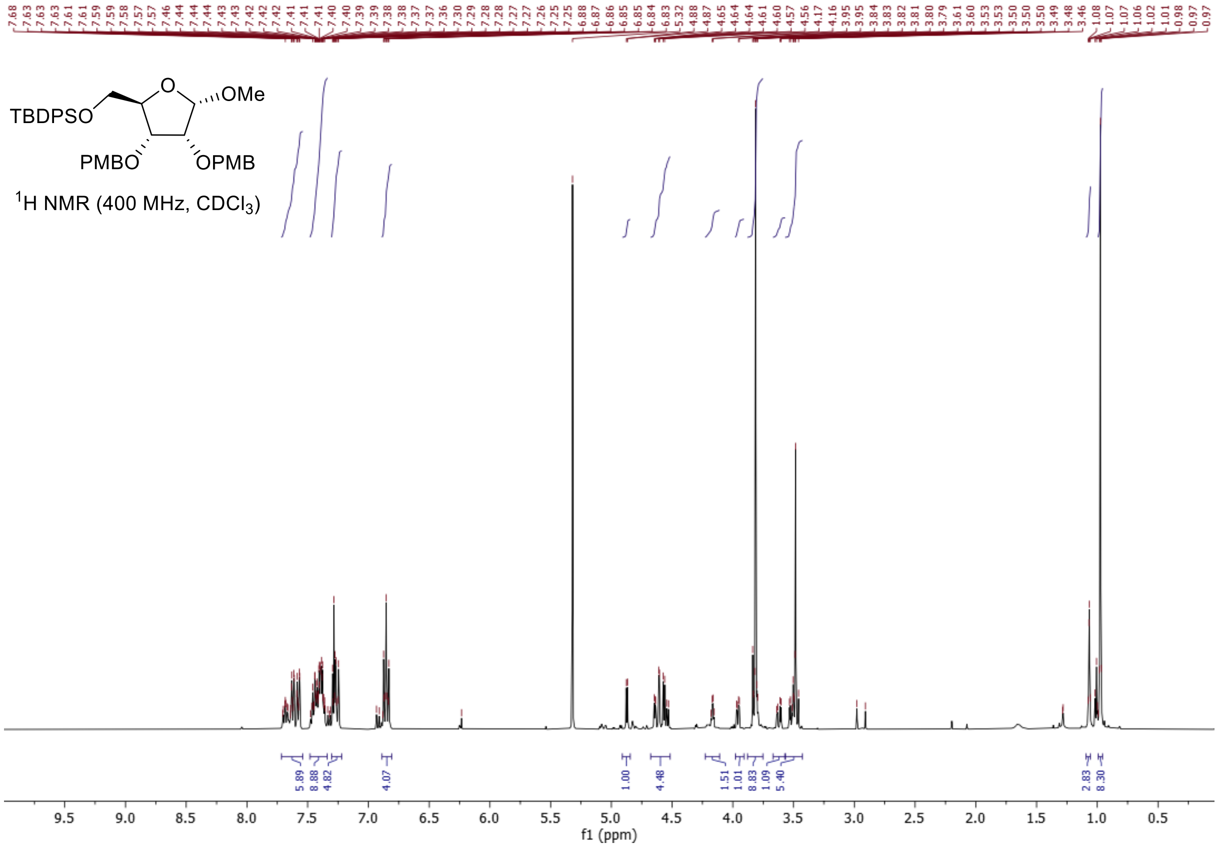
TBDPSO
OMe
PMBO
OPMB
1H NMR (400 MHz, CDCl3)
f1 (ppm)

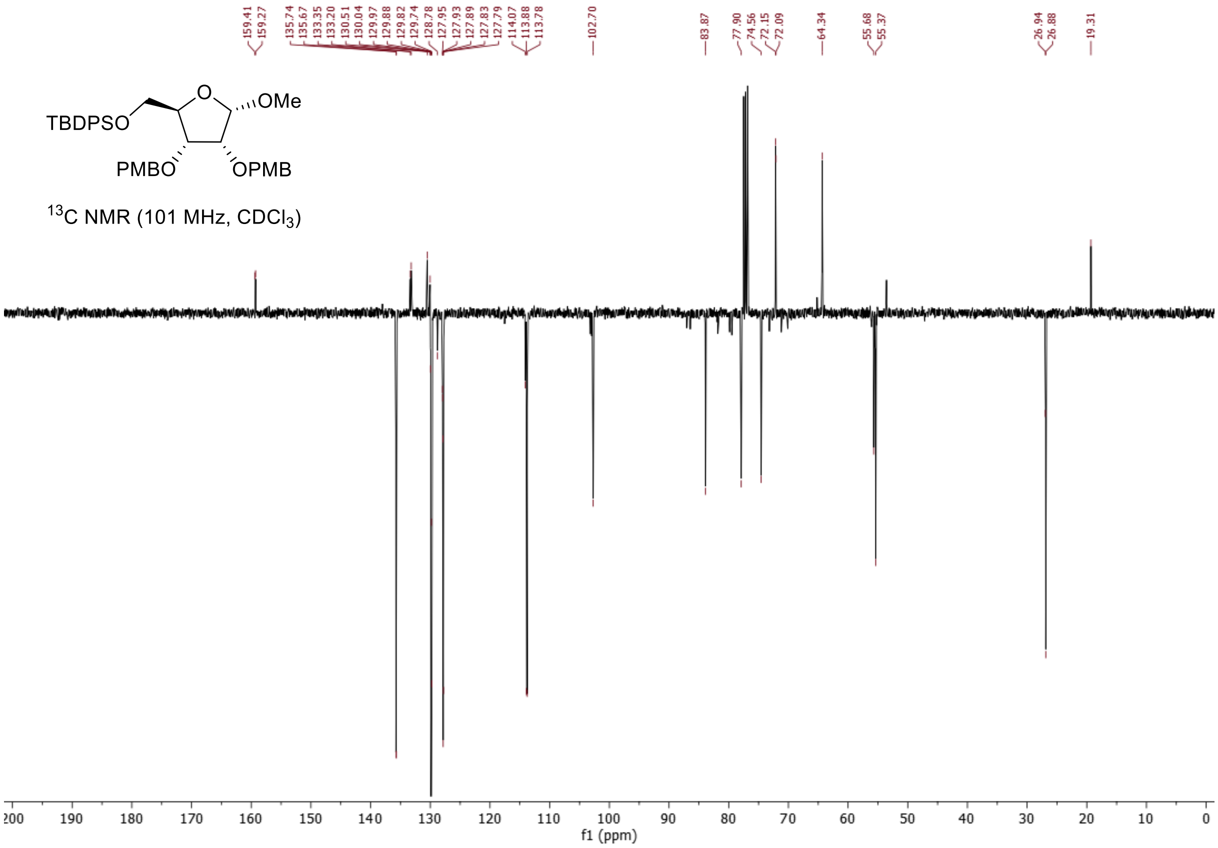
TBDPSO
OMe
PMBO
OPMB
13C NMR (101 MHz, CDCl3)
f1 (ppm)

Compound **60**

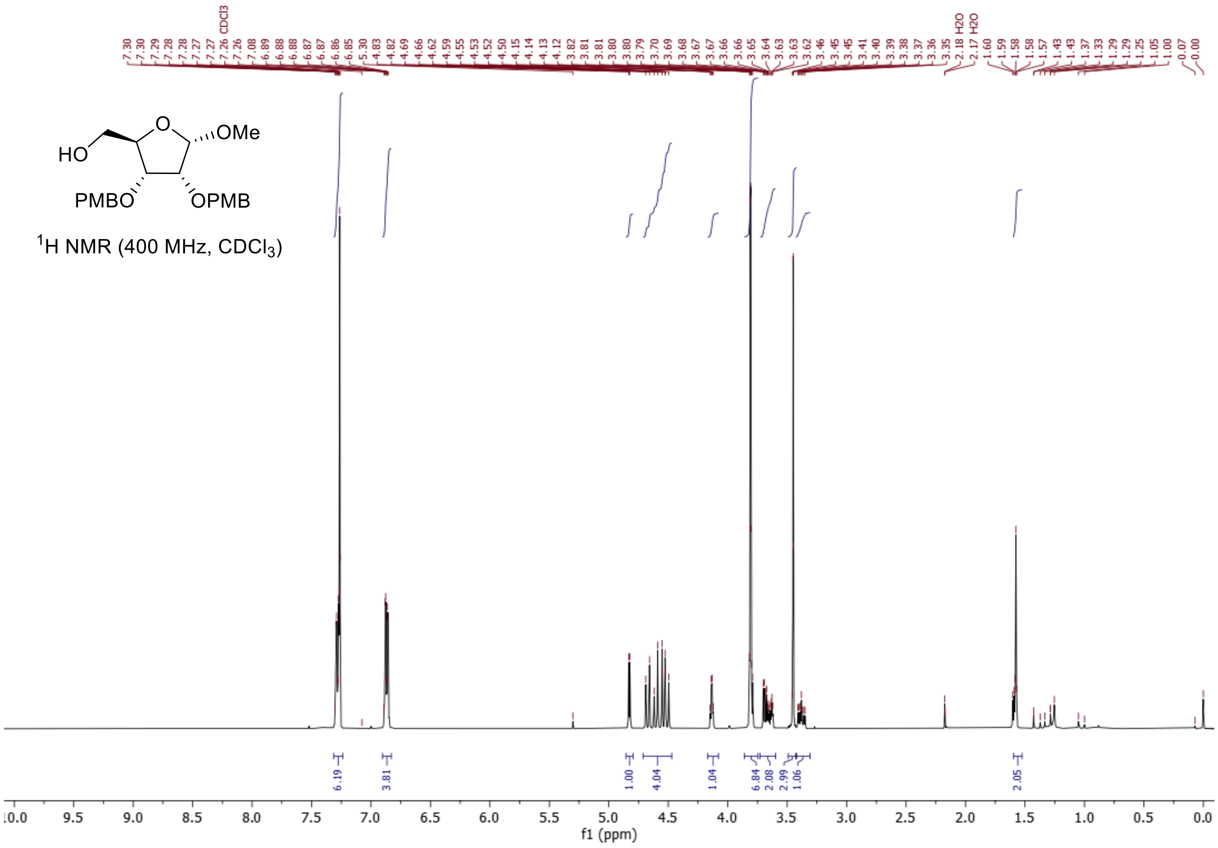
HO
OMe
PMBO
OPMB
1H NMR (400 MHz, CDCl3)
f1 (ppm)

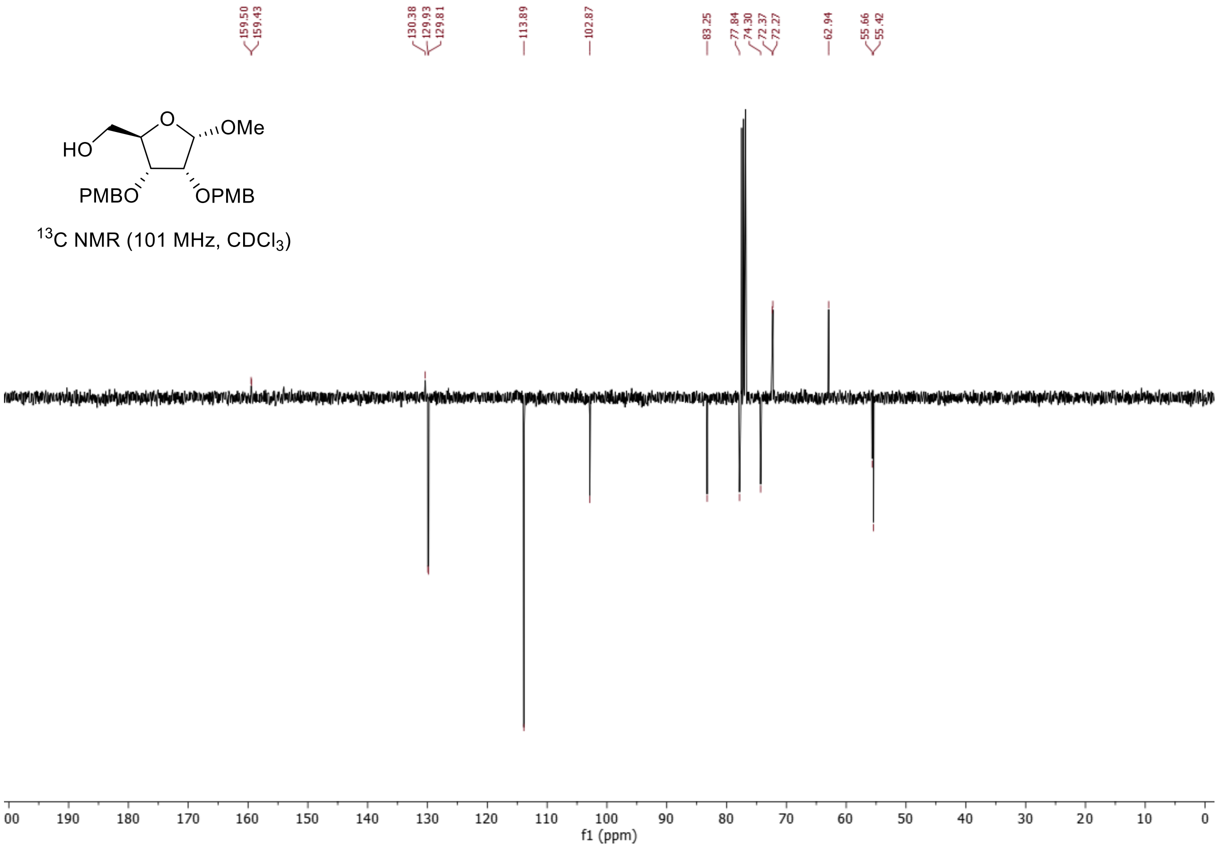
159.50
159.43
130.38
129.93
129.81
113.89
102.87
83.25
77.84
74.30
72.37
72.27
62.94
55.66
55.42
HO
O
OMe
PMBO
OPMB
13C NMR (101 MHz, CDCl3)
f1 (ppm)

Compound **61**

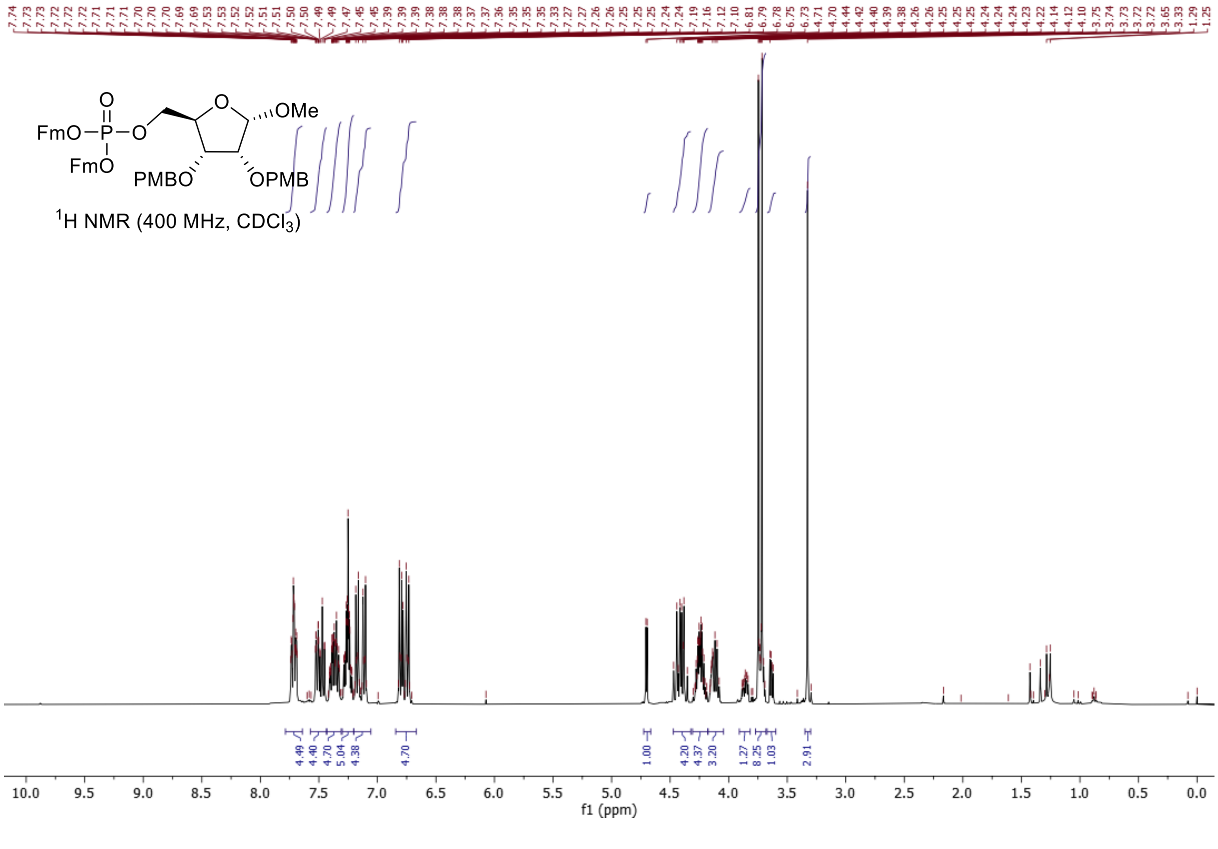
O
FmO
P
O
FmO
O
OMe
PMBO
OPMB
1H NMR (400 MHz, CDCl3)
f1 (ppm)

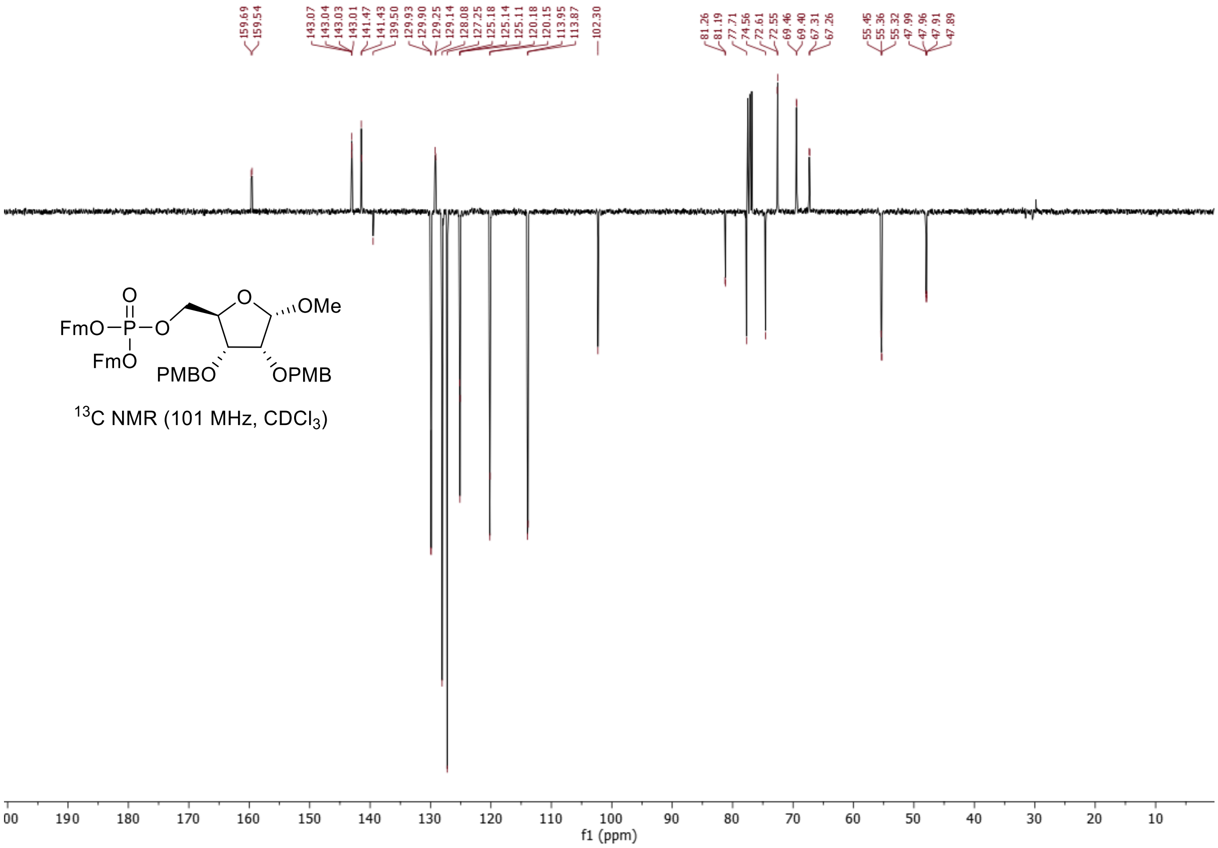
159.69
159.54
143.07
143.04
143.03
143.01
141.47
141.43
139.50
129.93
129.90
129.25
129.14
128.08
127.25
125.18
125.14
125.11
120.18
120.15
113.95
113.87
102.30
81.26
81.19
77.71
74.56
72.61
72.55
69.46
69.40
67.31
67.26
55.45
55.36
55.32
47.99
47.96
47.91
47.89
FmO
FmO
PMBO
OPMB
OMe
^{13}C NMR (101 MHz, $CDCl_3$)
f1 (ppm)

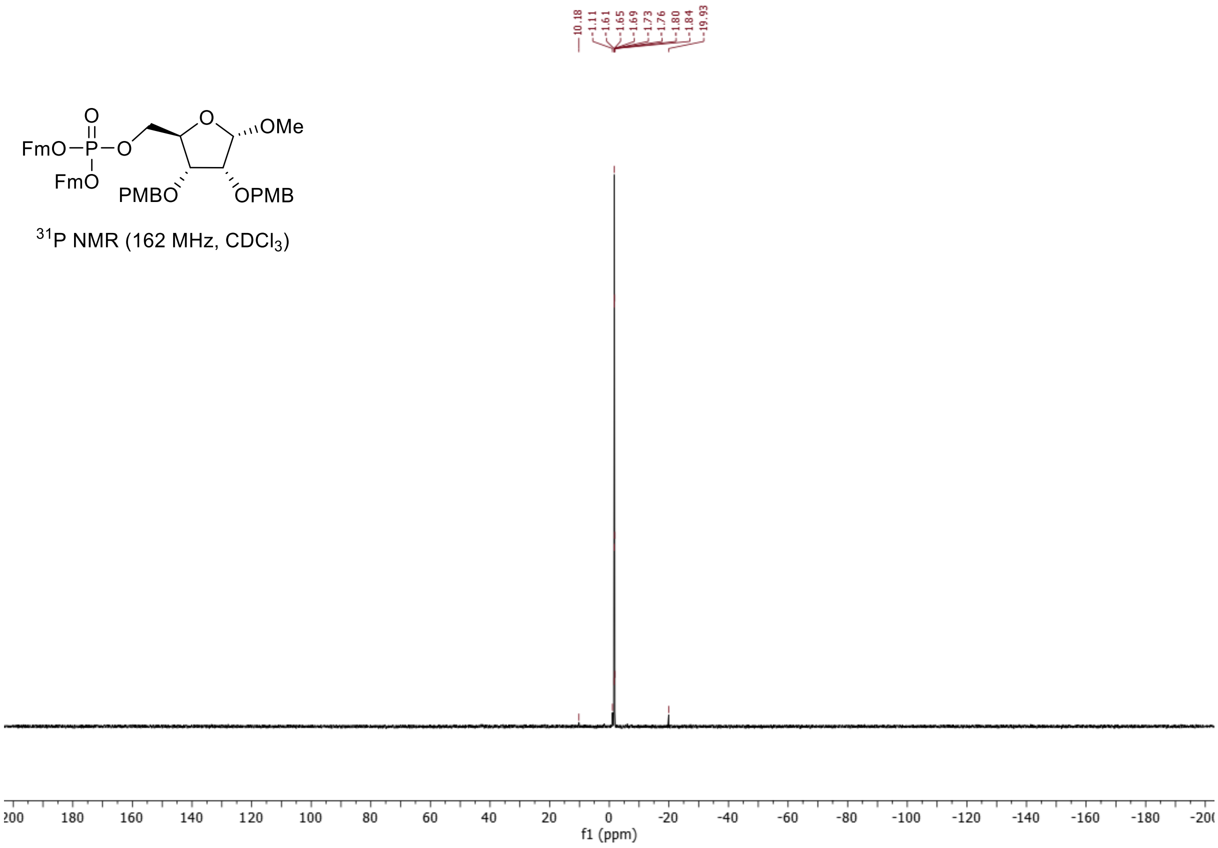
10.18
-1.11
-1.61
-1.65
-1.69
-1.73
-1.76
-1.80
-1.84
-19.93
FmO
FmO
PMBO
OPMB
OMe
^{31}P NMR (162 MHz, $CDCl_3$)
f1 (ppm)

Compound **63**

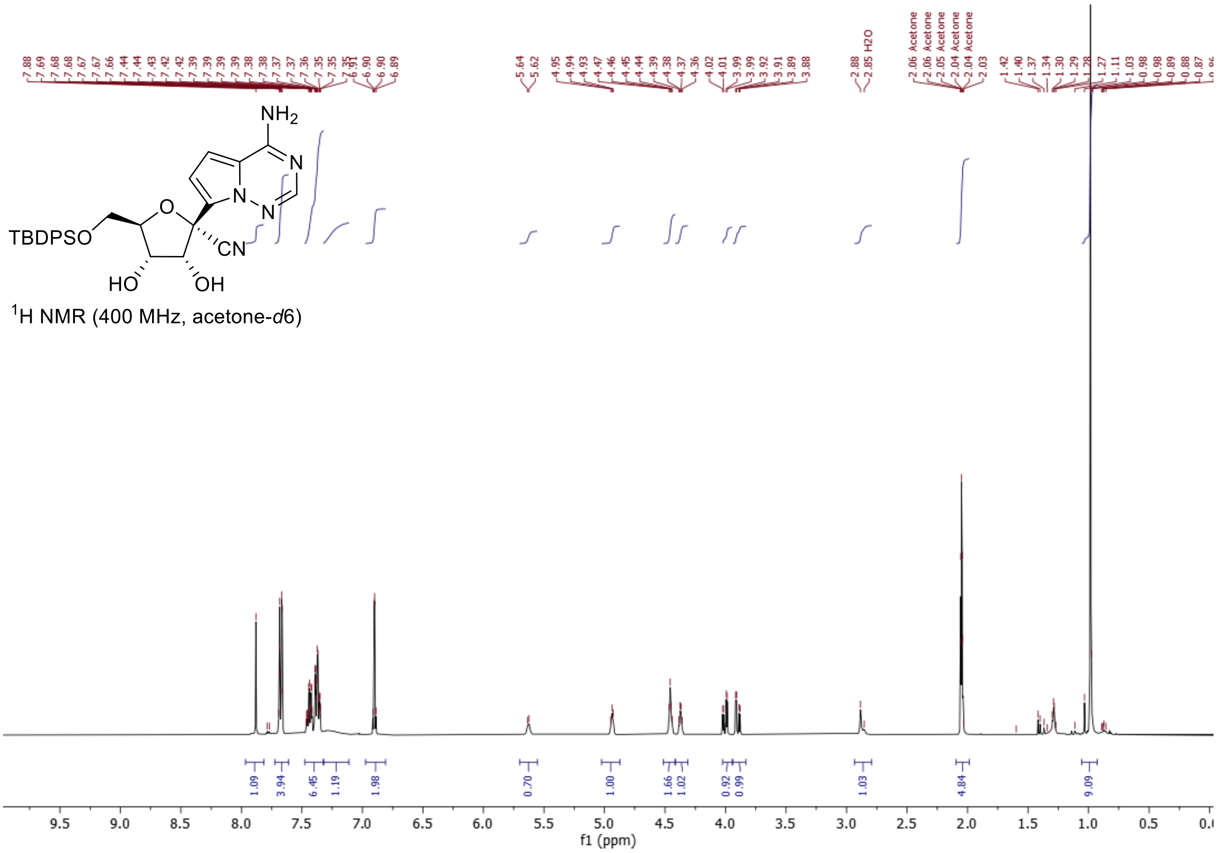

TBDPSO
NH2
CN
HO
OH
1H NMR (400 MHz, acetone-d6)
f1 (ppm)

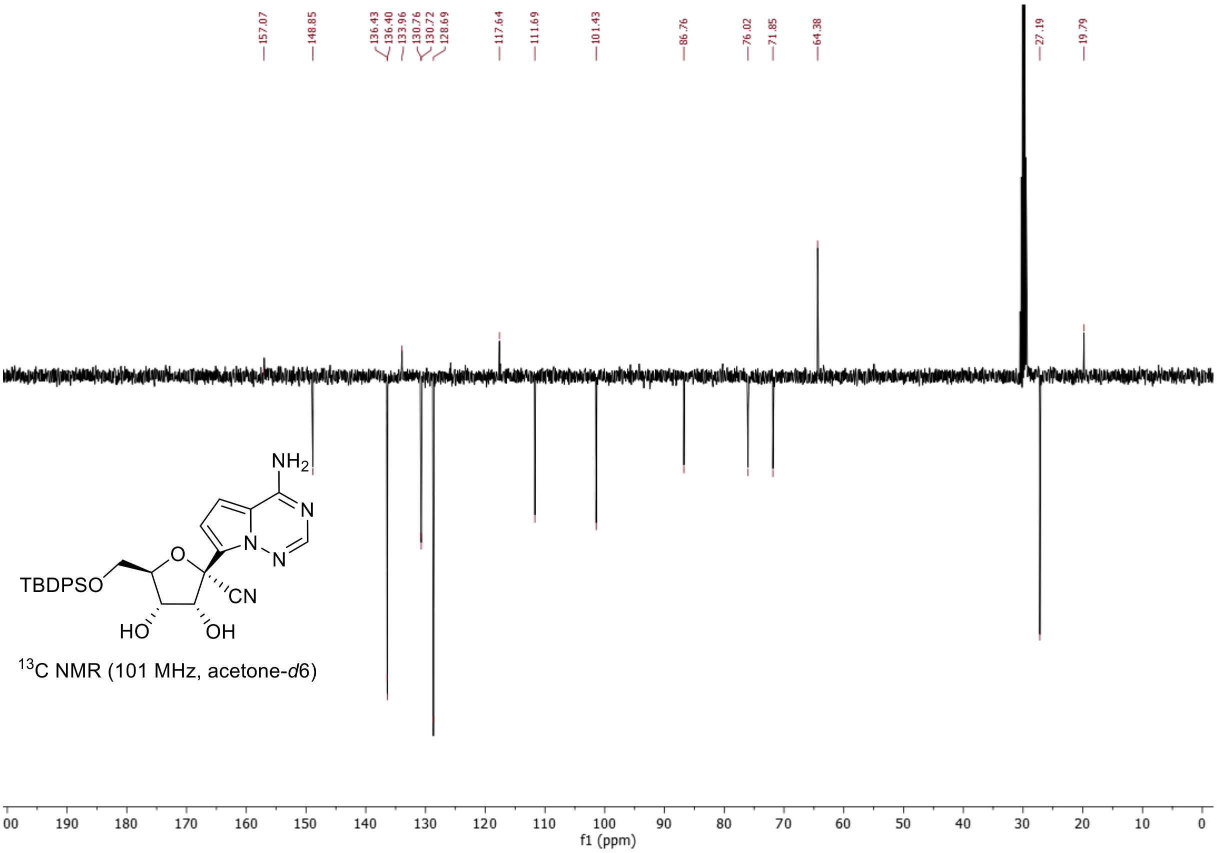

TBDPSO
NH2
CN
HO
OH
13C NMR (101 MHz, acetone-d6)
f1 (ppm)

Compound **64**

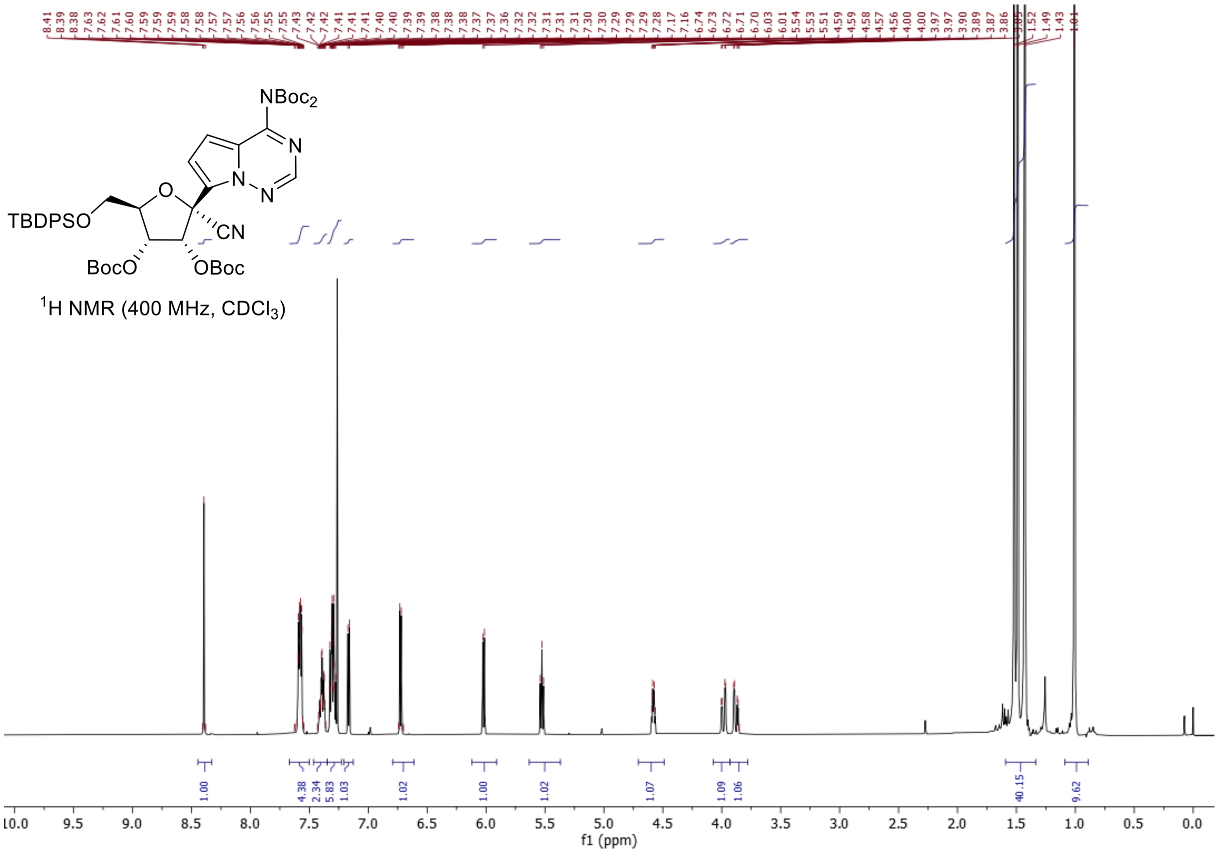
NBoc2
N
N
N
O
TBDPSO
CN
BocO
OBoc
1H NMR (400 MHz, CDCl3)
f1 (ppm)

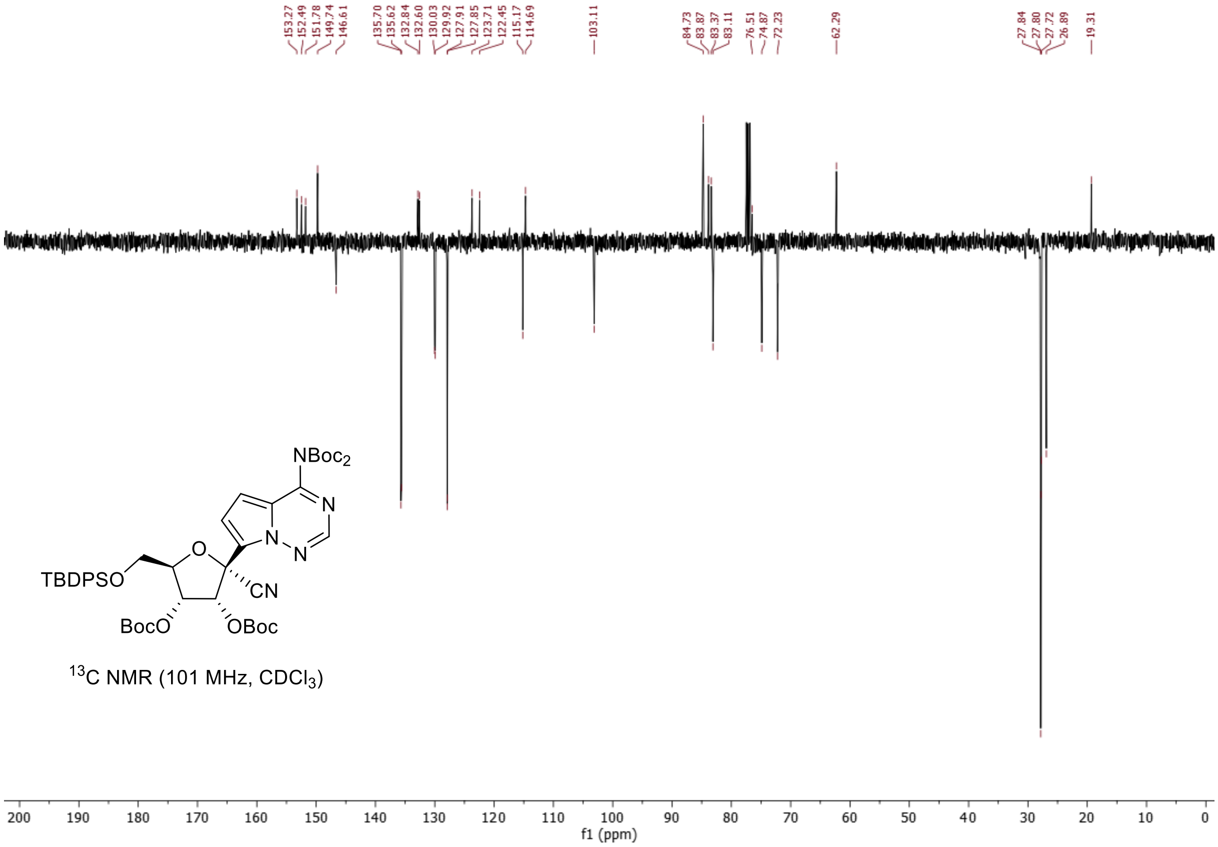
NBoc2
N
N
N
O
TBDPSO
CN
BocO
OBoc
13C NMR (101 MHz, CDCl3)
f1 (ppm)

Compound **65**

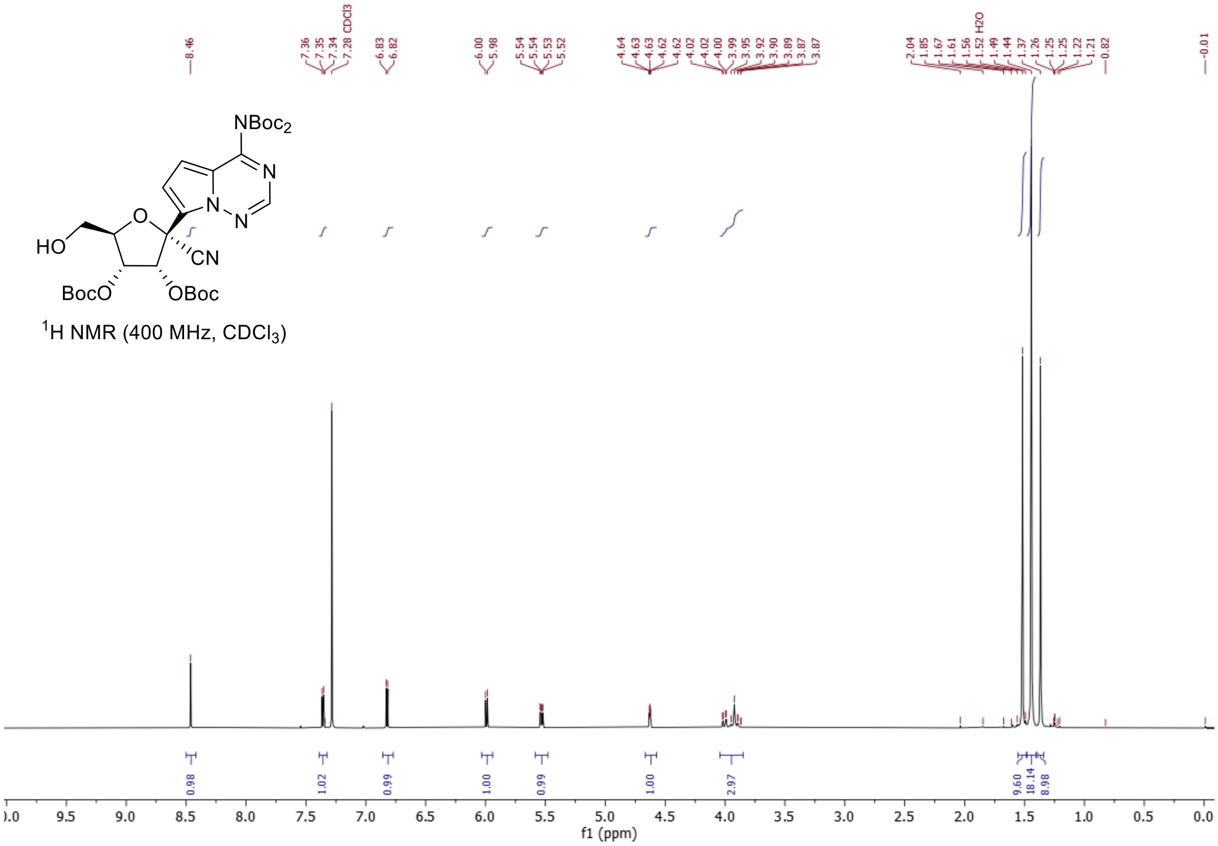

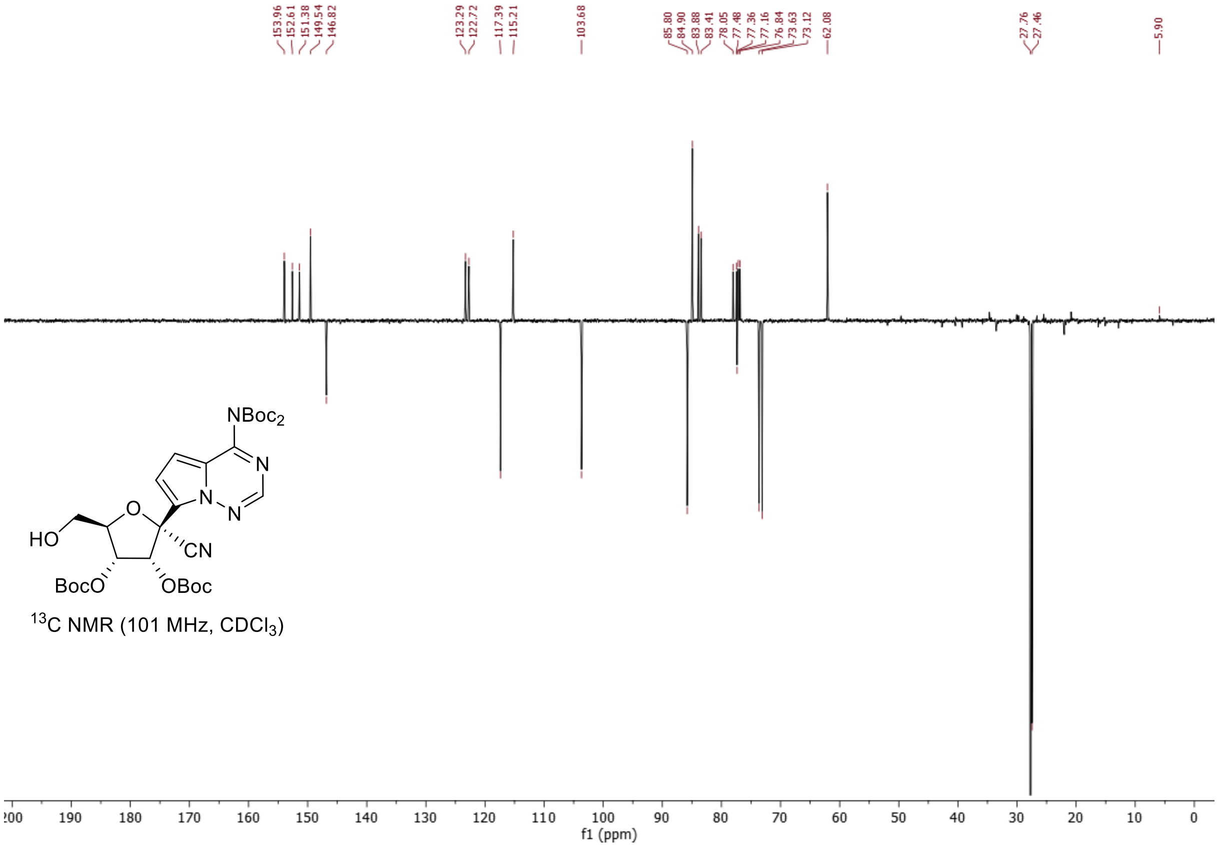

Compound **22**

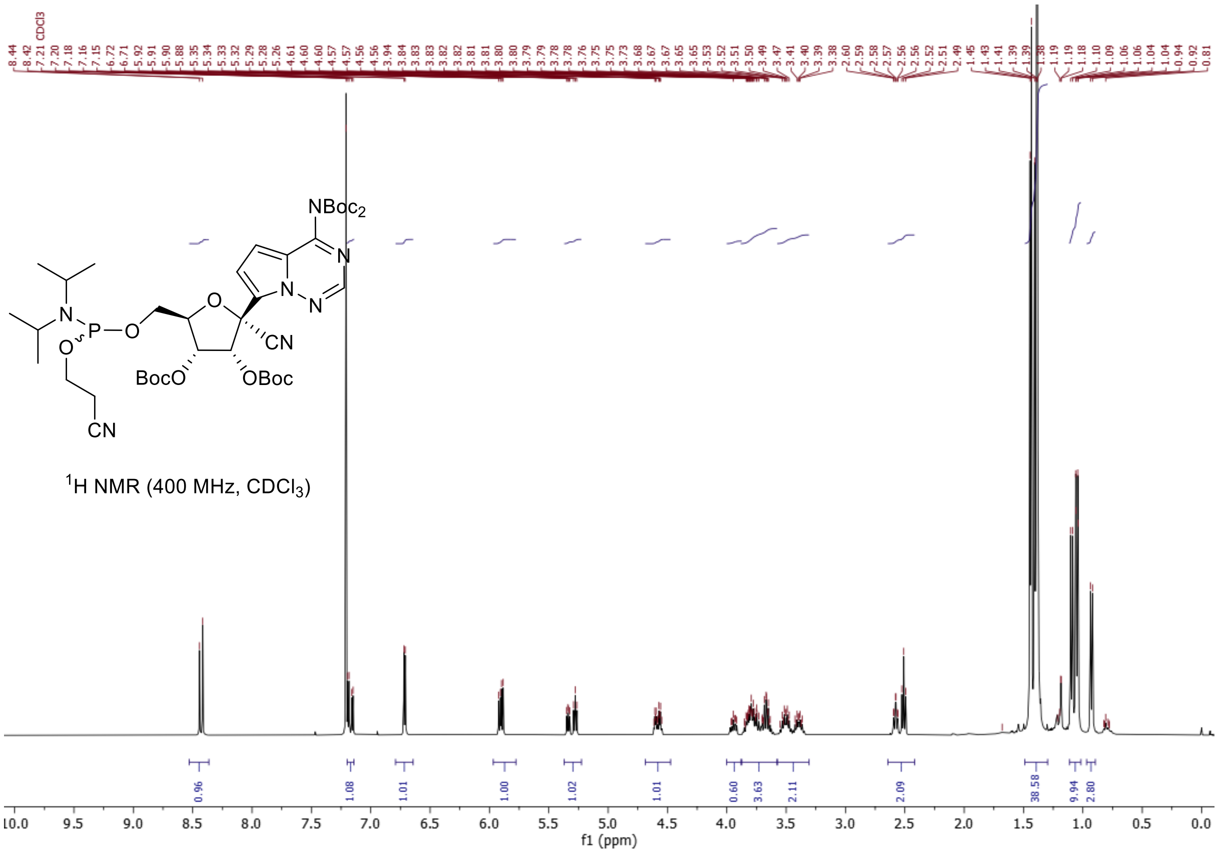

NBoc2
CN
BocO
OBoc
CN
1H NMR (400 MHz, CDCl3)
f1 (ppm)

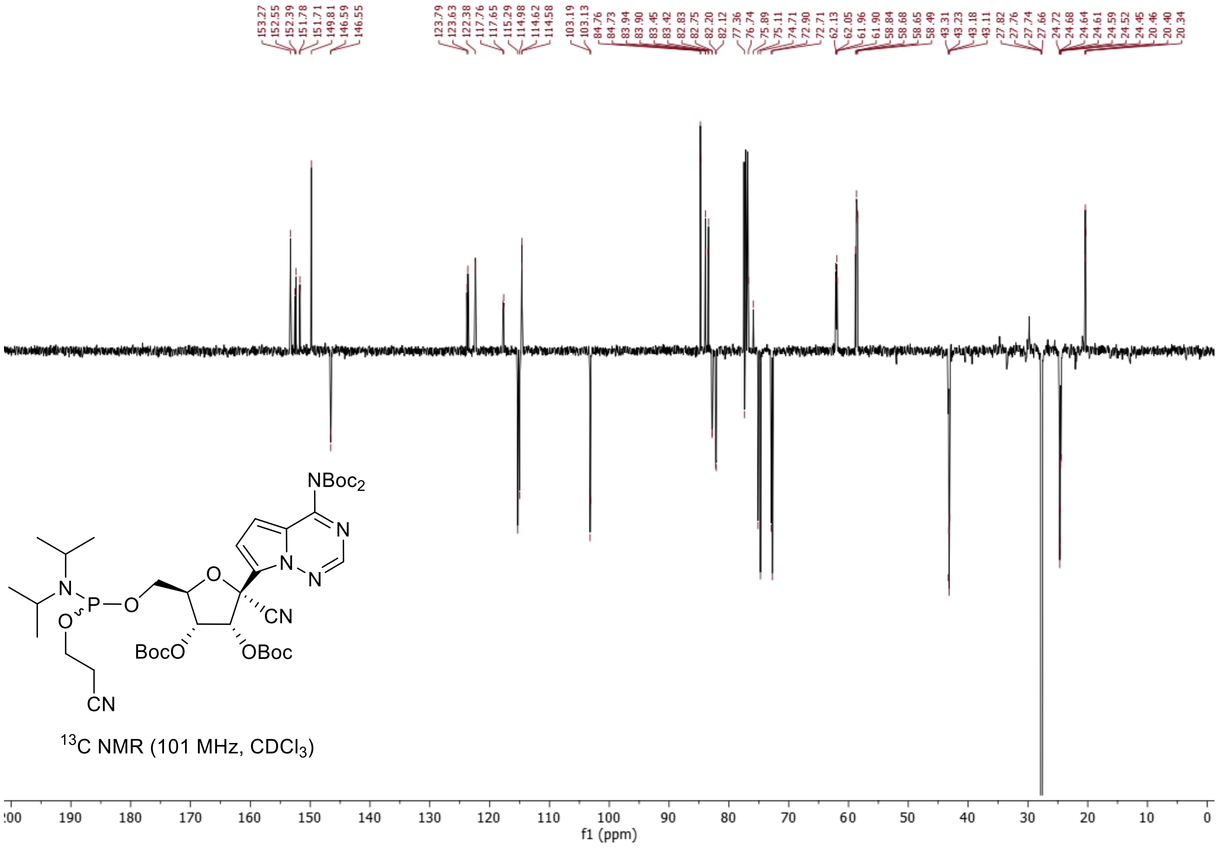

NBoc2
CN
BocO
OBoc
CN
13C NMR (101 MHz, CDCl3)
f1 (ppm)

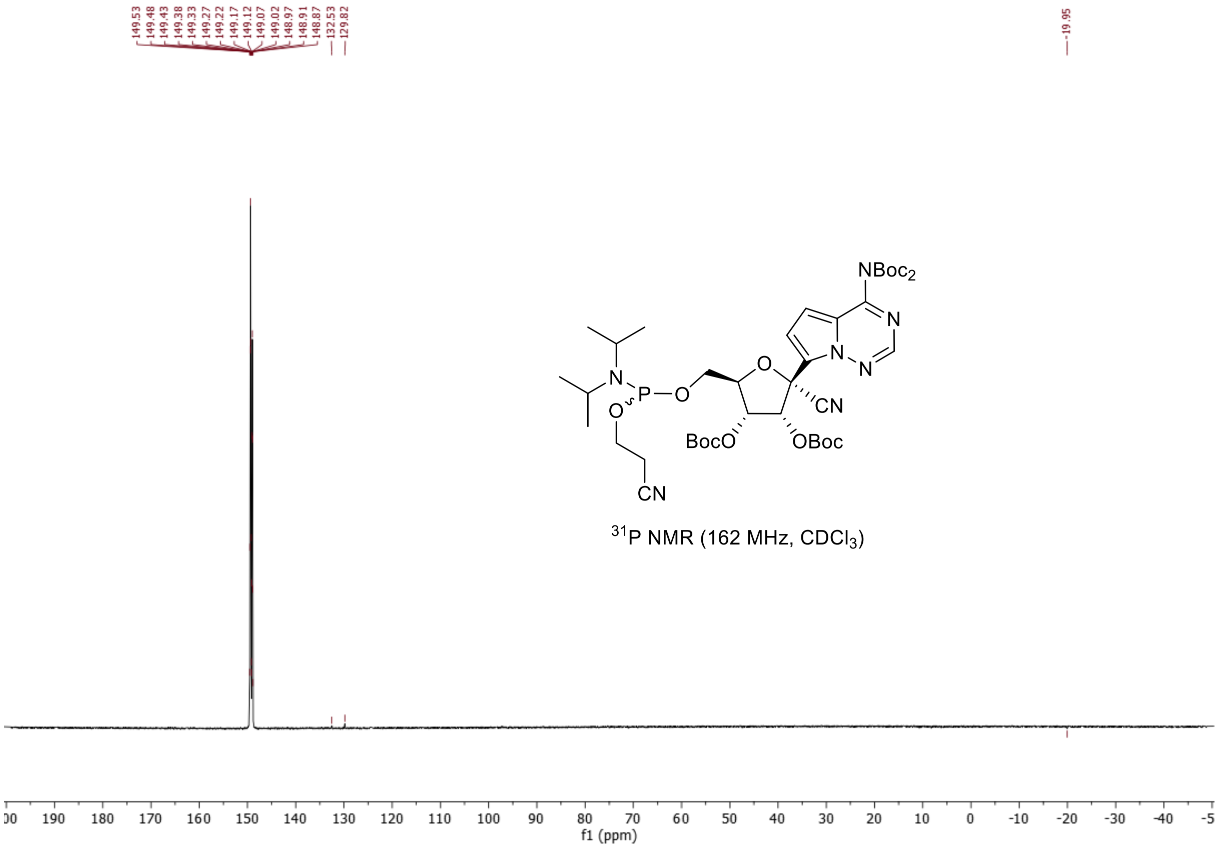

NBoc$_2$
N
O
P
O
CN
BocO
OBoc
CN
^{31}P NMR (162 MHz, CDCl$_3$)
f1 (ppm)

Compound **10**

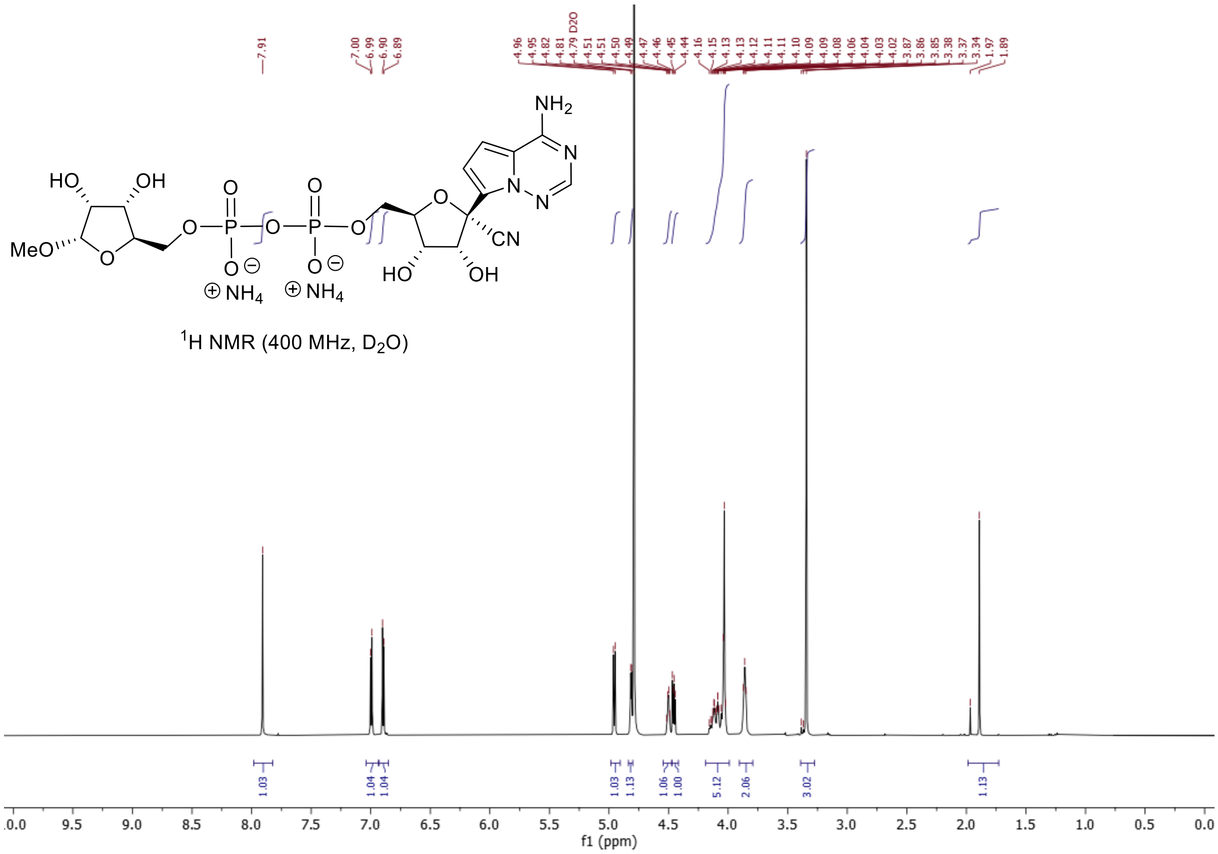

NH$_2$
HO
OH
MeO
O
P
O
NH$_4$
CN
HO
OH
^{1}H NMR (400 MHz, D$_2$O)
f1 (ppm)

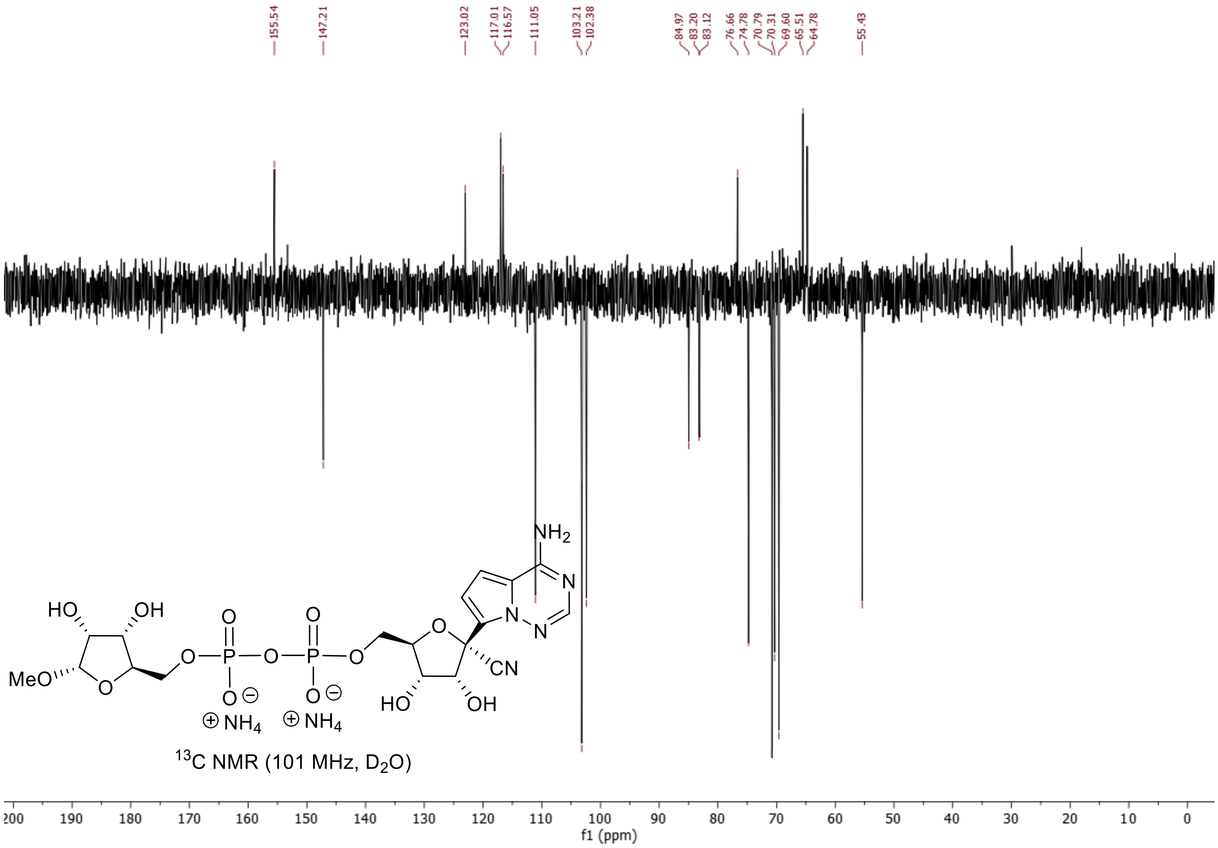
155.54
147.21
123.02
117.01
116.57
111.05
103.21
102.38
84.97
83.20
83.12
76.66
74.78
70.79
70.31
69.60
65.51
64.78
55.43
NH2
HO
OH
MeO
O
P
O⊖
⊕NH4
CN
N
13C NMR (101 MHz, D2O)
f1 (ppm)

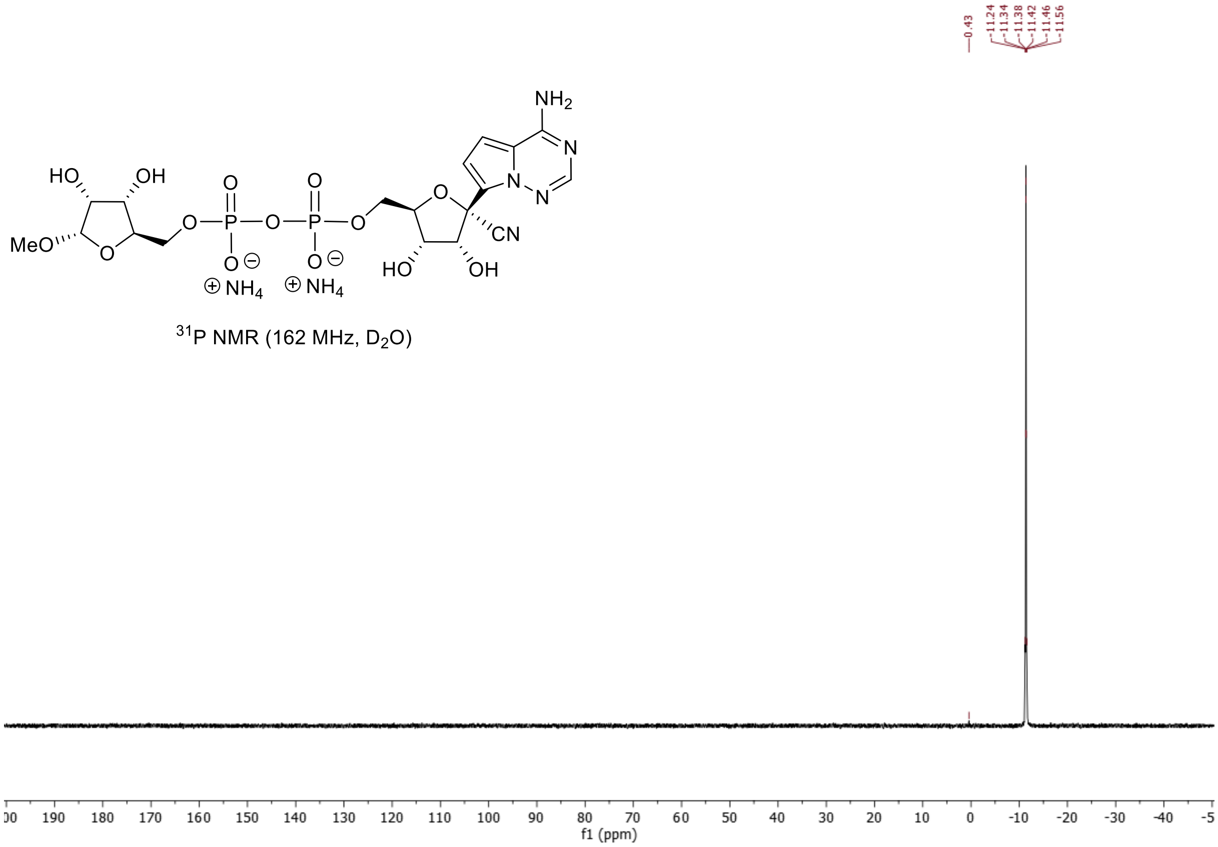
0.43
-11.24
-11.34
-11.38
-11.42
-11.46
-11.56
NH2
HO
OH
MeO
O
P
O⊖
⊕NH4
CN
N
31P NMR (162 MHz, D2O)
f1 (ppm)

Compound **11**

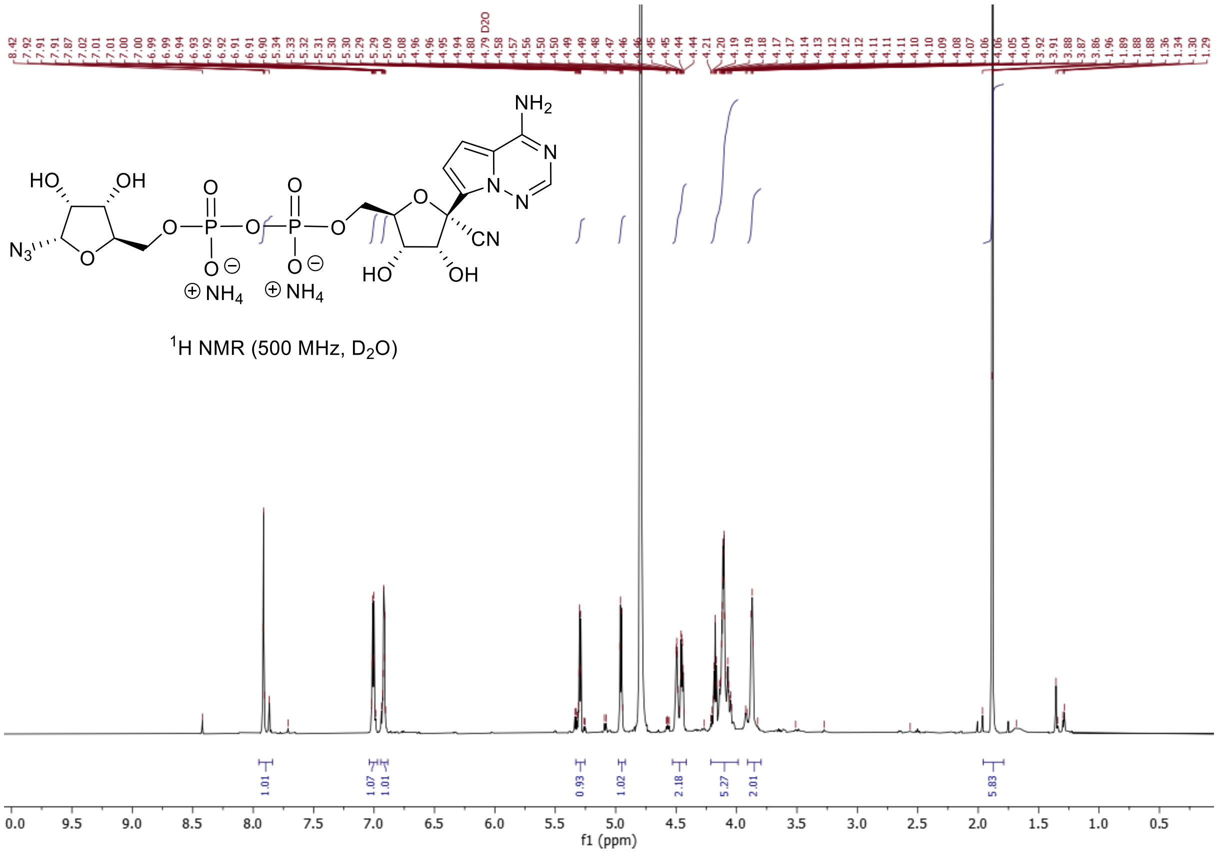

NH2
HO
OH
N3
O
P
O
NH4
CN
1H NMR (500 MHz, D2O)
f1 (ppm)

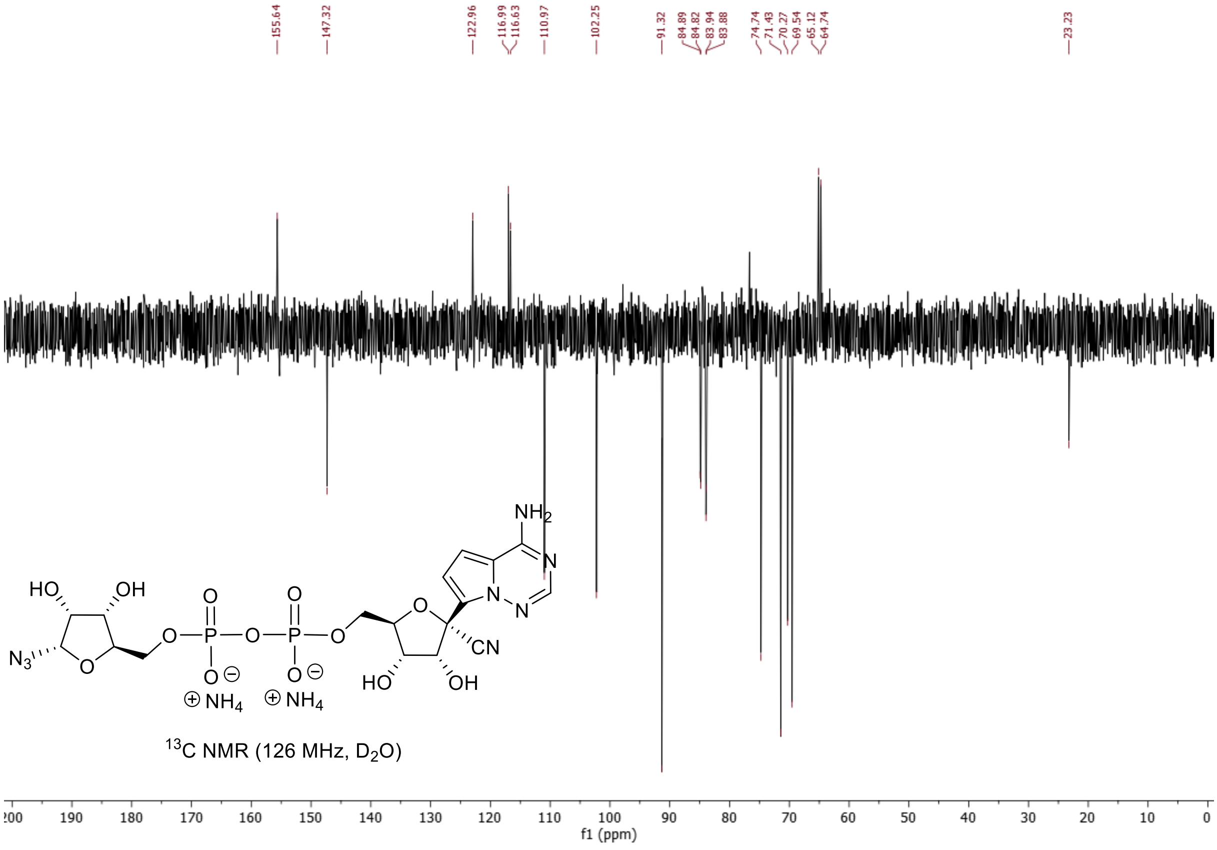

NH2
HO
OH
N3
CN
NH4
13C NMR (126 MHz, D2O)
f1 (ppm)

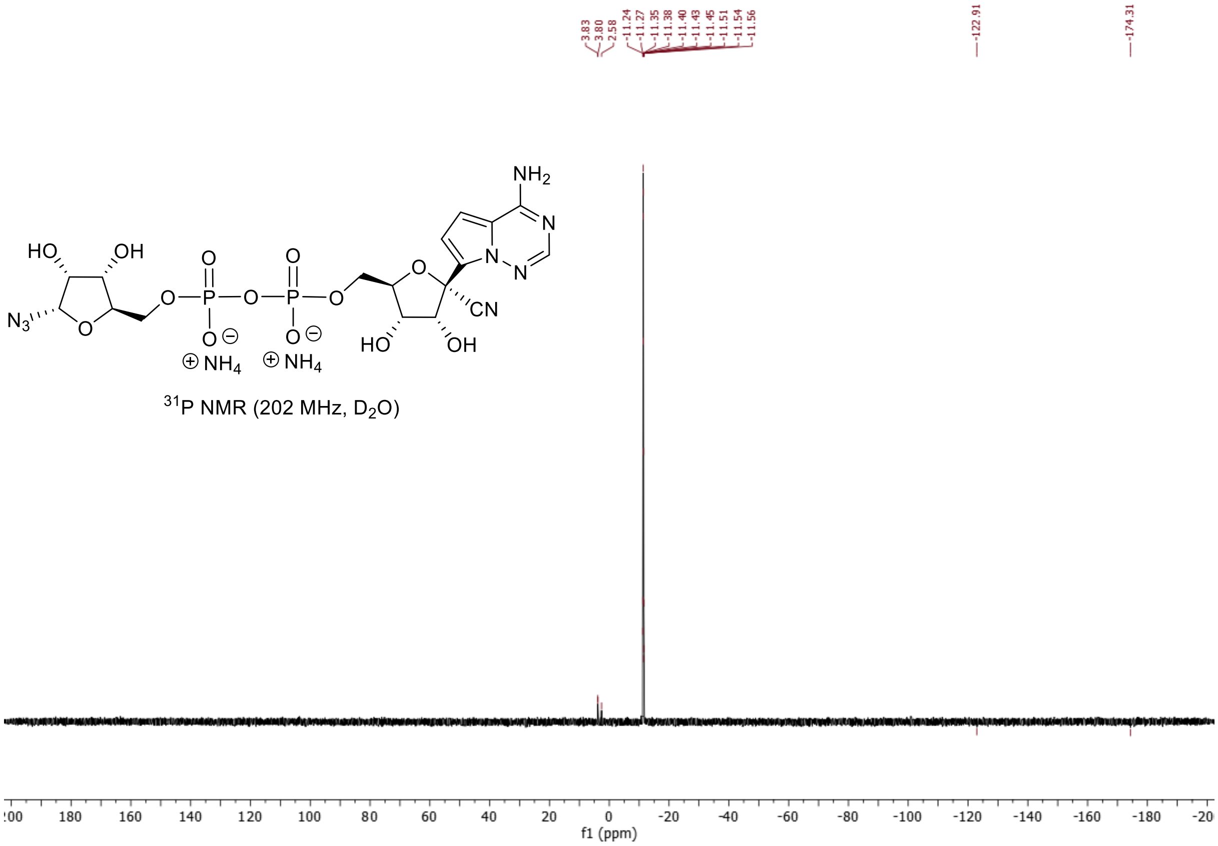

^{31}P NMR (202 MHz, D_2O)

Computational simulations

The NSP3 Mac1 high resolution crystal structure (PDB 7KQP, chain A) was used in all simulations. The waters 342, 366 and 479 were maintained during simulations. The ligands (10 and 11) were created based on the ADPr backbone from PDB 7KQP and the CAChe WorkSpace programme. Molecular docking was performed using Autodock4. For this, the receptor was maintained rigid and for ligands only hydroxyl, methyl and azide groups were allowed flexibility. The Lamarckian genetic algorithm was run 100 times for each ligand. For molecular dynamics simulation, ligand topology was generated by ACPYPE server (https://doi.org/10.1093/bioinformatics/btad350), atom charges were calculated using the AM1-BCC method (10.1002/jcc.10128) and all simulation were performed using the Gromacs 2020.5 version. For this, amber99SB-ildn (10.1002/prot.22711.) was selected as force field and TIP3P for the water model. In a dodecahedron box, Na^+ and Cl^- ions were added to neutralize the total charge in a final concentration of 140 mM (as NaCl). Initially, the system was minimized using 1000 kJ/mol/nm as limit force for up to 50,000 steps, followed by a NVT ensemble (for 100 ps at 310 K). Molecular dynamics data was obtained at the same temperature and pressure of 1 bar using the Parrinello-Rahman barostat (https://doi.org/10.1063/1.328693). After minimization, each system was simulated for 150 ns in triplicate (totalizing 450 ns by ligand). Hydrogen bond occupancy was calculated using the VMD 1.9.1 programme (https://www.ks.uiuc.edu/Research/vmd/) and binding enthalpy was calculated with the MMGBSA method available in the gmx_MMPBSA suite (https://valdes-tresanco-ms.github.io/gmx_MMPBSA/dev/installation/) at the same temperature.

Compound characterisation by Mac1 HTRF assay

Inhibition of SARS-CoV-2 NSP3 macrodomain was assessed by the displacement of an ADP-ribose conjugated biotin peptide (ARTK(Bio)QTARK(Aoa-RADP)S (Schuller ACS Chem Biol 2017) from His_6-tagged protein using a HTRF-technology-based screening assay which was performed as previously described (Schuller et al., 2021). IC_{50} curves were acquired with a maximum top compound concentration of 200 µM followed by an 8 point 1:1 dilution series in duplicate measurements or by 11 point 1:1 dilution series in triplicate measurements for confirmatory repeats (e.g. Compounds 11, 26 and 27). Compounds were dispensed into ProxiPlate-384 Plus (PerkinElmer) assay plates using an Echo 525 liquid handler (Labcyte). Binding assays were conducted in a final volume of 16 µl with 12.5 nM SARS-CoV-2 NSP3 macrodomain, 400 nM peptide, 1:20000 Anti-His_6-Eu^{3+} cryptate (HTRF donor, PerkinElmer) and 1:125 Streptavidin-XL665 (HTRF acceptor, PerkinElmer) in assay buffer (25 mM HEPES pH 7.0, 20 mM NaCl, 0.05% bovine serum albumin and 0.05% Tween-20). Macrodomain protein and peptide were first dispensed and incubated for 30 min at room temperature. This was followed by addition of the HTRF reagents and incubation at room temperature for 1 h. Fluorescence was measured using a PHERAstar microplate reader (BMG) using the HTRF module with dual emission protocol (A = excitation of 320 nm, emission of 665 nm, and B = excitation of 320 nm, emission of 620 nm). Raw data were processed to give an HTRF ratio (channel A/B × 10,000), which was used to generate IC50 curves. The IC_{50} values were determined by nonlinear regression using GraphPad Prism v.9 (GraphPad Software, CA, USA).

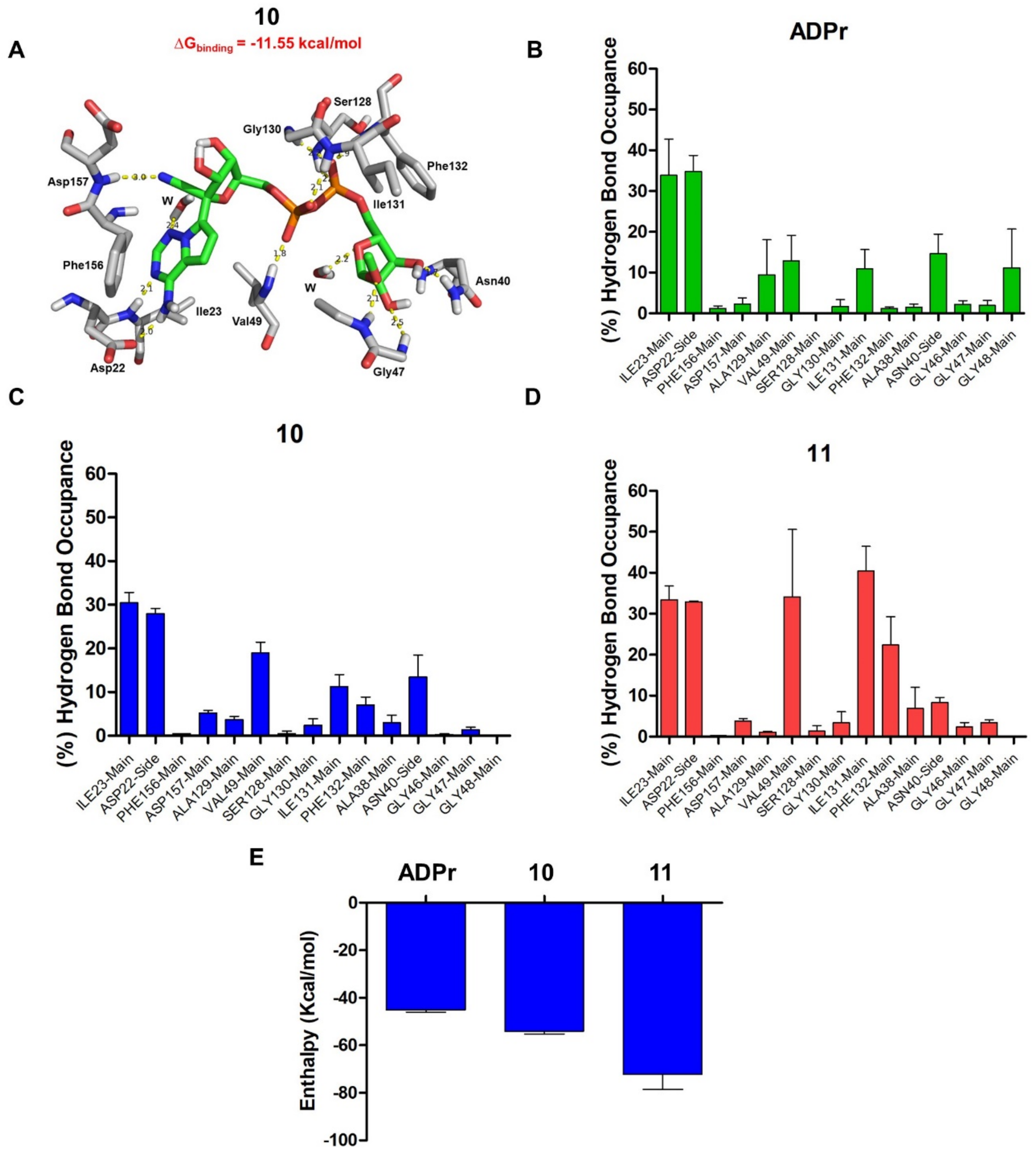

Figure S1. Molecular binding study for compounds **10** and **11** by computational docking into the Mac1 active site. **(A)** Molecular docking of **10** showing its binding mode as atom-coloured stick model. Hydrogen bond interactions are shown as yellow dashed lines. Hydrogen bond occupancy of ADPr **(B)**, **10 (C)** and **11 (D)**; hydrogen bond contributions are shown for each ligand-interacting residue of Mac1. Side: Side-chain interaction; Main: Main-chain interaction. **(E)** Binding enthalpy for ADPr, **10** and **11** obtained by molecular dynamics. Molecular docking was performed using Autodock4 and the PDB 7KQP Mac1 crystal structure.

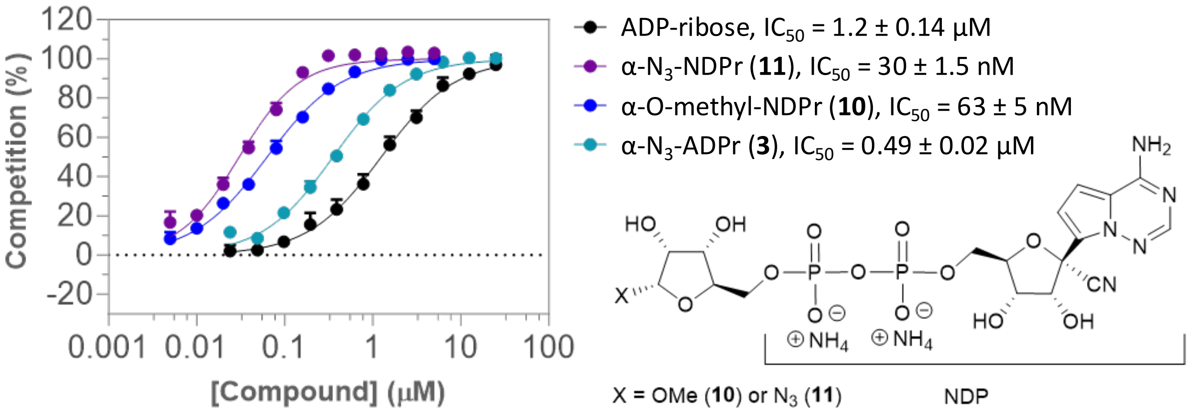

Figure S2. Biological evaluation of the NDPr-style inhibitors. As can be observed from the competition assay the RDPr compounds fare significantly better than the normal adenosine bearing analogues.

LCMS spectra

Compound **1**

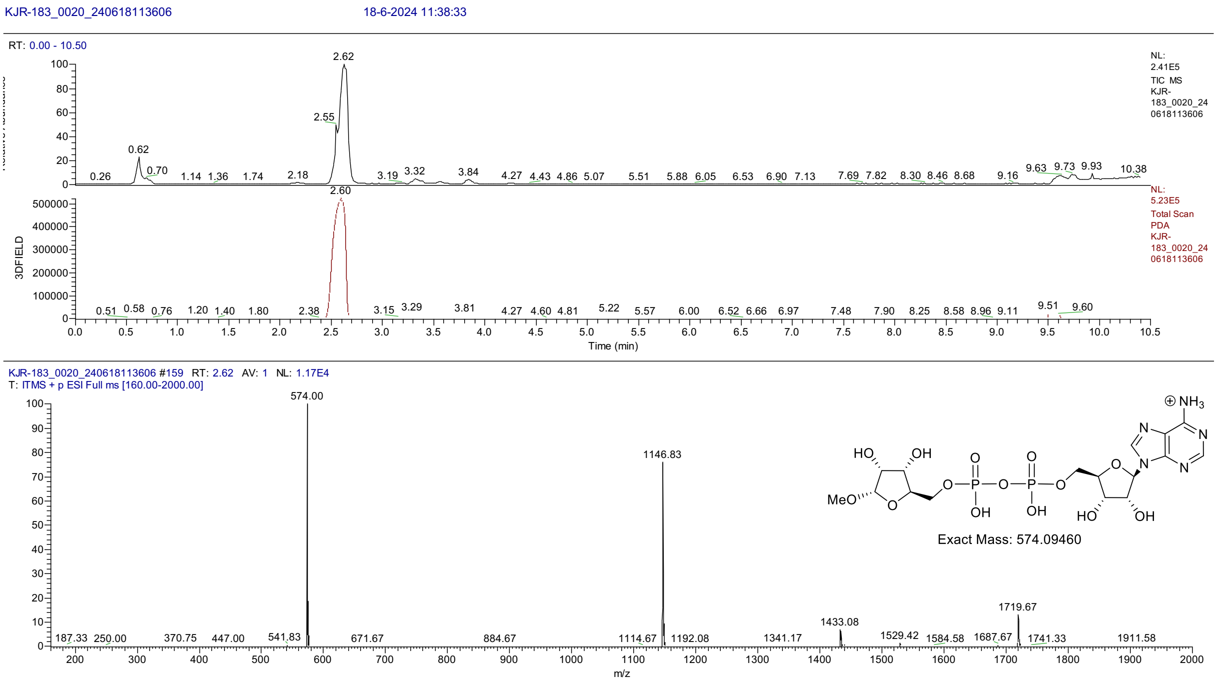

Compound **5**

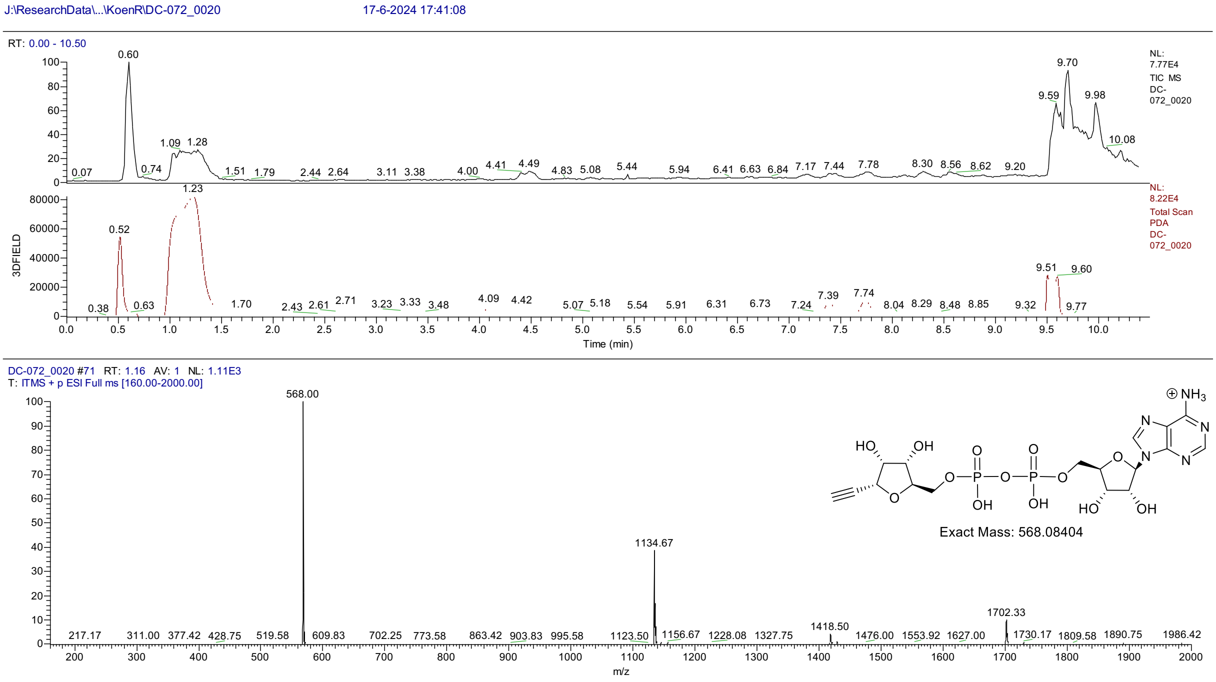

Compound **6**

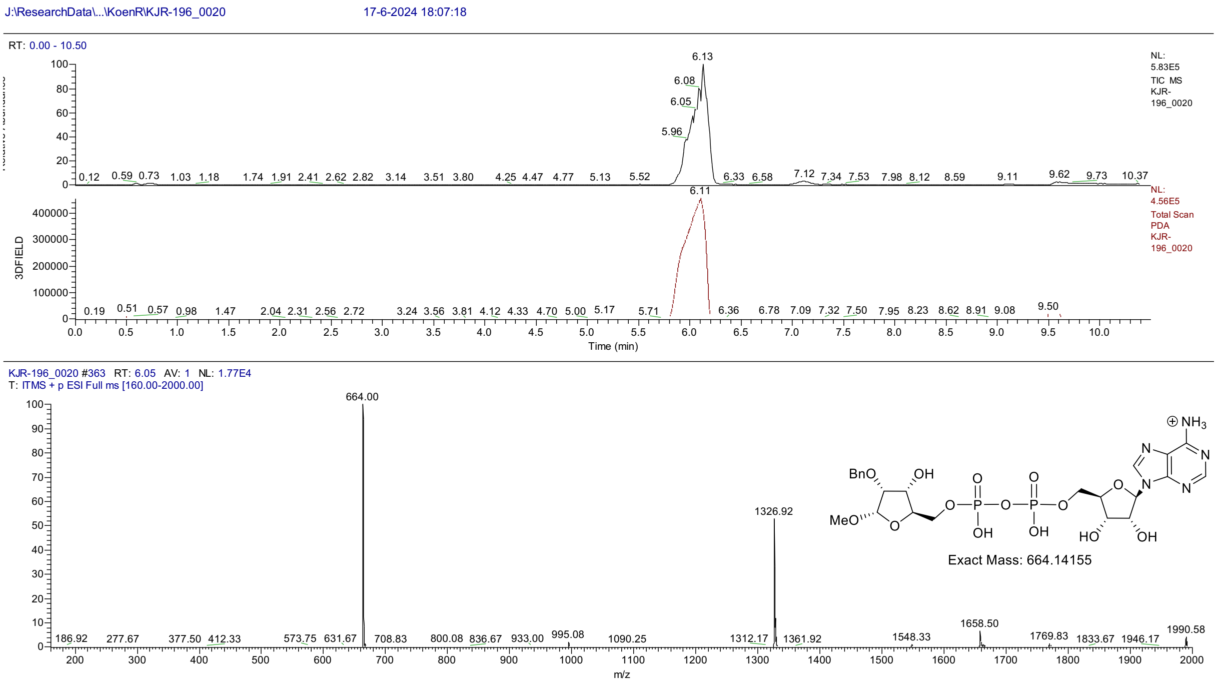

Compound **7**

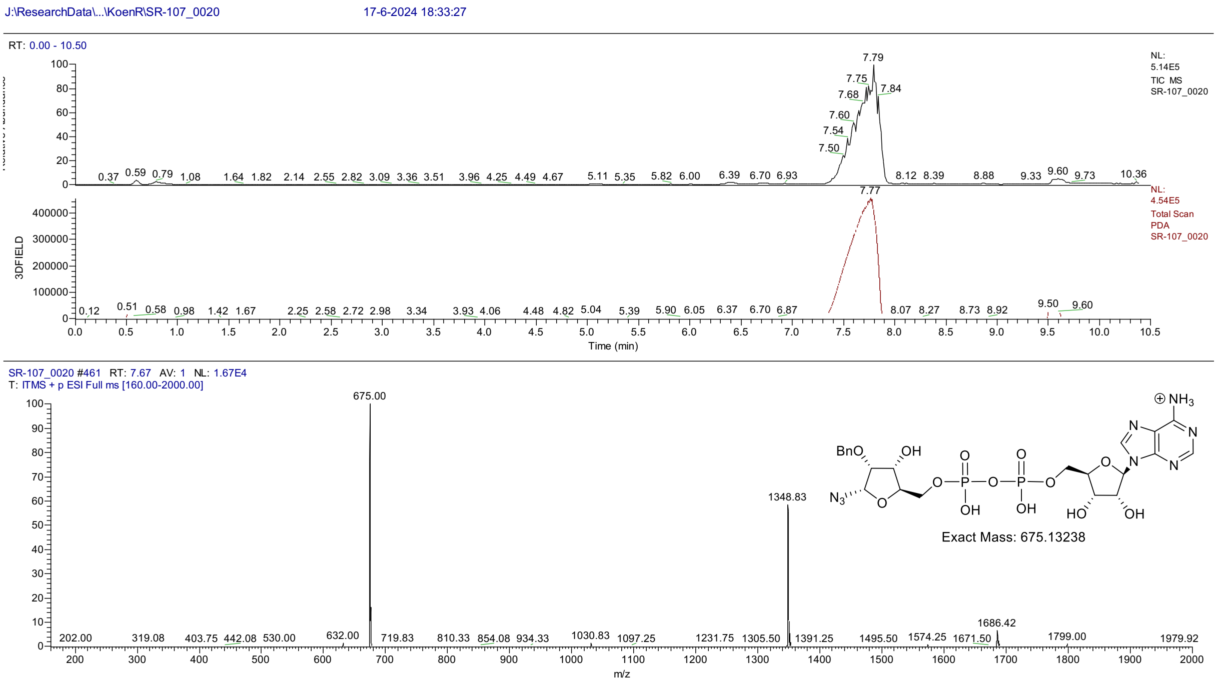

Compound **8**

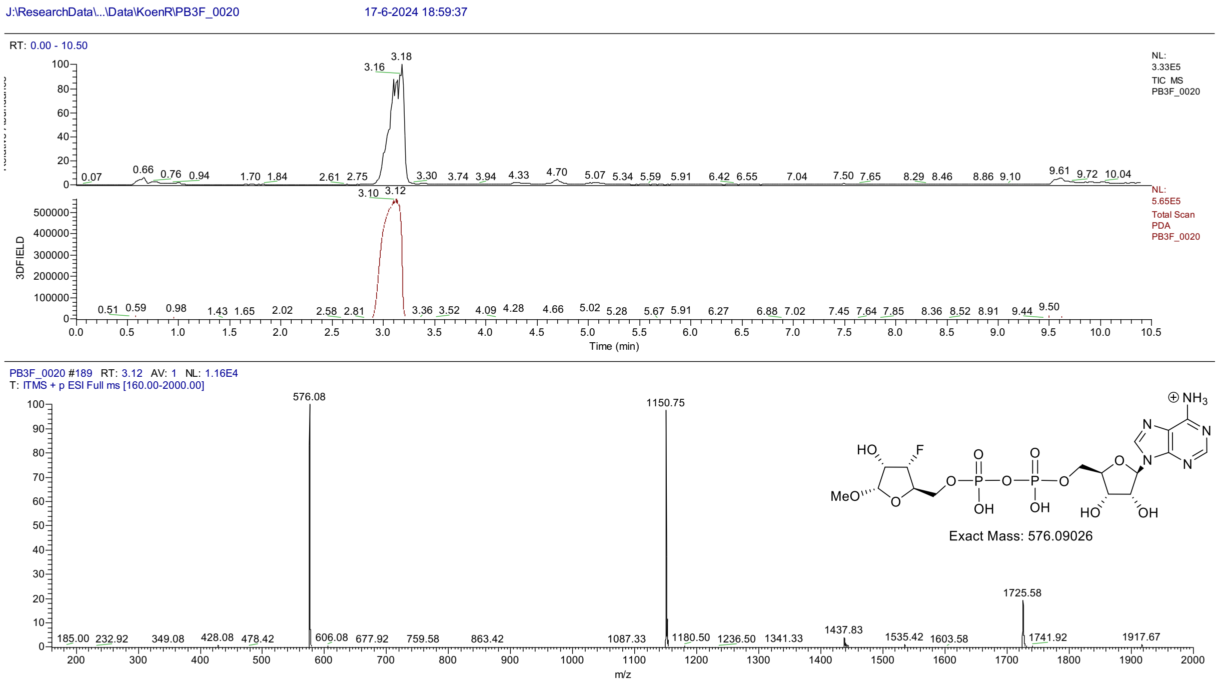

Compound **9**

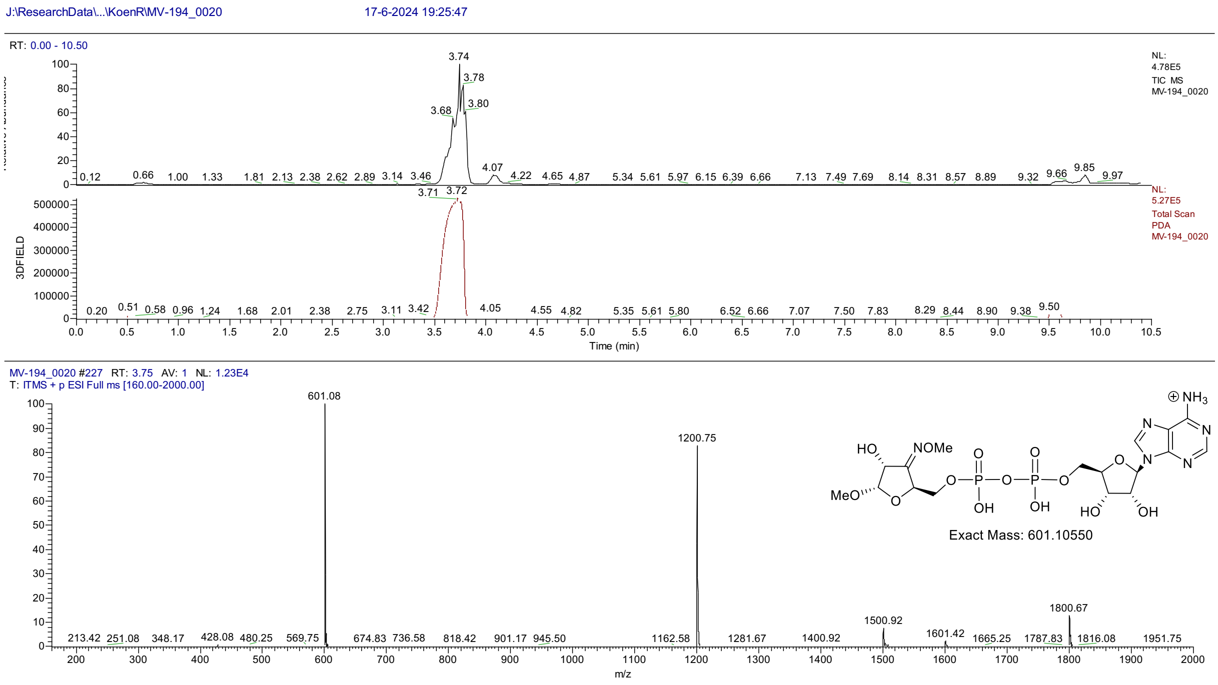

Compound **10**

J:\ResearchData\...\KoenR\KJR-439_0020 17-6-2024 19:51:56

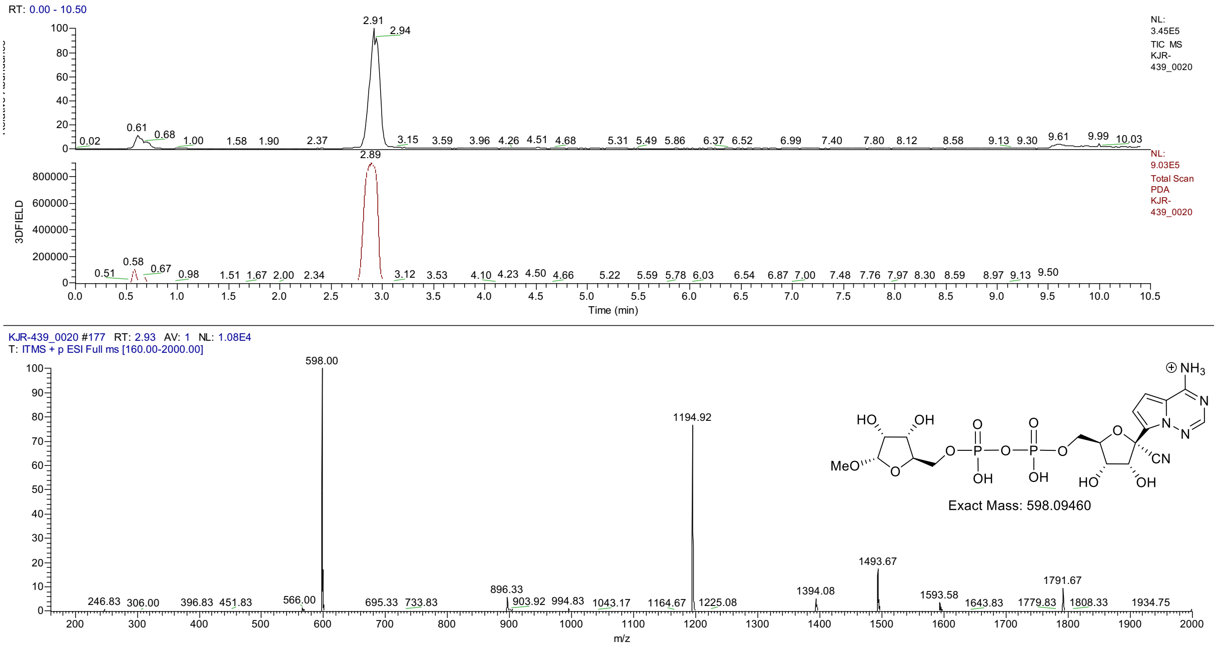

Compound **11**

KJR-440_0020_240618120208 18-6-2024 12:04:34

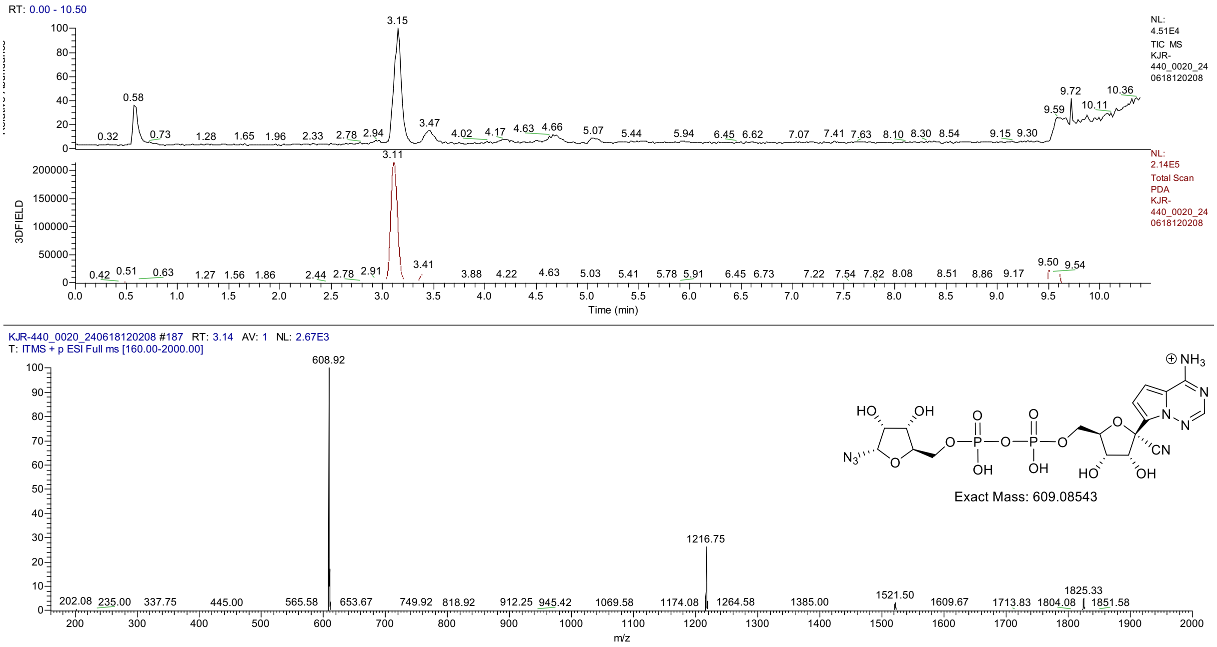

Supplement: Supplementary file 1 — ol4c01792_si_001.pdf [file ol4c01792_si_001.pdf]
